# Substrate-Controlled Divergent Synthesis of Benzimidazole-Fused Quinolines and Spirocyclic Benzimidazole-Fused Isoindoles

**DOI:** 10.1021/acs.joc.4c00164

**Published:** 2024-05-09

**Authors:** Ying-Ti Huang, Wan-Wen Huang, Yi-Ting Huang, Hong-Ren Chen, Indrajeet J. Barve, Chung-Ming Sun

**Affiliations:** † Department of Applied Chemistry, National Yang Ming Chiao Tung University, 1001 Ta-Hsueh Road, Hsinchu 300-10, Taiwan ROC; ‡ Department of Medicinal and Applied Chemistry, Kaohsiung Medical University, 100, Shih-Chuan first Road, Kaohsiung 807-08, Taiwan ROC; § Department of Chemistry, MES Abasaheb Garware College, Pune 411004, Maharashtra India

## Abstract

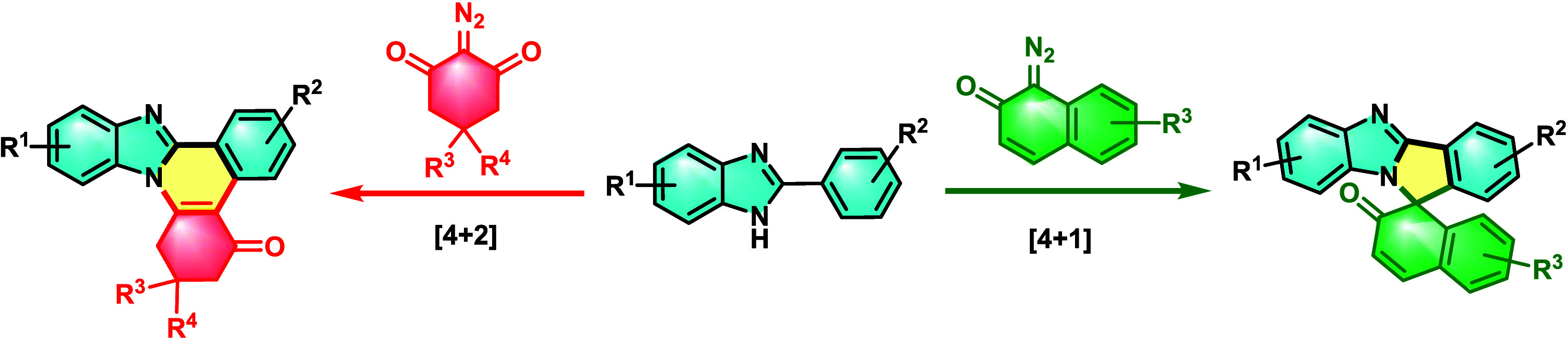

A Rh(III)-catalyzed annulation of 2-arylbenzimidazoles with α-diazo carbonyl compounds via C–H activation/carbene insertion/intramolecular cyclization is explored. The switchable product selectivity is achieved by the use of distinct α-diazo carbonyl compounds. Benzimidazole-fused quinolines are obtained through [4 + 2] annulation exclusively when 2-diazocyclohexane-1,3-diones are used, where they act as a C2 synthon. Alternatively, diazonaphthalen-1(2*H*)-ones merely function as a one-carbon unit synthon to generate a quaternary center through [4 + 1] cyclization to afford spirocyclic benzimidazole-fused isoindole naphthalen-2-ones. A thorough mechanistic study reveals the course of the reaction.

## Introduction

Diversity-oriented synthesis (DOS) is a strategy to synthesize a collection of skeletally or stereochemically distinct molecular scaffolds. This can accelerate the rate of exploration of biologically relevant chemical space and increase the chances of finding new chemical probes for various biological targets.^1^ A privileged-substructure-based DOS (p-DOS) strategy has been developed to accelerate this process further. This approach involves choosing a privileged substructure as a basic core and transforming it into different polyheterocyclic skeletons using various reagents, thus achieving skeletal diversity in a compound library.^2^

Diazo compounds are versatile precursors in modern synthetic organic chemistry. They are readily accessible and stable yet reactive as carbenes or metal–carbenoids. Under transition-metal catalysis, they can be transformed into metal–carbenoids. Therefore, α-diazo carbonyl compounds have been widely used as coupling partners for the construction of polycyclic heterocycles via a metal-catalyzed C–H activation/carbenoid insertion and cyclization approach.^3^

Benzimidazole is a privileged scaffold that is frequently found in many important drug candidates. Its derivatives exhibit a broad spectrum of bioactivities, such as anticancer, anti-inflammatory, antimicrobial, antibacterial, anti-human immunodeficiency virus (HIV), and antimalarial.^4^ Considering the usefulness of the benzimidazole framework, we chose 2-aryl benzimidazole as the core scaffold in this study as it has attracted widespread interest. We envisioned that the benzimidazole core could be transformed into benzimidazole-fused quinolines and spirocyclic benzimidazoles by reacting it with α-diazo carbonyl compounds such as 2-diazocyclohexane-1,3-diones or diazonaphthalen-1(2*H*)-ones through a transition-metal catalysis using the p-DOS strategy.

The literature has revealed a few pioneering works on the synthesis of spiro molecules and benzimidazole-fused quinolines. For example, Zhou demonstrated a strategy for the synthesis of spirocyclic indazole derivatives via Rh(III)-catalyzed C–H activation and spiroannulation.^5^ Guo et al. reported a [4 + 1] spiroannulation between isoquinolones and diazo compounds to access spirocyclic indazole compounds.^6^ Yang successfully coupled 2-(2-bromophenyl)-1*H*-benzo[*d*]imidazoles with 1,2-diketones by a copper catalyst through arylation/cyclocondensation.^7^ Dong disclosed a copper-catalyzed organic ligand-promoted coupling reaction between 2-(2-bromophenyl)-1*H*-benzo[*d*]imidazoles and cyclohexane-1,3-diones via an α-arylation/intramolecular nucleophilic addition cascade for the synthesis of benzimidazole-fused quinolines (Scheme )1^8a^. Zhong developed a strategy for the synthesis of benzimidazole-fused quinolines via Rh(III)-catalyzed annulation of 2-aryl benzimidazole and 1,3-dicarbonyl compounds.^8b^ Nunewar disclosed a Ru(II)-catalyzed reaction between 2-arylbenzimidazoles and iodonium ylides for the preparation of benzimidazole-fused quinolines.^8c^ Despite the elegance of the reported works, there is a high demand for a direct and operationally simple strategy to construct these significant frameworks. To the best of our knowledge, there has not been reported to date an [4 + 1] or [4 + 2] annulation reaction between 2-aryl benzimidazole and diazo carbonyl compounds.

**Scheme 1:**
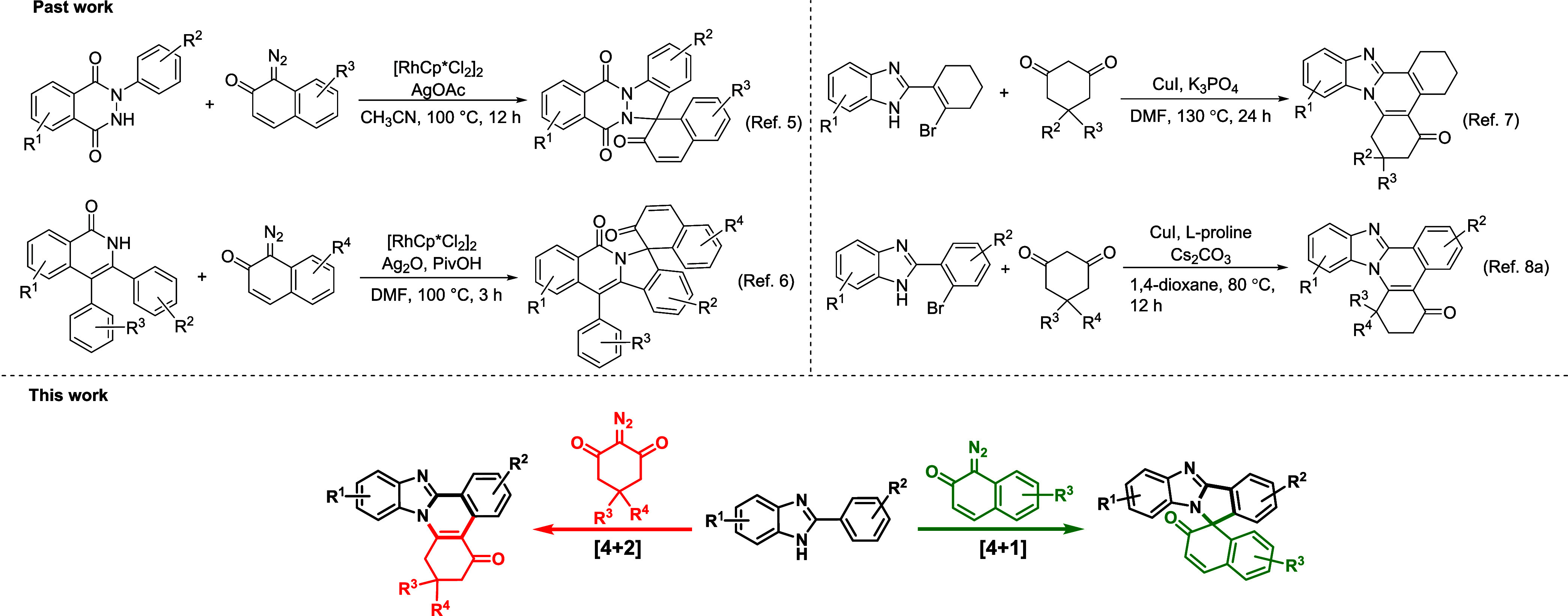
Various Approaches for the Synthesis of Spirocyclic Benzimidazoles and Benzimidazole-Fused Quinolines

Herein, we report the substrate-controlled synthesis of benzimidazole-fused quinolines (4 + 2) and spiro benzimidazole-fused isoindole naphthalen-2-ones (4 + 1) by rhodium-catalyzed C–H activation/annulation of 2-arylbenzimidazoles and 2-diazocyclohexane-1,3-diones or diazonaphthalen-1(2*H*)-ones.

## Results and Discussion

**Figure 1:**
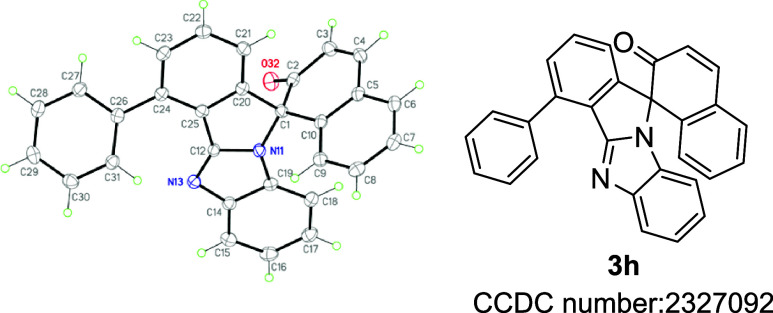
ORTEP diagram of 4-phenyl-2′*H*-spiro[benzo[4,5]imidazo[2,1-*a*]isoindole-11,1′-naphthalen]-2′-one **3h**.

We initially selected 2-(*m*-tolyl)-1*H*-benzo[*d*]imidazole **1d** and 1-diazonaphthalen-2(1*H*)-one **2a** as the model substrates to optimize the reaction conditions. To optimize the reaction conditions, we varied the catalyst, oxidant, solvent, and temperature (Table 1). Only the starting materials were recovered when we reacted **1d** with **2a** in the presence of Co(OAc)_2_, Pd(OAc)_2_, or [RuCl_2_(p-cymene)]_2_ (2.5 mol %) and AgOAc (2 equiv) as an oxidant in DCE at 80 °C for 15 h (Table 1, entries 1–3). However, a new spot on thin-layer chromatography (TLC) was observed and isolated in a 20% yield along with the starting materials when we performed the same reaction with [IrCp*Cl_2_]_2_ (Table 1, entry 4). The structure of the isolated compound was confirmed as the benzimidazole-fused isoindole **3d** by a standard set of characterization data analysis. Two doublets at 8.13 and 6.48 ppm corresponding to the protons of the C=C double bond of the naphthalen-2(1*H*)-one moiety were observed in the proton NMR spectrum. In the ^13^C NMR spectrum, peaks corresponding to the carbonyl group and spiro carbon were observed at 193.8 and 73.7 ppm, respectively. The structure of representative compound **3h** was unambiguously established by X-ray crystallography (Figure 1). The ORTEP diagram of **3h** showed that the isoindole and benzimidazole moieties are almost perpendicular to each other due to a spirocyclic carbon atom, and the overall structure is spatially three-dimensional. The formation of this product could be rationalized by a rhodium-catalyzed C–H activation of the benzimidazole ring, followed by a [4 + 1] annulation with the diazonaphthalenone. Use of the catalyst [RuCp*Cl_2_]_2_ slightly improved the yield of **3d** (32%) (Table 1, entry 5). We found that [RhCp*Cl_2_]_2_ was the only suitable catalyst for the [4 + 1] annulation reaction as compared to other catalysts (Table 1, entry 6).

**Table 1:**
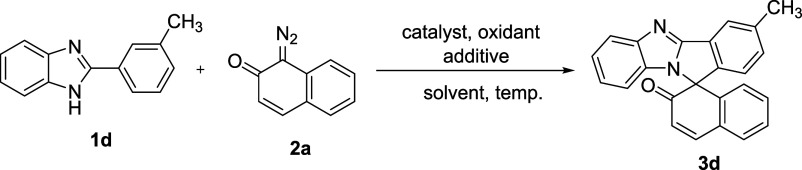
Optimization of Reaction Conditions^a^

entry	catalyst	oxidant	additive	solvent	*T* (°C)	yield (%)^b^
1	Co(OAc)_2_	AgOAc (2)		DCE	80	0
2	Pd(OAc)_2_	AgOAc (2)		DCE	80	0
3	[RuCl_2_(p-cymene)]_2_	AgOAc (2)		DCE	80	0
4	[IrCp*Cl_2_]_2_	AgOAc (2)		DCE	80	20
5	[RuCp*Cl_2_]_2_	AgOAc (2)		DCE	80	32
6	[RhCp*Cl_2_]_2_	AgOAc (2)		DCE	80	56
7	[RhCp*Cl_2_]_2_	CuO (2)		DCE	80	0
8	[RhCp*Cl_2_]_2_	Cu(OAc)_2_ (2)		DCE	80	39
9	[RhCp*Cl_2_]_2_	Ag_2_O (2)		DCE	80	38
10	[RhCp*Cl_2_]_2_	Ag_2_CO_3_ (2)		DCE	80	42
11	[RhCp*Cl_2_]_2_	AgSbF_6_ (2)		DCE	80	0
12	[RhCp*Cl_2_]_2_	AgOAc (4)		DCE	80	74
13	[RhCp*Cl_2_]_2_	AgOAc (4)		CH_3_CN	80	0
14	[RhCp*Cl_2_]_2_	AgOAc (4)		toluene	80	45
15	[RhCp*Cl_2_]_2_	AgOAc (4)		DMF	80	0
16	[RhCp*Cl_2_]_2_	AgOAc (4)		benzene	80	55
17	[RhCp*Cl_2_]_2_	AgOAc (4)		1,2-dioxane	80	20
18	[RhCp*Cl_2_]_2_	AgOAc (4)	AcOH	DCE	100	70
19	[RhCp*Cl_2_]_2_	AgOAc (4)	PivOH	DCE	80	67
20	[RhCp*Cl_2_]_2_	AgOAc (4)	NaOAc	DCE	80	71
21	[RhCp*Cl_2_]_2_	AgOAc (4)	Et_3_N	DCE	80	32
22	[RhCp*Cl_2_]_2_	AgOAc (4)	AcOH	DCE	80	**81**

a Reaction conditions: **1d** (0.24 mmol, 1 equiv), **2a** (0.26 mmol, 1.1 equiv), [RhCp*Cl_2_]_2_ (2.5 mol %), additive (2 equiv), solvent (5 mL), 80 °C, and 15 h.

b Isolated yield.

We then screened different oxidants, such as CuO, Cu(OAc)_2_, Ag_2_O, Ag_2_CO_3_, and AgSbF_6_ (Table 1, entries 7–11). We found that copper-based oxidants were ineffective, while AgOAc was a better choice among the silver-based oxidants. Then, we increased the amount of AgOAc to 4 equiv, and the yield of **3d** improved to 74% (Table 1, entry 12). Next, we assessed the effect of solvents such as CH_3_CN, toluene, DMF, benzene, and 1,4-dioxane and found that DCE was an ideal solvent (Table 1, entries 13–17). After that, we added AcOH as an additive at 100 °C and obtained the desired product in a 70% yield (Table 1, entry 18). The use of other additives, such as PivOH, NaOAc, and Et_3_N, indicated that AcOH was superior to these additives (Table 1, entries 19–21). The optimization study indicated that [RhCp*Cl_2_]_2_ (2.5 mol %), AcOH, and AgOAc (4 equiv) in DCE at 80 °C for 15 h was the optimal reaction condition and **3d** was obtained in an 81% yield (Table 1, entry 22).

With the optimized reaction conditions, we next explored the substrate scope and generality of this exclusive [4 + 1] annulation reaction (Table 2). Initially, we tested 2-arylbenzimidazoles bearing various substituents on the aryl ring. Nonsubstituted 2-aryl benzimidazole and substrates bearing −CH_3_ or −CF_3_ groups at the *para* position afforded the desired products (**3a**, **3b**, and **3c**) in lower yields. Substrates with the same substituents at the *meta* position on the aryl ring smoothly reacted with **2a** to give **3d** and **3e** in 81, and 78% yields, respectively. *Ortho-*substituted substrates afforded the corresponding products **3f** (70%) and **3g** (80%) in good yields. Notably, the substrate having a bulky substituent (-Ph) at the *ortho* position afforded **3h** in a 75% yield, indicating that the steric hindrance was well tolerated. The halogen substituents (–F, –Cl, and –Br) present at the *ortho* position on the aromatic ring of the 2-aryl benzimidazole were tolerated, and the corresponding products **3j** (67%), **3k** (64%), and **3l** (57%) were formed in moderate yields. The reaction of 2-(2,3-dimethoxyphenyl)-1*H*-benzo[*d*]imidazole **1m** with **2a** proceeded smoothly to give **3m** in a 74% yield. 2-Naphthyl benzimidazole **1n** reacted smoothly with **2a** to give **3n** in an 80% yield. Next, we investigated the scope of substrates with substituents on the benzimidazole ring. Accordingly, when 2-arylbenzimidazoles bearing –Cl, –Me, and –NO_2_ groups reacted with **2a**, the positional isomers **3o**(**3o′)**, **3p(3p′)**, and **3q(3q′)** were obtained in a 1:1 ratio. The dimethyl-substituted substrate **1r** furnished the desired product **3r** (85%) in an excellent yield. We also examined the substrate scope of various diazonaphthalen-1(2*H*)-ones. Bromo-substituted diazo compounds **2s** and **2t** gave products **3s** and **3t** in 78, and 79% yields, respectively, showing great compatibility toward the standard reaction conditions. Diazonaphthalen-1(2*H*)-ones bearing electron-donating or electron-withdrawing groups on the aromatic ring were compatible with the optimized reaction conditions and gave **3u** (77%) and **3v** (80%) in good yields. To expand the reaction scope further, other heterocycles such as 2-thienyl benzimidazole, 2-*N*-substituted pyrrole, and 2-phenyl imidazole were reacted with diazonaphthalen-1(2*H*)-one, but, in all of the cases the desired products were not obtained, and the starting materials were recovered (see Supporting Information, S4).

**Table 2:**
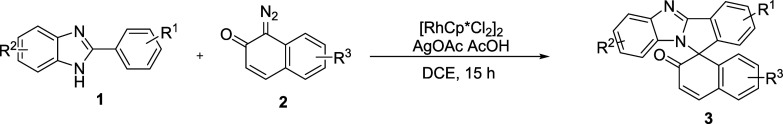

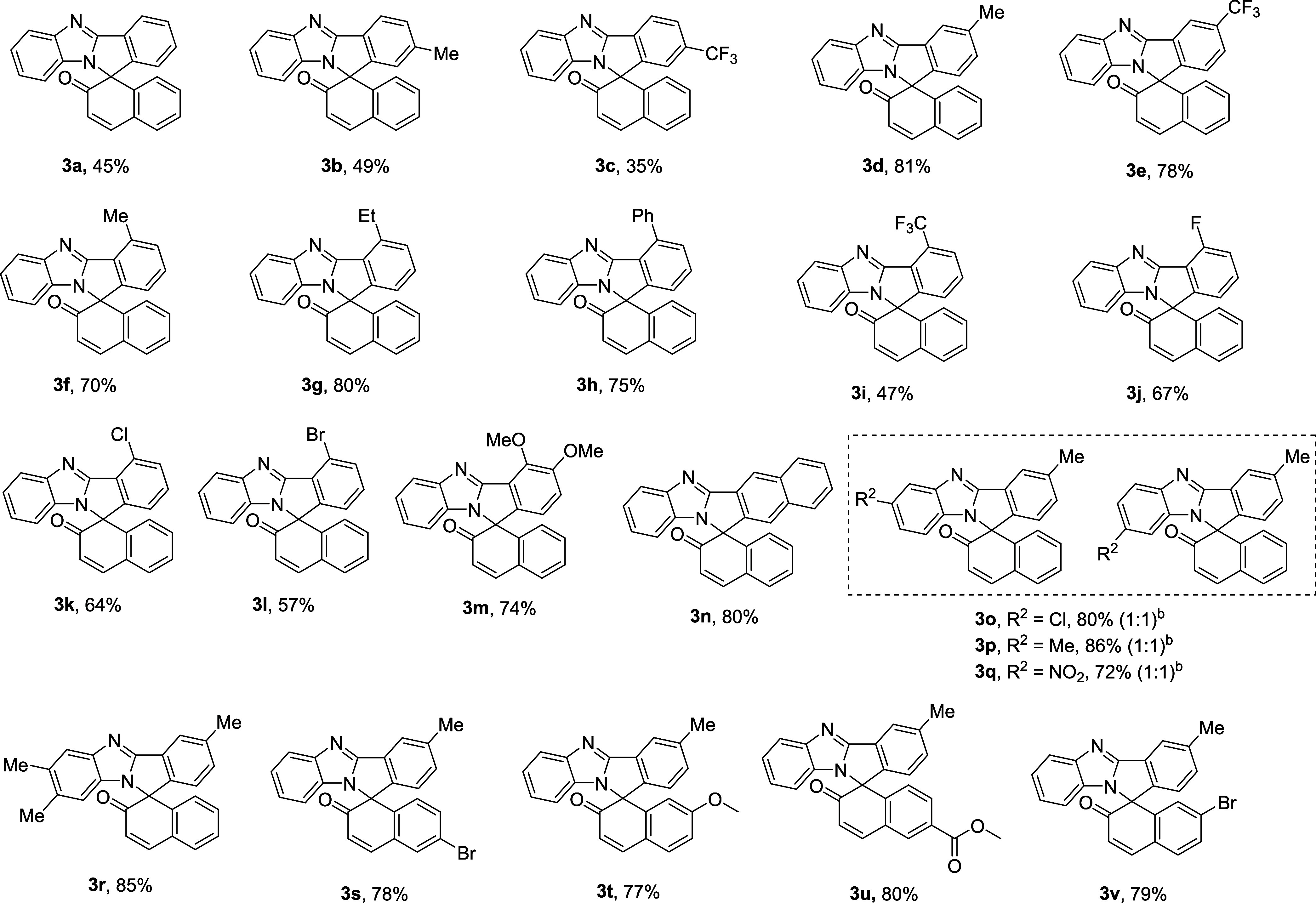
Substrate Scope for the Synthesis of Spirocyclic Benzimidazole-Fused Isoindoles **3**^a^

a Reaction conditions: **1** (1 equiv), **2** (1.1 equiv), [RhCp*Cl_2_]_2_ (2.5 mol %), AgOAc (4 equiv), AcOH (2 equiv), DCE (5 mL), 80 °C, and 15 h.

b Isomer ratio was determined by ^1^H NMR analysis of the crude mixture.

To further extend the diversity within the framework of the p-DOS strategy, we reacted 2-aryl benzimidazole **1a** with 2-diazocyclohexane-1,3-dione **4a** under various reaction conditions. Different catalyst and additive screening results revealed that [RhCp*Cl_2_]_2_ and AgOTf were suitable to synthesize benzimidazole-fused quinolines **5** through [4 + 2] annulation. Further solvent screening established the optimized reaction conditions as [RhCp*Cl_2_]_2_ (5 mol %) and AgOTf (20 mol %) in DMF at 100 °C in a sealed tube for 6 h for the current transformation (See S3).

**Figure 2:**
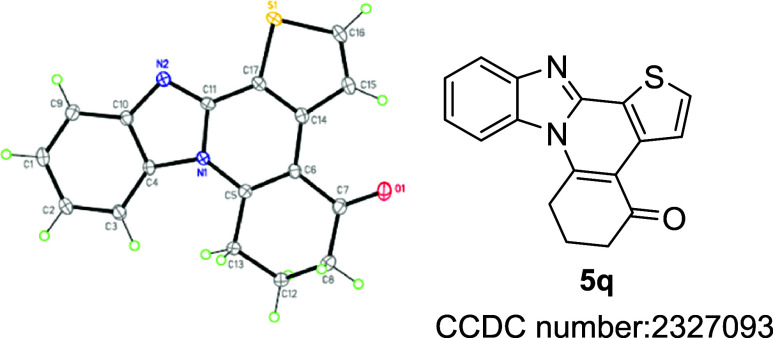
ORTEP diagram of **5q**.

Once the optimized condition was determined, the substrate scope for this [4 + 2] annulation reaction was investigated (Table 3). Initially, 2-aryl benzimidazole **1** with various substituents at *ortho*, *meta*, and *para* positions on the phenyl ring were reacted with **4a**. Accordingly, substrate **1** possessing electron-donating groups (*R*^2^ = –Me and –Et) afforded **5b** (73%) and **5c** (80%) in good yields. We obtained **5d** in an 84% yield from the chloro-substituted benzimidazole and **5e** in a 68% yield from the trifluoromethyl-substituted benzimidazole. The phenyl-substituted benzimidazole gave **5f** in a 90% yield. Next, we examined the effect of EDGs (*R*^2^ = CH_3_, OCH_3_) and EWGs (*R*^2^ = CF_3_) at the *meta* position on the aryl ring. We obtained **5g**, **5h**, and **5i** in 90, 94, and 70% yields at a less sterically hindered site in a regioselective manner. Next, we explored the effect of EDGs and EWGs at the *para* position on the aryl ring. We obtained **5j**, **5k**, **5l**, and **5m** in 88, 80, 75, and 80% yields, respectively. The *para* substitution did not strongly affect the C–H activation. After that, the scope of substrate **1** bearing different substituents on the benzimidazole core was explored. The methyl- and chloro-substituted benzimidazoles gave **5n** and **5o** in 81, and 78% yields, respectively. We tested benzimidazoles with heterocyclic rings other than the phenyl ring at the 2-position. The furyl-substituted benzimidazole did not give any product, probably due to the electron-withdrawing nature of oxygen to deactivate the C–H reaction site. However, the 2-thienyl, 2-*N*-substituted pyrrole, and 2-naphthyl benzimidazoles smoothly participated in the reaction to give **5q**, **5r**, and **5s** in 80, 82, and 89% yields. The exact structure of **5q** was confirmed by X-ray crystallography (Figure 2). The X-ray structure showed that the thiophene moiety is partially perpendicular to the benzimidazole moiety, and the whole structure is nonflat.

**Table 3:**
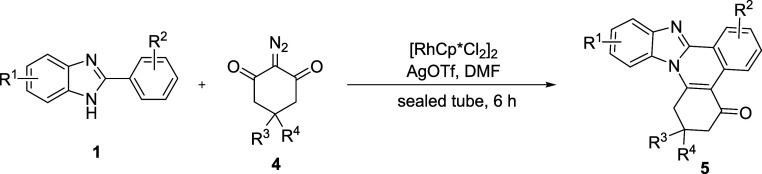

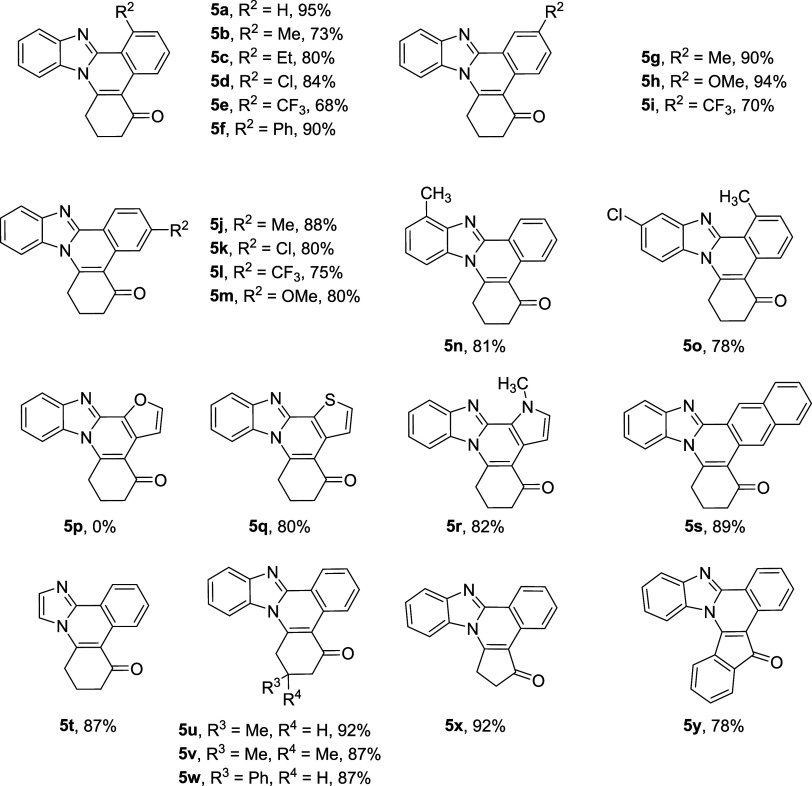
Synthesis of Benzimidazole-Fused Quinolines **5**^a^

a Reaction conditions: **1** (1 equiv), **4** (1.2 equiv), [RhCp*Cl_2_]_2_ (5 mol %), AgOTf (20 mol %), DMF (3 mL), sealed tube, 100 °C, and 6 h

**Scheme 2:**
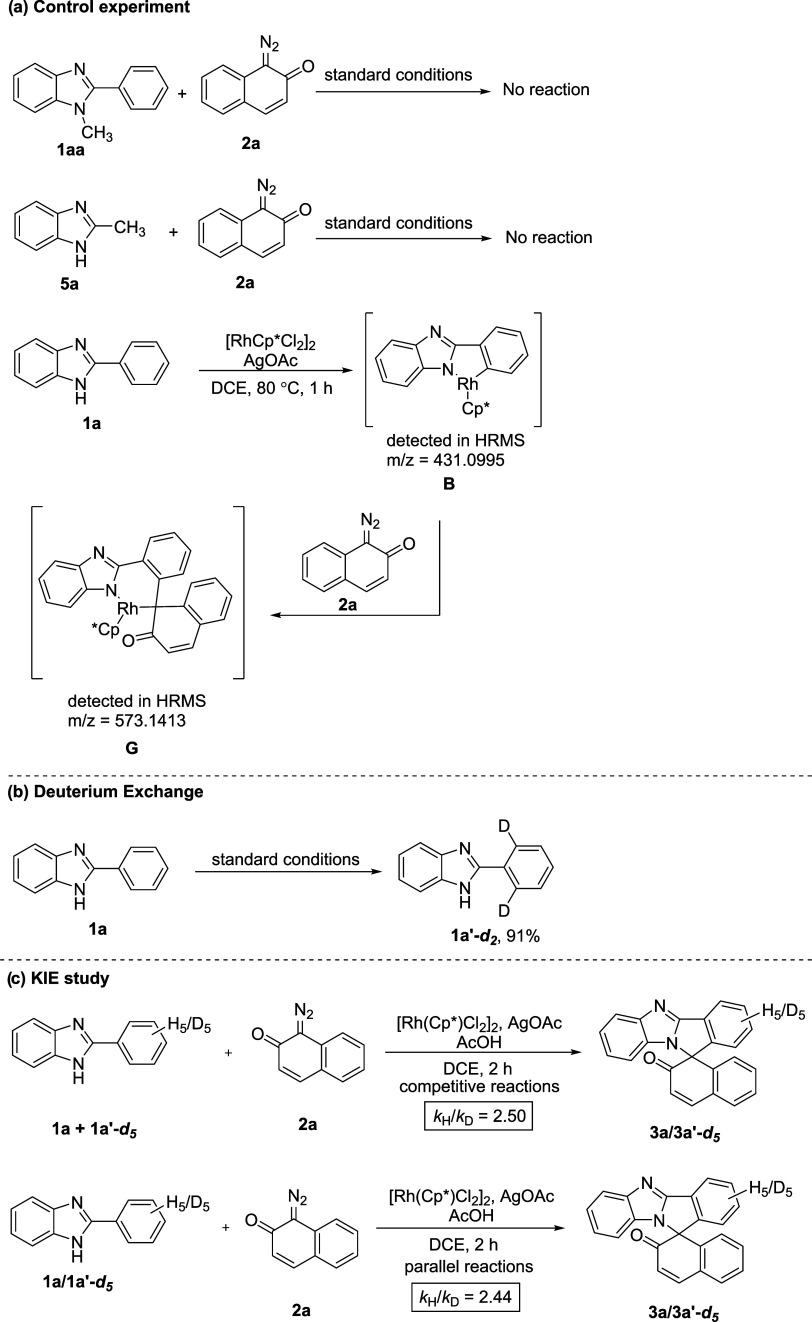
Mechanistic Study for the Formation of [4 + 1] Adduct **3**

It is worth noting that this metal-catalyzed reaction is not limited to the benzimidazole core only. Accordingly, 2-aryl imidazole **1t** reacted smoothly with **4a**, delivering **5t** in an 87% yield. Then, we explored the scope of DCHD bearing different substituents. We obtained **5v** and **5w** from DCHD bearing methyl and bulky phenyl substituents in an 87% yield, respectively. Next, we used 2-diazocyclopentane-1,3-dione (DCPD) and obtained product **5x** in a 92% yield. After that, **5y** was obtained in a 78% yield when 2-diazo-1*H*-indene-1,3(2*H*)-dione was reacted with **2a**. Finally, we conducted a series of control and deuterium-labeling experiments to comprehend the mechanistic details of this coupling reaction (Scheme 2). *N*-Methyl benzimidazole **1aa** did not react with **2a** under standard reaction conditions, indicating that the free NH is required for the coordination of the metal catalyst. When 2-methyl-1*H*-benzo[*d*]imidazole **5a** was treated with 1-diazonaphthalen-2(1*H*)-one **2a**, the reaction did not proceed. This outcome infers that under the standard reaction conditions, Csp^3^-H activation is not possible. In an attempt to trap the reaction intermediate, 2-aryl benzimidazole **1a** was treated with [RhCp*Cl_2_]_2_ and AgOAc in DCE at 80 C for 1h. Rhodacycle **B** was formed and detected by high-resolution mass spectrometry (HRMS) (*m*/*z* = 431.0995). This outcome indicated that rhodacycle **B** might have formed at the beginning of the catalytic cycle. When five-membered rhodacycle **B** was allowed to react with 1-diazonaphthalen-2(1*H*)-one **2a**, six-membered rhodacycle **G** was formed and detected by HRMS (*m*/*z* = 573.1413) (Scheme 2a). The reaction of **1a** with D_2_O under the optimized reaction conditions furnished **1a′–d**_**2**_ with 91% deuterium exchange at the two *ortho* positions. This H/D exchange experiment disclosed that the C–H activation step is reversible (Scheme 2b). Finally, we performed a kinetic isotopic effect (KIE) study. Thus, the competitive reaction between a mixture of **1a** and **1a’–d**_**5**_ and **2a** gave a *K*_H_/*K*_D_ value of 2.50. Whereas, two parallel reactions of **1a** and **1a’–d**_**5**_ with **2a** gave a *K*_H_/*K*_D_ value of 2.44. These significant primary KIE values suggest that C–H bond cleavage possibly occurs in the rate-determining step (Scheme 2c).

**Scheme 3:**
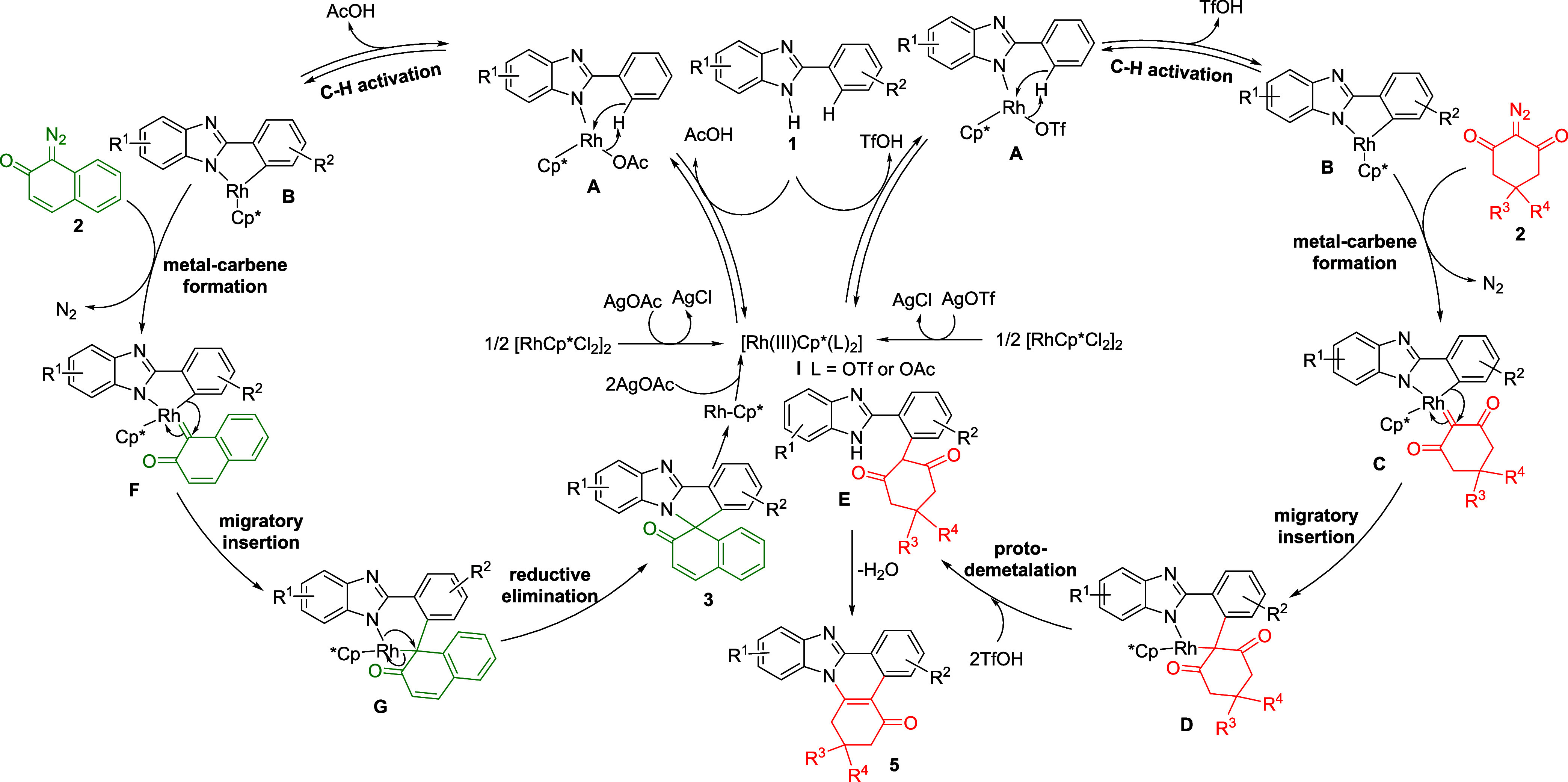
Plausible Mechanism for the Exclusive Formation of [4 + 1] Adduct **3** and [4 + 2] Adduct **5**

Based on the controlled experiments and previous reports,^9^ a plausible mechanistic pathway for the formation of [4 + 1] adduct **3** and [4 + 2] adduct **5** is depicted in Scheme 3. Initially, an active Rh(III) complex is generated via ligand exchange between [RhCp*Cl_2_]_2_ and AgOTf. Next, a Rh atom of [RhCp*(OTf)_2_] coordinates with a *N* atom of 2-aryl benzimidazole **1** and the subsequent C–H activation delivers five-membered rhodacycle **B**.

Coordination of rhodacycle **B** with 2-diazocyclohexane-1,3-dione **4** and the explusion of the N_2_ provides Rh-carbenoid intermediate **C**. Migratory insertion of **4** into the C–Rh bond furnishes six-membered rhodacycle **D**. Protodemetalation of the intermediate **D** forms intermediate **E**, and the active Rh (III) species is regenerated by the action of triflic acid for the next cycle. Finally, the intramolecular nucleophilic attack of NH of benzimidazole on the carbonyl group followed by expulsion of H_2_O gives [4 + 2] adduct **4** only. For the left catalytic cycle, **1** is reacted with active Rh(III) species to form five-membered rhodacycle **B**. In the next step, intermediate **F** forms via metal–carbene formation and extrusion of N_2_. Migratory insertion of **2** into the C–Rh bond affords six-membered rhodacycle **G**. In the next step, rhodacycle **G** undergoes reductive elimination to give [4 + 1] cyclized product **3** only accompanied by the release of Rh(I) species, which subsequently oxidizes by AgOAc to regenerate active Rh(III) species for the next catalytic cycle.

## Conclusions

In conclusion, we have demonstrated for the first time Rh(III)-catalyzed substrate-controlled synthesis of benzimidazole-fused quinolines and spirocyclic benzimidazole-fused isoindoles from 2-arylbenzimidazoles and α-diazo carbonyl compounds. Utilization of 2-diazocyclohexane-1,3-diones in the reaction gives substituted pentacyclic *N*-heterocycles only via [4 + 2] cyclization, whereas the use of diazonaphthalen-1(2*H*)-ones produce valuable spirocyclic benzimidazole-fused isoindoles by acting as a C1 synthon in an exclusive [4 + 1] annulations. A detailed mechanistic investigation showed that the reaction proceeds via benzimidazole-directed *ortho* C–H activation, followed by metal–carbene insertion. Intramolecular cyclization or reductive elimination finally provides respective [4 + 1] and [4 + 2] products.

## Data Availability

The data underlying this study are available in the published article and its Supporting Information.

## Supporting Information Available

Experimental procedures; characterization data and spectral data; and X-ray data for compounds **3h** and **5q** (PDF)
