# Supplementary material for: Substrate-Controlled Divergent Synthesis of Benzimidazole-Fused Quinolines and Spirocyclic Benzimidazole-Fused Isoindoles
Source: J Org Chem. 2024 May 9;89(11):7513–20. doi: 10.1021/acs.joc.4c00164 (PMC11165576; doi:10.1021/acs.joc.4c00164)
Supplement: Supplementary file 1 — jo4c00164_si_001.pdf [file jo4c00164_si_001.pdf]

Supporting Information

Substrate-controlled Divergent Synthesis of Benzimidazole-Fused Quinolines and Spirocyclic Benzimidazole-Fused Isoindoles

Ying-Ti Huang,[1] Wan-Wen Huang,[1] Yi-Ting Huang,[1] Hong-Ren Chen,[1] Indrajeet J. Barve[3] and Chung-Ming Sun[1,2]*

[1] *Department of Applied Chemistry, National Yang Ming Chiao Tung University,1001 Ta-Hsueh Road, Hsinchu 300-10, Taiwan, ROC*

[2] *Department of Medicinal and Applied Chemistry, Kaohsiung Medical University, 100, Shih-Chuan 1st Road, Kaohsiung 807-08, Taiwan, ROC*

[3] *Department of Chemistry, MES Abasaheb Garware College, Pune 411004, Maharashtra, India*

E-mail: cmsun@nycu.edu.tw

Table of Contents

General Methods	S2
Table S1	S3
Scheme S1	S4
A Representative procedure for the synthesis of **1a**, **2a**, **4a**, **3d** and **5a**	S5-S9
Mechanistic Study	S9-S13
Characterization Data of **3a**-**3v** and **5a**-**5y**	S14-S30
Spectral Data of **3a**-**3u**vand **5a**-**5y**	S31-S174
X-ray crystallographic data of **3h**	S175-S190
X-ray crystallographic data of **5q**	S191-S204

General Methods

^{1}H NMR (400 MHz and 600 Hz) and ^{13}C NMR (101 MHz and 151 Hz) spectra were recorded on 400 and 600 MHz automated spectrometers. Chemical shifts are reported in parts per million (ppm) on the δ scale from an internal standard (TMS). Analytical thin-layer chromatography (TLC) was performed using 0.25 mm silica gel-coated Kiselgel 60 F_{254} plates. Flash chromatography was performed using the indicated solvent and silica gel 60 (Merck, 230-400 mesh). High-resolution mass spectra (HRMS) were recorded in ESI mode using TOF mass spectrometer. Single crystal X-ray diffraction data was collected in Rigaku XtaLAB Synergy-DW diffractometer. All materials were purchased from commercial sources and used without further purification.

Table S1. Optimization of reaction conditions[a]

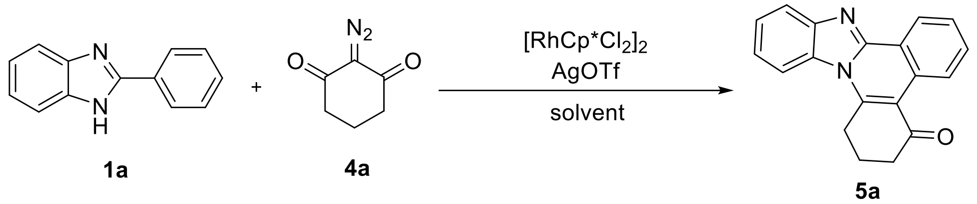

Entry	Catalyst	Additive	Solvent	T(°C)	Time (h)	Yield(%)[b]
1	$[RhCp^*Cl_2]_2$	$AgSbF_6$	THF	100	6	54
2	$[RuCl_2(p\text{-cymene})]_2$	$AgSbF_6$	THF	100	6	47
3	$[IrCp^*Cl_2]_2$	$AgSbF_6$	THF	100	6	5
4[c]	$[RhCp^*Cl_2]_2$	$AgSbF_6$	THF	100	6	37
5	$[RhCp^*Cl_2]_2$	AgOAc	THF	100	6	80
6	$[RhCp^*Cl_2]_2$	KPF_6	THF	100	6	33
7	$[RhCp^*Cl_2]_2$	$AgNTf_2$	THF	100	6	76
8	$[RhCp^*Cl_2]_2$	CsOAc	THF	100	6	81
9	$[RhCp^*Cl_2]_2$	CuOAc	THF	100	6	78
10	$[RhCp^*Cl_2]_2$	AgOTf	THF	100	6	94
11	$[RhCp^*Cl_2]_2$	AgOTf	ACN	100	6	29
12	$[RhCp^*Cl_2]_2$	AgOTf	toluene	100	6	79
13	$[RhCp^*Cl_2]_2$	AgOTf	DCE	100	6	94
14	$[RhCp^*Cl_2]_2$	AgOTf	EtOH	100	6	65
15	$[RhCp^*Cl_2]_2$	AgOTf	DCM	50	6	81
16	$[RhCp^*Cl_2]_2$	AgOTf	DMF	100	6	98

[a]Reaction conditions: **1a** (1 equiv), **4a** (1.2 equiv), $[RhCp^*Cl_2]_2$ (5 mol%), AgOTf (20 mol%), solvent (3mL), sealed tube, 100 °C, 6 h. [b]Isolated yield. [c]reflux.

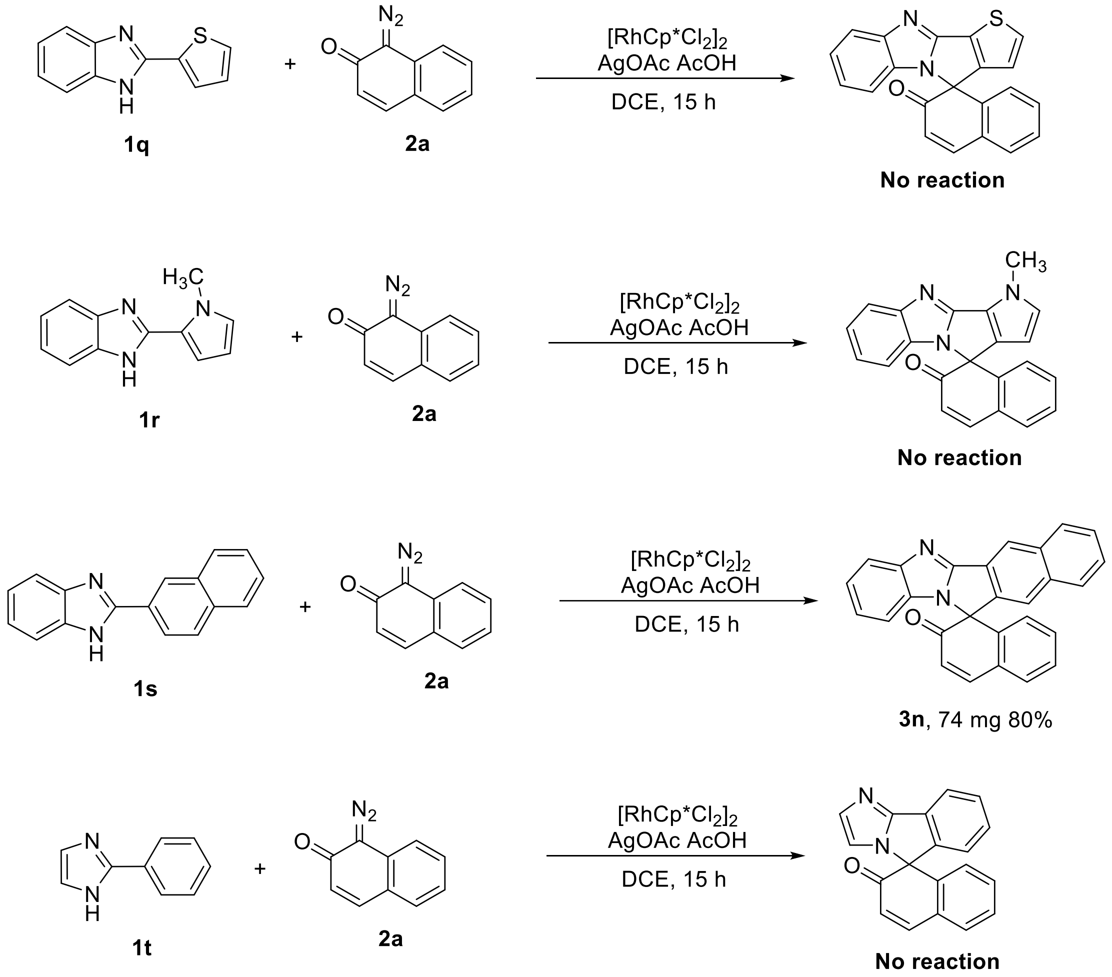

Scheme S1. Reactions of **2a** with **1q, 1r, 1s** and **1t**

A representative procedure for the synthesis of 1a

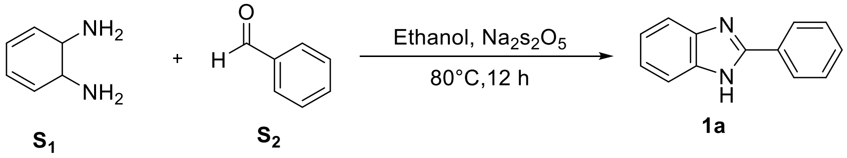

To an oven-dried 50 mL round bottom flask equipped with a magnetic stirring bar was added **S1** (100 mg, 0.909 mmol), and $Na_2S_2O_5$ (863 mg, 4.54 mmol) in Ethanol (5 mL). To the above reaction mixture was added **S2** (105 mg, 0.999 mmol), and the reaction was stirred at 80 °C (oil bath) for 12 h. After completion of the reaction, the reaction mixture was concentrated under vacuum. The crude product was purified by flash column chromatography on silica gel (30% ethyl acetate/hexane) to obtain product **1a** (140 mg, 79%).

^{1}H NMR (400 MHz, DMSO-d_6) δ 8.19 – 8.14 (m, 2H), 7.62 – 7.51 (m, 4H), 7.51 – 7.44 (m, 1H), 7.22 – 7.16 (m, 2H).

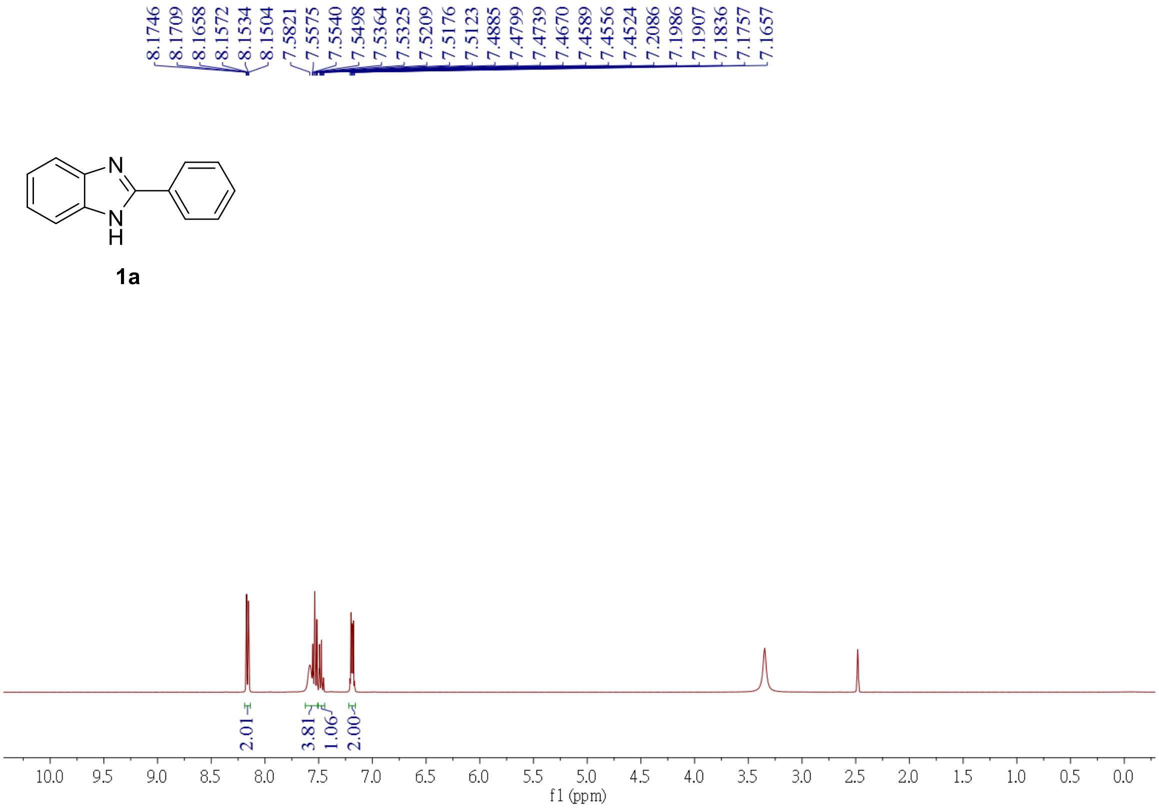

^{1}H NMR Spectrum (400 MHz) of **1a** in DMSO-d_6

A representative procedure for the synthesis of 2a

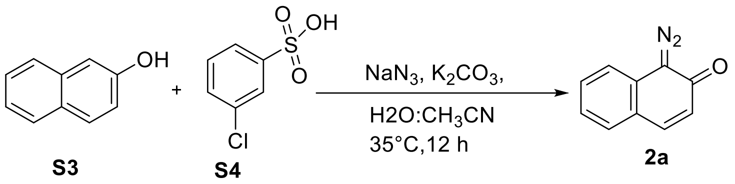

A solution of sodium azide (1.2 g, 19.80 mmol), K_2CO_3 (3.5g, 25.74 mmol) and 3-chlorobenzenesulfonic acid **S_4** (2.2 g, 11.88 mmol) in H_2O (20 ml) was stirred for 15 minutes at room temperature to obtained clear solution. To the above reaction mixture was added a solution of naphthalen-2-ol **S3** (2 g, 9.90 mmol) in CH_3CN (5 ml) and the resulting reaction mixture was stirred at 35°C for 12 h. After completion of the reaction, the reaction mixture was quenched by ice water (10 mL) and extracted with ethyl acetate (15 mL x 3). The combined organic layers were dried over Na_2SO_4 and concentrated under vacuum. The crude product was purified by flash chromatography on silica gel (30% ethyl acetate/hexane) to obtain the desired product **2a** (1.5 g, 90%) ^{1}H NMR (400 MHz, $CDCl_3$) δ 7.44 (d, J = 9.8 Hz, 1H), 7.38 (d, J = 7.8 Hz, 1H), 7.32 (td, J = 8.3, 7.8, 1.2 Hz, 1H), 7.10 – 7.06 (m, 2H), 6.47 (d, J = 9.8 Hz, 1H).

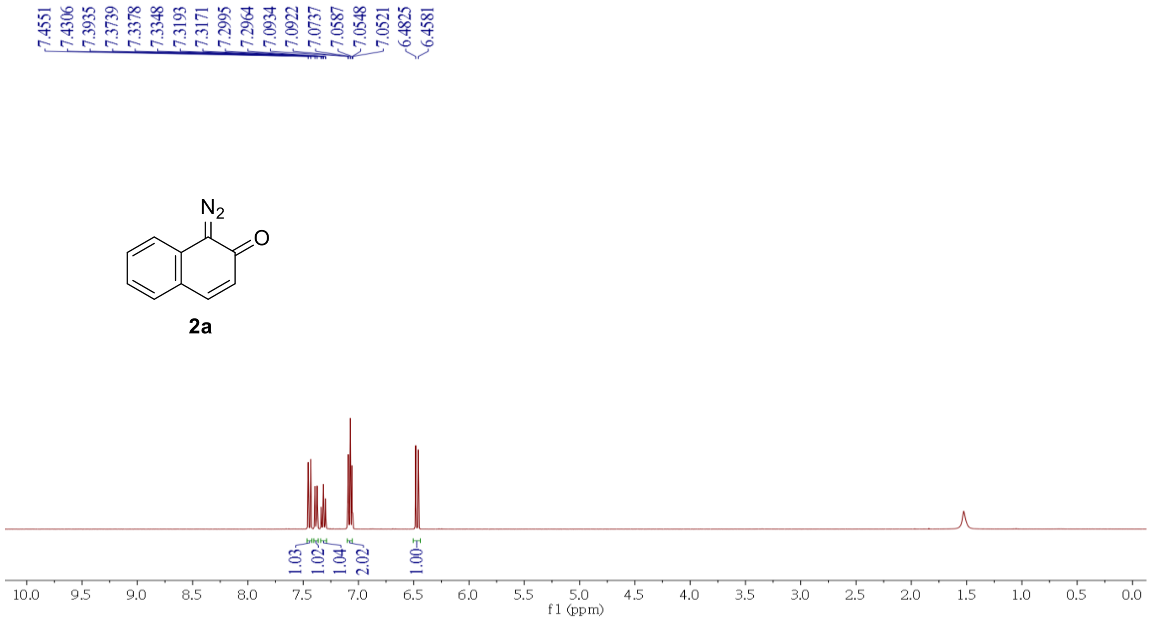

^{1}H NMR Spectrum (400 MHz) of **2a** in $CDCl_3$

A representative procedure for the synthesis of 4a

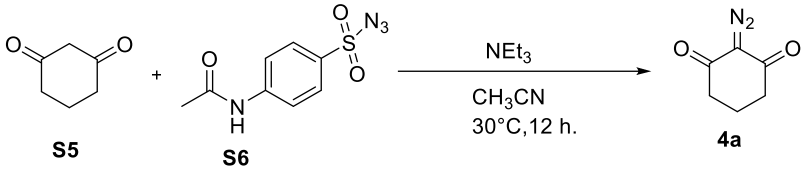

S5 S6 4a

A 50 mL round-bottomed flask equipped with a stirring bar was charged with cyclohexane-1,3-dione **S5** (1 g, 8.91 mmol) in CH_3CN (15 mL). To the above solution was added successively 4-acetamidobenzenesulfonyl azide **S6** (2.3 g, 9.81 mmol) and triethyl amine (3.7 mL, 26.78 mmol) at 0°C and stirred at 30 °C for 12 h. After completion of the reaction, the reaction mixture was diluted with H_2O (15 mL) and extracted with ethyl acetate (15 ml x 3). The combined organic layers were dried over Na_2SO_4 and concentrated under vacuum. The crude product was purified by flash chromatography on silica gel (20% ethyl acetate/hexane) to obtain desired product **4a** (1.1 g, 89%). ^{1}H NMR (400 MHz, $CDCl_3$): δ = 2.58 (t, *J* = 6.4 Hz, 4 H), 2.03 (p, *J*= 6.4 Hz, 2 H).

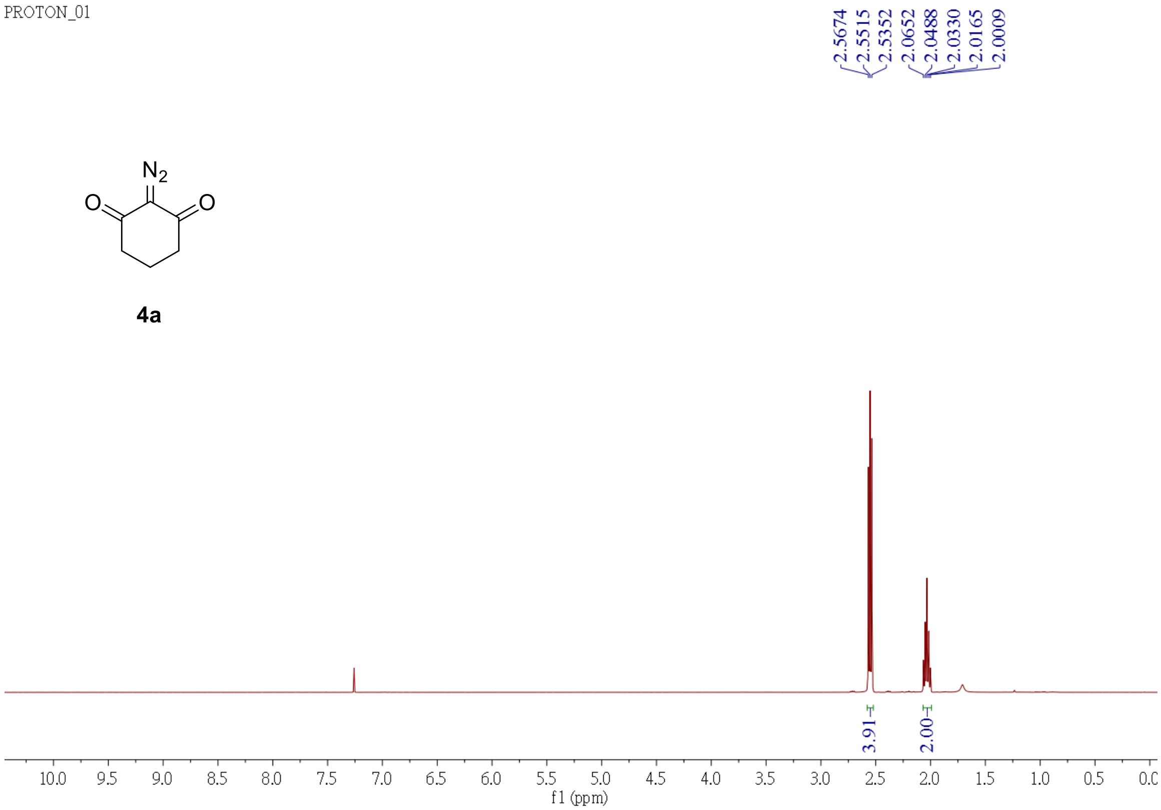

^{1}H NMR Spectrum (400 MHz) of **4a** in $CDCl_3$

A representative procedure for the synthesis of 3d

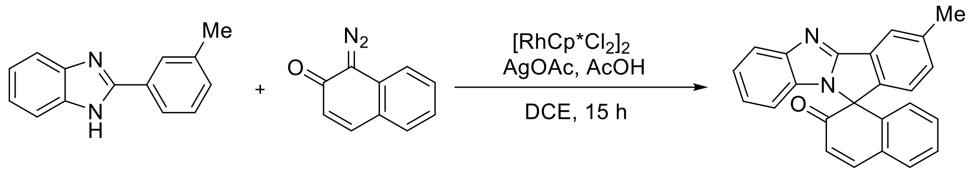

1d **2a** **3d**

To an oven-dried 25 mL round bottom flask equipped with a magnetic stirring bar was added **1d** (50 mg, 0.24 mmol), $[RhCp^*Cl_2]_2$ (3.71 mg, 2.5 mol%), AgOAc (159.62 mg, 0.96 mmol) and DCE (5 mL). To the above reaction mixture was added **2a** (44.95 mg, 0.26 mmol) and AcOH (29.33 mg, 0.48 mmol), and the reaction was stirred at 80 °C (oil bath) for 15 h. After completion of the reaction, the reaction mixture was filtered through a Celite bed and the filtrate was concentrated. The crude product was purified by flash column chromatography on silica gel (100% DCE) to obtain product **3d** (67 mg, 81%).

synthesis of 3d on 1 mmol scale

To an oven-dried 25 mL round bottom flask equipped with a magnetic stirring bar was added **1d** (210 mg, 1.0 mmol), $[RhCp^*Cl_2]_2$ (15.6 mg, 2.5 mol%), AgOAc (673.21 mg, 4.03 mmol) and DCE (10 mL). To the above reaction mixture was added **2a** (171.6 mg, 1.0 mmol) and AcOH (121.1 mg, 2.01 mmol), and the reaction was stirred at 80 °C (oil bath) for 15 h. After completion of the reaction, the reaction mixture was filtered through a Celite bed and the filtrate was concentrated. The crude product was purified by flash column chromatography on silica gel (100% DCE) to obtain product **3d** (274 mg, 78%).

A representative procedure for the synthesis of 5a

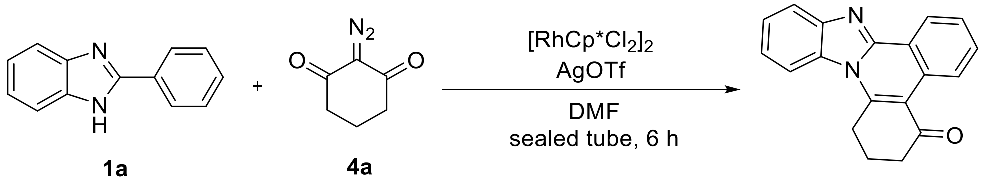

1a **4a** **5a**

To an oven-dried sealed tube equipped with a magnetic stirring bar was added **1a** (100 mg, 0.5 mmol), **4a** (89 mg, 0.6 mmol), $[RhCp^*Cl_2]_2$ (16 mg, 5 mol%), AgOTf (28 mg, 0.1 mmol) and

DMF (3 mL). The reaction mixture was heated at 100 °C (oil bath) for 6 h. After completion of the reaction, the solvent was removed under reduced pressure. The crude product was purified by flash column chromatography on silica gel (30% ethyl acetate/*n*-hexane) to obtain product **5a** (140 mg, 95%).

Preparation of single crystal of 3h and 5q

In a 5 mL sample vial, 5 mg of **3h** or **5q** was dissolved in ethyl acetate (2.5 mL) and 1 mL of *n*-hexane was added to it. The vial was kept at room temperature for 5 days. Slow evaporation of the solvent afforded crystals for single-crystal X-ray diffraction.

Mechanistic Study

a) Control experiment

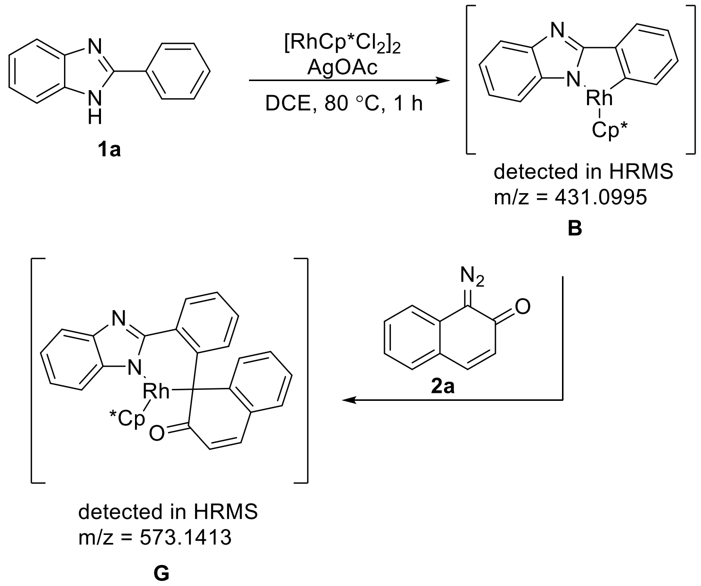

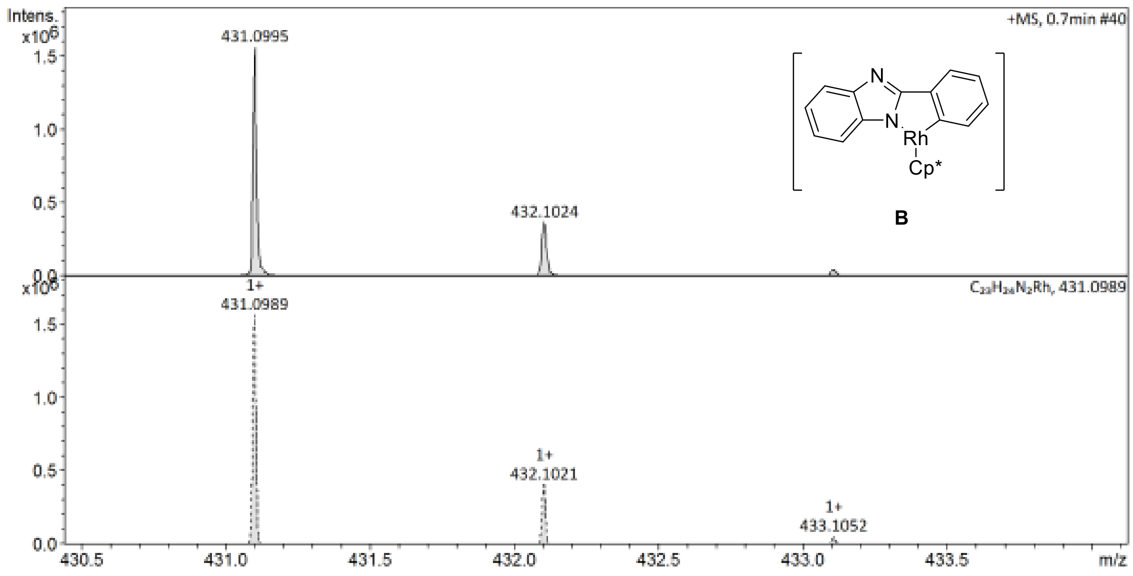

Display Report

Meas. m/z	#	Ion Formula	m/z	err [ppm]	mSigma	# Sigma	Score	rdb	e⁻ Conf	N-Rule	Adduct
431.0995	1	C23H24N2Rh	431.0989	1.4	15.2	1	100.00	13.5	even	ok	M+H

HRMS (ESI) of intermediate **B**

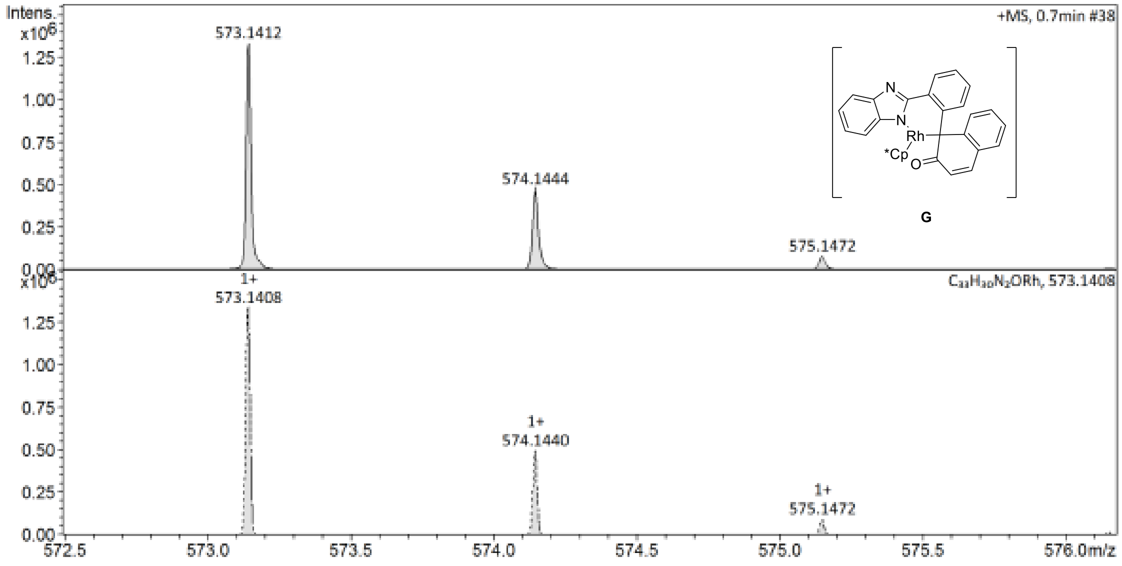

Display Report

Meas. m/z	#	Ion Formula	m/z	err [ppm]	mSigma	# Sigma	Score	rdb	e⁻ Conf	N-Rule	Adduct
573.1412	1	C33H30N2ORh	573.1408	-0.8	9.4	1	100.00	20.5	even	ok	M+H

HRMS (ESI) of intermediate **G**

b) Deuterium exchange

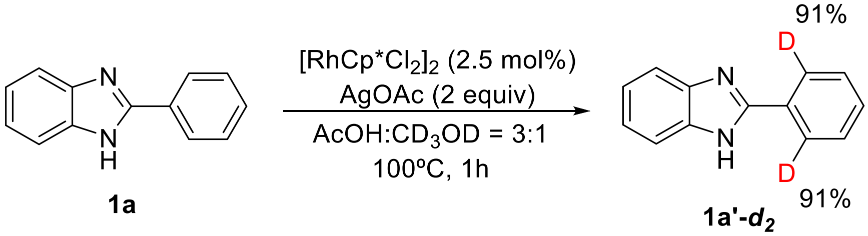

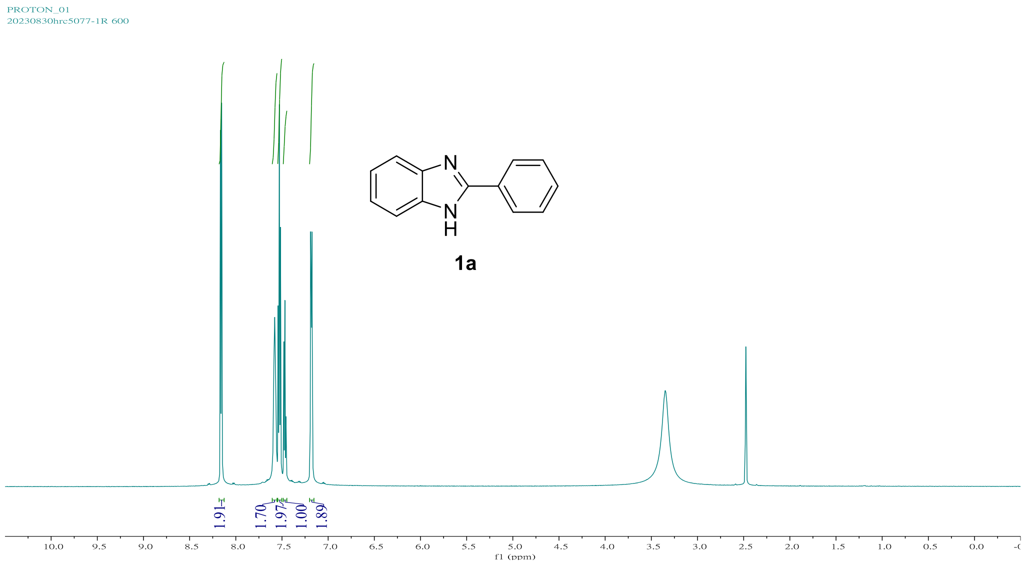

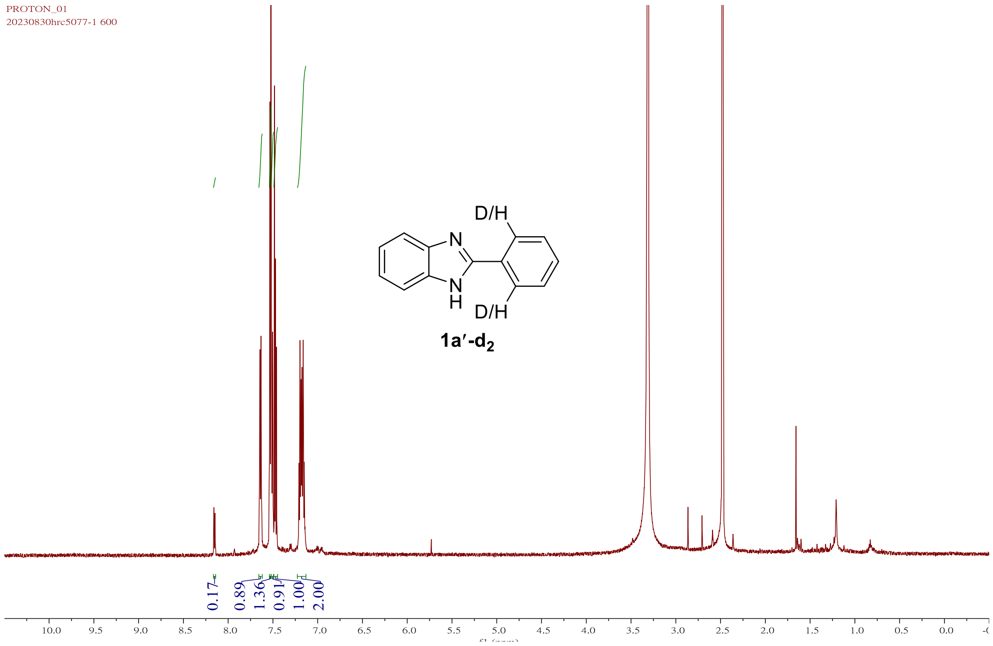

c) KIE study

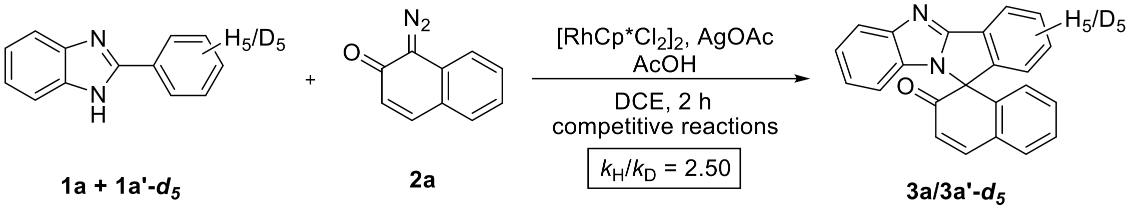

Competitive reactions: An oven-dried 25 mL round bottom flask was charged with **1a** (50 mg, 0.25 mmol) and **1a′-d5** (50 mg, 0.25 mmol), $[RhCp^*Cl_2]_2$ (3.9 mg, 2.5 mol%), AgOAc (171.85 mg, 1.02 mmol) and DCE (5 mL). To the above reaction mixture was added **2a** (48.1 mg, 0.28 mmol) and AcOH (31.0 mg, 0.51 mmol) and the reaction mixture was stirred at 80 °C (oil bath) for 2 h. After completion of the reaction, the reaction mixture was filtered through a Celite bed and the filtrate was concentrated. Purification was performed by flash column chromatography on silica gel (100% DCE) to afford the corresponding products. The KIE value was determined using ^{1}H NMR.

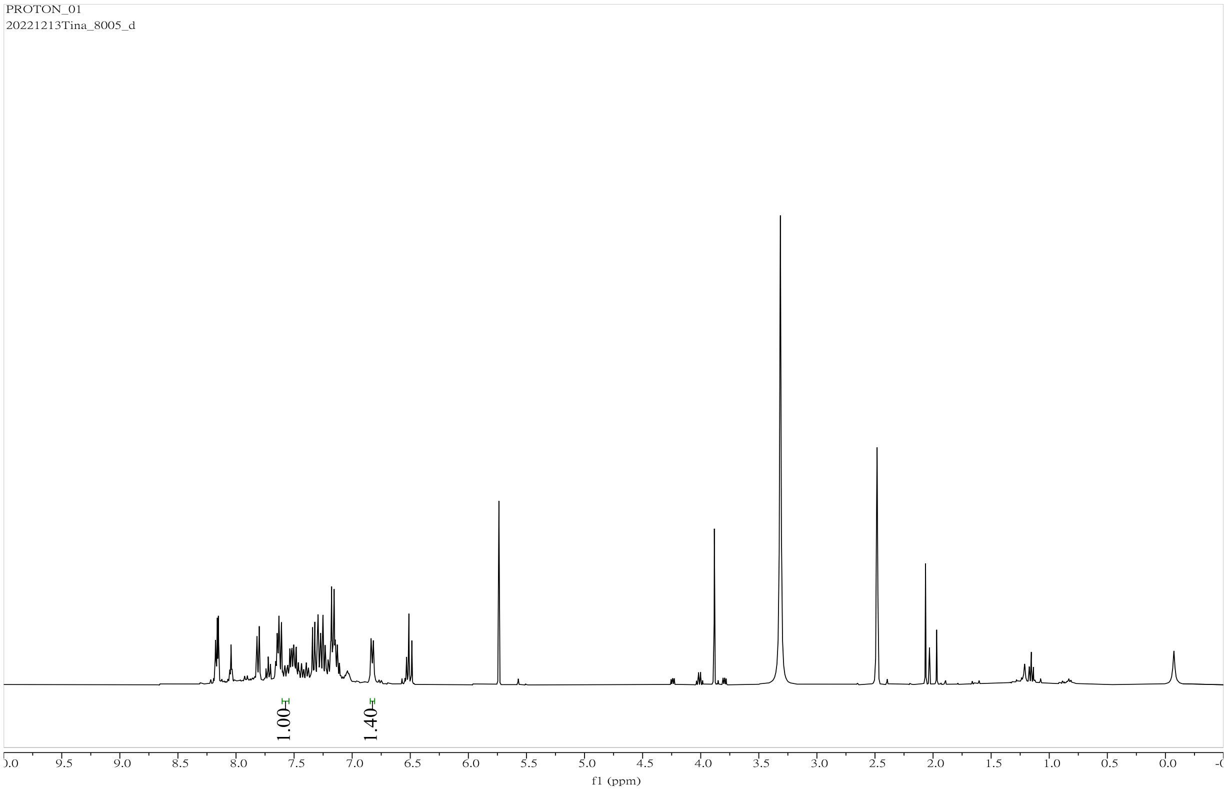

^{1}H NMR Spectrum (400 MHz) for competitive reactions in DMSO-d_6

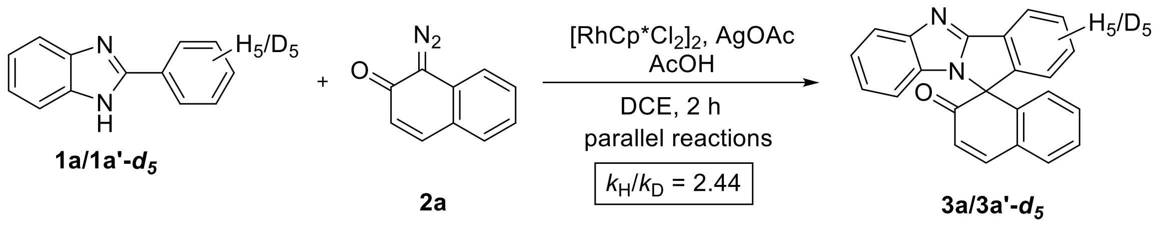

Parallel reactions: Two separate oven-dried 25 mL round bottom flasks were charged with **1a** (50 mg, 0.25 mmol) or **1a′-d₅** (50 mg, 0.25 mmol), $[RhCp^*Cl_2]_2$ (3.9 mg, 2.5 mol%), AgOAc (171.85 mg, 1.02 mmol) and DCE (5 mL). To the above reaction mixture was added **2a** (48.1 mg, 0.28 mmol) and AcOH (31.0 mg, 0.51 mmol) and the reaction mixture was stirred at 80 °C (oil bath) for 2 h. Each of the reaction mixture was filtered through a Celite bed separately and the respective filtrates were concentrated. Purification was performed by flash column chromatography on silica gel (100% DCE) to afford the corresponding products. The KIE value was determined using ^{1}H NMR.

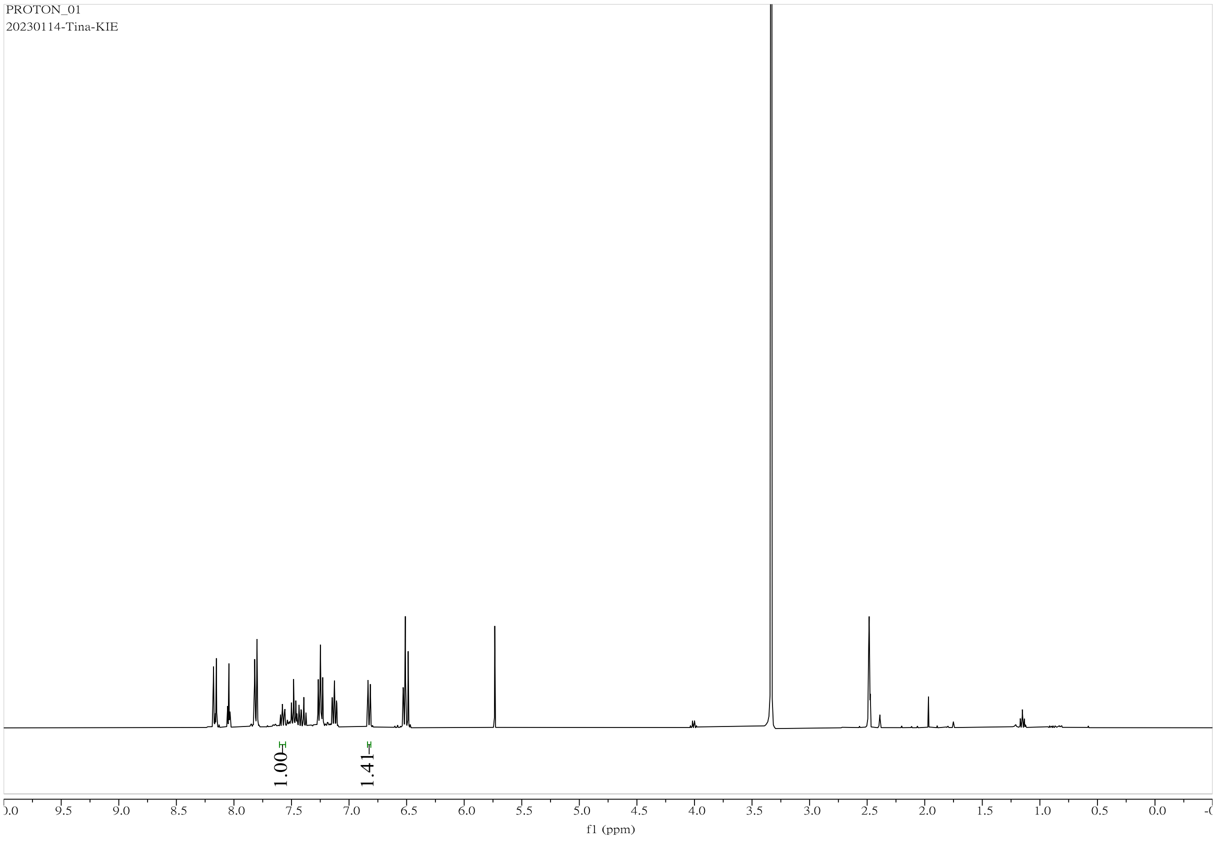

^{1}H NMR Spectrum (400 MHz) for parallel reactions in DMSO-d_6

Characterization Data for 3a-3v and 5a-5y

2'*H*-spiro [benzo [4, 5] imidazo [2, 1-*a*] isoindole-11, 1'-naphthalen]-2'-one (**3a**)

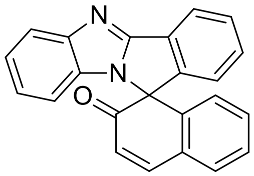

Flash chromatography for purification: 100% DCE; Off-white solid, yield: 39 mg, 45%; ^{1}H NMR (400 MHz, Acetone-d_6) δ 8.09 (d, *J* = 10.1 Hz, 1H), 8.04 (d, *J* = 7.7 Hz, 1H), 7.78 (dd, *J* = 12.8, 7.9 Hz, 2H), 7.56 (td, *J* = 7.3, 1.6 Hz, 1H), 7.50 – 7.38 (m, 3H), 7.25 (dtd, *J* = 11.0, 7.9, 1.3 Hz, 2H), 7.14 – 7.09 (m, 1H), 6.85 (d, *J* = 7.9 Hz, 1H), 6.59 (d, *J* = 7.8 Hz, 1H), 6.43 (d, *J* = 10.1 Hz, 1H); ^{13}C{1H} NMR (101 MHz, Acetone-d_6) δ 193.0, 158.7, 149.5, 147.8, 147.5, 137.7, 131.6, 131.2, 131.0, 130.7, 130.3, 129.9, 129.7, 127.6, 125.9, 124.7, 123.1, 122.7, 122.5, 122.3, 120.7, 110.3, 74.0; HRMS (ESI, *m/z*) calculated for $C_{23}H_{15}N_2O$ $(M + H)^+$ 335.1179, found 335.1178.

2-methyl-2'*H*-spiro [benzo [4, 5] imidazo [2, 1-*a*] isoindole-11, 1'-naphthalen]-2'-one (**3b**)

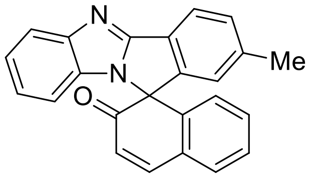

Flash chromatography for purification: 100% DCE; Light brown solid, yield: 41 mg, 49%; ^{1}H NMR (400 MHz, Acetonitrile-d_3) δ 8.02 (d, *J* = 10.1 Hz, 1H), 7.95 (d, *J* = 7.8 Hz, 1H), 7.84 (d, *J* = 8.1 Hz, 1H), 7.77 – 7.71 (m, 1H), 7.50 (td, *J* = 7.6, 1.1 Hz, 1H), 7.40 (d, *J* = 8.3 Hz, 1H), 7.32 – 7.22 (m, 3H), 7.19 – 7.12 (m, 1H), 6.85 (d, *J* = 8.0 Hz, 1H), 6.64 (d, *J* = 7.8 Hz, 1H), 6.44 (d, *J* = 10.1 Hz, 1H), 2.35 (s, 3H). HRMS (ESI, *m/z*) calculated for $C_{24}H_{17}N_2O$ $(M + H)^+$ 349.1341, found 349.1330.

2-(trifluoromethyl)-2'*H*-spiro [benzo [4, 5] imidazo [2, 1-*a*] isoindole-11, 1'-naphthalen]-2'-one (**3c**)

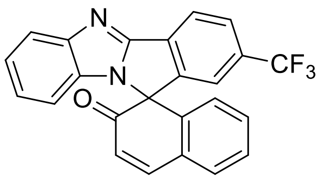

Flash chromatography for purification: 100% DCE; Off-white solid, yield: 27 mg, 35%; ^{1}H NMR (600 MHz, DMSO-d_6) δ 8.28 (d, *J* = 7.8 Hz, 1H), 8.21 (d, *J* = 10.2 Hz, 1H), 7.99 (d, *J* = 7.8 Hz, 1H), 7.88 – 7.81 (m, 2H), 7.63 (s, 1H), 7.52 (td, J = 7.6, 0.8 Hz, 1H), 7.31 – 7.25 (m, 2H), 7.18 (ddd, *J* = 8.2, 7.2, 1.0 Hz, 1H), 6.88 (d, *J* = 8.2 Hz, 1H), 6.59 (d, *J* = 7.8 Hz, 1H), 6.53 (d, *J* = 10.1 Hz, 1H); ^{13}C{1H} NMR (101 MHz, DMSO-d_6) δ 193.0, 156.9, 149.3, 149.1, 148.3, 136.2, 132.0, 131.9, 131.4, 131.3, 131.0, 130.7, 130.6, 130.6, 130.3, 128.1, 128.1, 126.5, 125.3, 124.4, 124.4, 124.1, 123.3, 122.6, 121.3, 119.5 (q, *J* = 3.8 Hz), 111.0, 73.5; ^{19}F NMR (376 MHz, DMSO-d_6) δ -60.83; HRMS (ESI, *m/z*) calculated for $C_{24}H_{14}F_3N_2O$ $(M + H)^+$ 403.1058, found 403.1054.

3-methyl-2'*H*-spiro [benzo [4, 5] imidazo [2, 1-*a*] isoindole-11, 1'-naphthalen]-2'-one (3d)

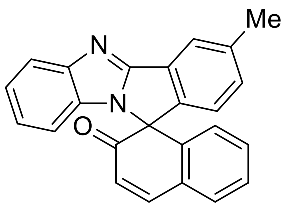

Flash chromatography for purification: 100% DCE; Light brown solid, yield: 68 mg, 81%; ^{1}H NMR (400 MHz, DMSO-d_6) δ 8.13 (d, *J* = 10.0 Hz, 1H), 7.86 (s, 1H), 7.82 (d, *J* = 8.0 Hz, 1H), 7.78 (d, *J* = 6.4 Hz, 1H), 7.44 (td, *J* = 7.6, 1.2 Hz, 1H), 7.26 – 7.16 (m, 4H), 7.12 – 7.06 (m, 1H), 6.80 (d, *J* = 7.9 Hz, 1H), 6.48 (d, *J* = 10.0 Hz, 2H), 2.36 (s, 3H); ^{13}C{1H} NMR (101 MHz, DMSO-d_6) δ 193.8, 158.7, 149.2, 148.5, 145.1, 140.4, 137.6, 131.7, 131.7, 131.5, 131.4, 130.5, 130.0, 127.3, 125.9, 124.7, 123.6, 123.3, 122.7, 122.5, 120.9, 110.7, 73.8, 21.3; HRMS (ESI, *m/z*) calculated for $C_{24}H_{14}F_3N_2O$ $(M + H)^+$ 403.1058, found 403.1054.

3-(trifluoromethyl)-2'*H*-spiro [benzo [4, 5] imidazo [2, 1-*a*] isoindole-11, 1'-naphthalen]-2'-one (3e)

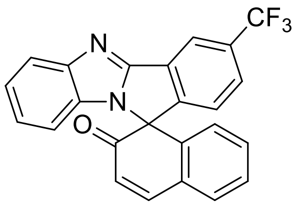

Flash chromatography for purification: 100% DCE; Off-white solid, yield: 60 mg, 78%;^{1}H NMR (600 MHz, DMSO-d_6) δ 8.40 (s, 1H), 8.23 (d, *J* = 10.1 Hz, 1H), 7.89 – 7.81 (m, 2H), 7.63 (d, *J* = 8.1 Hz, 1H), 7.54 (t, *J* = 7.5 Hz, 1H), 7.30 (q, *J* = 7.8 Hz, 2H), 7.20 (t, *J* = 7.6 Hz, 1H), 6.90 (d, *J* = 8.0 Hz, 1H), 6.61 (d, *J* = 7.8 Hz, 1H), 6.55 (d, *J* = 10.1 Hz, 1H); ^{13}C{1H} NMR (101 MHz, DMSO-d_6) δ 193.0, 157.2, 151.2, 149.2, 149.2, 136.4, 132.1, 131.9, 131.5, 131.4, 131.2, 130.7, 130.6, 128.6, 128.2, 128.1, 126.4, 125.5, 124.6, 124.4, 124.2, 123.3, 122.8, 121.3, 120.0, 120.0, 111.0, 73.9; ^{19}F NMR (376 MHz, DMSO-d_6) δ -61.14; HRMS (ESI, *m/z*) calculated for $C_{24}H_{14}F_3N_2O$ $(M + H)^+$ 403.1058, found 403.1051.

4-methyl-2'*H*-spiro [benzo [4, 5] imidazo [2, 1-*a*] isoindole-11, 1'-naphthalen]-2'-one (3f)

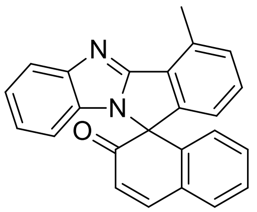

Flash chromatography for purification: 100% DCE; Brown solid, yield: 59 mg, 70%; ^{1}H NMR (400 MHz, $CDCl_3$) δ 8.01 (d, *J* = 9.4 Hz, 1H), 7.78 (d, *J* = 10.4 Hz, 1H), 7.55 (d, *J* = 8.4 Hz, 1H), 7.40 (t, *J* = 8.5 Hz, 1H), 7.30 (d, *J* = 9.8 Hz, 2H), 7.25 – 7.11 (m, 4H), 6.81 (d, *J* = 8.4 Hz, 1H), 6.64 (d, *J* = 10.8 Hz, 1H), 6.43 (d, *J* = 10.1 Hz, 1H), 2.93 (s, 3H); ^{13}C{1H} NMR (101 MHz, Chloroform-*d*) δ 193.5, 159.6, 149.4, 147.2, 146.7, 138.0, 136.3, 131.3, 131.2, 131.0, 130.5, 130.1, 129.8, 129.5, 126.4, 126.0, 125.1, 123.4, 122.5, 121.0, 119.6, 110.2, 73.9, 19.2; HRMS (ESI, *m/z*) calculated for $C_{24}H_{17}N_2O$ $(M + H)^+$ 349.1341, found 349.1331.

4-ethyl-2'*H*-spiro [benzo [4, 5] imidazo [2, 1-*a*] isoindole-11,1'-naphthalen]-2'-one (3g)

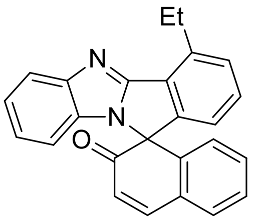

Flash chromatography for purification: 100% DCE; Light brown solid, yield: 65 mg, 80%; ^{1}H NMR (400 MHz, Chloroform-*d*) δ 8.05 (d, *J* = 8.2 Hz, 1H), 7.77 (d, *J* = 10.0 Hz, 1H), 7.54 (d, *J* = 7.6 Hz, 1H), 7.39 (td, *J* = 7.5, 1.2 Hz, 1H), 7.32 (dd, *J* = 10.5, 7.5 Hz, 2H), 7.28 – 7.23 (m, 1H), 7.20 – 7.13 (m, 3H), 6.80 (d, *J* = 8.0 Hz, 1H), 6.62 (d, *J* = 7.8 Hz, 1H), 6.40 (d, *J* = 10.0 Hz, 1H), 3.42 (hept, *J* = 7.3 Hz, 2H), 1.45 (t, *J* = 7.6 Hz, 3H); ^{13}C{1H} NMR (101 MHz, Chloroform-*d*) δ 192.9, 158.5, 147.3, 146.7, 143.0, 137.5, 131.4, 130.6, 130.4, 130.1, 129.7, 129.4, 126.4, 125.1, 124.7, 123.8, 123.1, 120.7, 119.7, 110.3, 26.1, 14.6; HRMS (ESI, *m/z*) calculated for $C_{25}H_{19}N_2O$ $(M + H)^+$ 363.1497, found 363.1490.

4-phenyl-2'*H*-spiro [benzo [4, 5] imidazo [2, 1-*a*] isoindole-11, 1'-naphthalen]-2'-one (3h)

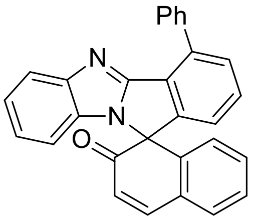

Flash chromatography for purification: 100% DCE; White solid; yield: 57 mg, 75%; ^{1}H NMR (400 MHz, DMSO-d_6) δ 8.17 (d, *J* = 10.1 Hz, 1H), 7.82 (dd, *J* = 12.5, 7.3 Hz, 3H), 7.70 (d, *J* = 8.1 Hz, 1H), 7.58 – 7.43 (m, 6H), 7.35 (d, *J* = 7.4 Hz, 1H), 7.26 (t, *J* = 7.6 Hz, 1H), 7.18 (t, *J* = 7.6 Hz, 1H), 7.09 (t, *J* = 7.5 Hz, 1H), 6.76 (d, *J* = 7.9 Hz, 1H), 6.61 (d, *J* = 7.7 Hz, 1H), 6.51 (d, *J* = 10.0 Hz, 1H).; ^{13}C{1H} NMR (101 MHz, Chloroform-*d*) δ 193.2, 158.3, 148.1, 146.7, 140.0, 137.9, 137.7, 131.5, 131.5, 130.8, 130.6, 130.2, 130.1, 129.7, 128.8, 128.6, 126.6, 125.2, 124.4, 123.6, 122.7, 121.5, 121.1, 110.0, 73.4.; HRMS (ESI, *m/z*) Calcd. $C_{29}H_{18}N_2O$ $[M+H]^+$: 411.1492; Found 411.1487.

4-(trifluoromethyl)-2'*H*-spiro [benzo [4, 5] imidazo [2, 1-*a*] isoindole-11, 1'-naphthalen]-2'-one (3i)

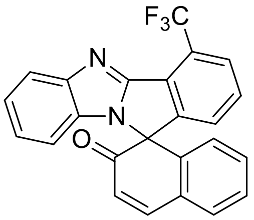

Flash chromatography for purification: 100% DCE; Brown solid, yield: 36 mg, 47%; ^{1}H NMR (400 MHz, Chloroform-*d*) δ 8.06 (d, *J* = 8.2 Hz, 1H), 7.81 (d, *J* = 10.1 Hz, 1H), 7.76 (d, *J* = 7.7 Hz, 1H), 7.58 (dd, *J* = 7.7, 1.3 Hz, 1H), 7.51 (d, *J* = 7.8 Hz, 1H), 7.44 (dd, *J* = 7.6, 1.2 Hz, 1H), 7.41 – 7.38 (m, 1H), 7.30 (ddd, *J* = 8.3, 7.2, 1.2 Hz, 1H), 7.22 (dd, *J* = 7.7, 1.3 Hz, 1H), 7.19 – 7.15 (m, 1H), 6.81 (d, *J* = 8.0 Hz, 1H), 6.63 (d, *J* = 8.3 Hz, 1H), 6.43 (d, *J* = 10.0 Hz, 1H); ^{13}C{1H} NMR (101 MHz, Chloroform-*d*) δ 192.5, 155.7, 149.4, 148.7, 146.8, 145.7, 136.9, 134.8, 131.5, 130.8, 130.7, 130.6, 130.1, 129.9, 129.8, 129.6, 127.4, 127.1, 126.8, 126.8, 126.7,

126.5, 126.3, 126.1, 126.1, 125.7, 125.7, 125.0, 124.4, 124.1, 122.9, 122.0, 121.6, 120.3, 110.2, 73.1; ^{19}F NMR (376 MHz, Chloroform-*d*) δ -61.91; HRMS (ESI, *m/z*) calculated for $C_{24}H_{14}F_3N_2O$ (M + H)$^+$ 403.1058, found 403.1054.

4-fluoro-2'*H*-spiro [benzo [4, 5] imidazo [2, 1-*a*] isoindole-11, 1'-naphthalen]-2'-one (3j)

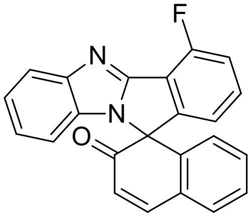

Flash chromatography for purification: 100% DCE; Off-white solid, yield: 56 mg, 67%; ^{1}H NMR (400 MHz, DMSO-d_6) δ 8.17 (d, J = 10.0 Hz, 1H), 7.86 (d, J = 8.1 Hz, 1H), 7.81 (d, J = 7.7 Hz, 1H), 7.51 – 7.40 (m, 3H), 7.29 – 7.20 (m, 3H), 7.17 – 7.11 (m, 1H), 6.84 (d, J = 8.0 Hz, 1H), 6.61 (d, J = 7.7 Hz, 1H), 6.51 (d, J = 10.1 Hz, 1H); ^{13}C{1H} NMR (101 MHz, DMSO-d_6) δ 193.1, 156.8 (d, J^1 = 256.54 Hz), 155.3, 155.3, 149.7 (d, J^3 = 4.04 Hz), 149.2, 148.9, 136.7, 133.5, 133.4, 131.8, 131.6, 131.0, 130.5, 130.3, 126.3, 124.4, 124.1, 122.9, 121.1, 119.3 (d, J^3 = 4.04 Hz), 117.7 (d, J^2 = 19.19 Hz), 115.1 (d, J^3 = 17.17 Hz), 110.7, 73.7; ^{19}F NMR (376 MHz, DMSO-d_6) δ -113.8; HRMS (ESI, *m/z*) calculated for $C_{23}H_{14}FN_2O$ (M + H)$^+$ 353.1090, found 353.1083.

4-chloro-2'*H*-spiro [benzo [4, 5] imidazo [2, 1-*a*] isoindole-11, 1'-naphthalen]-2'-one (3k)

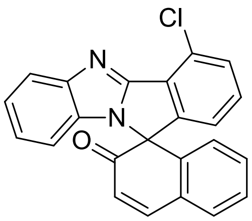

Flash chromatography for purification: 100% DCE; Gray solid, yield: 52 mg, 64%; ^{1}H NMR (400 MHz, DMSO-d_6) δ 8.18 (d, J = 9.7 Hz, 1H), 7.89 (d, J = 8.1 Hz, 1H), 7.82 (dd, J = 7.7, 1.3 Hz, 1H), 7.63 (dd, J = 8.0, 0.9 Hz, 1H), 7.49 (td, J = 7.6, 1.2 Hz, 1H), 7.42 (t, J = 7.9 Hz, 1H), 7.34 (dd, J = 7.7, 0.9 Hz, 1H), 7.26 (dtd, J = 10.0, 7.4, 1.3 Hz, 2H), 7.16 (ddd, J = 8.3, 7.3, 1.1 Hz, 1H), 6.84 (d, J = 7.9 Hz, 1H), 6.59 (d, J = 8.4 Hz, 1H), 6.51 (d, J = 10.0 Hz, 1H); ^{13}C{1H} NMR (101 MHz, DMSO-d_6) δ 193.2, 156.5, 149.3, 149.2, 149.0, 136.8, 132.5, 131.9, 131.7, 131.3, 131.0, 130.6, 130.4, 130.3, 130.2, 128.6, 126.3, 125.6, 125.3, 124.9, 124.5, 124.3, 123.0, 121.7, 121.3, 121.3, 110.7, 73.3; HRMS (ESI, *m/z*) calculated for $C_{23}H_{14}ClN_2O$ (M + H)$^+$ 369.0795, found 369.0801.

4-bromo-2'*H*-spiro [benzo [4, 5] imidazo [2, 1-*a*] isoindole-11, 1'-naphthalen]-2'-one (3l)

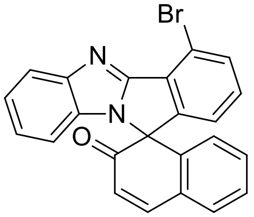

Flash chromatography for purification: 100% DCE; Light brown solid, yield: 43 mg, 57%; ^{1}H NMR (400 MHz, DMSO-d_6) δ 8.17 (d, *J* = 10.0 Hz, 1H), 7.90 (d, *J* = 8.2 Hz, 1H), 7.81 (d, *J* = 6.4 Hz, 1H), 7.78 (dd, *J* = 7.8, 1.0 Hz, 1H), 7.49 (td, *J* = 7.5, 1.2 Hz, 1H), 7.41 – 7.22 (m, 4H), 7.19 – 7.12 (m, 1H), 6.84 (d, *J* = 7.9 Hz, 1H), 6.58 (d, *J* = 7.8 Hz, 1H), 6.50 (d, *J* = 10.0 Hz, 1H); ^{13}C{1H} NMR (101 MHz, DMSO-d_6) δ 193.3, 157.1, 149.5, 149.1, 1489.0, 136.9, 134.5, 132.6, 132.0, 131.8, 131.1, 130.7, 130.5, 127.2, 126.4, 124.6, 124.4, 123.1, 122.2, 121.4, 116.7, 110.8, 73.2; HRMS (ESI, *m/z*) calculated for $C_{23}H_{14}BrN_2O$ $(M + H)^+$ 413.0290, found 413.0282.

3, 4-dimethoxy-2'*H*-spiro [benzo [4, 5] imidazo [2, 1-*a*] isoindole-11, 1'-naphthalen]-2'-one (3m)

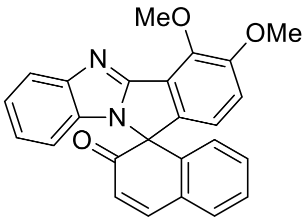

Flash chromatography for purification: 100% DCE; White solid, yield: 57 mg, 74%; ^{1}H NMR (400 MHz, DMSO-d_6) δ 8.14 (d, *J* = 10.1 Hz, 1H), 7.86 (d, *J* = 8.1 Hz, 1H), 7.80 (d, *J* = 7.5 Hz, 1H), 7.49 (t, *J* = 7.1 Hz, 1H), 7.31 – 7.21 (m, 2H), 7.17 – 7.10 (m, 1H), 7.07 (d, *J* = 8.5 Hz, 1H), 7.02 (d, *J* = 8.4 Hz, 1H), 6.79 (d, *J* = 8.0 Hz, 1H), 6.56 (d, *J* = 7.7 Hz, 1H), 6.48 (d, *J* = 10.0 Hz, 1H), 4.16 (s, 3H), 3.81 (s, 3H); ^{13}C{1H} NMR (101 MHz, Chloroform-*d*) δ 192.7, 156.3, 153.6, 146.6, 146.1, 139.6, 137.6, 131.4, 130.5, 130.4, 130.0, 129.7, 126.4, 125.0, 123.9, 123.2, 120.8, 117.3, 114.8, 110.3, 73.8, 62.1, 56.6; HRMS (ESI, *m/z*) calculated for $C_{25}H_{18}N_2O_3$ $(M+H)^+$ 395.1390, found 395.1394.

2'H-spiro[benzo[f]benzo[4,5]imidazo[2,1-a]isoindole-12,1'-naphthalen]-2'-one (3n)

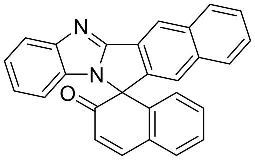

3n

Flash chromatography for purification: 100% DCE; Off-white solid, yield: 74 mg, 80%; ^{1}H NMR (400 MHz, DMSO-d_6) δ 8.67 (s, 1H), 8.25 (d, J = 10.1 Hz, 1H), 8.18 (d, *J* = 7.8 Hz, 1H), 8.02 – 7.93 (m, 2H), 7.90 – 7.84 (m, 2H), 7.61 (dt, *J* = 14.7, 6.7 Hz, 2H), 7.52 (t, *J* = 8.0 Hz, 1H), 7.29 (q, *J* = 8.8, 8.2 Hz, 2H), 7.17 (t, *J* = 7.6 Hz, 1H), 6.86 (d, *J* = 8.0 Hz, 1H), 6.62 (d, *J* = 7.8 Hz, 1H), 6.56 (d, *J* = 10.0 Hz, 1H); ^{13}C{1H} NMR (101 MHz, DMSO-d_6) δ 193.8, 158.1, 149.4, 148.9, 143.9, 138.1, 133.8, 133.6, 131.8, 131.7, 131.3, 130.6, 130.2, 129.5, 129.0, 128.3, 128.0, 126.4, 124.7, 124.5,

123.9, 123.0, 122.7, 122.1, 121.0, 110.9, 73.4, 74.0; HRMS (ESI, *m/z*) calculated for $C_{27}H_{16}N_2O$ $(M + H)^+$ 385.1335, found 385.1336.

7-chloro-3-methyl-2'*H*-spiro [benzo [4, 5] imidazo [2, 1-*a*] isoindole-11, 1'-naphthalen]-2'-one & 8-chloro-3-methyl-2'*H*-spiro [benzo [4, 5] imidazo [2, 1-*a*] isoindole-11, 1'-naphthalen]-2'-one (3o & 3o')

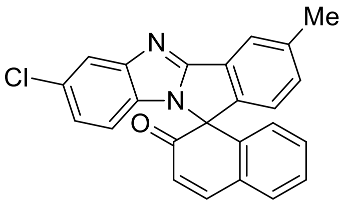

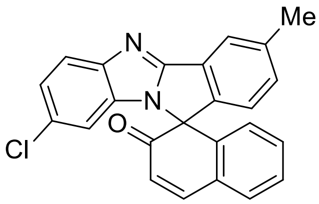

Flash chromatography for purification: 100% DCE; Off-white solid, yield: 63 mg, 80%; ^{1}H NMR (600 MHz, DMSO-d_6) δ 8.13 (d, *J* = 10.1 Hz, 1H), 7.85 (s, 1H), 7.79 (dd, *J* = 7.6, 5.0 Hz, 2H), 7.47 (t, *J* = 7.5 Hz, 1H), 7.26 – 7.22 (m, 3H), 7.12 (t, *J* = 7.6 Hz, 1H), 6.80 (d, *J* = 8.0 Hz, 1H), 6.50 – 6.45 (m, 2H), 2.39 (s, 3H).

^{13}C{1H} NMR (101 MHz, Chloroform-*d*) δ 1923.0, 192.9, 160.1, 149.5, 147.3, 146.8, 146.7, 144.6, 144.6, 140.4, 137.4, 137.3, 131.7, 131.5, 131.4, 131.4, 130.7, 130.6, 130.1, 129.8, 129.8, 129.7, 129.1, 128.4, 127.0, 126.9, 126.1, 125.1, 125.0, 123.9, 123.9, 123.8, 123.4, 122.1, 121.6, 120.6, 110.9, 110.4, 21.6; HRMS (ESI, *m/z*) calculated for $C_{24}H_{16}ClN_2O$ $(M + H)^+$ 383.0951, found 383.0950.

3, 7-dimethyl-2'*H*-spiro [benzo [4, 5] imidazo [2, 1-*a*] isoindole-11, 1'-naphthalen]-2'-one & 3, 8-dimethyl-2'*H*-spiro [benzo [4, 5] imidazo [2, 1-*a*] isoindole-11, 1'-naphthalen]-2'-one (3p & 3p')

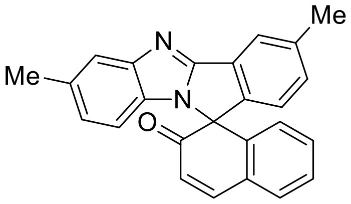

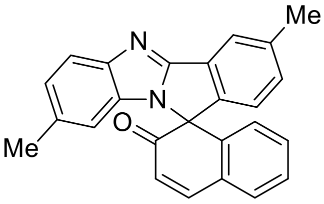

Flash chromatography for purification: 100% DCE; Light yellow solid, yield: 70 mg, 86%; ^{1}H NMR (400 MHz, Chloroform-*d*) δ 7.91 (d, *J* = 7.4 Hz, 1H), 7.79 – 7.68 (m, 2H), 7.55 – 7.49 (m, 1H), 7.37 (tdd, *J* = 7.6, 4.9, 1.2 Hz, 1H), 7.20 – 7.14 (m, 2H), 7.13 – 6.95 (m, 2H), 6.71 – 6.57 (m, 2H), 6.39 (dd, *J* = 10.0, 5.6 Hz, 1H), 2.46 (s, 1H), 2.40 (d, *J* = 0.9 Hz, 3H), 2.32 (s, 2H); HRMS (ESI, *m/z*) calculated for $C_{25}H_{19}N_2O$ $(M + H)^+$ 363.1497, found 363.1495.

3-methyl-7-nitro-2'*H*-spiro [benzo [4, 5] imidazo [2, 1-*a*] isoindole-11, 1'-naphthalen]-2'-one & 3-methyl-8-nitro-2'*H*-spiro [benzo [4, 5] imidazo [2, 1-*a*] isoindole-11, 1'-naphthalen]-2'-one (3q & 3q')

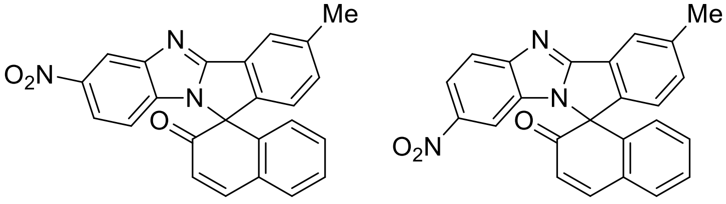

Flash chromatography for purification: 100% DCE; Off-white solid, yield: 56 mg, 72%; ^{1}H NMR (400 MHz, Chloroform-*d*) δ 8.81 (d, *J* = 2.1 Hz, 1H), 8.24 (dd, *J* = 9.0, 2.2 Hz, 1H), 8.09 (dd, *J* = 8.8, 2.1 Hz, 1H), 8.02 – 8.94 (m, 3H), 7.87 – 7.78 (m, 2H), 7.74 (d, *J* = 2.1 Hz, 1H), 7.59 (dd, *J* = 11.4, 7.6 Hz, 2H), 7.44 (q, *J* = 7.5 Hz, 2H), 7.22 (dd, *J* = 15.0, 7.4 Hz, 7H), 6.86 (d, *J* = 8.8 Hz, 1H), 6.59 (d, *J* = 7.7 Hz, 2H), 6.43 (dd, *J* = 10.0, 4.6 Hz, 2H), 2.44 (s, 6H); ^{13}C{1H} NMR (101 MHz, Chloroform-*d*) δ 193.1, 193.0, 160.7, 160.3, 150.1 – 149.8 (m), 147.8, 147.1, 146.9, 146.8, 137.5, 136.6, 136.6, 131.5, 131.4, 131.4, 130.7, 130.6, 130.2, 130.1, 129.8, 129.7, 129.5, 129.0, 128.2, 126.2, 125.5, 125.4, 125.1, 125.0, 123.3, 121.7, 120.8, 119.6, 110.8, 110.2, 74.1, 74.1, 19.2.; HRMS (ESI, *m/z*) calculated for $C_{24}H_{15}N_3O_3$ $[M+H]^+$ 394.1186, found 394.1188.

3, 7, 8-trimethyl-2'*H*-spiro [benzo [4, 5] imidazo [2, 1-a] isoindole-11, 1'-naphthalen]-2'-one (3r)

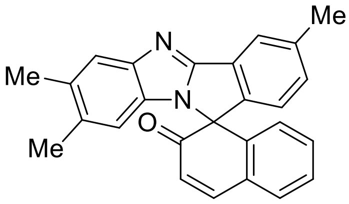

Flash chromatography for purification: 100% DCE; Light brown solid, yield: 68 mg, 85%; ^{1}H NMR (400 MHz, Chloroform-*d*) δ 7.92 (s, 1H), 7.76 (d, *J* = 10.0 Hz, 1H), 7.68 (s, 1H), 7.53 (dd, *J* = 7.6, 1.3 Hz, 1H), 7.38 (td, *J* = 7.6, 1.2 Hz, 1H), 7.20 – 7.14 (m, 2H), 7.08 (d, *J* = 8.0 Hz, 1H), 6.61 (d, *J* = 6.7 Hz, 1H), 6.57 (s, 1H), 6.40 (d, *J* = 10.0 Hz, 1H), 2.40 (s, 3H), 2.35 (s, 3H), 2.22 (s, 3H); ^{13}C{1H} NMR (101 MHz, Chloroform-*d*) δ 193.3, 157.8, 146.6, 144.4, 140.2, 137.9, 1323.0, 132.0, 131.4, 130.8, 130.5, 130.1, 129.5, 127.3, 126.2, 125.2, 123.7, 122.0, 120.8, 110.6, 21.6, 20.5, 20.5; HRMS (ESI, *m/z*) calculated for $C_{26}H_{21}N_2O$ $(M + H)^+$ 377.1654, found 377.1645.

6'-bromo-3-methyl-2'*H*-spiro [benzo [4, 5] imidazo [2, 1-*a*] isoindole-11, 1'-naphthalen]-2'-one (3s)

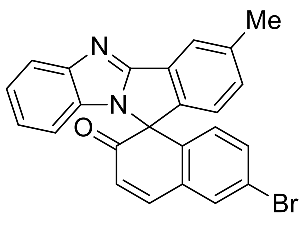

Flash chromatography for purification: 100% DCE; Brown solid, yield: 80 mg, 78%; ^{1}H NMR (400 MHz, DMSO-d_6) δ 8.13 – 8.04 (m, 2H), 7.83 (s, 1H) , 7.78 (d, *J* = 8.1 Hz, 1H), 7.41 (d, *J* =8.9 Hz, 1 H), 7.27-7.20 (m, 3H), 7.11 (t, *J* = 7.6 Hz, 1H), 6.86 (d, *J* = 7.8 Hz, 1H), 6.54 (d, *J* = 10.1 Hz, 1H), 6.44 (d, *J* = 8.2 Hz, 1H), 2.38 (s, 3H); $^{13}C\{1H\}$ NMR (101 MHz, Chloroform-*d*) δ 192.2, 158.4, 148.0, 144.8, 144.0, 140.5, 136.3, 133.9, 133.0, 131.8, 131.2, 130.8, 127.8, 127.0, 126.3, 124.0, 123.7, 123.5, 123.0, 121.9, 120.7, 110.2, 21.5; HRMS (ESI, *m/z*) calculated for $C_{24}H_{16}BrN_2O$ $(M + H)^+$ 427.0446, found 427.0447.

6'-methoxy-3-methyl-2'*H*-spiro [benzo [4, 5] imidazo [2, 1-*a*] isoindole-11, 1'-naphthalen]-2'-one (3t)

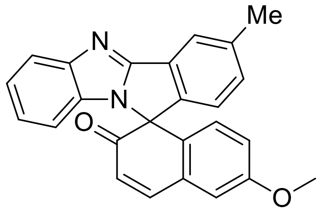

Flash chromatography for purification: 100% DCE; Light brown solid, yield: 70 mg, 77%; ^{1}H NMR (400 MHz, Acetonitrile-d_3) δ 7.92 – 7.83 (m, 2H), 7.79 (d, *J* = 8.1 Hz, 1H), 7.25-7.20 (m, 4H), 7.12 (t, J = 7.6 Hz, 1H), 6.82 (d, J = 7.9 Hz,1H), 6.73 (d, J = 10.4 Hz, 1H), 6.50 (d, J = 8.6 Hz, 1H), 6.38 (d, J = 10.1 Hz, 1H), 3.78 (s, 3H), 2.41(s, 3H).$^{13}C\{1H\}$ NMR (101 MHz, Chloroform-*d*) δ 193.2, 162.2, 159.0, 149.0, 146.7, 145.0, 140.2, 140.1, 132.3, 131.3, 131.0, 127.5, 124.2, 123.6, 123.4, 123.1, 122.7, 122.4, 122.1, 120.9, 114.1, 112.8, 110.4, 55.5, 21.6; HRMS (ESI, *m/z*) calculated for $C_{25}H_{19}N_2O_2$ $(M + H)^+$ 379.1447, found 379.1441.

Methyl 3-methyl-2'-oxo-2'*H*-spiro [benzo [4, 5] imidazo [2, 1-*a*] isoindole-11, 1'-naphthalene]-6'-carboxylate (3u)

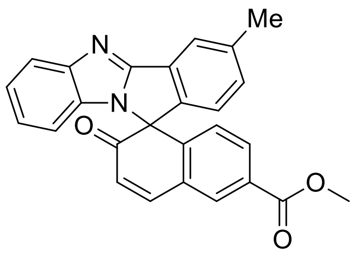

Flash chromatography for purification: 100% DCE; Brown solid, yield: 78 mg, 80%; ^{1}H NMR (400 MHz, Acetone-d_6) δ 8.42 (d, *J* = 1.8 Hz, 1H), 8.28 (d, *J* = 10.1 Hz, 1H), 8.00 – 7.90 (m, 2H), 7.87 (d, *J* = 8.1 Hz, 1H), 7.42 (d, *J* = 8.0 Hz, 1H), 7.30 (d, *J* = 8.0 Hz, 2H), 7.19 (t, *J* = 7.6 Hz, 1H), 6.96 (d, *J* = 8.0 Hz, 1H), 6.83 (d, *J* = 8.1 Hz, 1H), 6.59 (d, *J* = 10.1 Hz, 1H), 3.95 (s, 3H), 2.50 (s, 3H). $^{13}C\{1H\}$ NMR (101 MHz, Chloroform-*d*) δ 192.3, 165.6, 158.6, 148.8, 145.5, 143.8, 142.3, 140.5, 131.9, 131.5, 131.3, 131.0, 131.0, 130.3, 127.4, 126.4, 126.0, 123.8, 123.5, 123.0, 121.9,

120.9, 110.1, 52.5, 21.5; HRMS (ESI, *m/z*) calculated for $C_{26}H_{19}N_2O_3$ (M + H)$^+$ 407.1396, found 407.1393.

7'-bromo-3-methyl-2'*H*-spiro [benzo [4, 5] imidazo [2, 1-*a*] isoindole-11, 1'-naphthalen]-2'-one (3v)

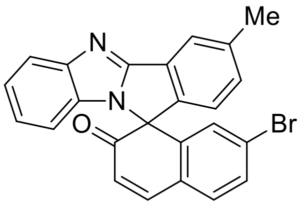

Flash chromatography for purification: 100% DCE; Light brown solid, yield: 81 mg, 79%; ^{1}H NMR (400 MHz, DMSO-d_6) δ 8.14 (d, *J* = 10.1 Hz, 1H), 7.86 (s, 1H), 7.83 – 7.78 (m, 1H), 7.76 (s, 1H), 7.73 (dd, *J* = 8.2, 1.9 Hz, 1H), 7.30 – 7.23 (m, 3H), 7.18 – 7.11 (m, 1H), 6.90 (d, *J* = 7.9 Hz, 1H), 6.69 (d, *J* = 1.8 Hz, 1H), 6.52 (d, *J* = 10.0 Hz, 1H), 2.40 (s, 3H).^{13}C{1H} NMR (101 MHz, Chloroform-*d*) δ 192.1, 158.7, 148.7, 145.4, 144.1, 140.5, 139.6, 132.8, 131.6, 131.1, 131.0, 129.2, 128.9, 127.3, 126.0, 125.3, 123.8, 123.6, 122.8, 122.0, 120.9, 110.2, 73.4, 21.5; HRMS (ESI, *m/z*) calculated for $C_{24}H_{16}BrN_2O$ (M + H)$^+$ 427.0446, found 427.0448.

2,3-dihydrobenzo[4,5]imidazo[1,2-*f*]phenanthridin-4(1*H*)-one (5a)

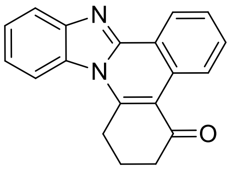

3aa

Flash chromatography for purification: 30% ethyl acetate/*n*-hexane; Off-white solid, 0.14 g, 95%; ^{1}H NMR (600 MHz, $CDCl_3$): δ 9.32 (d, *J* = 8.4 Hz, 1H), 8.82 (d, *J* = 7.9 Hz, 1H), 8.05 (d, *J* = 8.4 Hz, 1H), 8.01 (d, *J* = 8.0 Hz, 1H), 7.72 (t, *J* = 7.7 Hz, 1H), 7.64 (t, *J* = 7.5 Hz, 1H), 7.53 (t, *J* = 7.6 Hz, 1H), 7.37 (t, *J* = 7.8 Hz, 1H), 3.71 (t, *J* = 6.2 Hz, 2H), 2.86 – 2.76 (m, 2H), 2.39 (p, *J* = 6.3 Hz, 2H); ^{13}C{1H} NMR (150 MHz, $CDCl_3$): δ 197.4, 148.5, 148.3, 145.2, 131.4, 131.1, 128.8, 127.9, 126.8, 125.4, 124.9, 122.6, 122.2, 120.5, 115.2, 114.8, 38.9, 29.3, 21.0; HRMS (ESI, *m/z*) calculated for $C_{19}H_{15}N_2O$ (M + H)$^+$ 287.1184, found 287.1174.

8-methyl-2,3-dihydrobenzo[4,5]imidazo[1,2-*f*]phenanthridin-4(1*H*)-one (5b)

Flash chromatography for purification: 30% ethyl acetate/*n*-hexane; Pale yellow solid, 0.158 g, 73%; ^{1}H NMR (600 MHz, $CDCl_3$): δ 9.16 (d, *J* = 8.4

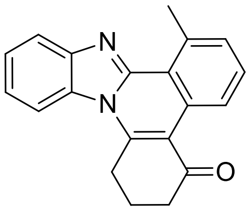

3ab

Hz, 1H), 8.04 (d, J = 8.4 Hz, 1H), 8.01 (d, J = 8.1 Hz, 1H), 7.57 (t, J = 7.9 Hz, 1H), 7.51 (t, J = 7.6 Hz, 1H), 7.43 (d, J = 7.3 Hz, 1H), 7.37 (t, J = 7.8 Hz, 1H), 3.67 (t, J = 6.2 Hz, 2H), 3.20 (s, 3H), 2.84 – 2.73 (m, 2H), 2.35 (p, J = 6.3 Hz, 2H); ^{13}C{1H} NMR (150 MHz, $CDCl_3$): δ 197.3, 148.5, 148.2, 145.1, 138.4, 130.8, 130.4, 130.0, 129.8, 124.9, 124.2, 122.5, 121.3, 120.8, 115.2, 115.1, 39.1, 29.5, 25.4, 21.0; HRMS (ESI, *m/z*) calculated for $C_{20}H_{17}N_2O$ $(M + H)^+$ 301.1341, found 301.1333.

8-ethyl-2,3-dihydrobenzo[4,5]imidazo[1,2-*f*]phenanthridin-4(1*H*)-one (5c)

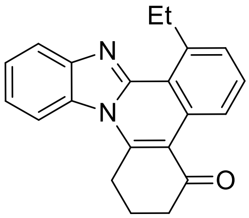

3ac

Flash chromatography for purification: 30% ethyl acetate/*n*-hexane; Brown solid, 0.170 g, 80%; ^{1}H NMR (600 MHz, $CDCl_3$): δ 9.13 (d, J = 8.4 Hz, 1H), 7.96 (t, J = 8.9 Hz, 2H), 7.58 (t, J = 7.8 Hz, 1H), 7.47 (t, J = 7.6 Hz, 1H), 7.44 (d, J = 7.3 Hz, 1H), 7.30 (t, J = 7.7 Hz, 1H), 3.76 (q, J = 7.3 Hz, 2H), 3.58 (t, J = 6.0 Hz, 2H), 2.79 – 2.72 (m, 2H), 2.31 (p, J = 6.2 Hz, 2H), 1.38 (t, J = 7.4 Hz, 3H); ^{13}C{1H} NMR (150 MHz, $CDCl_3$): δ 197.3, 148.0, 147.7, 145.1, 144.5, 130.3, 130.1, 130.0, 129.1, 124.8, 124.2, 122.4, 120.7, 120.5, 115.1, 115.1, 39.0, 29.6, 29.4, 21.0, 15.4; HRMS (ESI, *m/z*) calculated for $C_{21}H_{19}N_2O$ $(M + H)^+$ 315.1497, found 315.1498.

8-chloro-2,3-dihydrobenzo[4,5]imidazo[1,2-*f*]phenanthridin-4(1*H*)-one (5d)

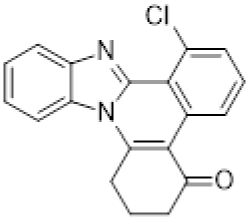

3ad

Flash chromatography for purification: 30% ethyl acetate/*n*-hexane; Yellow solid, 0.177 g, 84%; ^{1}H NMR (400 MHz, $CDCl_3$): δ 9.30 (d, J = 8.4 Hz, 1H), 8.12 (dd, J = 19.6, 8.3 Hz, 2H), 7.74 (d, J = 7.0 Hz, 1H), 7.58 (dt, J = 17.8, 8.0 Hz, 2H), 7.42 (t, J = 7.5 Hz, 1H), 3.76 (t, J = 6.2 Hz, 2H), 2.88 – 2.80 (m, 2H), 2.40 (p, J = 6.3 Hz, 2H); ^{13}C{1H} NMR (100 MHz, $CDCl_3$): δ 196.9, 149.1, 145.9, 144.8, 132.2, 131.5, 131.1, 130.3, 125.4, 123.4, 121.5, 119.9, 115.0, 114.6, 39.0, 29.6, 20.9; HRMS (ESI, *m/z*) calculated for $C_{19}H_{14}ClN_2O$ $(M + H)^+$ 321.0795, found 321.0792.

8-(trifluoromethyl)-2,3-dihydrobenzo[4,5]imidazo[1,2-*f*]phenanthridin-4(1*H*)-one (5e)

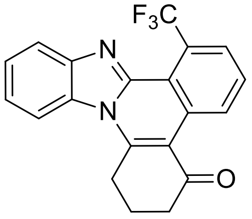

3ae

Flash chromatography for purification: 30% ethyl acetate/*n*-hexane; Pale yellow solid, 0.094 g, 68%; ^{1}H NMR (600 MHz, $CDCl_3$): δ 9.58 (d, *J* = 8.3 Hz, 1H), 8.10 (t, *J* = 8.4 Hz, 2H), 8.06 (d, *J* = 8.5 Hz, 1H), 7.77 (t, *J* = 7.8 Hz, 1H), 7.54 (t, *J* = 7.6 Hz, 1H), 7.40 (t, *J* = 8.0 Hz, 1H), 3.75 (t, *J* = 6.0 Hz, 2H), 2.92 – 2.77 (m, 2H), 2.40 (p, *J* = 6.3 Hz, 2H); ^{13}C{1H} NMR (150 MHz, $CDCl_3$): δ 196.9, 149.4, 144.9, 144.5, 131.1, 130.8, 130.5, 129.3, 127.4 (q, *J* = 7.5 Hz), 126.7, 125.5,124.1 (q, *J* = 284.1 Hz), 123.4, 121.6, 120.1, 115.0, 114.2, 39.0, 29.5, 20.9; ^{19}F NMR (376 MHz, $CDCl_3$): δ -58.9; HRMS (ESI, *m/z*) calculated for $C_{20}H_{14}F_3N_2O$ $(M + H)^+$ 355.1058, found 355.1057.

8-phenyl-2,3-dihydrobenzo[4,5]imidazo[1,2-*f*]phenanthridin-4(1*H*)-one (5f)

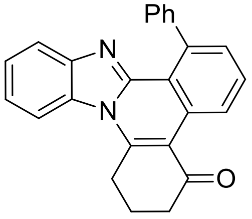

3af

Flash chromatography for purification: 30% ethyl acetate/*n*-hexane; Off-white solid, 0.121 g, 90%; ^{1}H NMR (600 MHz, $CDCl_3$): δ 9.33 (d, *J* = 8.4 Hz, 1H), 7.87 (d, *J* = 8.3 Hz, 1H), 7.72 – 7.65 (m, 1H), 7.56 (d, *J* = 8.1 Hz, 1H), 7.46 (dd, *J* = 11.7, 5.6 Hz, 4H), 7.41 (d, *J* = 3.5 Hz, 2H), 7.31 (t, *J* = 7.6 Hz, 1H), 7.19 (t, *J* = 7.6 Hz, 1H), 3.59 (t, *J* = 5.9 Hz, 2H), 2.84 – 2.74 (m, 2H), 2.33 (p, *J* = 6.1 Hz, 2H); ^{13}C{1H} NMR (150 MHz, $CDCl_3$): δ 197.4, 148.7, 147.1, 144.5, 143.5, 141.5, 131.2, 130.3, 130.0, 129.6, 129.4, 127.5, 126.7, 125.9, 124.7, 122.4, 121.0, 119.9, 114.8, 114.8, 39.1, 29.4, 21.0; HRMS (ESI, *m/z*) calculated for $C_{25}H_{19}N_2O$ $(M + H)^+$ 363.1497, found 363.1490.

7-methyl-2,3-dihydrobenzo[4,5]imidazo[1,2-*f*]phenanthridin-4(1*H*)-one (5g)

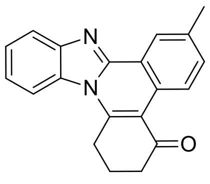

3ag

Flash chromatography for purification: 30% ethyl acetate/*n*-hexane; Pale yellow solid, 0.209 g, 90%; ^{1}H NMR (600 MHz, $CDCl_3$): δ 9.07 (d, *J* = 8.5 Hz, 1H), 8.44 (s, 1H), 7.91 (d, *J* = 8.0 Hz, 1H), 7.85 (d, *J* = 8.4 Hz, 1H), 7.44 (t, *J* = 7.6 Hz, 1H), 7.41 (d, *J* = 8.6 Hz, 1H), 7.24 (t, *J* = 7.8 Hz, 1H), 3.48 (t, *J* = 6.2 Hz, 2H), 2.80 – 2.64 (m, 2H), 2.44 (s, 3H), 2.29 (p, *J* = 6.3 Hz, 2H); ^{13}C{1H} NMR (150 MHz, $CDCl_3$): δ 197.5, 148.0, 147.5, 145.0, 137.9, 132.4, 131.3, 126.4, 126.3, 125.2, 124.3, 122.3, 121.9, 120.2, 115.1, 114.5, 38.8, 29.0, 21.3, 20.8; HRMS (ESI, *m/z*) calculated for $C_{20}H_{17}N_2O$ $(M + H)^+$ 301.1341, found 301.1340.

7-methoxy-2,3-dihydrobenzo[4,5]imidazo[1,2-*f*]phenanthridin-4(1*H*)-one (5h)

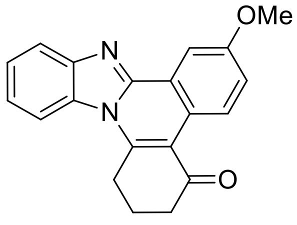

3ah

Flash chromatography for purification: 30% ethyl acetate/*n*-hexane; Pale yellow solid, 0.200 g, 94%; ^{1}H NMR (600 MHz, $CDCl_3$): δ 9.27 (d, *J* = 9.3 Hz, 1H), 8.19 (d, *J* = 2.8 Hz, 1H), 8.09 (d, *J* = 8.4 Hz, 1H), 8.03 (d, *J* = 8.1 Hz, 1H), 7.55 (t, *J* = 7.6 Hz, 1H), 7.39 (t, *J* = 8.2 Hz, 1H), 7.32 (dd, *J* = 9.2, 2.9 Hz, 1H), 4.02 (s, 3H), 3.72 (t, *J* = 6.2 Hz, 2H), 2.86 – 2.74 (m, 2H), 2.39 (p, *J* = 6.3 Hz, 2H); ^{13}C{1H} NMR (150 MHz, $CDCl_3$): δ 197.6, 159.0, 148.1, 146.2, 145.1, 131.6, 128.6, 125.4, 123.8, 122.9, 122.5, 121.2, 120.4, 115.3, 114.9, 105.1, 55.7, 38.9, 29.2, 21.1; HRMS (ESI, *m/z*) calculated for $C_{20}H_{17}N_2O_2$ $(M + H)^+$ 317.1290, found 317.1288.

7-(trifluoromethyl)-2, 3-dihydrobenzo [4, 5] imidazo [1, 2-*f*] phenanthridin-4(1H)-one (5i)

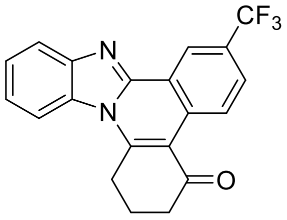

Flash chromatography for purification: 30% ethyl acetate/*n*-hexane; White solid, yield: 47 mg, 70%; ^{1}H NMR (600 MHz, $CDCl_3$) δ 9.44 (d, *J* = 8.8 Hz, 1H), 9.07 (s, 1H), 8.01 (d, *J* = 8.4 Hz, 1H), 7.97 (d, *J* = 8.1 Hz, 1H), 7.88 (d, *J* = 8.8 Hz, 1H), 7.52 (t, *J* = 7.6 Hz, 1H), 7.38 (t, *J* = 7.7 Hz, 1H), 3.70 (t, *J* = 5.9 Hz, 2H), 2.83 (t, *J* = 6.4 Hz, 2H), 2.42 (p, *J* = 5.8 Hz, 2H); ^{13}C{1H} NMR (151 MHz, $CDCl_3$) δ 196.9, 150.1, 146.8, 131.1, 131.0, 129.7 (q, $^1J_{CF}$ = 33.1 Hz, CF_3), 127.7, 127.0, 126.0, 124.6, 123.4, 122.8, 122.2, 121.6, 120.3, 115.3, 114.2, 38.7, 29.3, 20.7; ^{19}F NMR (564 MHz, $CDCl_3$) δ -62.5; HRMS (ESI, *m/z*) calculated for $C_{21}H_{17}N_2O_3$ $(M + H)^+$ 355.1053, found 355.1057.

6-methyl-2,3-dihydrobenzo[4,5]imidazo[1,2-*f*]phenanthridin-4(1*H*)-one (5j)

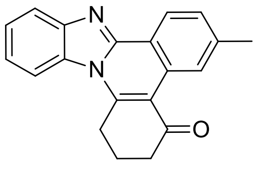

3ai

Flash chromatography for purification: 30% ethyl acetate/*n*-hexane; Brown solid, 0.191 g, 88%; ^{1}H NMR (600 MHz, $CDCl_3$): δ 9.13 (s, 1H), 8.71 (d, *J* = 8.1 Hz, 1H), 8.04 (d, *J* = 8.4 Hz, 1H), 7.99 (d, *J* = 8.1 Hz, 1H), 7.52 (t, *J* = 7.6 Hz, 1H), 7.47 (d, *J* = 8.1 Hz, 1H), 7.36 (t, *J* = 7.8 Hz, 1H), 3.71 (t, *J* = 6.2 Hz, 2H), 2.86 – 2.77 (m, 2H), 2.56 (s, 3H), 2.39 (d, *J* = 6.2 Hz, 2H); ^{13}C{1H} NMR (150 MHz, $CDCl_3$): δ 197.5, 151.7, 148.5, 145.2, 141.6, 131.4, 129.7, 129.4, 128.9, 126.6, 126.4, 125.4, 124.8, 122.3, 120.3, 115.1, 39.0, 29.3, 22.4, 21.0; HRMS (ESI, *m/z*) calculated for $C_{20}H_{17}N_2O$ $(M + H)^+$ 301.1341, found 301.1339.

6-chloro-2,3-dihydrobenzo[4,5]imidazo[1,2-*f*]phenanthridin-4(1*H*)-one (5k)

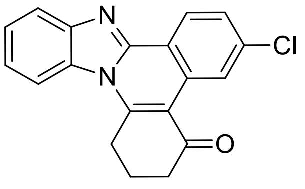

3aj

Flash chromatography for purification: 30% ethyl acetate/*n*-hexane; Pale yellow solid, 0.112 g, 80%; ^{1}H NMR (400 MHz, $CDCl_3$): δ 9.14 (d, *J* = 2.0 Hz, 1H), 8.85 (d, *J* = 8.7 Hz, 1H), 8.27 (d, *J* = 8.0 Hz, 1H), 7.84 (t, *J* = 6.8 Hz, 1H), 7.78 (d, *J* = 8.1 Hz, 1H), 7.52 (dd, *J* = 8.7, 1.9 Hz, 1H), 7.48 (d, *J* = 6.8 Hz, 1H), 3.50 (t, *J* = 6.1 Hz, 2H), 2.80 – 2.72 (m, 2H), 2.06 (p, *J* = 6.3 Hz, 2H); ^{13}C{1H} NMR (100 MHz, $CDCl_3$): δ 197.4, 162.0, 151.9, 146.5, 146.0, 139.2, 135.3, 130.8, 128.7, 128.4, 127.0, 126.5, 126.2, 125.3, 120.1, 117.3, 39.2, 31.3, 22.4; HRMS (ESI, *m/z*) calculated for $C_{19}H_{14}ClN_2O$ $(M + H)^+$ 321.0795, found 321.0791.

6-(trifluoromethyl)-2,3-dihydrobenzo[4,5]imidazo[1,2-*f*]phenanthridin-4(1*H*)-one (5l)

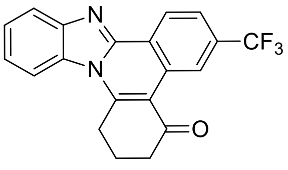

3ak

Flash chromatography for purification: 30% ethyl acetate/*n*-hexane; Pale yellow solid, 0.152 g, 75%; ^{1}H NMR (400 MHz, $CDCl_3$): δ 9.73 (s, 1H), 8.91 (d, *J* = 8.4 Hz, 1H), 8.08 (d, *J* = 8.5 Hz, 1H), 8.04 (d, *J* = 8.1 Hz, 1H), 7.83 (d, *J* = 8.4 Hz, 1H), 7.58 (t, *J* = 7.4 Hz, 1H), 7.44 (t, *J* = 8.4 Hz, 1H), 3.75 (t, *J* = 6.2 Hz, 2H), 2.88 – 2.80 (m, 2H), 2.42 (p, *J* = 6.3 Hz, 2H); ^{13}C{1H} NMR (100 MHz, $CDCl_3$): δ 197.0, 149.7, 147.1, 145.1, 132.4 (q, *J* = 32 Hz), 131.4, 128.7, 125.9, 125.5, 125.4, 124.5 (q, *J* = 3.43 Hz), 124.0 (q, *J* = 4.17 Hz), 123.4, 122.7, 120.9, 115.3, 114.0, 38.7, 29.7, 29.3, 20.8; ^{19}F NMR (376 MHz, $CDCl_3$): δ -62.5; HRMS (ESI, *m/z*) calculated for $C_{20}H_{14}F_3N_2O$ $(M + H)^+$ 355.1058, found 355.1057.

6-methoxy-2,3-dihydrobenzo[4,5]imidazo[1,2-*f*]phenanthridin-4(1*H*)-one (5m)

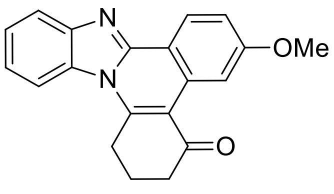

3al

Flash chromatography for purification: 30% ethyl acetate/*n*-hexane; Off-white solid, 0.113 g, 80%; ^{1}H NMR (600 MHz, $CDCl_3$): δ 8.90 (d, *J* = 2.5 Hz, 1H), 8.69 (d, *J* = 8.8 Hz, 1H), 7.97 (d, *J* = 8.4 Hz, 1H), 7.95 (d, *J* = 8.1 Hz, 1H), 7.50 (t, *J* = 7.6 Hz, 1H), 7.31 (t, *J* = 7.6 Hz, 1H), 7.21 (dd, *J* = 8.8, 2.6 Hz, 1H), 3.96 (s, 3H), 3.66 (t, *J* = 6.2 Hz, 2H), 2.84 – 2.76 (m, 2H), 2.38 (p, *J* = 6.3 Hz, 2H); ^{13}C{1H} NMR (150 MHz, $CDCl_3$): δ 197.7, 162.0, 149.2, 148.4, 145.4, 131.3, 130.8, 126.5, 125.3, 122.0, 120.0, 117.5, 115.9, 115.1, 114.1, 108.2, 55.5, 39.0, 29.4, 20.9; HRMS (ESI, *m/z*) calculated for $C_{20}H_{17}N_2O_2$ $(M + H)^+$ 317.1290, found 317.1288.

10-methyl-2,3-dihydrobenzo[4,5]imidazo[1,2-*f*]phenanthridin-4(1*H*)-one (5n)

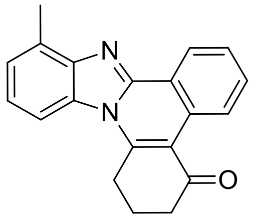

3am

Flash chromatography for purification: 30% ethyl acetate/*n*-hexane; Pale yellow solid, 0.176 g, 81%; ^{1}H NMR (400 MHz, $CDCl_3$): δ 9.17 (d, *J* = 8.4 Hz, 1H), 8.69 (d, *J* = 8.7 Hz, 1H), 7.63 (d, *J* = 8.4 Hz, 1H), 7.58 (t, *J* = 7.1 Hz, 1H), 7.48 (t, *J* = 7.1 Hz, 1H), 7.24 (d, *J* = 7.3 Hz, 1H), 7.14 (t, *J* = 7.8 Hz, 1H), 3.43 (t, *J* = 6.2 Hz, 2H), 2.76 (s, 3H), 2.73 – 2.63 (m, 2H), 2.27 (p, *J* = 6.3 Hz, 3H); ^{13}C{1H} NMR (100 MHz, $CDCl_3$): δ 197.5, 148.5, 147.2, 144.2, 130.8, 130.6, 130.3, 128.5, 127.4, 126.4, 125.5, 124.7, 122.2, 122.1, 114.3, 112.5, 38.7, 29.0, 20.8, 17.0; HRMS (ESI, *m/z*) calculated for $C_{20}H_{17}N_2O$ $(M + H)^+$ 301.1341, found 301.1339.

11-chloro-8-methyl-2,3-dihydrobenzo[4,5]imidazo[1,2-*f*]phenanthridin-4(1*H*)-one (5o)

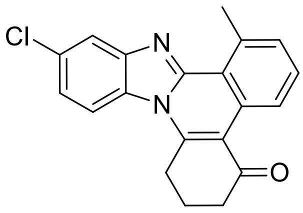

3an

Flash chromatography for purification: 30% ethyl acetate/*n*-hexane; Pale yellow solid, 0.108 g, 78%; ^{1}H NMR (600 MHz, $CDCl_3$): δ 9.14 (d, *J* = 8.4 Hz, 1H), 7.95 (d, *J* = 2.1 Hz, 1H), 7.91 (d, *J* = 8.9 Hz, 1H), 7.60 – 7.54 (m, 1H), 7.42 (d, *J* = 7.3 Hz, 1H), 7.29 (dd, *J* = 8.9, 2.1 Hz, 1H), 3.59 (t, *J* = 6.2 Hz, 2H), 3.13 (s, 3H), 2.81 – 2.77 (m, 2H), 2.35 (p, *J* = 6.3 Hz, 2H); ^{13}C{1H} NMR (150 MHz, $CDCl_3$): δ 197.2, 149.5, 147.5, 146.0, 138.6, 130.9, 130.5, 130.2, 130.0, 128.9, 124.2, 122.6, 121.0, 120.4, 115.7, 115.5, 39.0, 29.3, 25.4, 20.9; HRMS (ESI, *m/z*) calculated for $C_{20}H_{16}ClN_2O$ $(M + H)^+$ 335.0951, found 335.0949.

6,7-dihydrobenzo[4,5]imidazo[1,2-*a*]thieno[2,3-*c*]quinolin-4(5*H*)-one (5q)

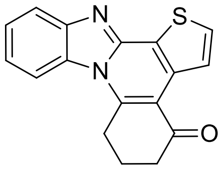

3ap

Flash chromatography for purification: 30% ethyl acetate/*n*-hexane; Pale yellow solid, 0.176 g, 80%; ^{1}H NMR (400 MHz, $CDCl_3$): δ 8.55 (d, *J* = 5.3 Hz, 1H), 8.06 (d, *J* = 8.5 Hz, 1H), 7.96 (d, *J* = 8.2 Hz, 1H), 7.73 (d, *J* = 5.3 Hz, 1H), 7.52 (t, *J* = 7.4 Hz, 1H), 7.33 (t, *J* = 7.3 Hz, 1H), 3.72 (t, *J* = 6.2 Hz, 2H), 2.83 – 2.75 (m, 2H), 2.42 (p, *J* = 6.3 Hz, 2H); ^{13}C{1H} NMR (100 MHz, $CDCl_3$): δ 195.9, 147.5, 145.6, 145.6, 136.0, 130.8, 130.5, 126.6, 126.1, 125.8, 122.1, 120.2, 115.1, 114.7, 37.6, 28.5, 21.3; HRMS (ESI, *m/z*) calculated for $C_{17}H_{13}N_2OS$ $(M + H)^+$ 293.0749, found 293.0744.

1-methyl-1,5,6,7-tetrahydro-4*H*-benzo[4,5]imidazo[1,2-*a*]pyrrolo[2,3-*c*]quinolin-4-one (5r)

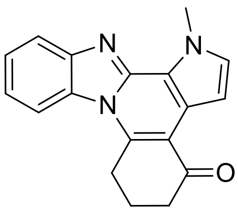

3aq

Flash chromatography for purification: 30% ethyl acetate/*n*-hexane; Yellow solid, 0.181 g, 82%; ^{1}H NMR (600 MHz, $CDCl_3$): δ 7.98 (d, *J* = 8.3 Hz, 1H), 7.90 (d, *J* = 8.1 Hz, 1H), 7.48 (t, *J* = 7.6 Hz, 1H), 7.28 (d, *J* = 2.7 Hz, 2H), 6.95 (d, *J* = 2.7 Hz, 1H), 4.22 (s, 3H), 3.48 (t, *J* = 6.1 Hz, 2H), 2.72 – 2.62 (m, 2H), 2.30 (p, *J* = 6.2 Hz, 2H); ^{13}C{1H} NMR (150 MHz, $CDCl_3$): δ 197.0, 145.7, 143.4, 142.8, 130.6, 130.4, 124.9, 121.5, 120.9, 120.2, 119.5, 115.0, 113.7, 104.9, 37.4, 36.1, 28.1, 21.7; HRMS (ESI, *m/z*) calculated for $C_{18}H_{16}N_3O$ $(M + H)^+$ 290.1293, found 290.1290.

7,8-dihydrobenzo[*j*]benzo[4,5]imidazo[1,2-*f*]phenanthridin-9(6*H*)-one (5s)

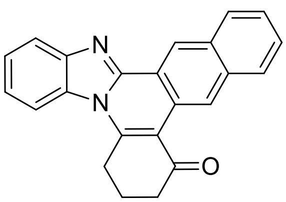

3ar

Flash chromatography for purification: 30% ethyl acetate/*n*-hexane; Yellow solid, 0.184 g, 89%; ^{1}H NMR (400 MHz, $CDCl_3$): δ 9.87 (s, 1H), 9.33 (s, 1H), 8.03 (dt, *J* = 17.1, 8.3 Hz, 4H), 7.67 – 7.48 (m, 3H), 7.38 (t, *J* = 7.8 Hz, 1H), 3.70 (t, *J* = 6.2 Hz, 2H), 2.90 – 2.78 (m, 2H), 2.41 (p, *J* = 5.9 Hz, 2H); ^{13}C{1H} NMR (100 MHz, $CDCl_3$): δ 197.6, 148.6, 148.5, 145.0, 134.6, 131.9, 129.3, 128.2, 127.3, 126.9, 126.7, 125.2, 124.7, 123.0, 120.5, 114.9, 114.8, 39.0, 29.4, 21.0; HRMS (ESI, *m/z*) calculated for $C_{23}H_{17}N_2O$ $(M + H)^+$ 337.1341, found 337.1336.

6,7-dihydroimidazo[1,2-*f*]phenanthridin-8(5*H*)-one (5t)

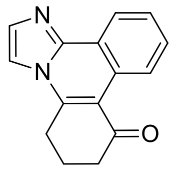

3as

Flash chromatography for purification: 30% ethyl acetate/*n*-hexane; Pale yellow solid, 0.215 g, 87%; ^{1}H NMR (600 MHz, $CDCl_3$): δ 9.30 (d, *J* = 8.3 Hz, 1H), 8.64 (d, *J* = 7.5 Hz, 1H), 7.67 (s, 1H), 7.63 (dt, *J* = 15.7, 7.1 Hz, 2H), 7.58 (s, 1H), 3.19 (t, *J* = 6.3 Hz, 2H), 2.83 – 2.76 (m, 2H), 2.35 (p, *J* = 6.4 Hz, 2H); ^{13}C{1H} NMR (150 MHz, $CDCl_3$): δ 197.8, 144.1, 144.0, 133.3, 129.4, 127.9, 126.9, 126.7, 123.1, 122.6, 115.7, 112.0, 39.4, 27.1, 20.6; HRMS (ESI, *m/z*) calculated for $C_{15}H_{13}N_2O$ $(M + H)^+$ 237.1028, found 237.1024.

2-methyl-2,3-dihydrobenzo[4,5]imidazo[1,2-*f*]phenanthridin-4(1*H*)-one (5u)

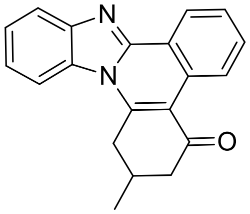

3at

Flash chromatography for purification: 30% ethyl acetate/*n*-hexane; Pale yellow solid, 0.214 g, 92%; ^{1}H NMR (400 MHz, $CDCl_3$): δ 9.26 (d, *J* = 8.4 Hz, 1H), 8.73 (d, *J* = 8.0 Hz, 1H), 7.94 (t, *J* = 8.3 Hz, 2H), 7.72 – 7.62 (m, 1H), 7.58 (t, *J* = 7.1 Hz, 1H), 7.48 (t, *J* = 7.4 Hz, 1H), 7.31 (t, *J* = 7.2 Hz, 1H), 3.70 (dd, *J* = 16.6, 3.8 Hz, 1H), 3.12 (dd, *J* = 17.2, 9.9 Hz, 1H), 2.78 (q, *J* = 11.1, 10.4 Hz, 1H), 2.50 (d, *J* = 9.9 Hz, 2H), 1.32 (d, *J* = 6.0 Hz, 3H); $^{13}C\{1H\}$ NMR (100 MHz, $CDCl_3$): δ 197.5, 148.2, 147.9, 145.0, 131.0, 128.9, 128.7, 127.8, 126.6, 125.3, 124.8, 122.5, 122.0, 120.3, 115.2, 114.2, 46.9, 37.1, 28.3, 21.2; HRMS (ESI, *m/z*) calculated for $C_{20}H_{17}N_2O$ $(M + H)^+$ 301.1341, found 301.1338.

2,2-dimethyl-2,3-dihydrobenzo[4,5]imidazo[1,2-*f*]phenanthridin-4(1*H*)-one (5v)

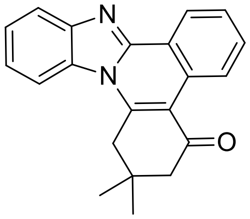

3au

Flash chromatography for purification: 30% ethyl acetate/*n*-hexane; Pale yellow solid, 0.212 g, 87%; ^{1}H NMR (600 MHz, $CDCl_3$): δ 9.21 (t, *J* = 7.6 Hz, 1H), 8.69 (t, *J* = 7.5 Hz, 1H), 7.90 (t, *J* = 6.9 Hz, 1H), 7.82 (t, *J* = 10.1 Hz, 1H), 7.62 (q, *J* = 7.0 Hz, 1H), 7.54 (q, *J* = 6.9 Hz, 1H), 7.43 (q, *J* = 7.0 Hz, 1H), 7.25 (q, *J* = 7.2 Hz, 1H), 3.29 (d, *J* = 12.8 Hz, 2H), 2.58 (d, *J* = 5.6 Hz, 2H), 1.19 (d, *J* = 4.5 Hz, 6H); $^{13}C\{1H\}$ NMR (150 MHz, $CDCl_3$): δ 197.5, 148.2, 146.6, 144.9, 131.2, 130.9, 128.4, 127.7, 127.6, 126.5, 125.3, 124.7, 122.4, 121.9, 120.2, 115.3, 113.4, 52.3, 42.6, 32.1, 28.5; HRMS (ESI, *m/z*) calculated for $C_{21}H_{19}N_2O$ $(M + H)^+$ 315.1497, found 315.1491.

2-phenyl-2,3-dihydrobenzo[4,5]imidazo[1,2-*f*]phenanthridin-4(1*H*)-one (5w)

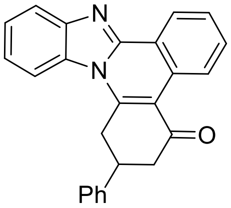

3av

Flash chromatography for purification: 30% ethyl acetate/*n*-hexane; Pale yellow solid, 0.244 g, 87%; ^{1}H NMR (600 MHz, $CDCl_3$): δ 9.31 (d, *J* = 8.4 Hz, 1H), 8.77 (d, *J* = 7.9 Hz, 1H), 7.96 (d, *J* = 8.0 Hz, 1H), 7.81 (d, *J* = 8.4 Hz, 1H), 7.69 (t, *J* = 7.7 Hz, 1H), 7.60 (t, *J* = 7.5 Hz, 1H), 7.47 (dq, *J* = 7.4, 3.4 Hz, 3H), 7.44 – 7.35 (m, 3H), 7.24 (d, *J* = 7.4 Hz, 1H), 3.95 (d, *J* = 16.3 Hz, 1H), 3.69 – 3.53 (m, 2H), 3.11 – 2.95 (m, 2H); $^{13}C\{1H\}$ NMR (150 MHz, $CDCl_3$): δ 196.7, 148.2, 147.6, 145.0, 142.0, 131.2, 131.1, 129.2, 129.0, 128.6, 128.0, 127.7, 126.8, 126.6, 126.5,

125.4, 124.8, 122.6, 122.1, 120.4, 115.2, 114.3, 45.4, 39.0, 37.1; HRMS (ESI, *m/z*) calculated for $C_{25}H_{19}N_2O$ (M + H)$^+$ 363.1497, found 363.1499.

1,2-dihydro-3*H*-benzo[4,5]imidazo[2,1-*a*]cyclopenta[*c*]isoquinolin-3-one (5x)

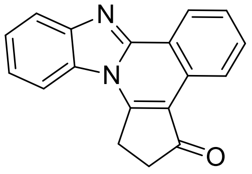

3aw

Flash chromatography for purification: 30% ethyl acetate/*n*-hexane; Off-white solid, 0.194 g, 92%; ^{1}H NMR (600 MHz, $CDCl_3$): δ 8.93 (d, *J* = 8.0 Hz, 1H), 8.74 (d, *J* = 8.0 Hz, 1H), 8.02 (d, *J* = 8.1 Hz, 1H), 7.92 (d, *J* = 8.2 Hz, 1H), 7.74 (t, *J* = 7.6 Hz, 1H), 7.67 (t, *J* = 7.6 Hz, 1H), 7.55 (t, *J* = 7.6 Hz, 1H), 7.43 (t, *J* = 7.7 Hz, 1H), 3.67 – 3.61 (m, 2H), 2.98 – 2.91 (m, 2H); ^{13}C{1H} NMR (150 MHz, $CDCl_3$): δ 201.3, 158.3, 148.6, 144.7, 131.2, 130.4, 128.5, 127.2, 125.7, 125.1, 123.8, 123.3, 121.8, 120.5, 117.9, 112.7, 35.2, 25.2; HRMS (ESI, *m/z*) calculated for $C_{18}H_{13}N_2O$ (M + H)$^+$ 273.1028, found 273.1022.

10*H*-benzo[4,5]imidazo[2,1-*a*]indeno[1,2-*c*]isoquinolin-10-one (5y)

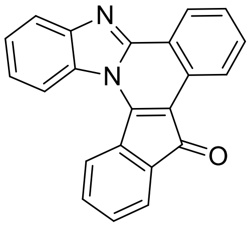

3ax

Flash chromatography for purification: 30% ethyl acetate/*n*-hexane; Red solid, 0.249 g, 78%; ^{1}H NMR (400 MHz, $CDCl_3$): δ 8.80 (d, *J* = 8.2 Hz, 1H), 8.69 (d, *J* = 7.5 Hz, 1H), 8.30 (d, *J* = 8.6 Hz, 1H), 8.08 (d, *J* = 5.4 Hz, 1H), 7.99 (d, *J* = 8.6 Hz, 1H), 7.68 (t, *J* = 7.5 Hz, 1H), 7.65 – 7.33 (m, 6H); ^{13}C{1H} NMR (100 MHz, $CDCl_3$): δ 191.0, 150.0, 145.4, 137.6, 134.2, 133.3, 132.1, 131.3, 130.9, 130.2, 128.2, 127.2, 125.9, 125.3, 124.0, 123.8, 122.6, 122.4, 122.3, 120.6, 120.3, 114.5; HRMS (ESI, *m/z*) calculated for $C_{22}H_{13}N_2O$ (M + H)$^+$ 321.1028, found 321.1024.

Spectral Data for 3a-3v and 5a-5y

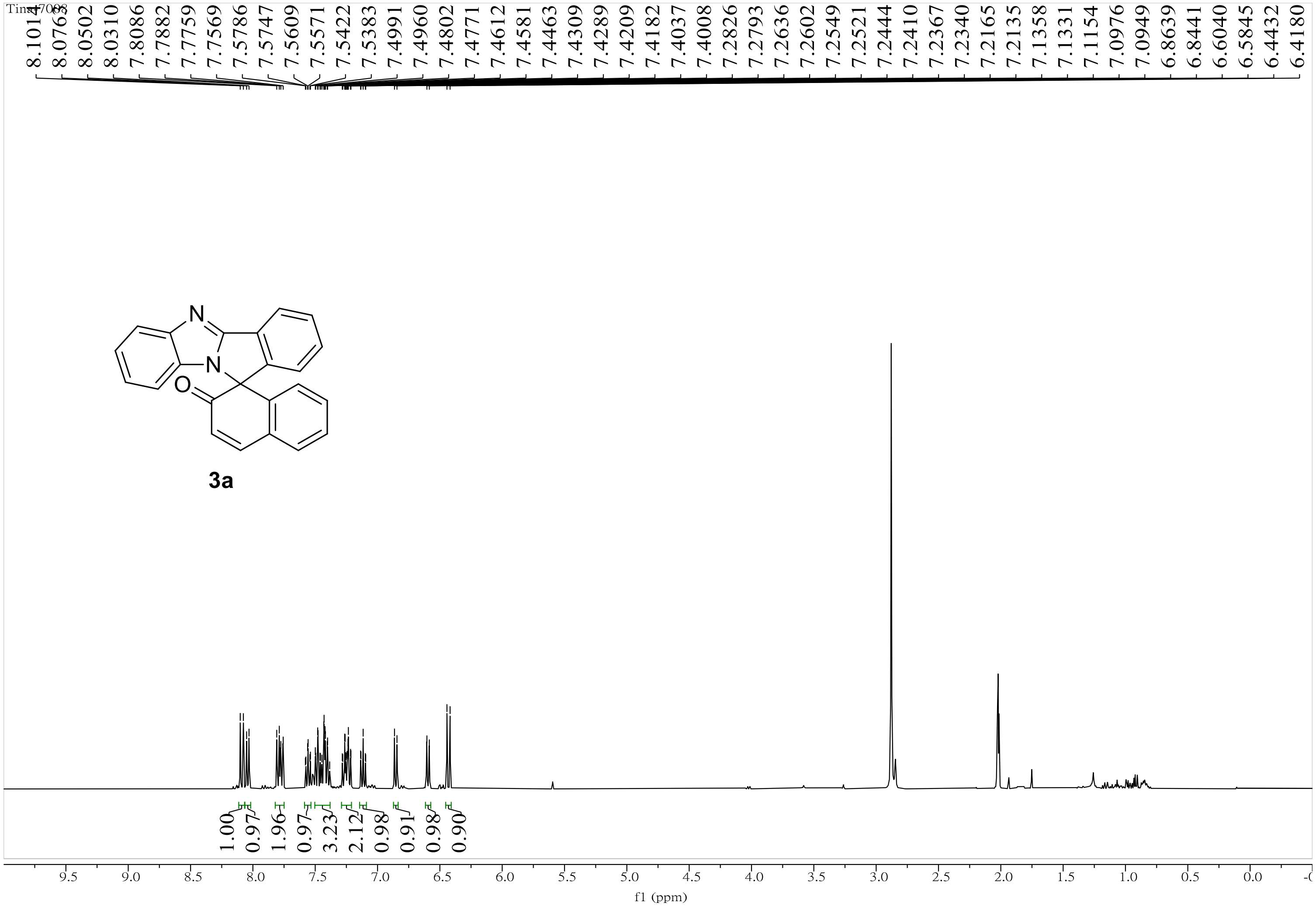

^{1}H NMR spectrum (400 MHz) of compound **3a** in acetone-d_6

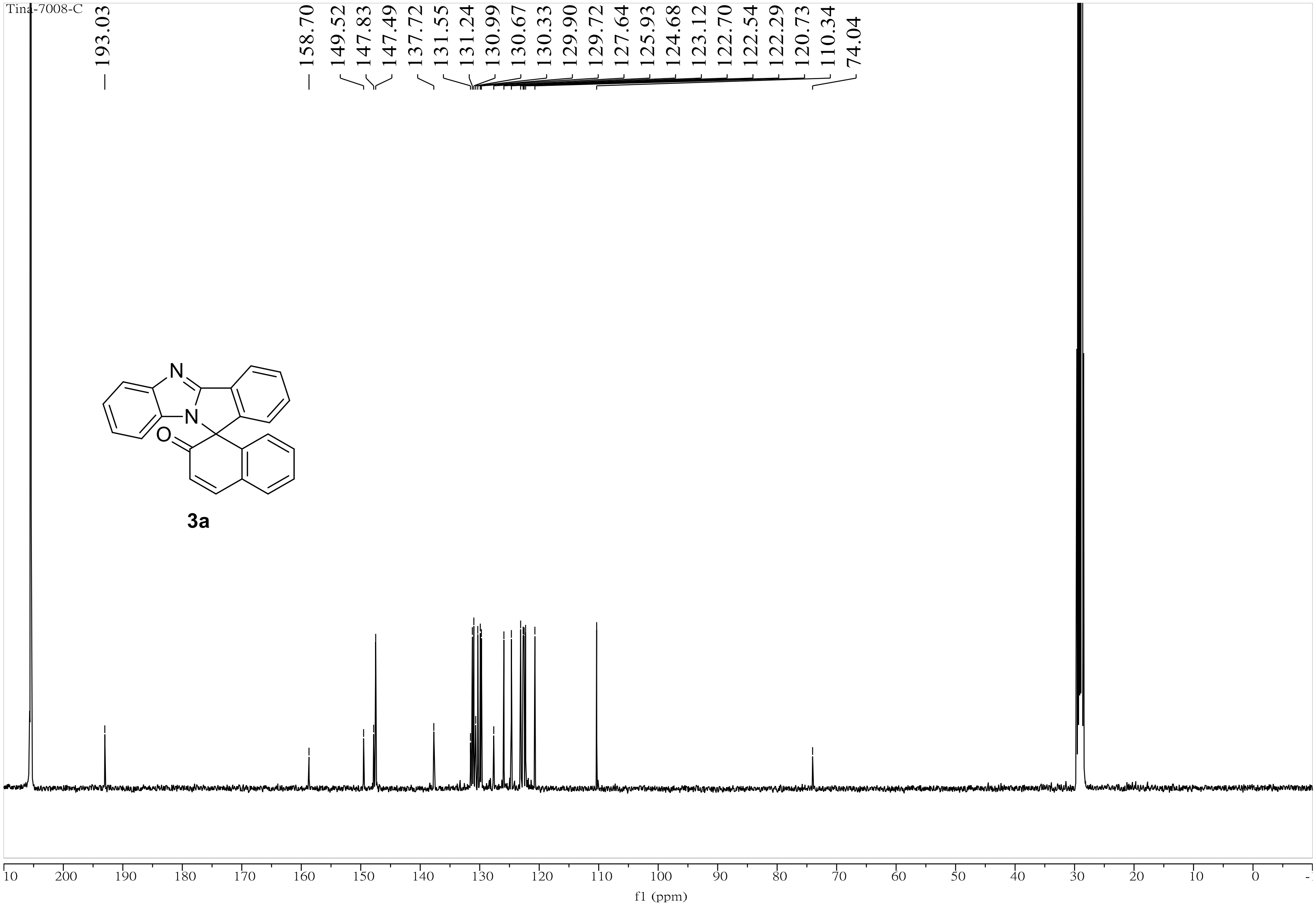

$^{13}C\{^1H\}$ NMR spectrum (101 MHz) of compound **3a** in acetone-d_6

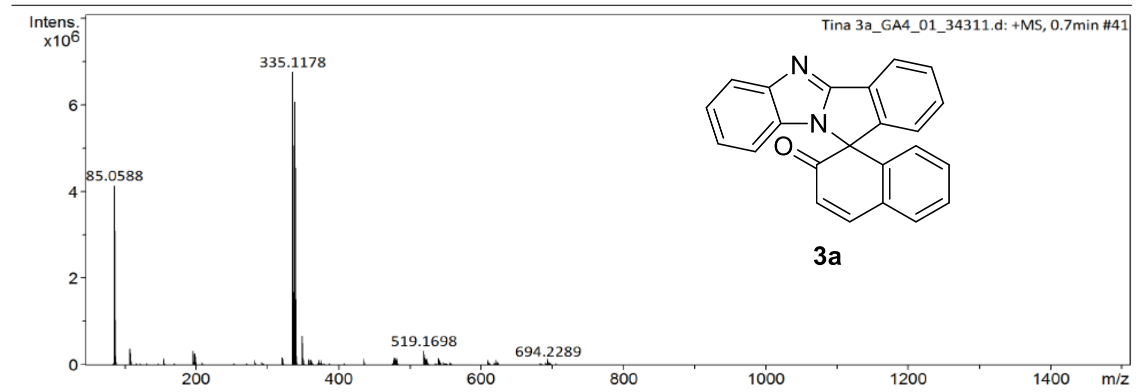

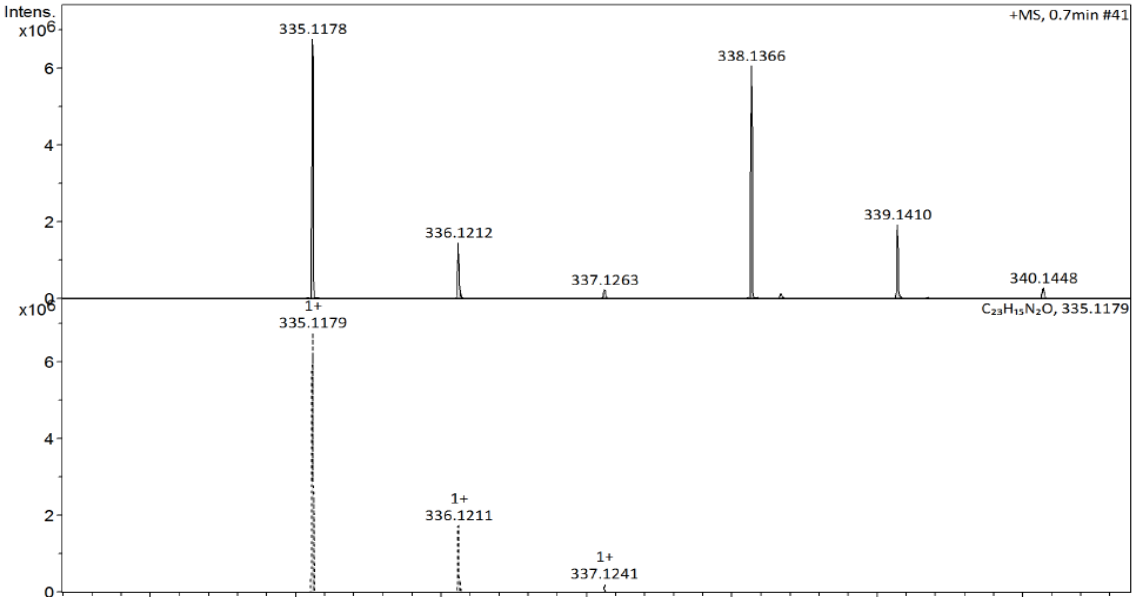

Display Report

Meas. m/z	#	Ion Formula	m/z	err [ppm]	mSigma	# Sigma	Score	rdb	e⁻ Conf	N-Rule	Adduct
335.1178	1	C23H15N2O	335.1179	-0.2	23.7	1	100.00	17.5	even	ok	M

HRMS (ESI) of compound **3a**

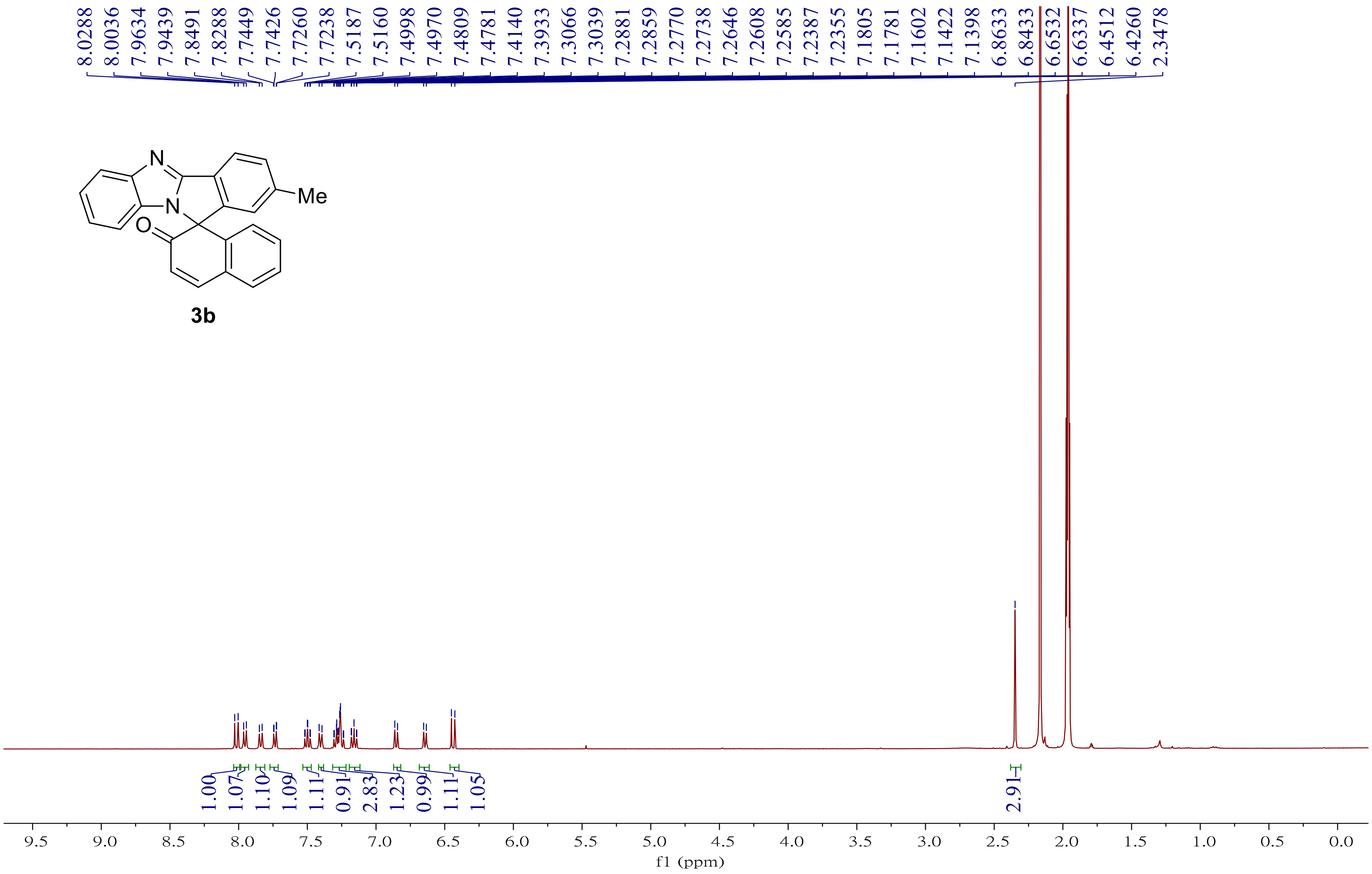

^1H NMR spectrum (400 MHz) of compound **3b** in Acetonitrile-d_3

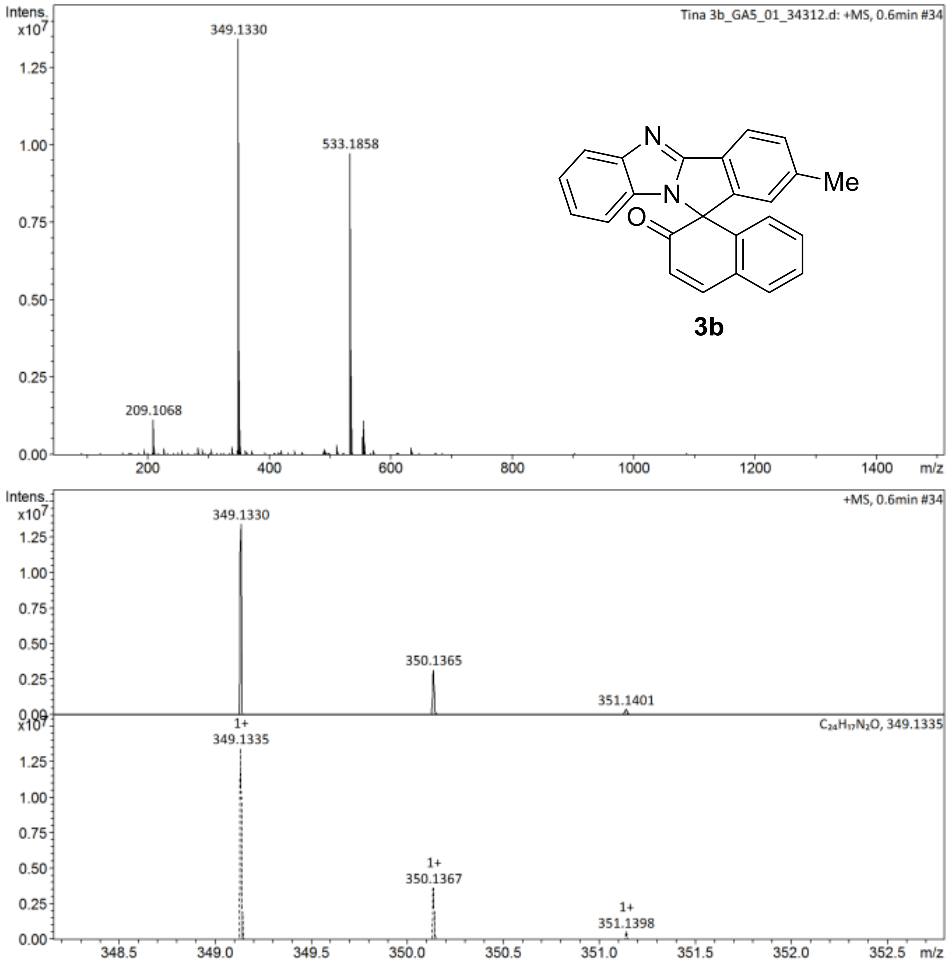

Display Report

Meas. m/z	#	Ion Formula	m/z	err [ppm]	mSigma	# Sigma	Score	rdb	e⁻ Conf	N-Rule	Adduct
349.1330	1	C24H17N2O	349.1335	1.4	19.0	1	100.00	17.5	even	ok	M

HRMS (ESI) of compound **3b**

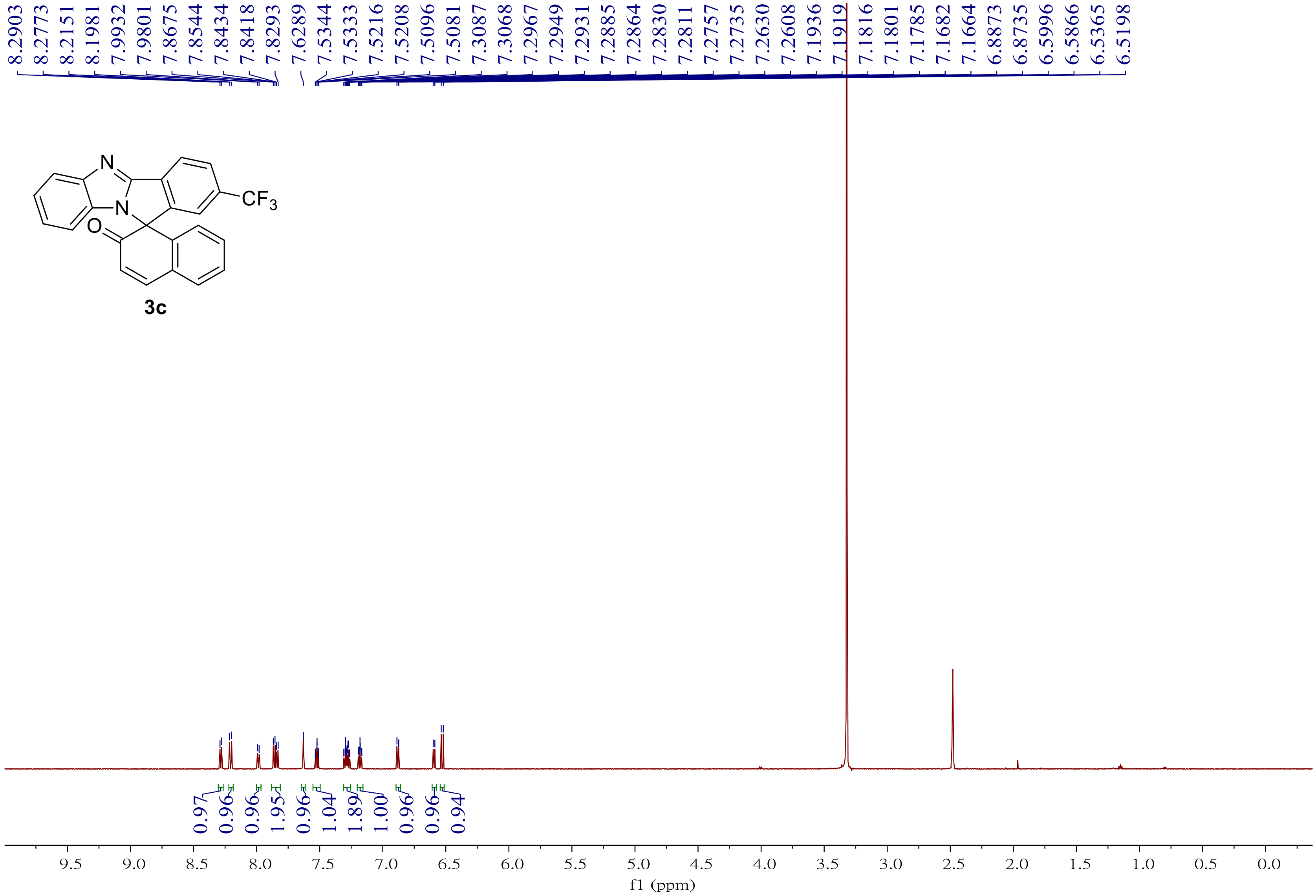

^{1}H NMR spectrum (600 MHz) of compound **3c** in DMSOd_6

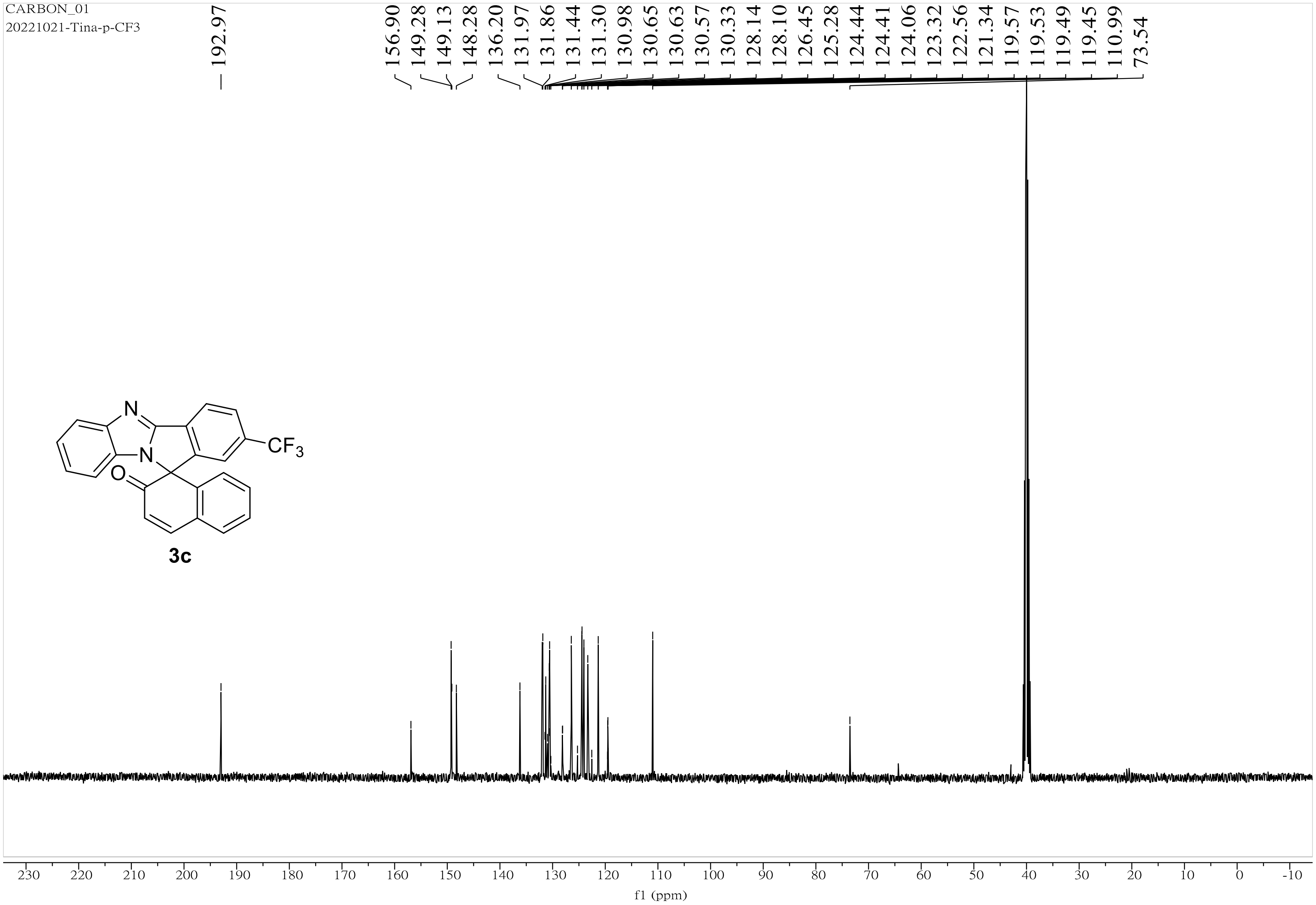

^{13}C{1H} NMR spectrum (101 MHz) of compound **3c** in DMSO-d_6

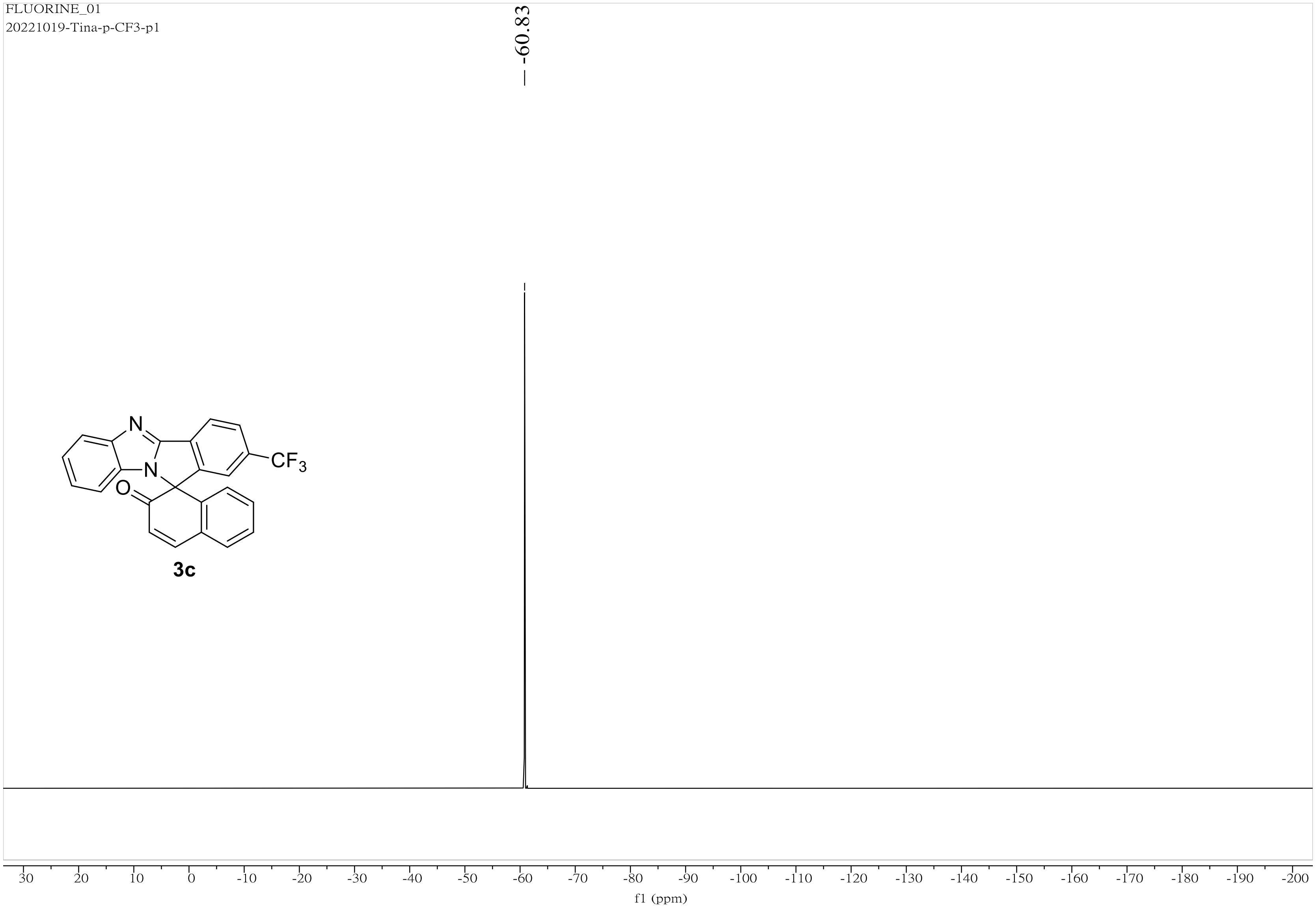

^{19}F NMR spectrum (376 MHz) of compound **3c** in DMSO-d_6

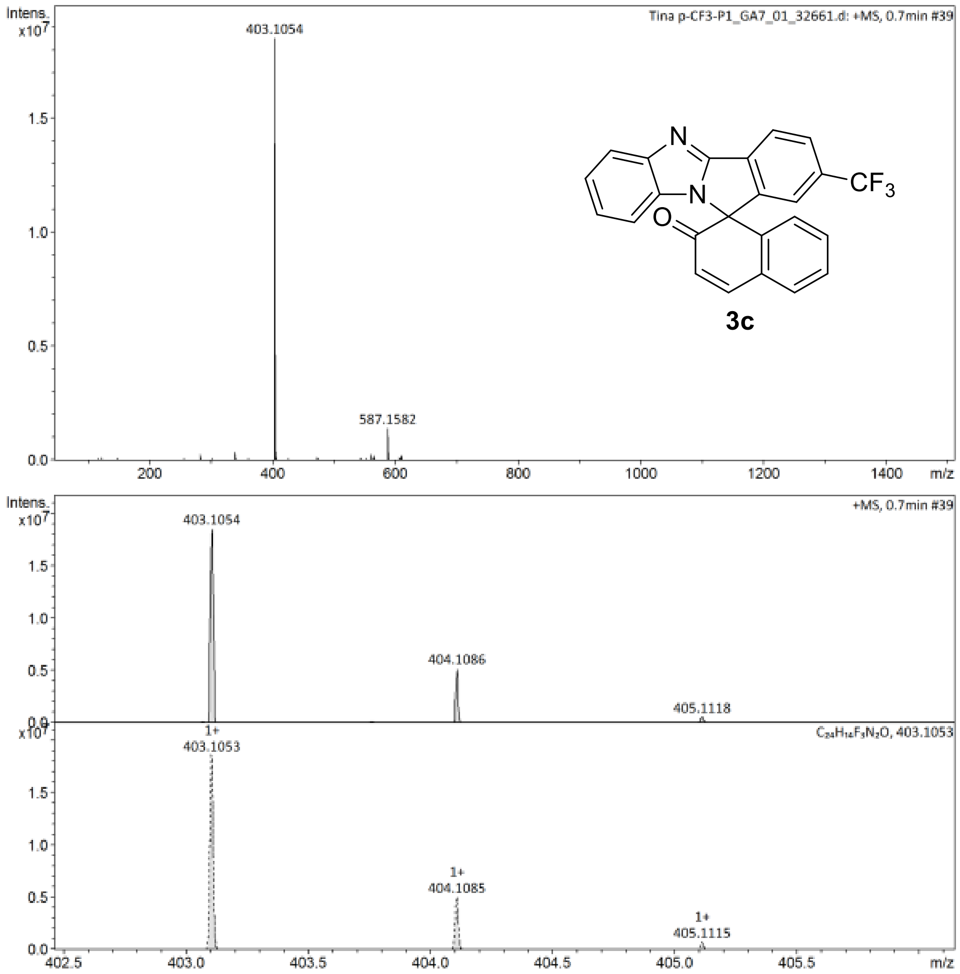

Display Report

Meas. m/z	#	Ion Formula	m/z	err [ppm]	mSigma	# Sigma	Score	rdb	e⁻ Conf	N-Rule	Adduct
403.1054	1	C24H14F3N2O	403.1053	-0.3	7.6	1	100.00	17.5	even	ok	M+H

HRMS (ESI) of compound **3c**

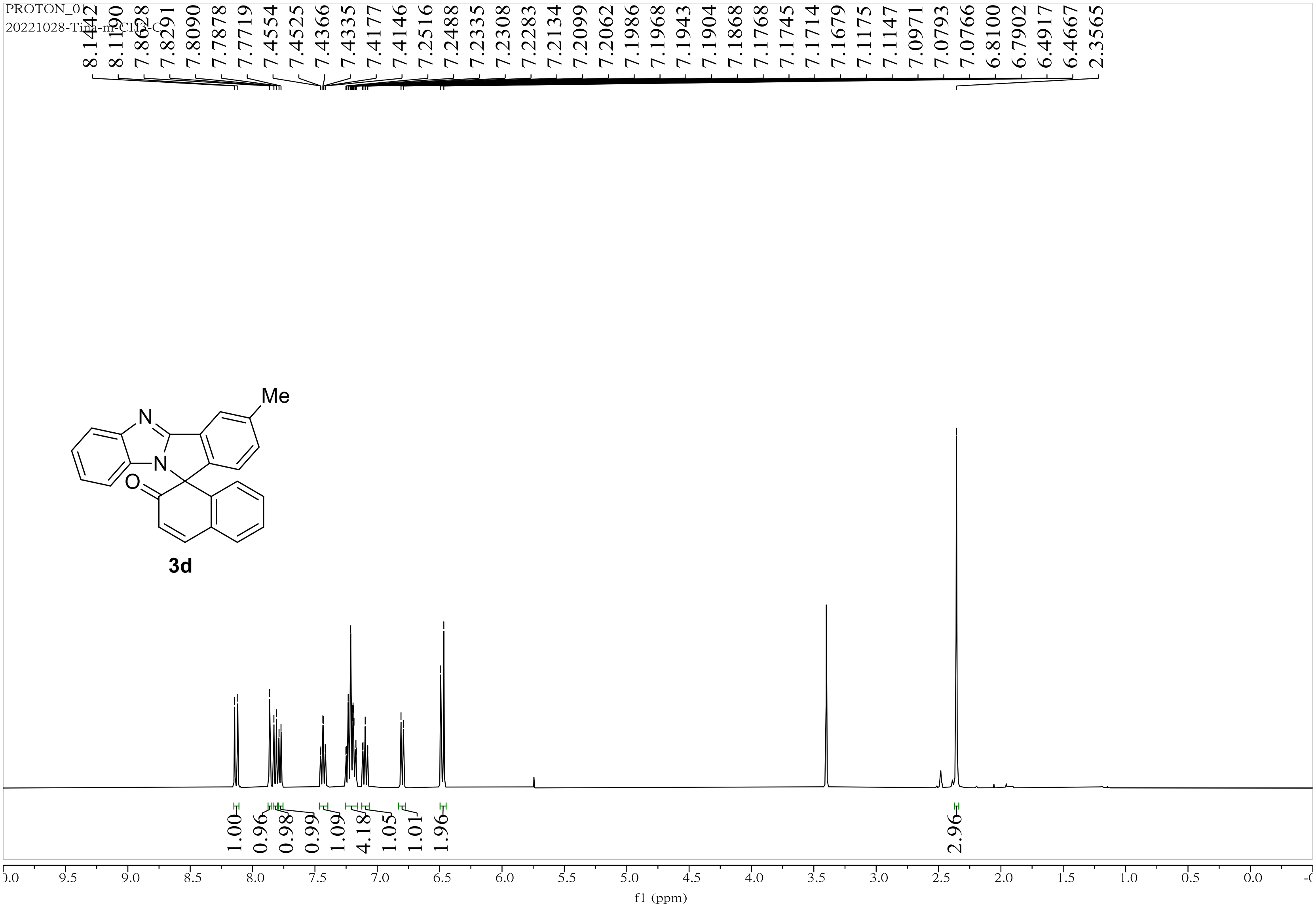

^{1}H NMR spectrum (400 MHz) of compound **3d** in DMSOd_6

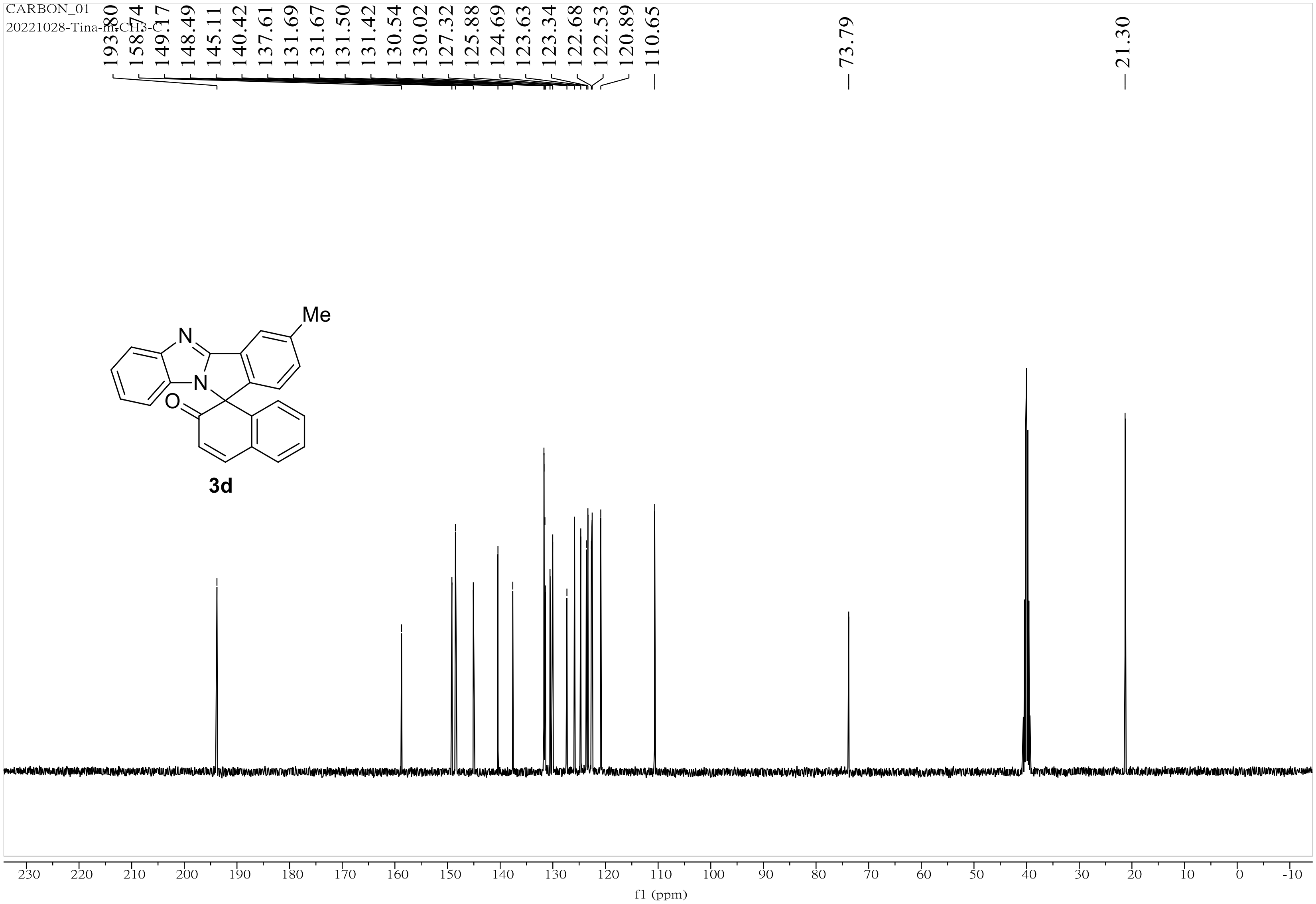

$^{13}C\{1H\}$ NMR spectrum (101 MHz) of compound **3d** in DMSO-d_6

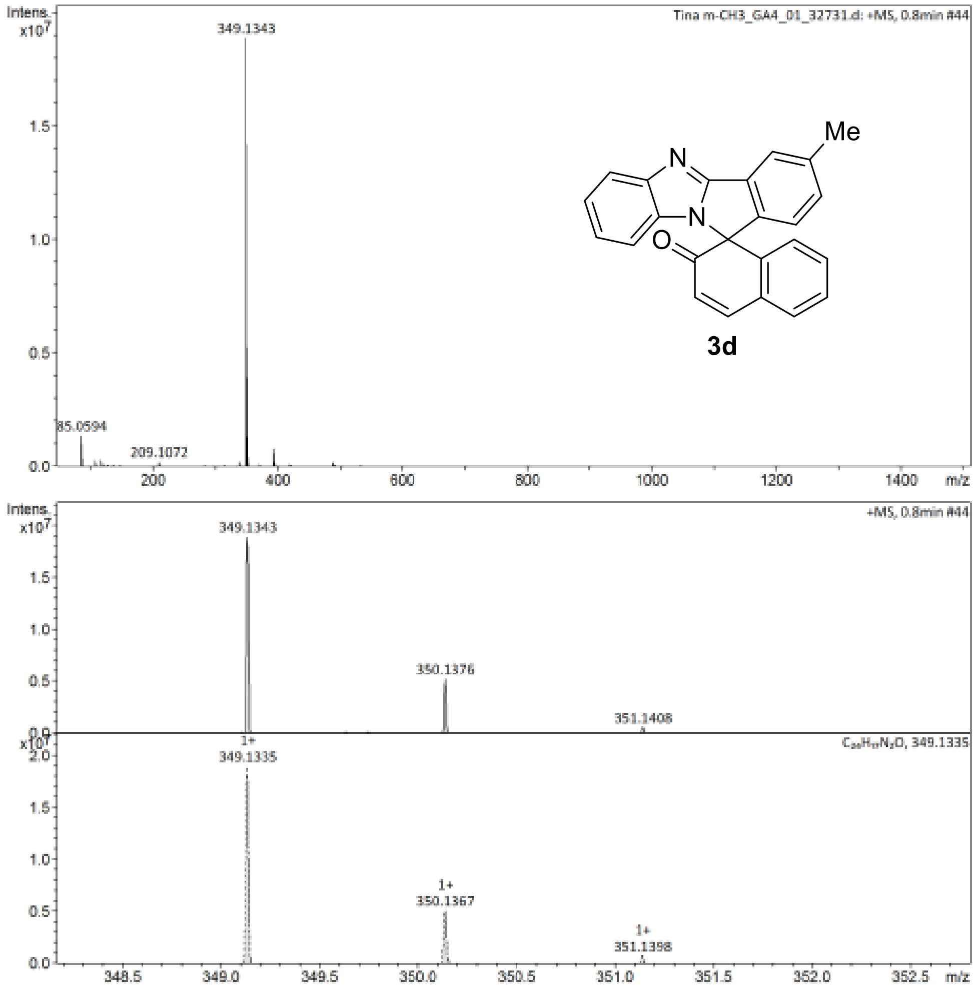

Display Report

Meas. m/z	#	Ion Formula	m/z	err [ppm]	mSigma	# Sigma	Score	rdb	e⁻ Conf	N-Rule	Adduct
349.1343	1	C24H17N2O	349.1335	2.3	6.3	1	100.00	17.5	even	ok	M+H

HRMS (ESI) of compound **3d**

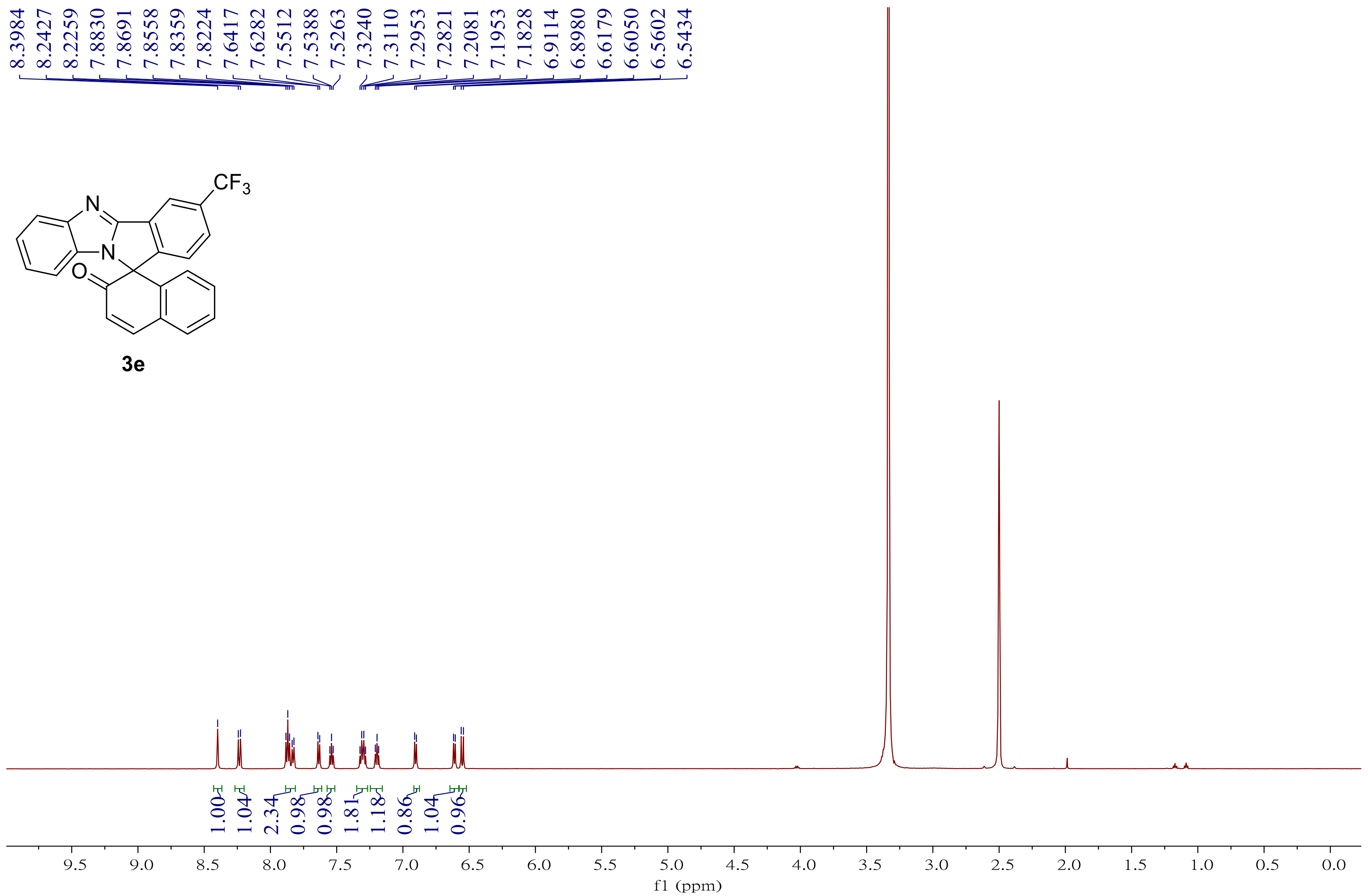

^{1}H NMR spectrum (600 MHz) of compound **3e** in DMSO-d_6

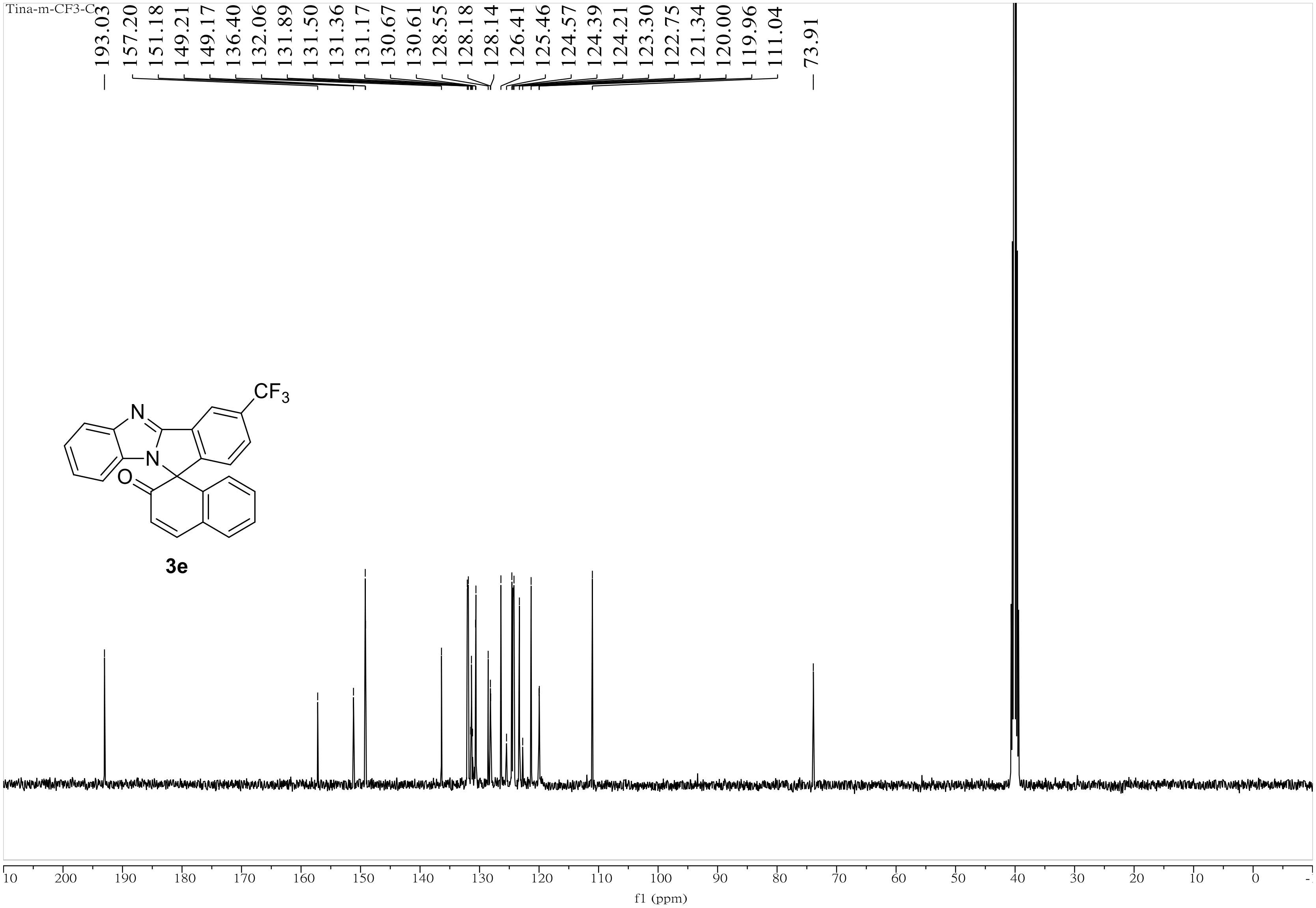

$^{13}C\{1H\}$ NMR spectrum (101 MHz) of compound **3e** in DMSO-d_6

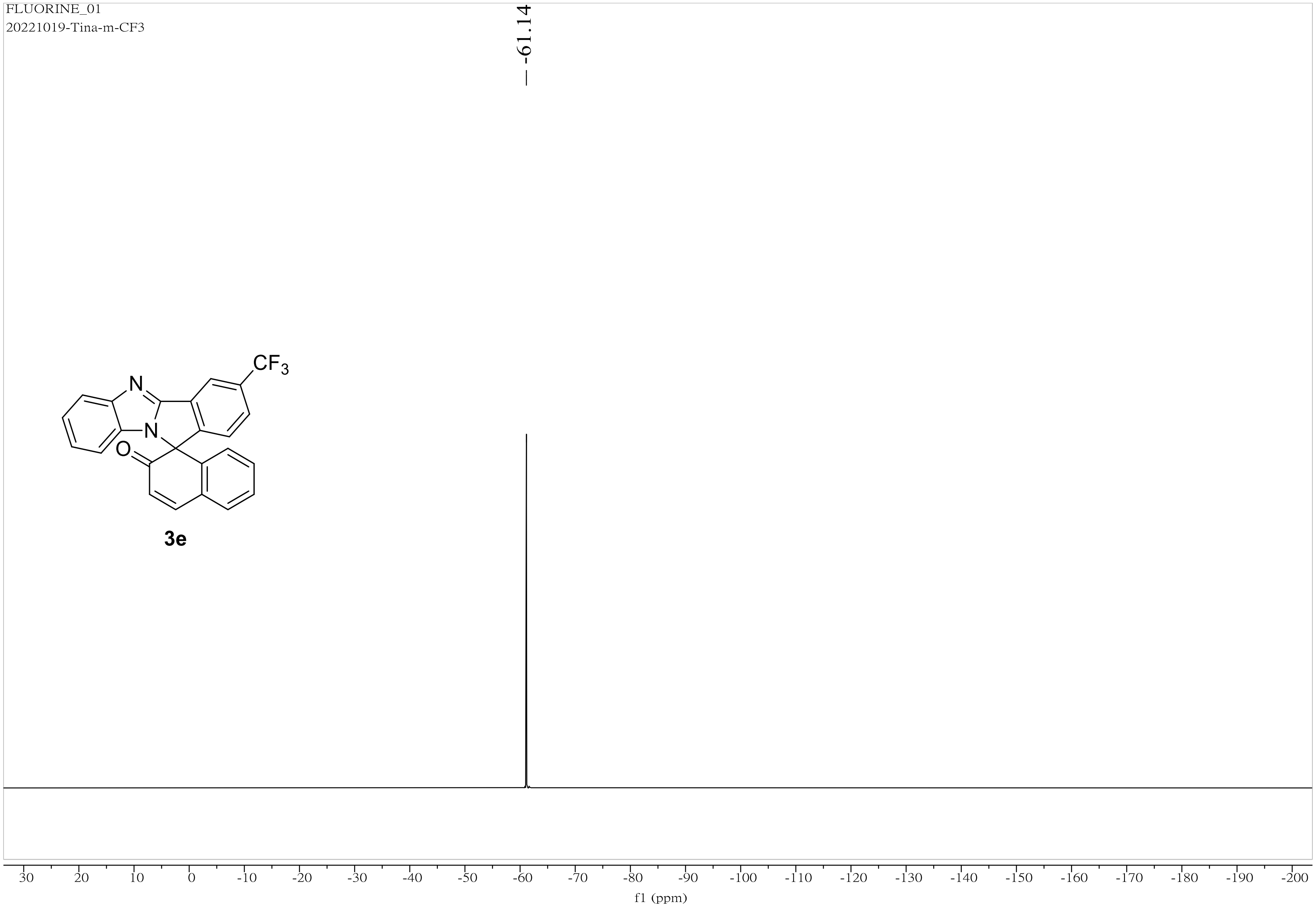

^{19}F NMR spectrum (376 MHz) of compound **3e** in DMSO-d_6

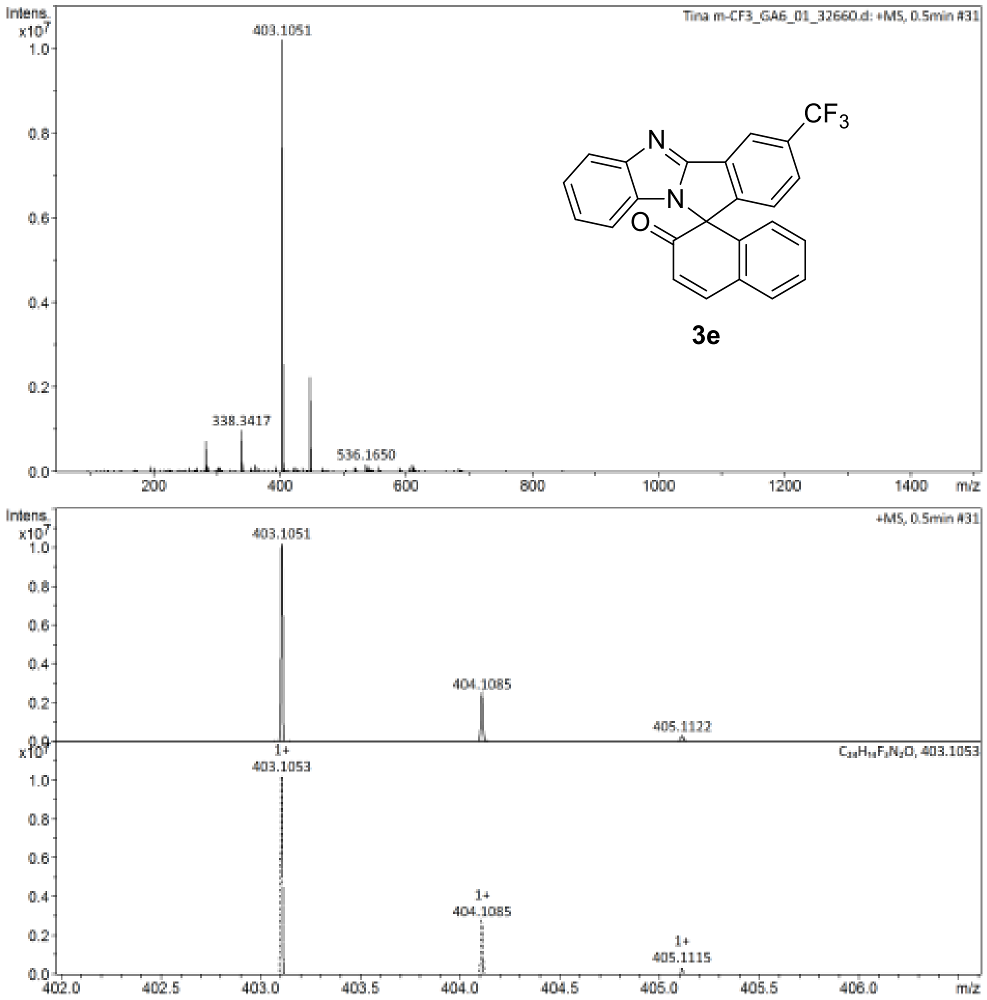

Display Report

Meas. m/z	#	Ion Formula	m/z	err [ppm]	mSigma	# Sigma	Score	rdb	e⁻ Conf	N-Rule	Adduct
403.1051	1	C24H14F3N2O	403.1053	0.5	16.9	1	100.00	17.5	even	ok	M+H

HRMS (ESI) of compound **3e**

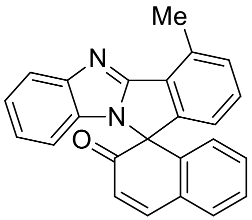

3f

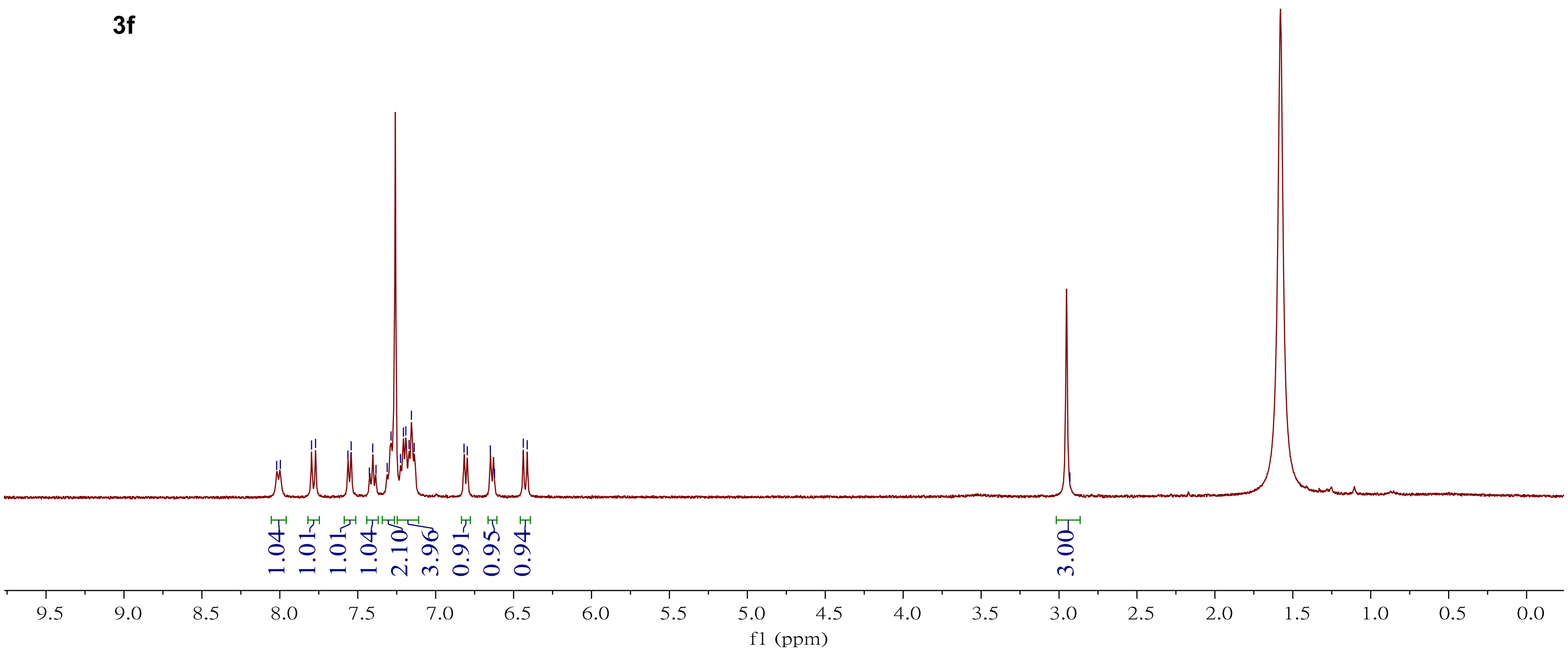

^{1}H NMR spectrum (400 MHz) of compound **3f** in $CDCl_3$

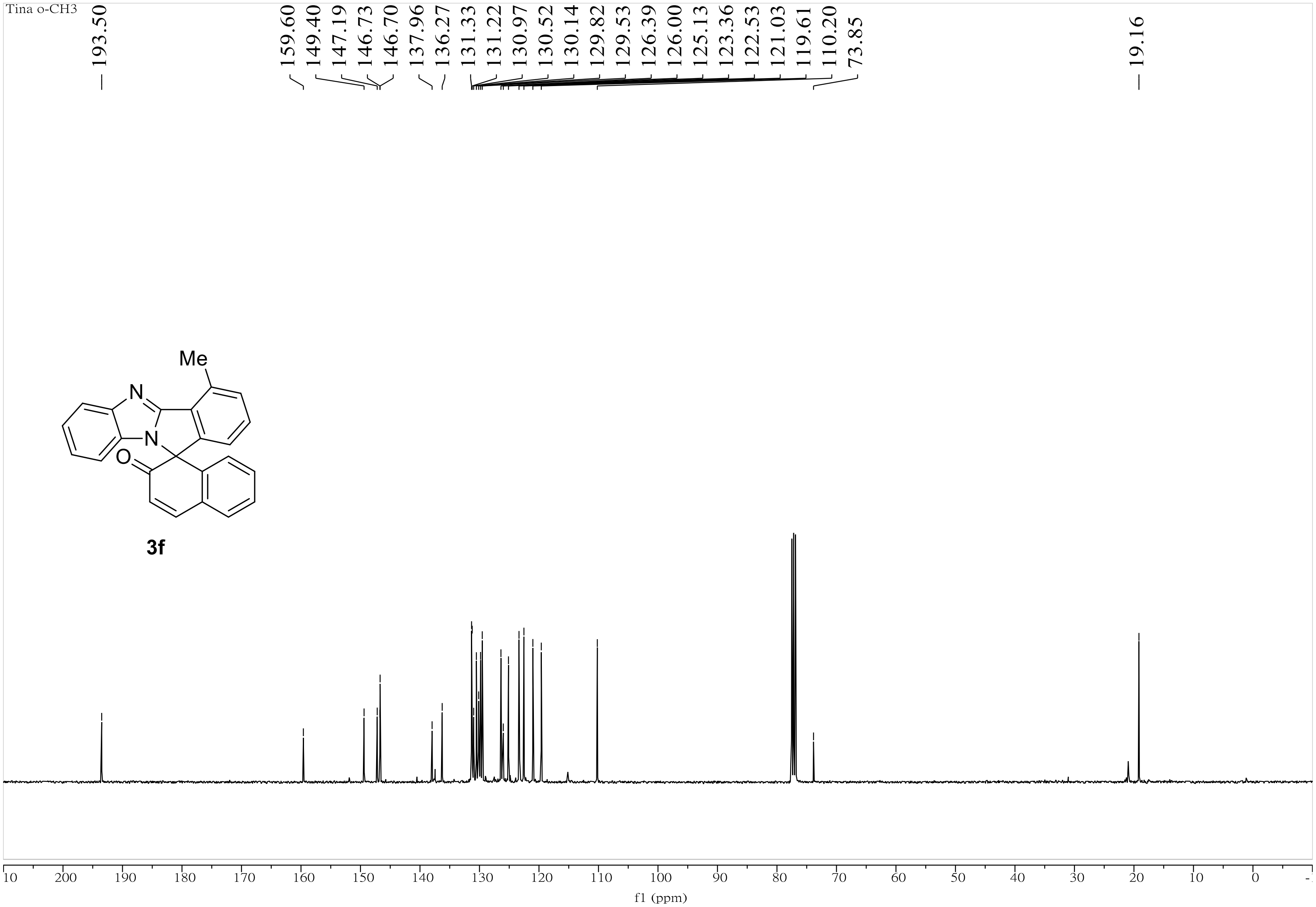

^{13}C{1H} NMR spectrum (101 MHz) of compound **3f** in $CDCl_3$

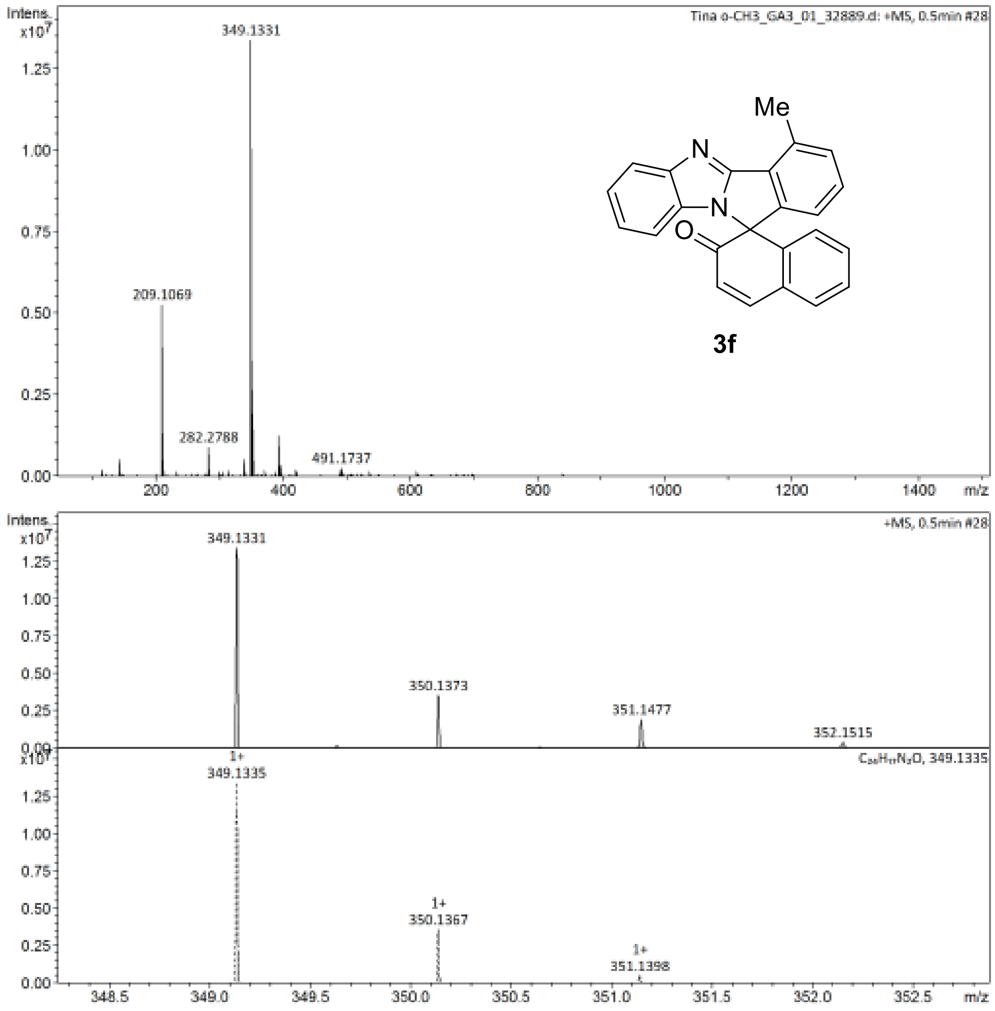

Display Report

Meas. m/z	#	Ion Formula	m/z	err [ppm]	mSigma	# Sigma	Score	rdb	e⁻ Conf	N-Rule	Adduct
349.1331	1	C24H17N2O	349.1335	1.1	21.5	1	100.00	17.5	even	ok	M+H

HRMS (ESI) of compound **3f**

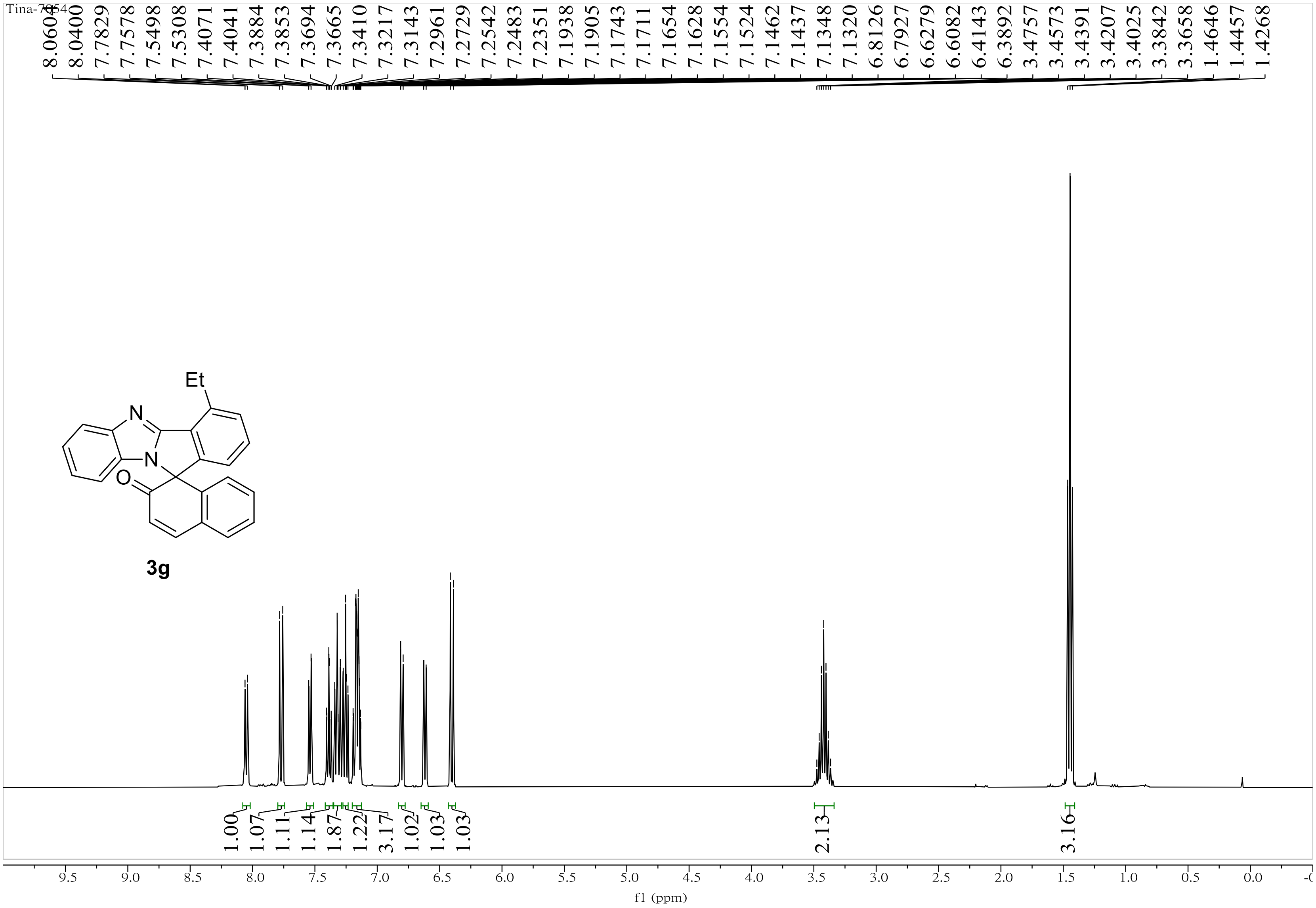

^{1}H NMR spectrum (400 MHz) of compound **3g** in $CDCl_3$.

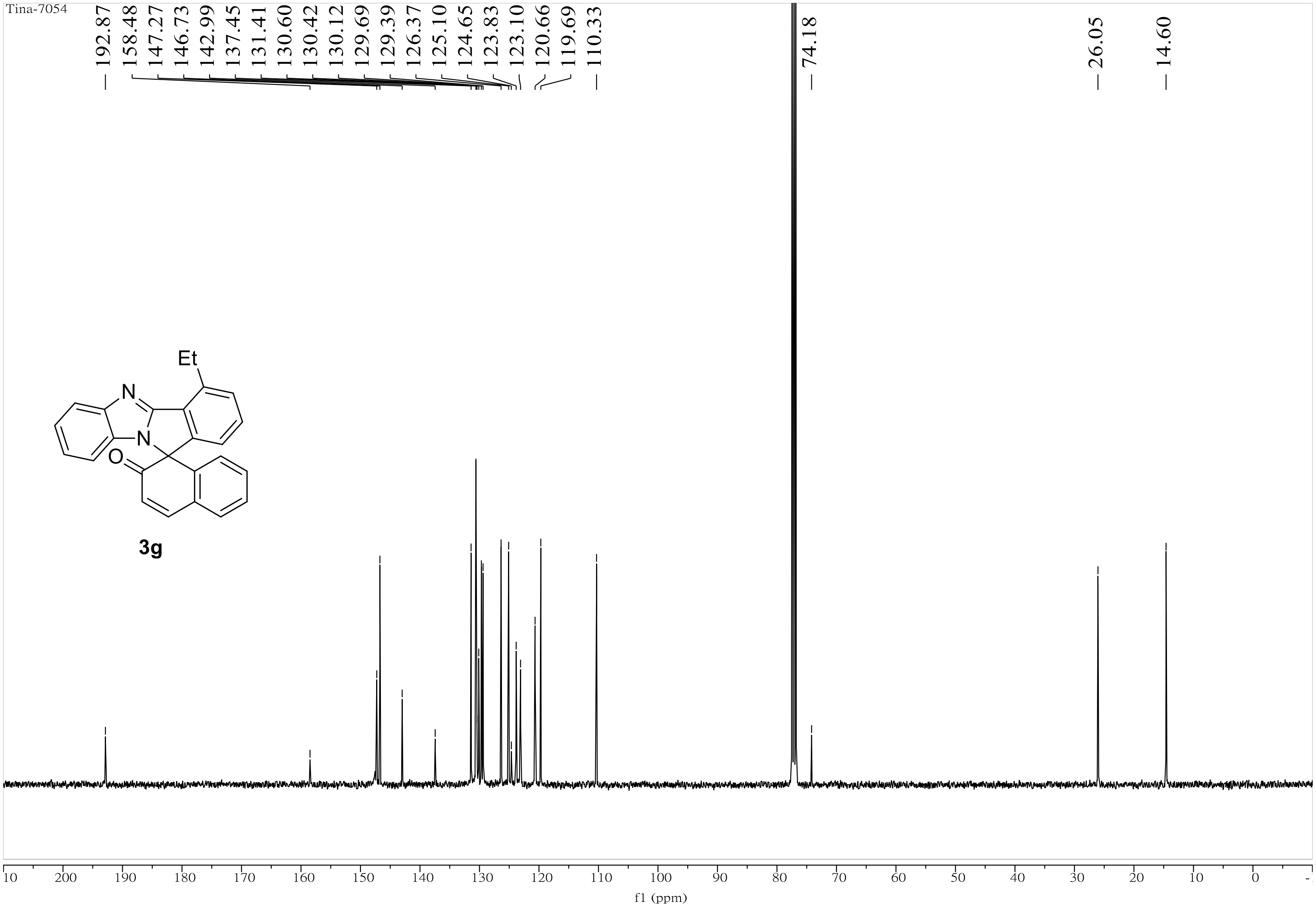

$^{13}C\{1H\}$ NMR spectrum (101 MHz) of compound **3g** in $CDCl_3$.

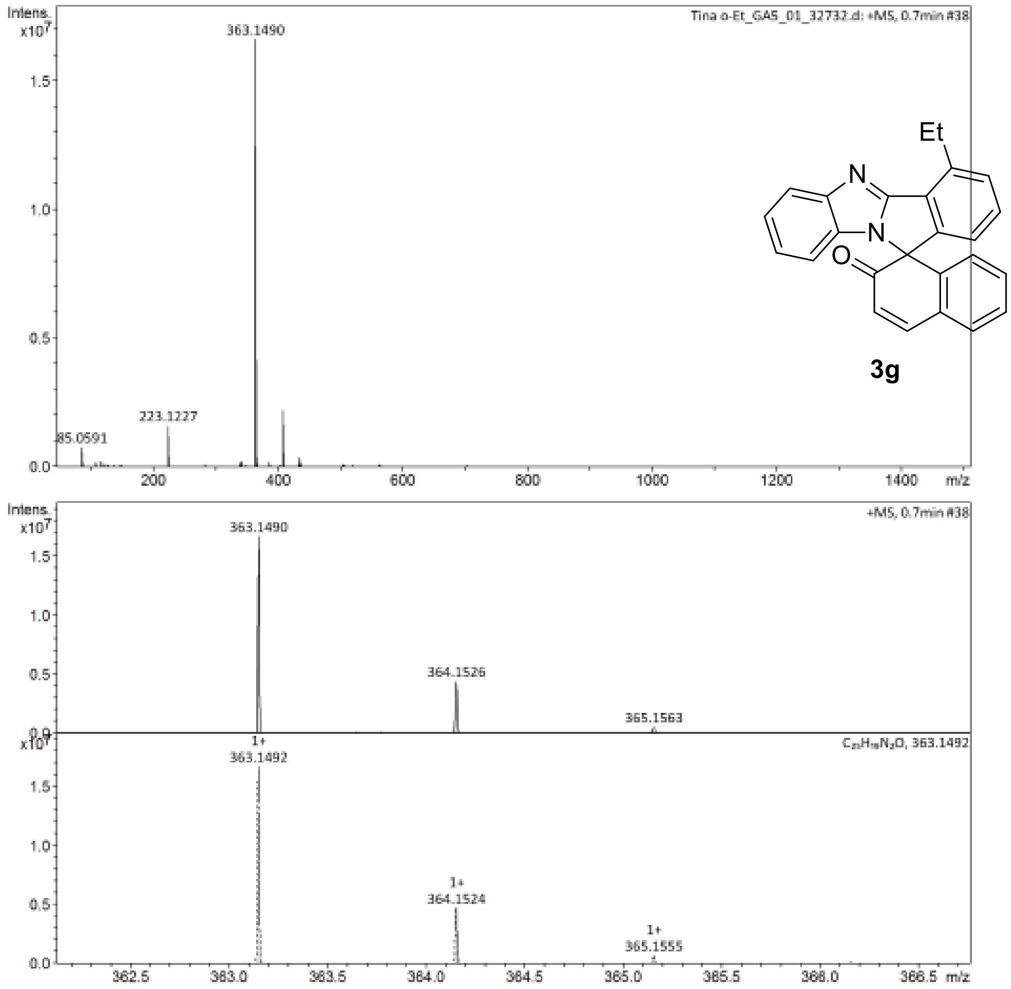

Display Report

Meas. m/z	#	Ion Formula	m/z	err [ppm]	mSigma	# Sigma	Score	rdb	e¯ Conf	N-Rule	Adduct
363.1490	1	C25H19N2O	363.1492	-0.4	9.3	1	100.00	17.5	even	ok	M+H

HRMS (ESI) of compound **3g**.

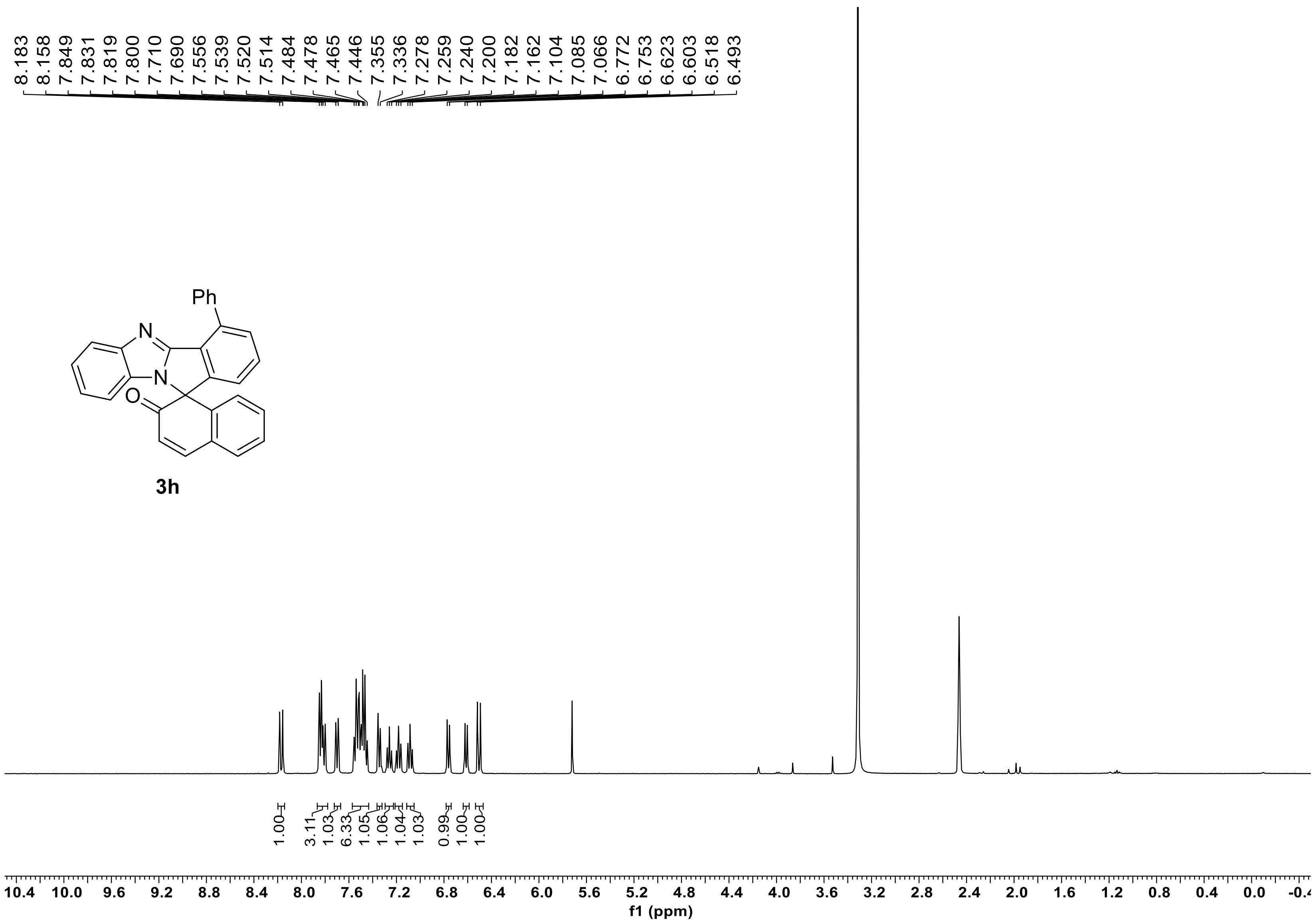

^{1}H NMR spectrum (400 MHz) of compound **3h** in DMSO-d_6.

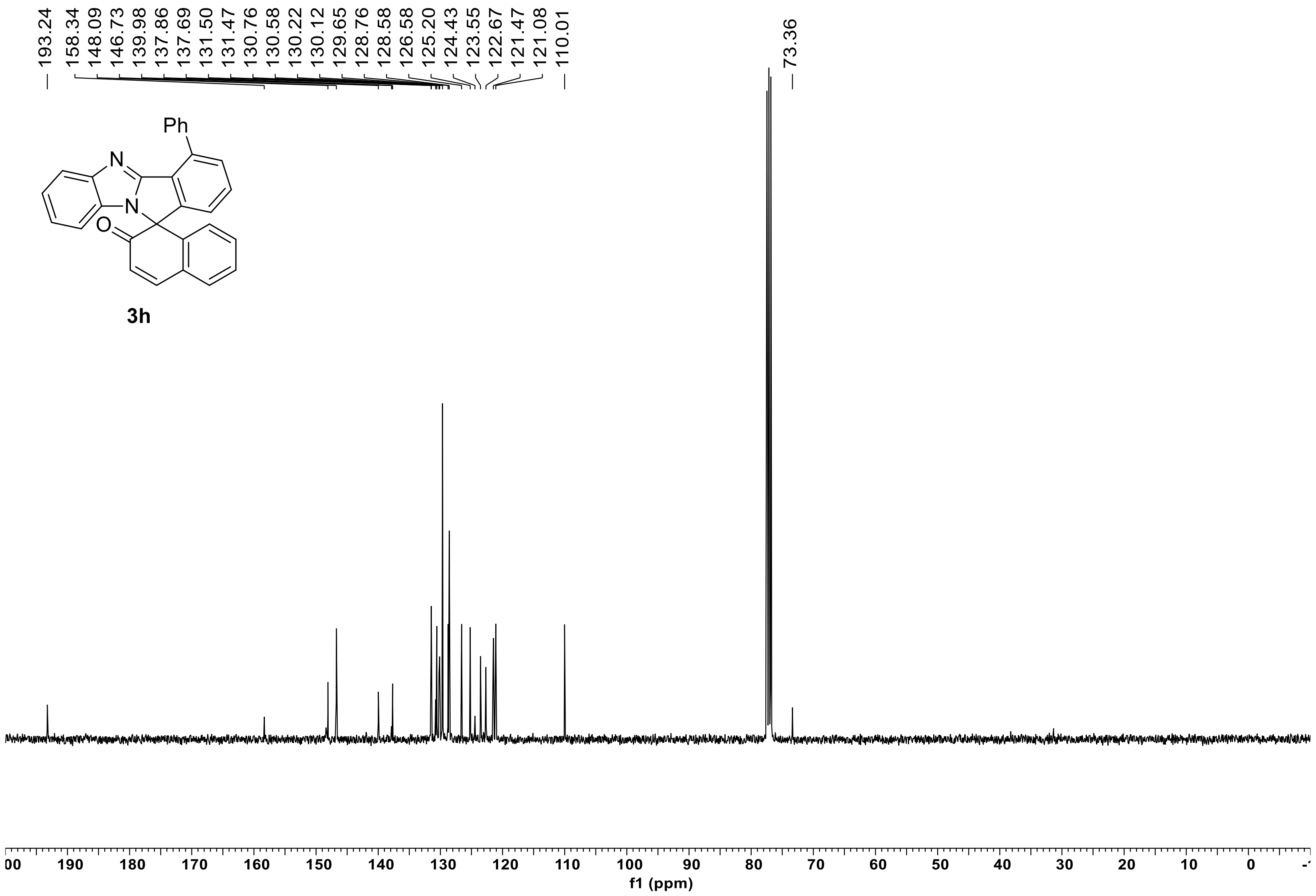

$^{13}C\{1H\}$ NMR spectrum (101 MHz) of compound **3h** in DMSO-d_6.

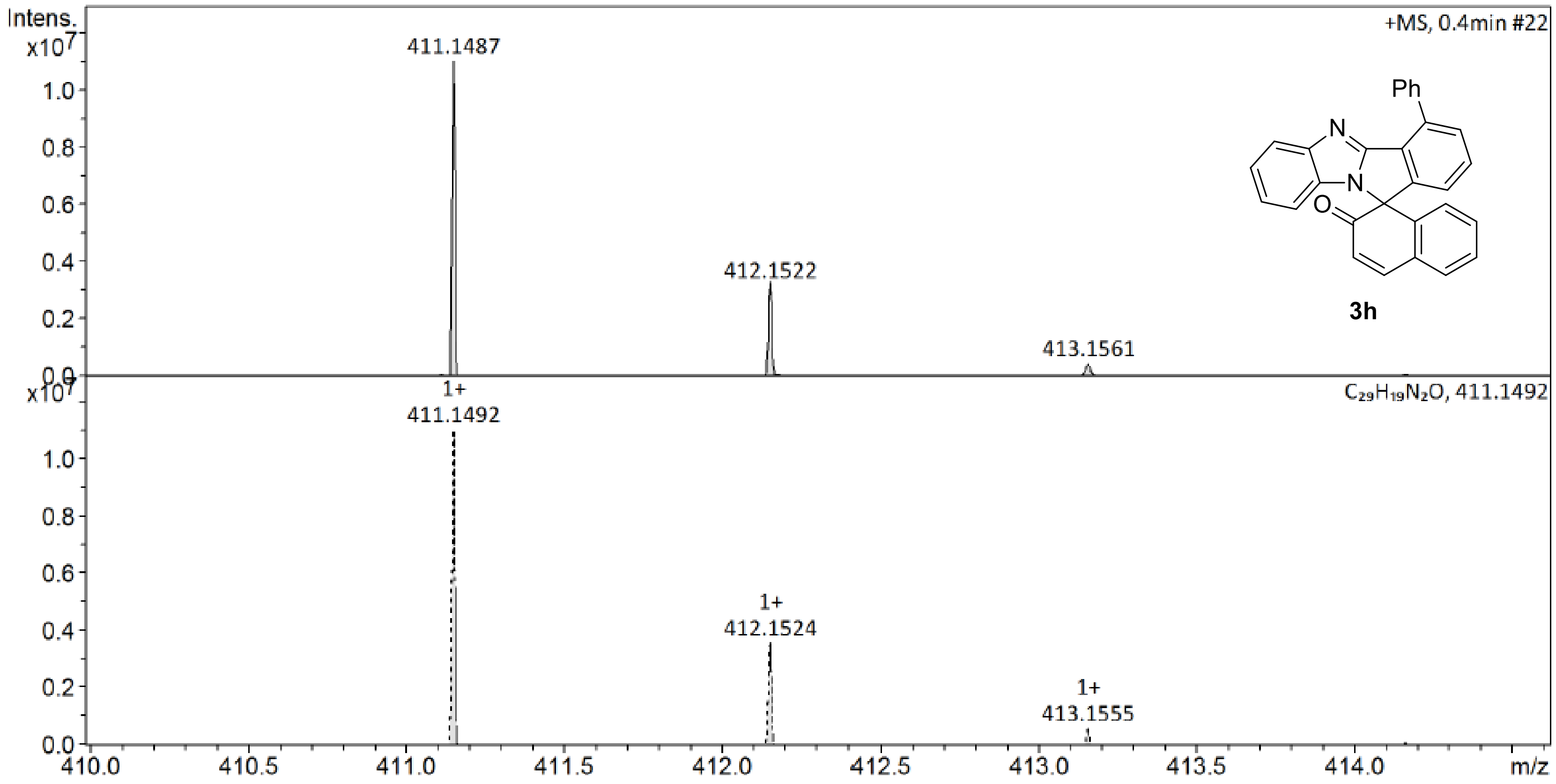

Display Report

Meas. m/z	#	Ion Formula	m/z	err [ppm]	mSigma	# Sigma	Score	rdb	e⁻ Conf	N-Rule	Adduct
411.1487	1	C29H19N2O	411.1492	1.3	18.9	1	100.00	21.5	even	ok	M+H

HRMS (ESI) of compound **3h**.

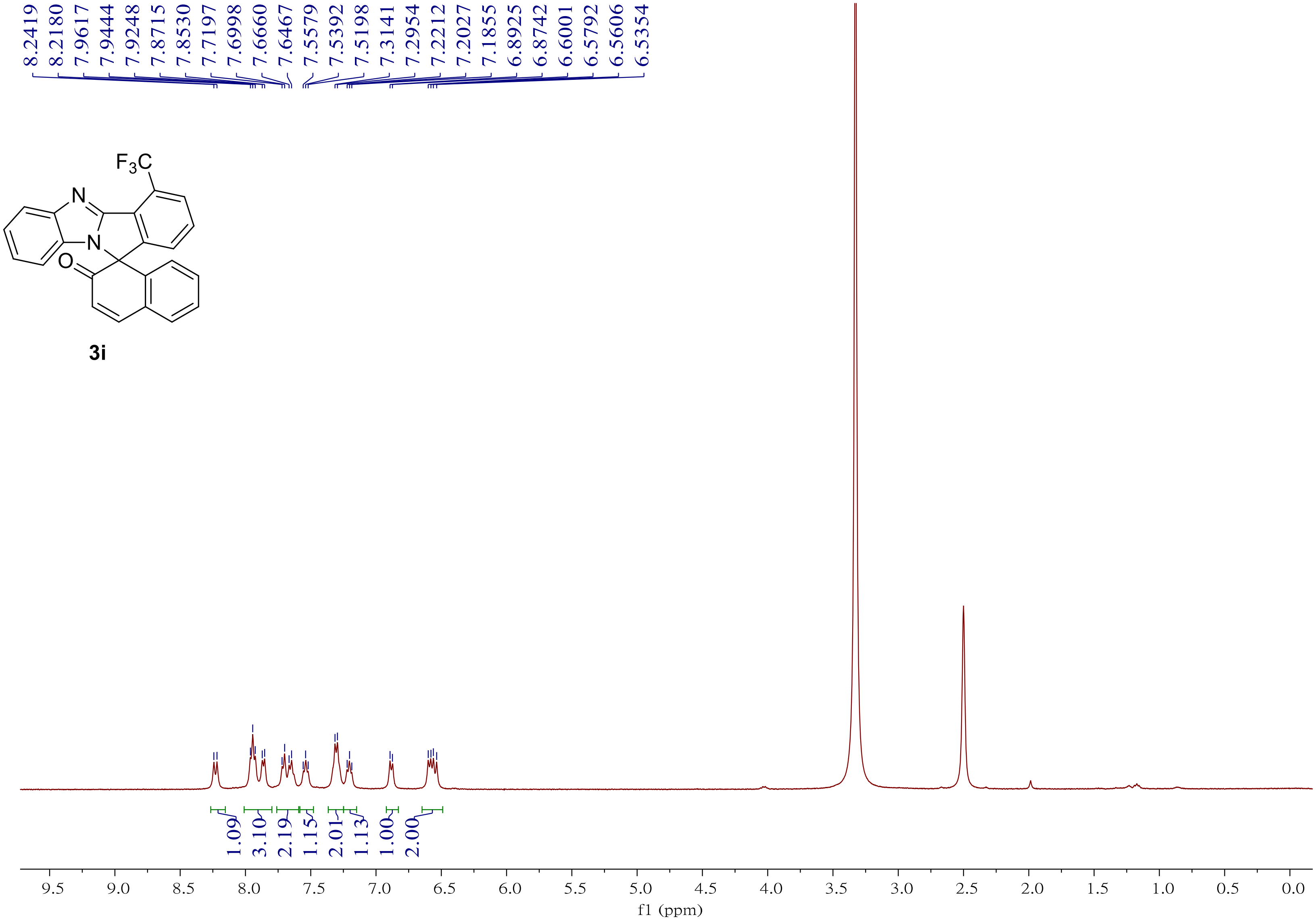

^{1}H NMR spectrum (400 MHz) of compound **3i** in DMSO-d_6.

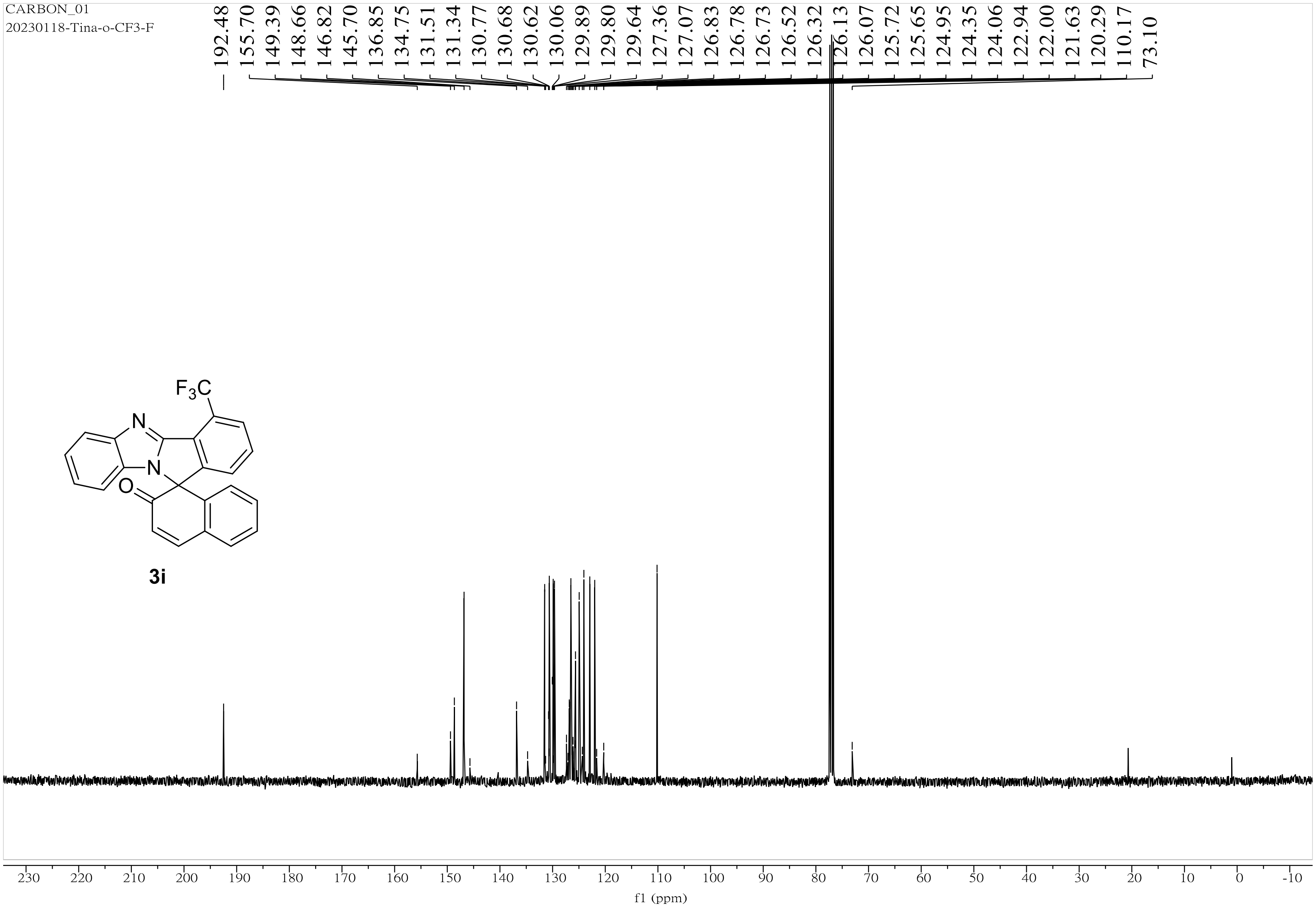

^{13}C{1H} NMR spectrum (101 MHz) of compound **3i** in DMSO-d_6.

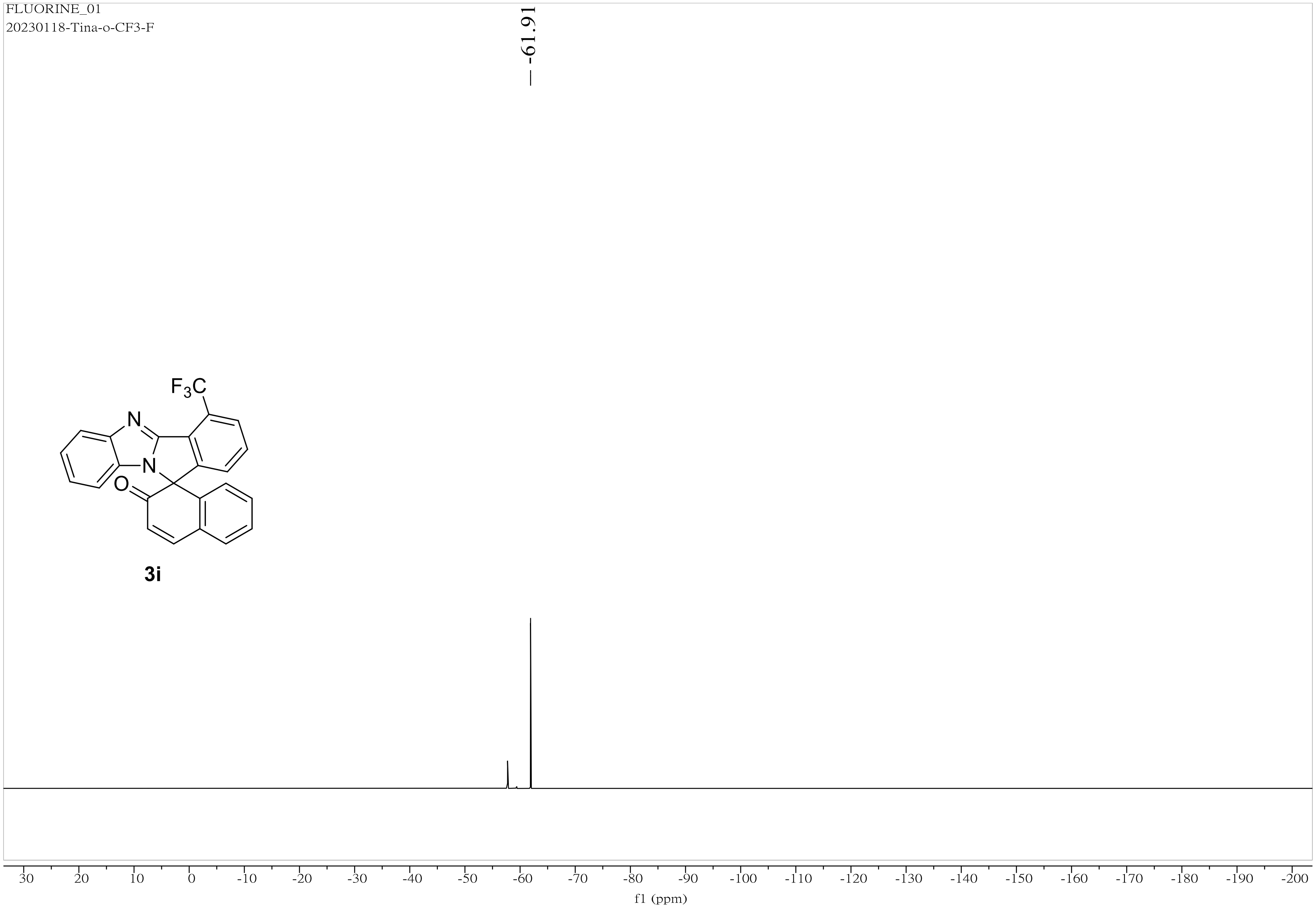

^{19}F NMR spectrum (376 MHz) of compound **3i** in DMSO-d_6.

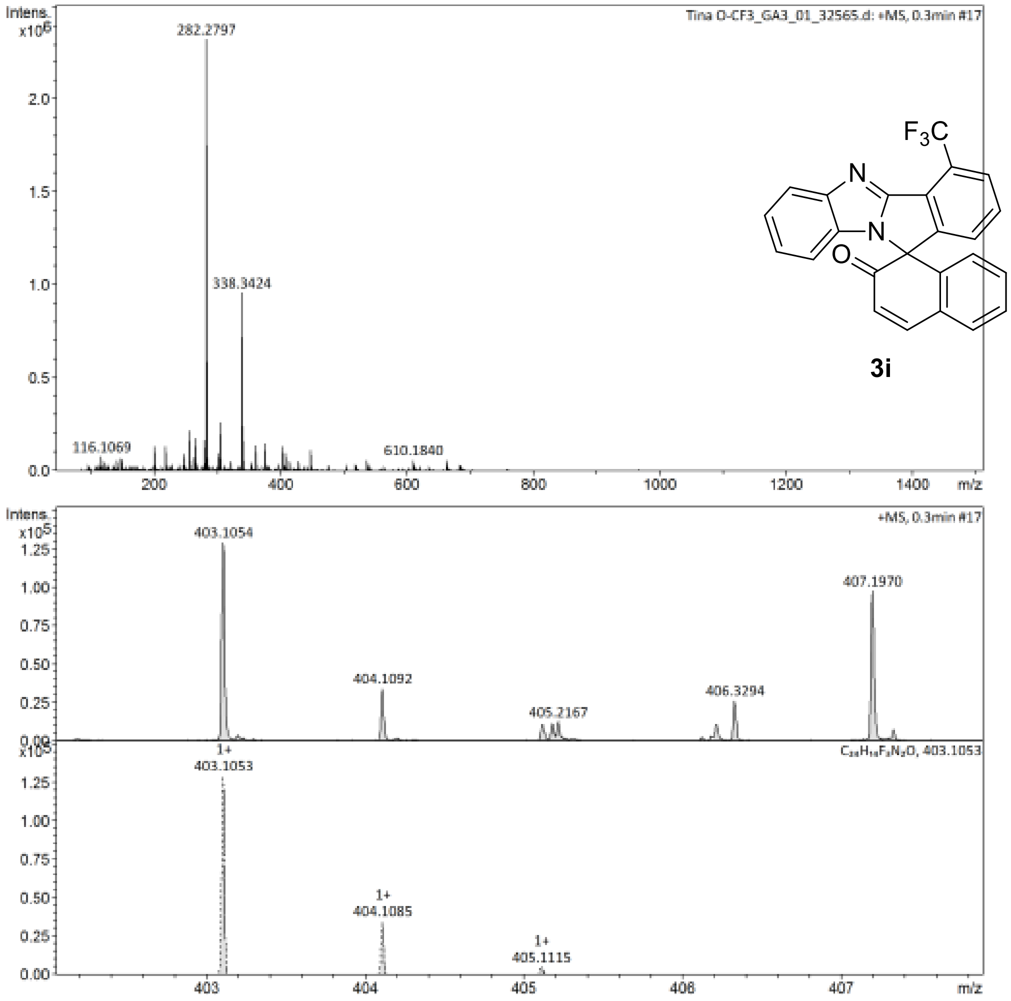

Display Report

Meas. m/z	#	Ion Formula	m/z	err [ppm]	mSigma	# Sigma	Score	rdb	e⁻ Conf	N-Rule	Adduct
403.1054	1	C24H14F3N2O	403.1053	-0.3	26.2	1	100.00	17.5	even	ok	M+H

HRMS (ESI) of compound **3i**.

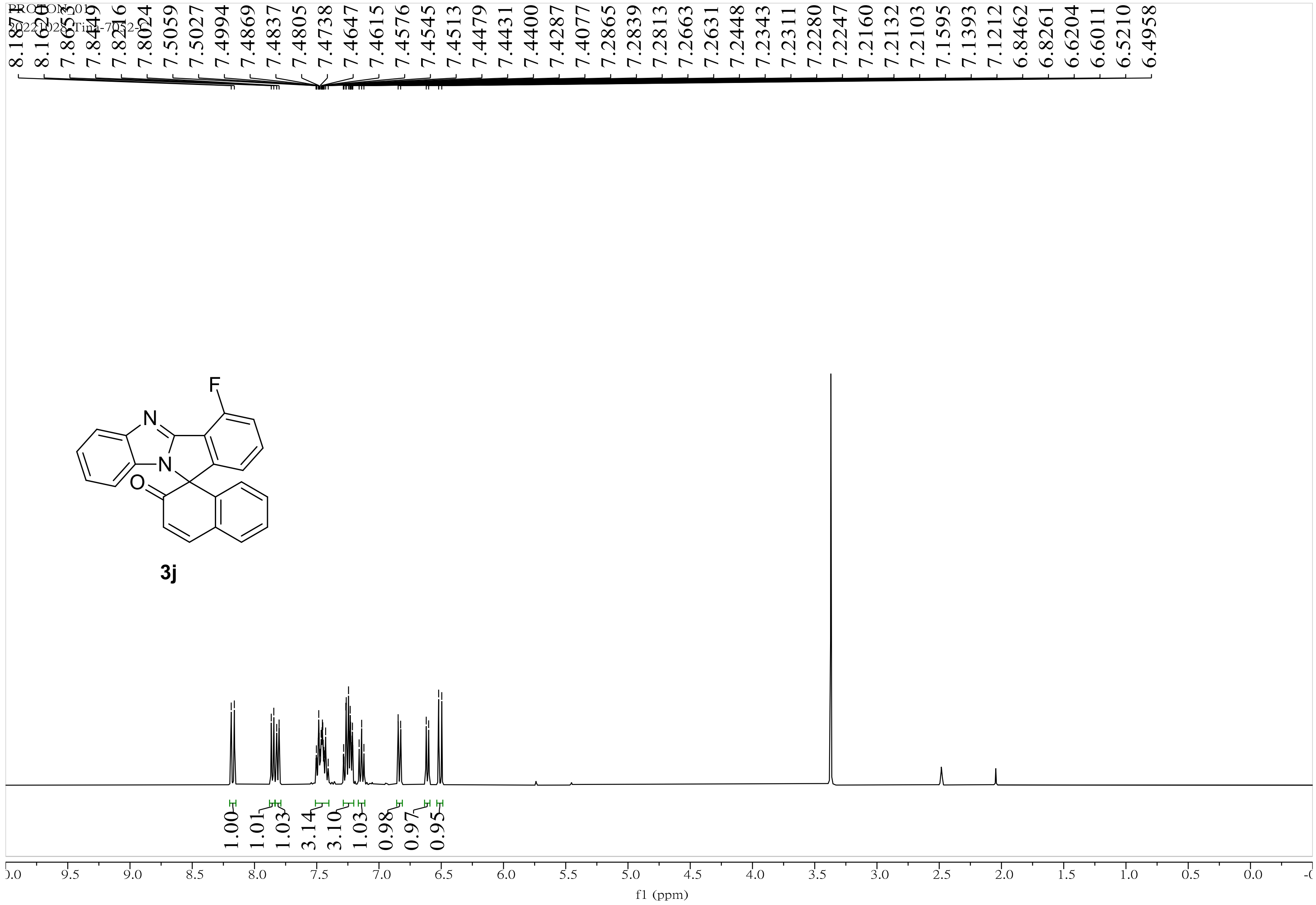

^{1}H NMR spectrum (400 MHz) of compound **3j** in DMSO-d_6.

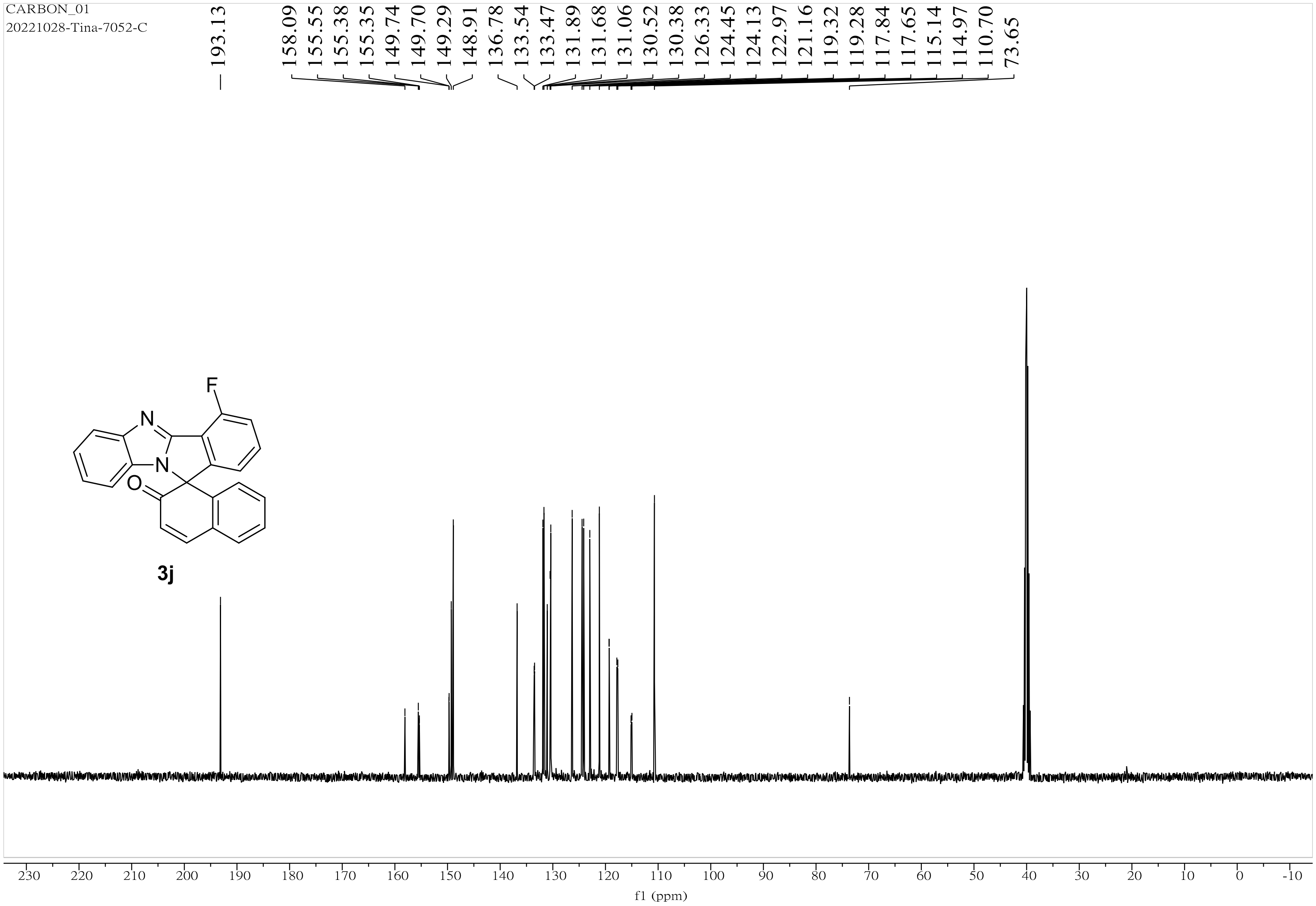

$^{13}C\{1H\}$ NMR spectrum (101 MHz) of compound **3j** in DMSO-d_6.

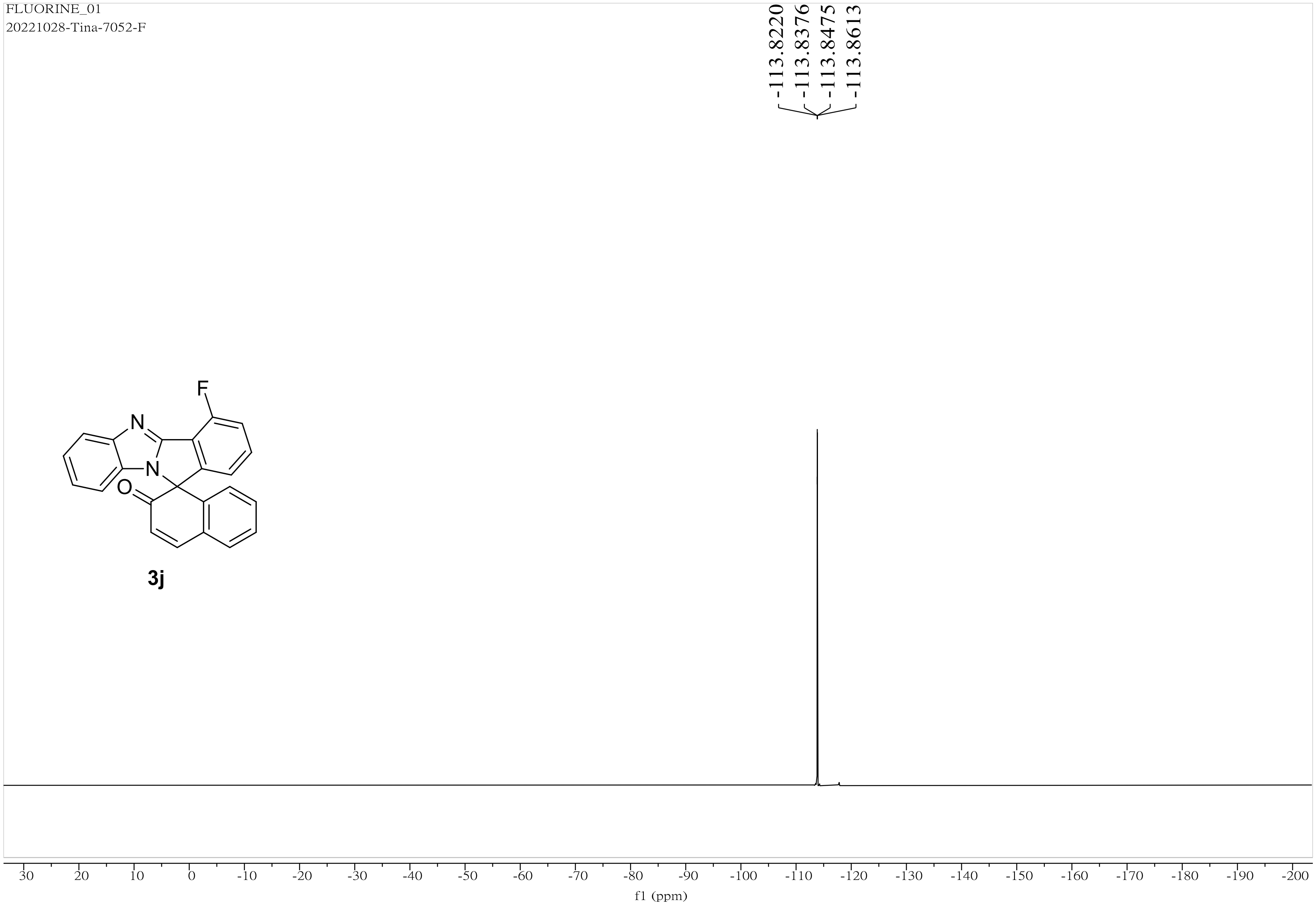

^{19}F NMR spectrum (376 MHz) of compound **3j** in DMSO-d_6

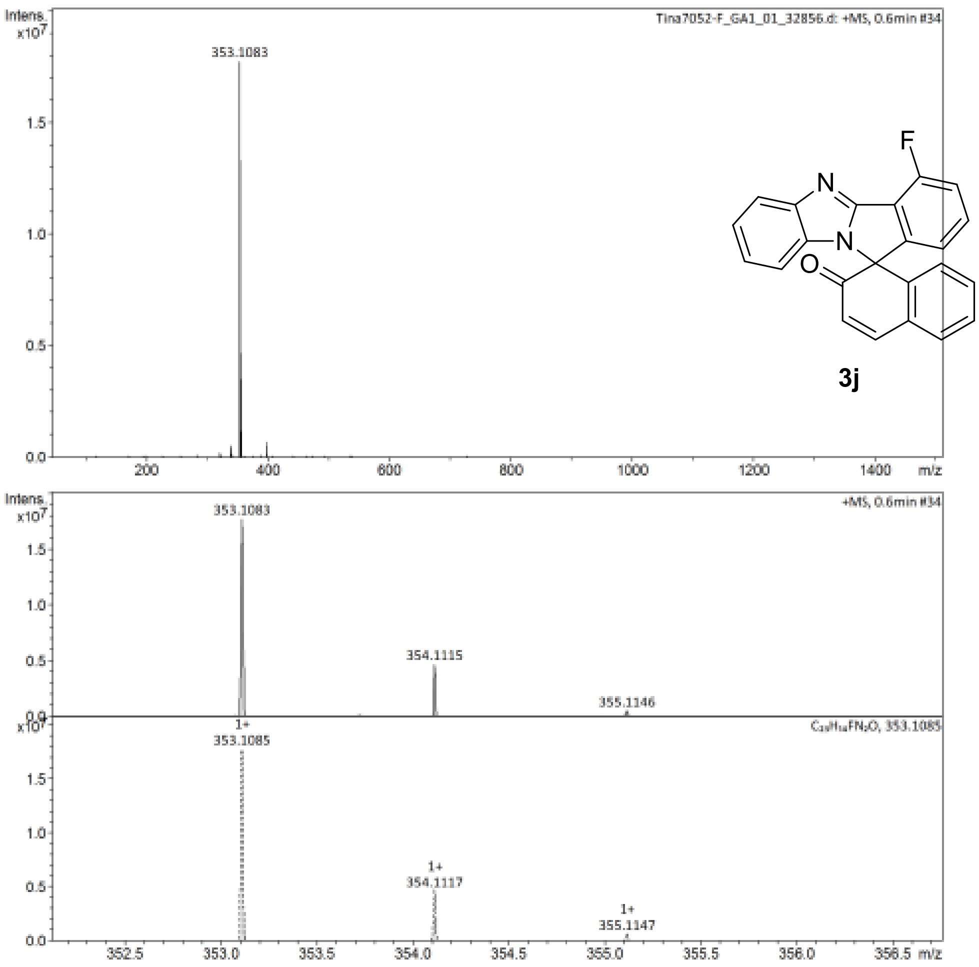

Display Report

Meas. m/z	#	Ion Formula	m/z	err [ppm]	mSigma	# Sigma	Score	rdb	e⁻ Conf	N-Rule	Adduct
353.1083	1	C23H14FN2O	353.1085	0.5	4.8	1	100.00	17.5	even	ok	M+H

HRMS (ESI) of compound **3j**.

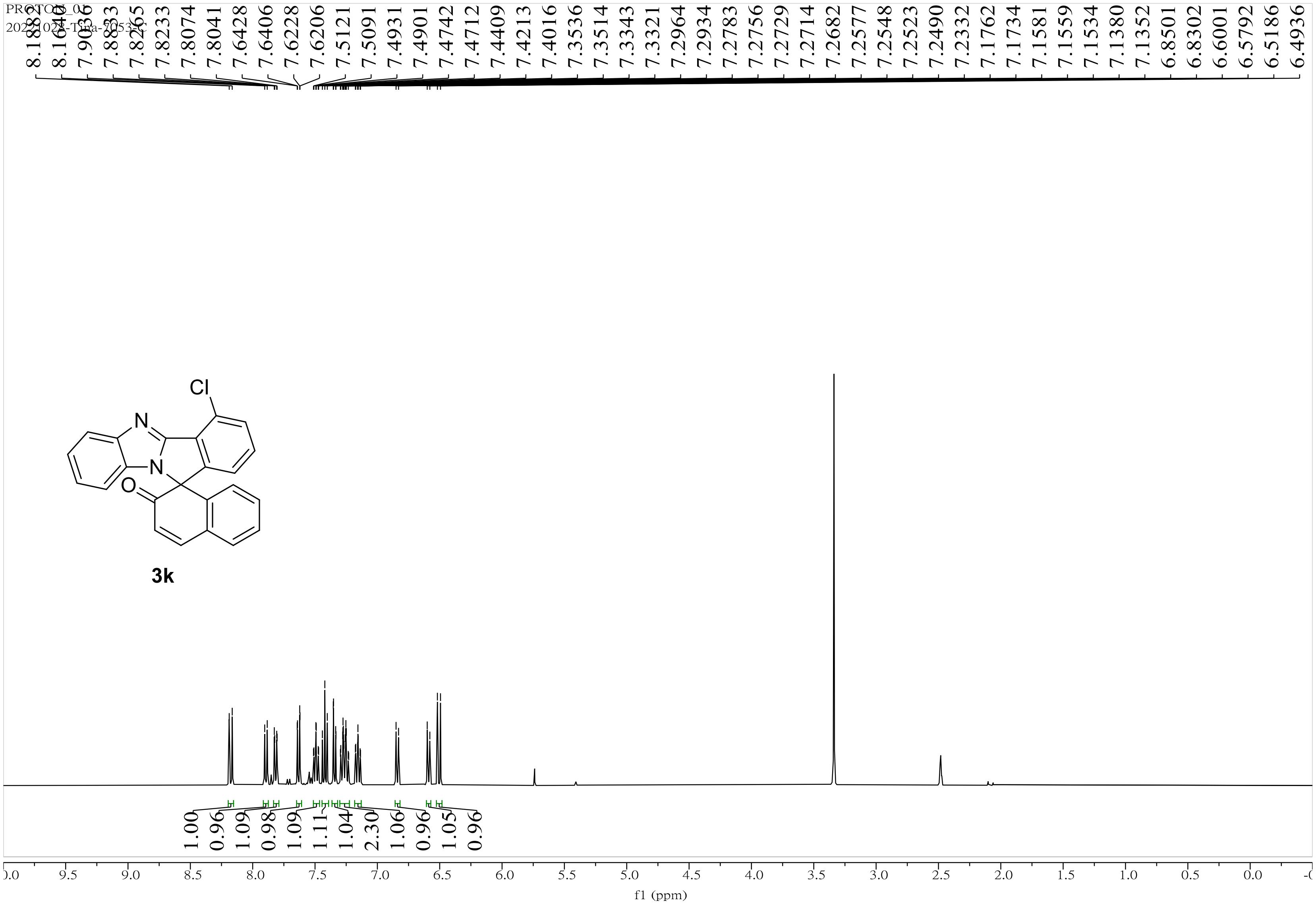

^{1}H NMR spectrum (400 MHz) of compound **3k** in DMSO-d_6.

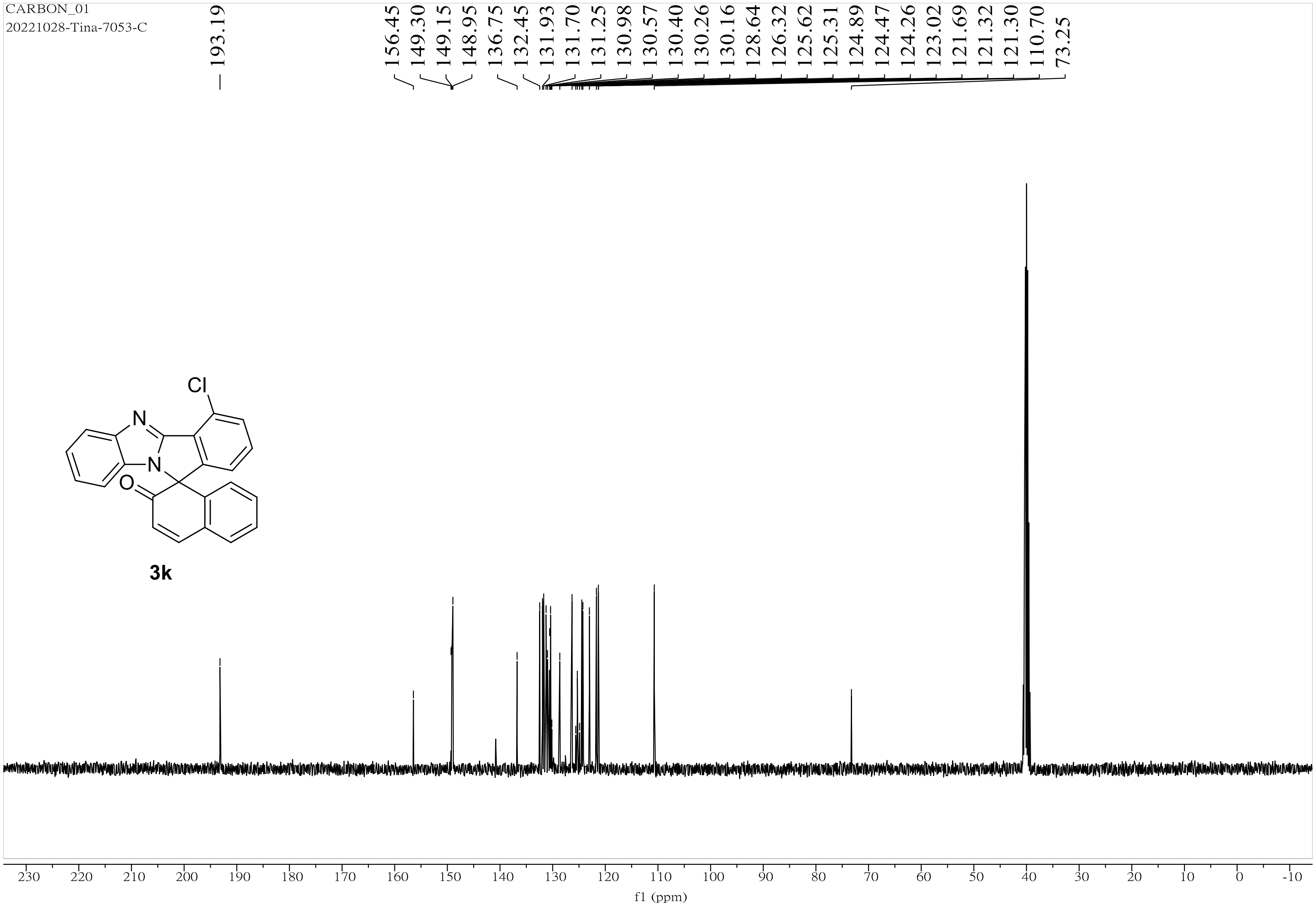

$^{13}C\{1H\}$ NMR spectrum (101 MHz) of compound **3k** in DMSO-d_6.

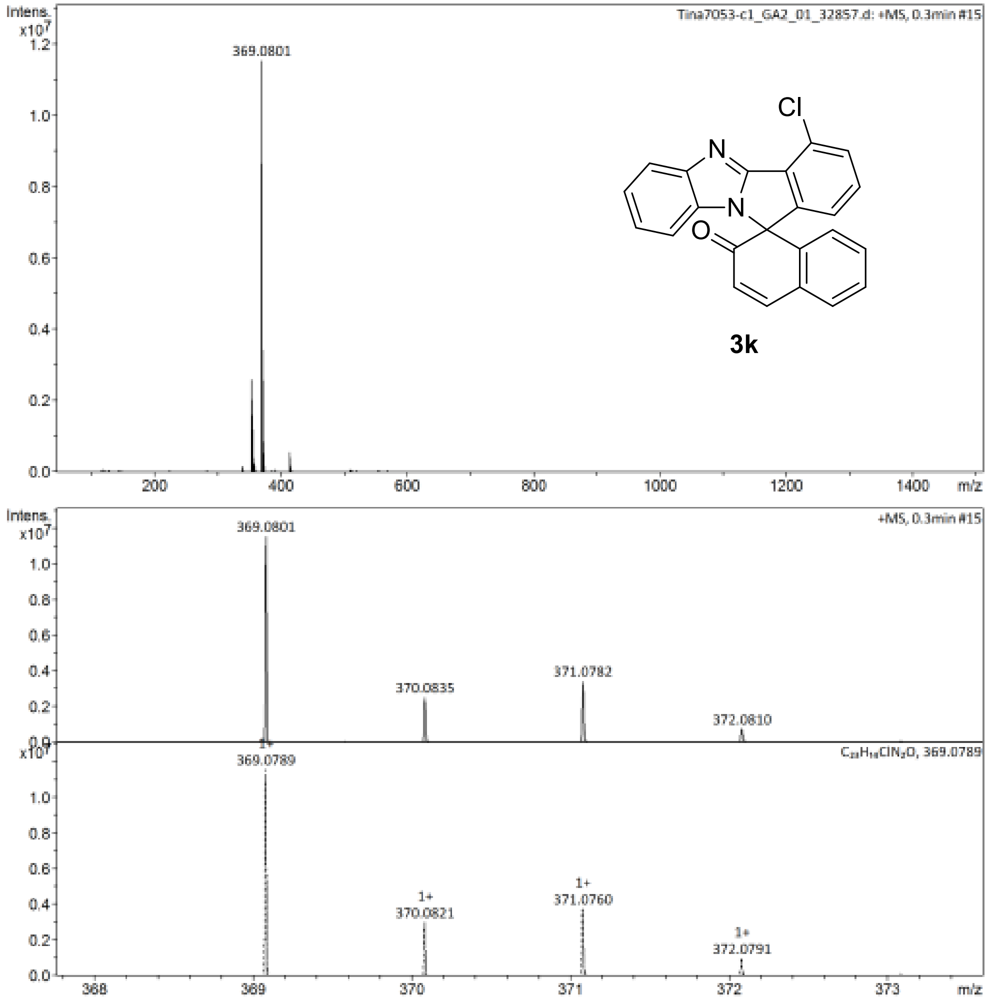

Display Report

Meas. m/z	#	Ion Formula	m/z	err [ppm]	mSigma	# Sigma	Score	rdb	e⁻ Conf	N-Rule	Adduct
369.0801	1	C23H14ClN2O	369.0789	3.1	32.9	1	100.00	17.5	even	ok	M+H

HRMS (ESI) of compound **3k**.

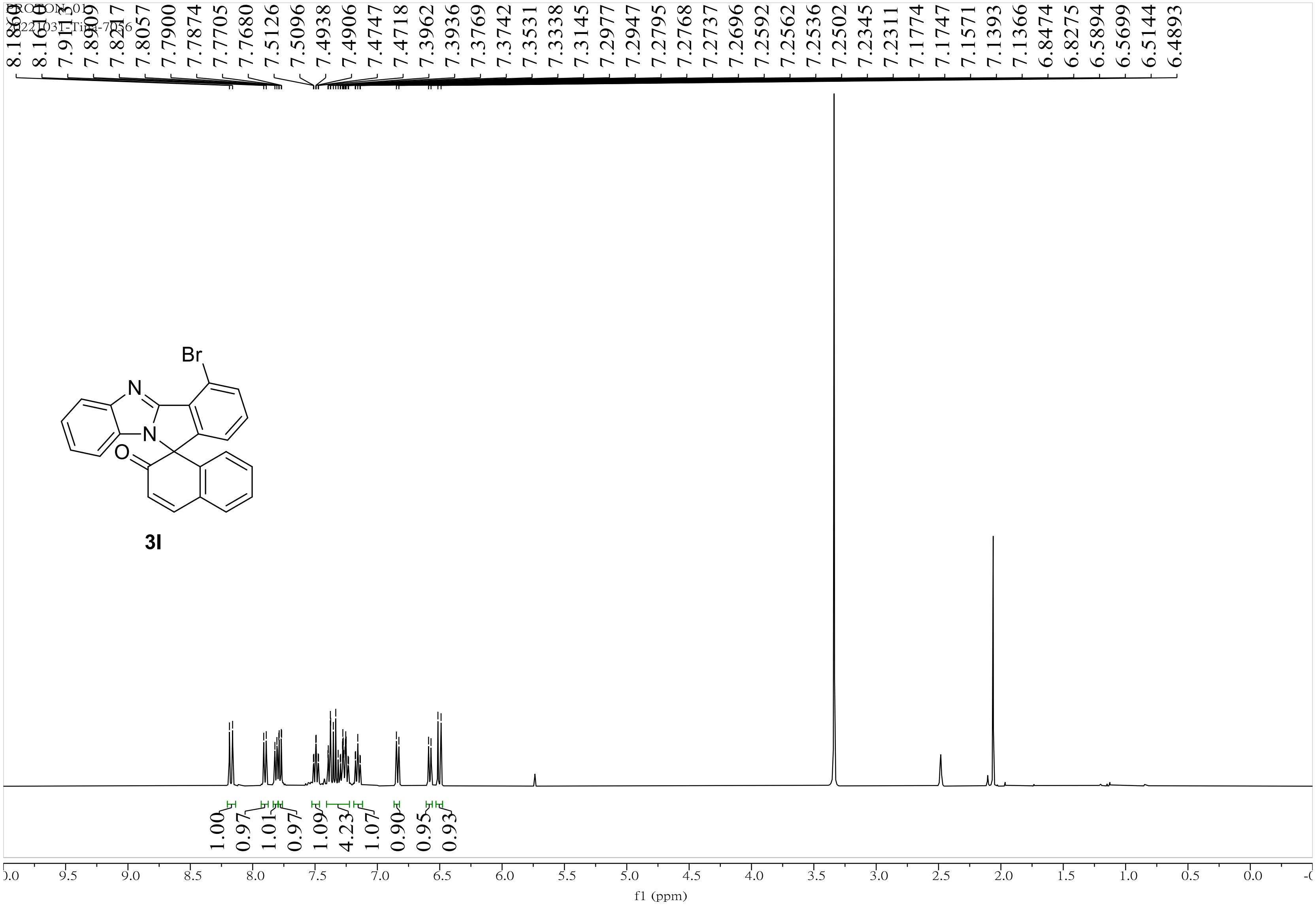

^{1}H NMR spectrum (400 MHz) of compound **3l** in DMSO-d_6.

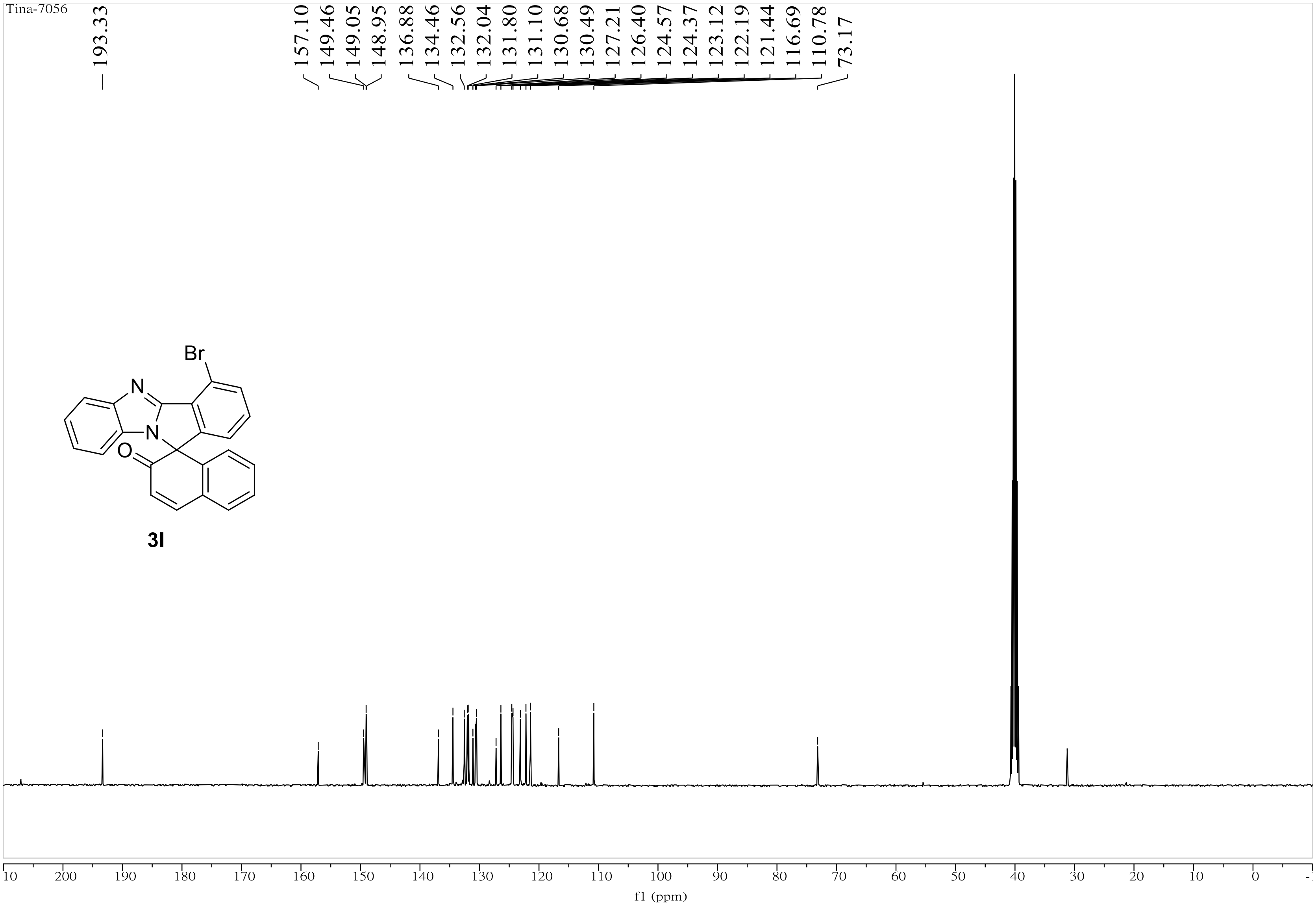

^{13}C{1H} NMR spectrum (101 MHz) of compound **3l** in DMSO-d_6.

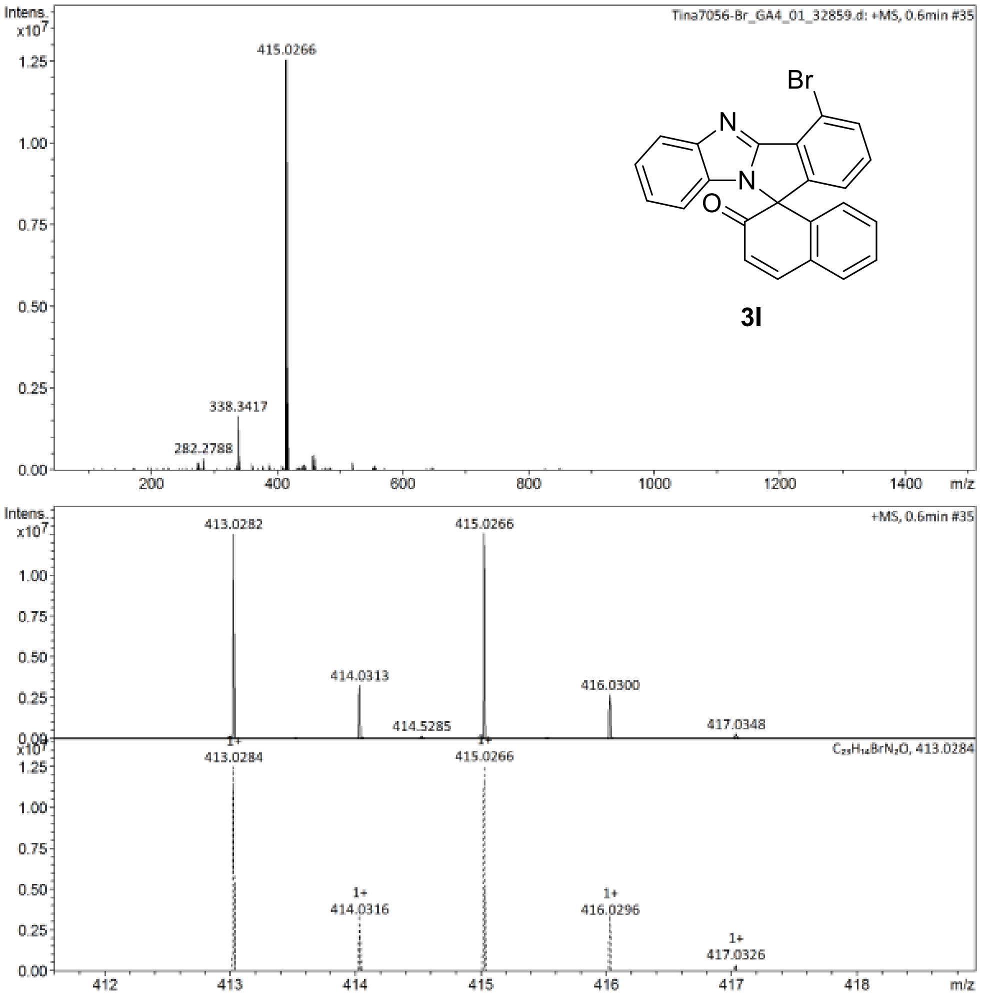

Display Report

Meas. m/z	#	Ion Formula	m/z	err [ppm]	mSigma	# Sigma	Score	rdb	e^- Conf	N-Rule	Adduct
413.0282	1	C23H14BrN2O	413.0284	0.5	16.9	1	100.00	17.5	even	ok	M+H

HRMS (ESI) of compound **3l**.

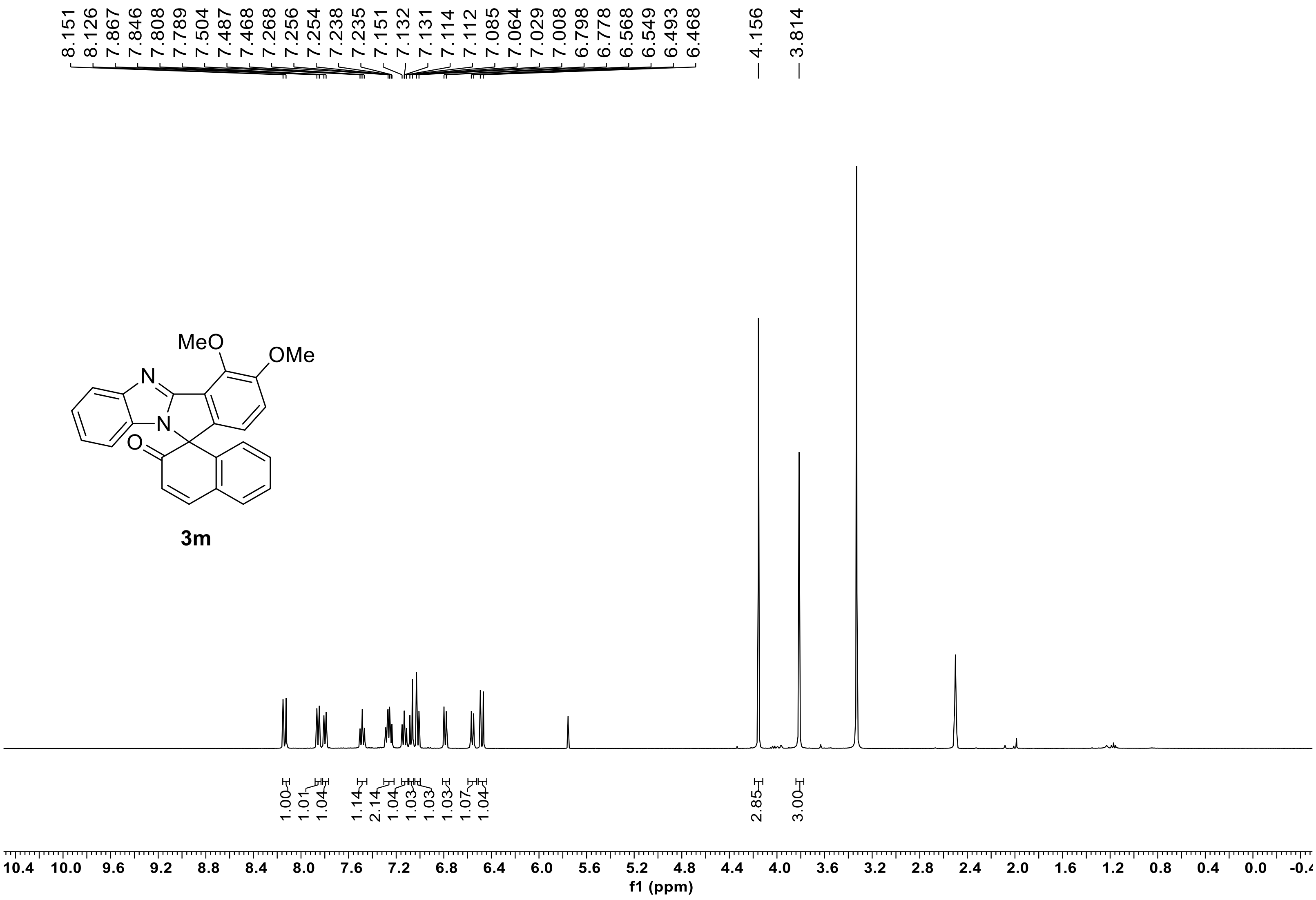

^{1}H NMR spectrum (400 MHz) of compound **3m** in DMSO-d_6.

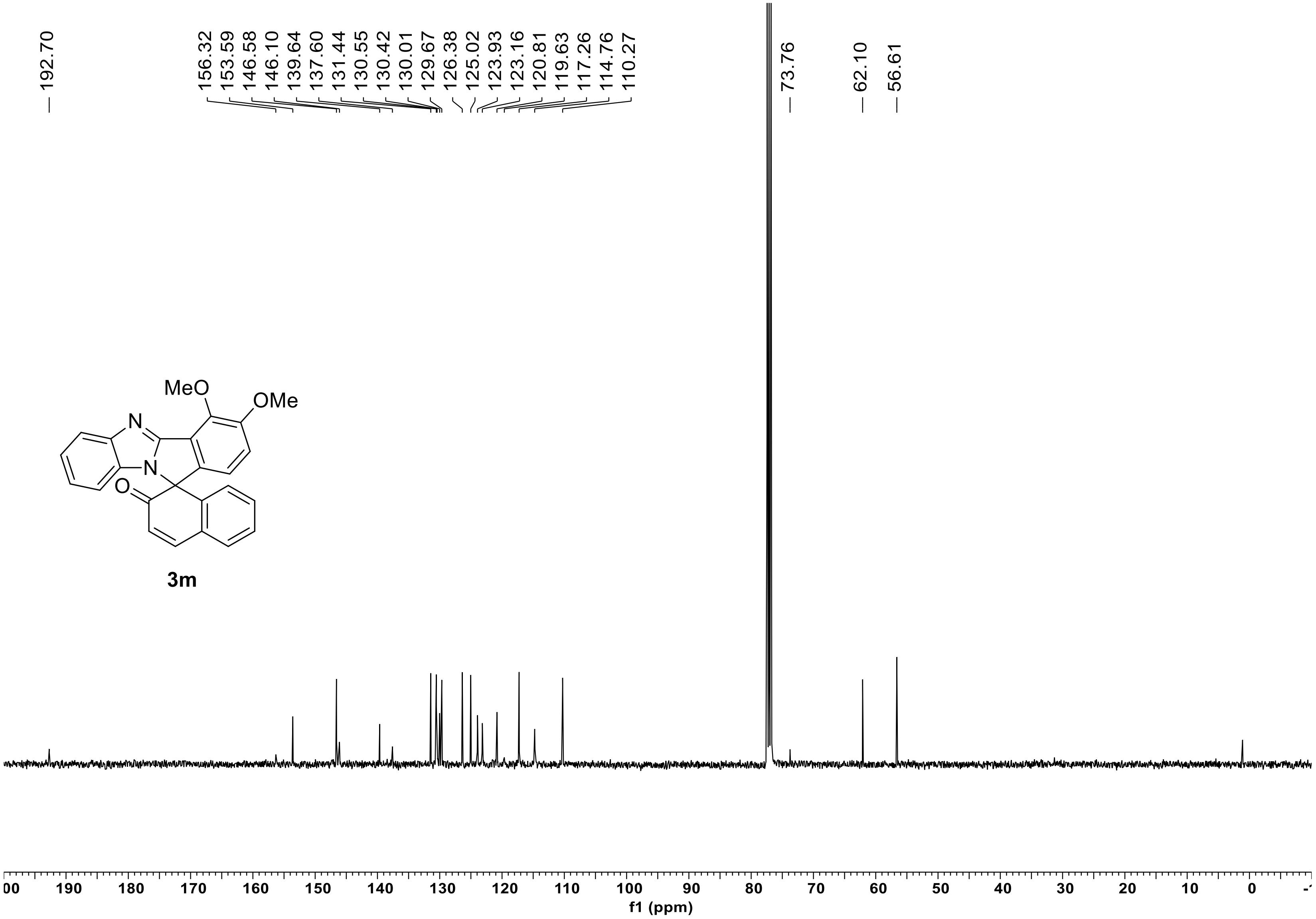

^{13}C{1H} NMR spectrum (101 MHz) of compound **3m** in DMSO-d_6.

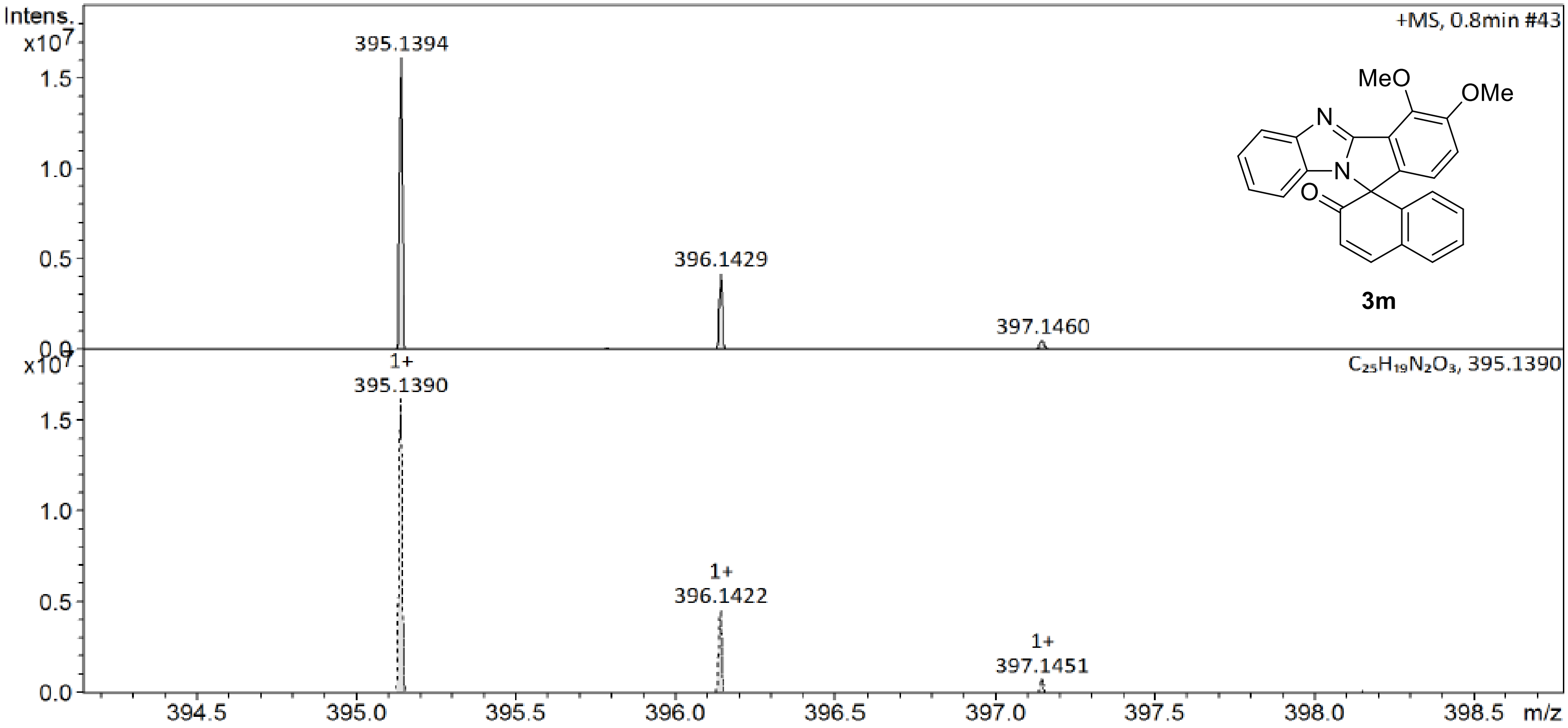

Display Report

Meas. m/z	#	Ion Formula	m/z	err [ppm]	mSigma	# Sigma	Score	rdb	e¯ Conf	N-Rule	Adduct
395.1394	1	C25H19N2O3	395.1390	1.0	12.1	1	100.00	17.5	even	ok	M+H

HRMS (ESI) of compound **3m**.

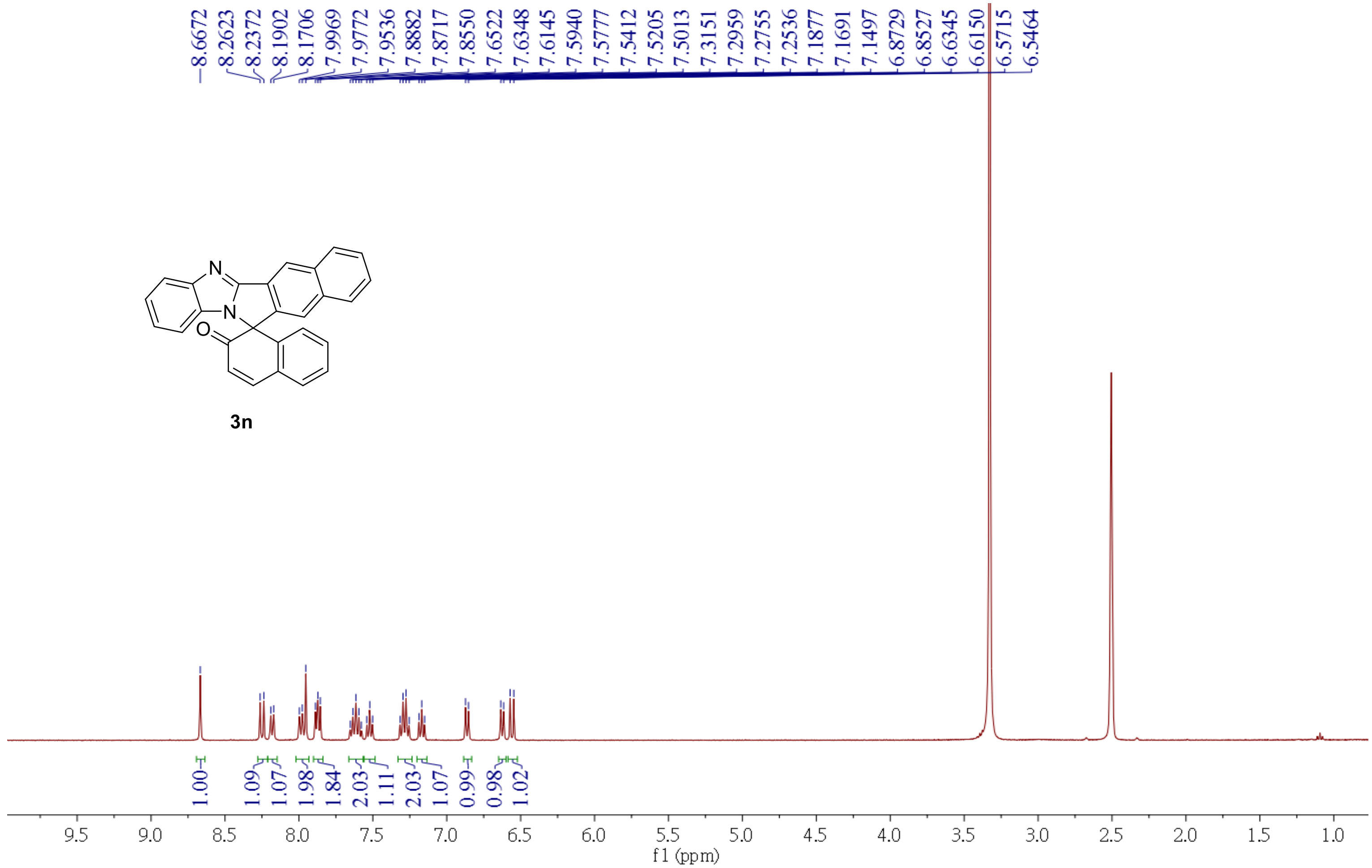

^{1}H NMR spectrum (400 MHz) of compound **3n** in DMSO-d_6

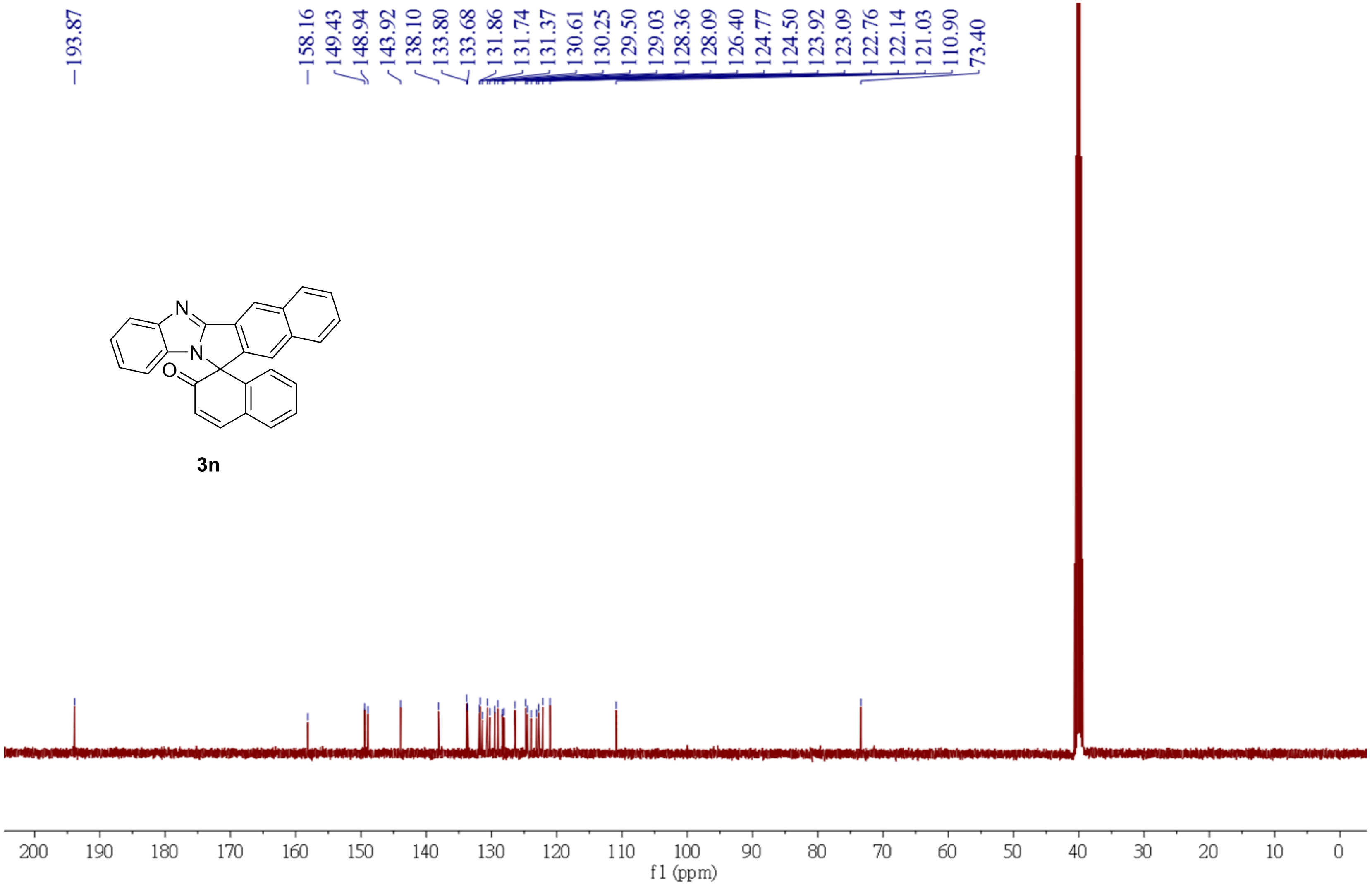

$^{13}C\{1H\}$ NMR spectrum (150 MHz) of compound **3n** in DMSO-d_6

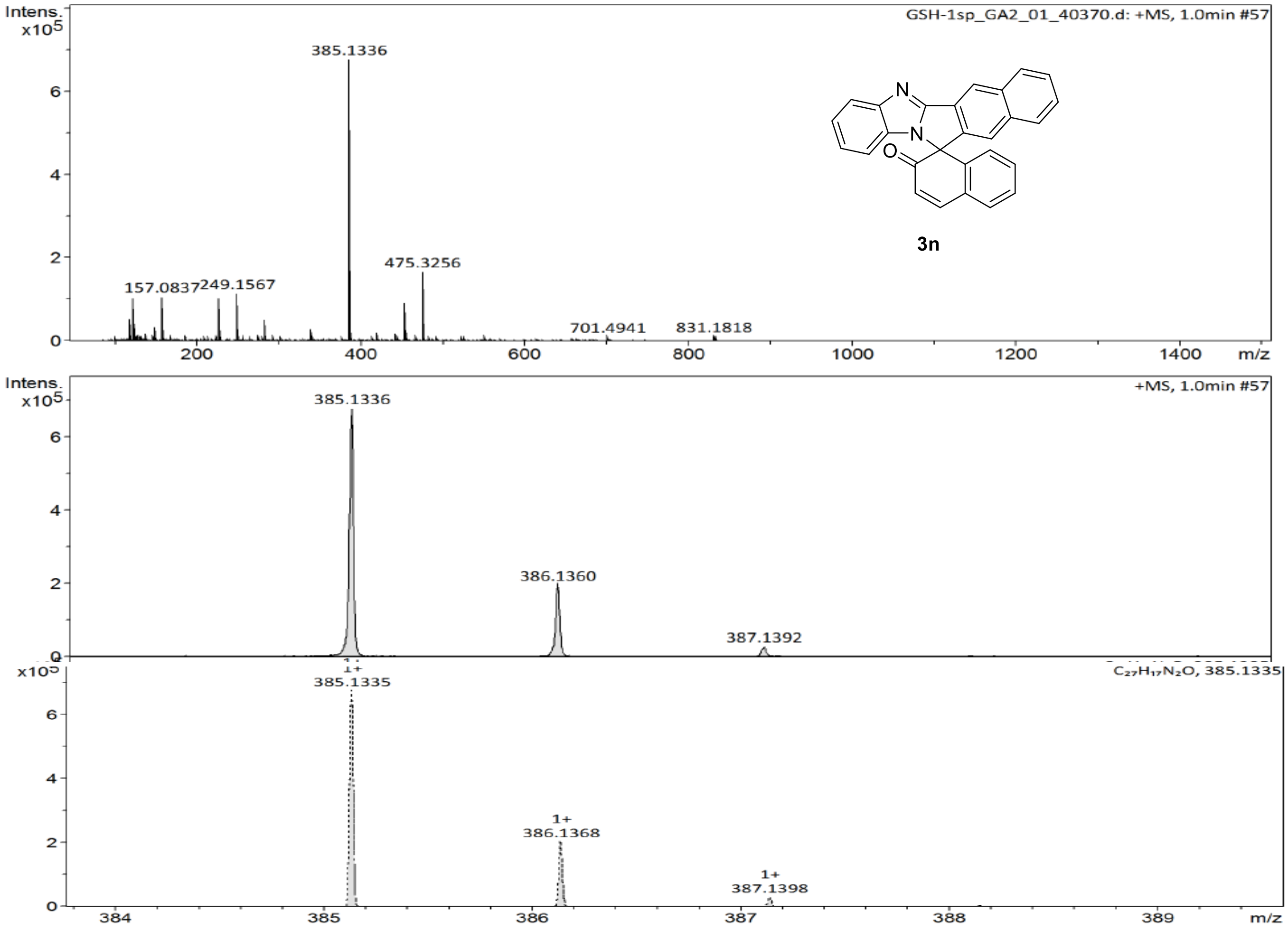

Display Report

Meas. m/z	#	Ion Formula	m/z	err [ppm]	mSigma	# Sigma	Score	rdb	e⁻ Conf	N-Rule	Adduct
385.1336	1	C27H17N2O	385.1335	0.1	7.7	1	100.00	20.5	even	ok	M

HRMS (ESI) of compound **3n**

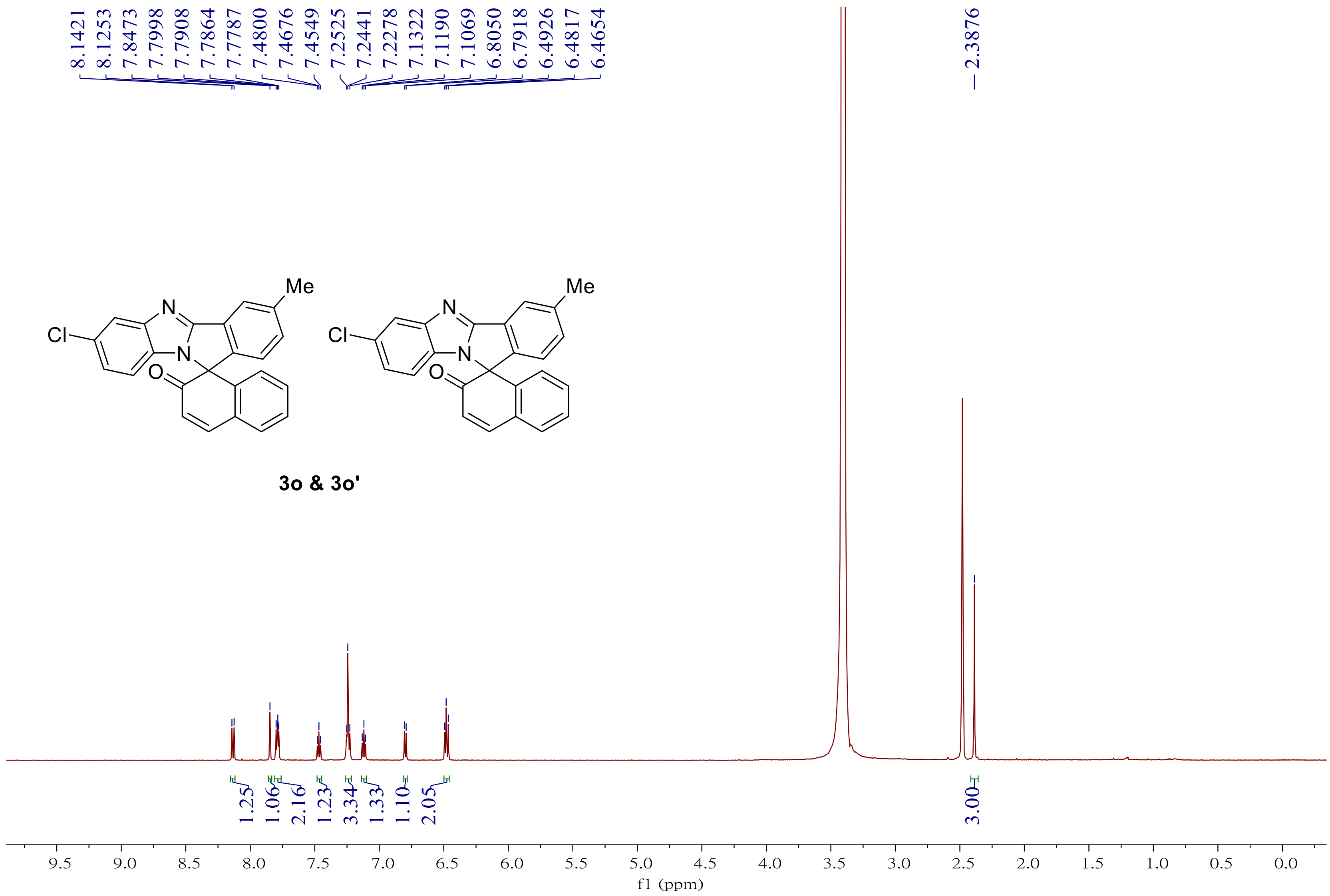

3o & 3o'

^{1}H NMR spectrum (400 MHz) of compound **3o & 3o'** in $CDCl_3$.

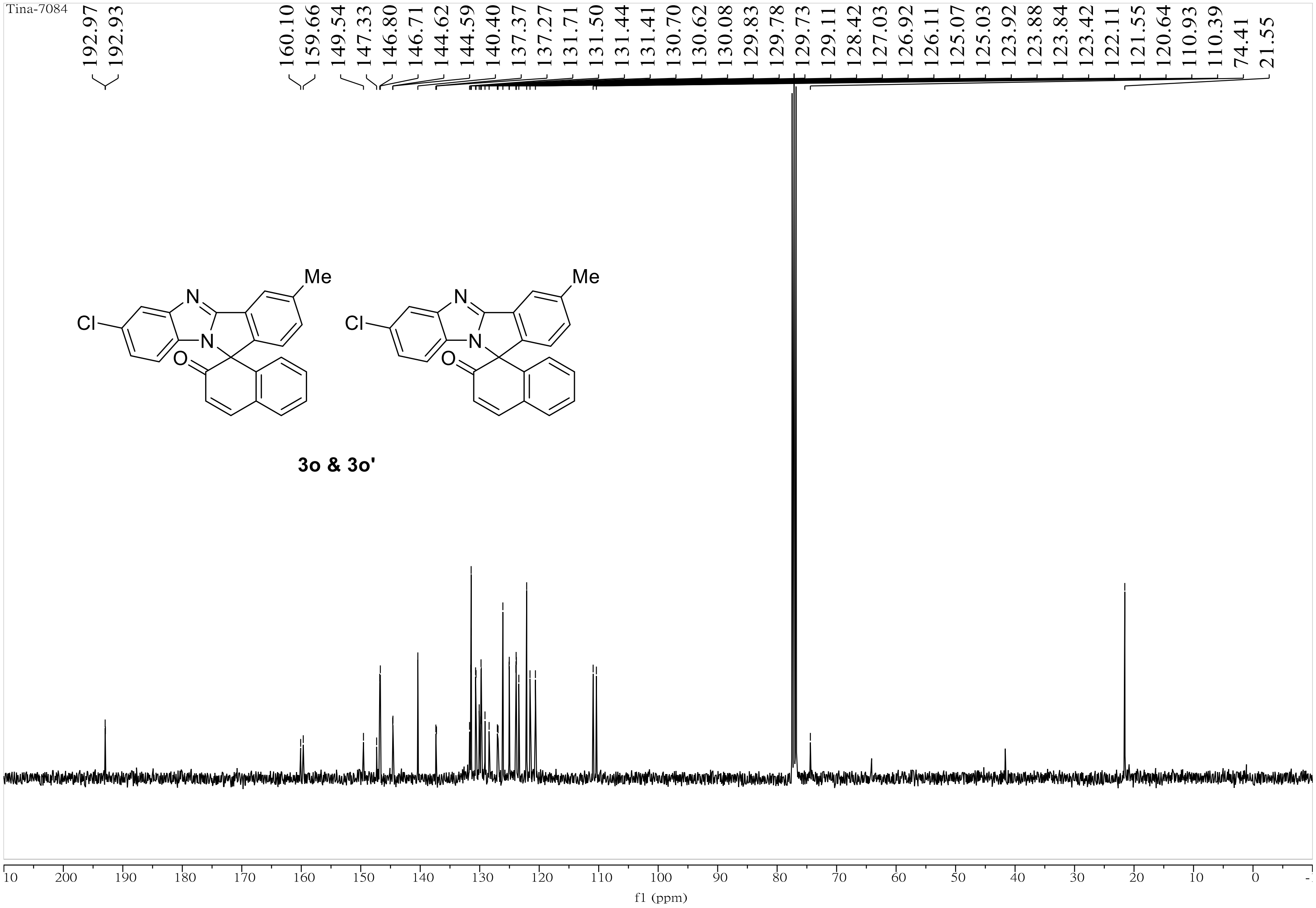

$^{13}C\{1H\}$ NMR spectrum (101 MHz) of compound **3o & 3o'** in $CDCl_3$.

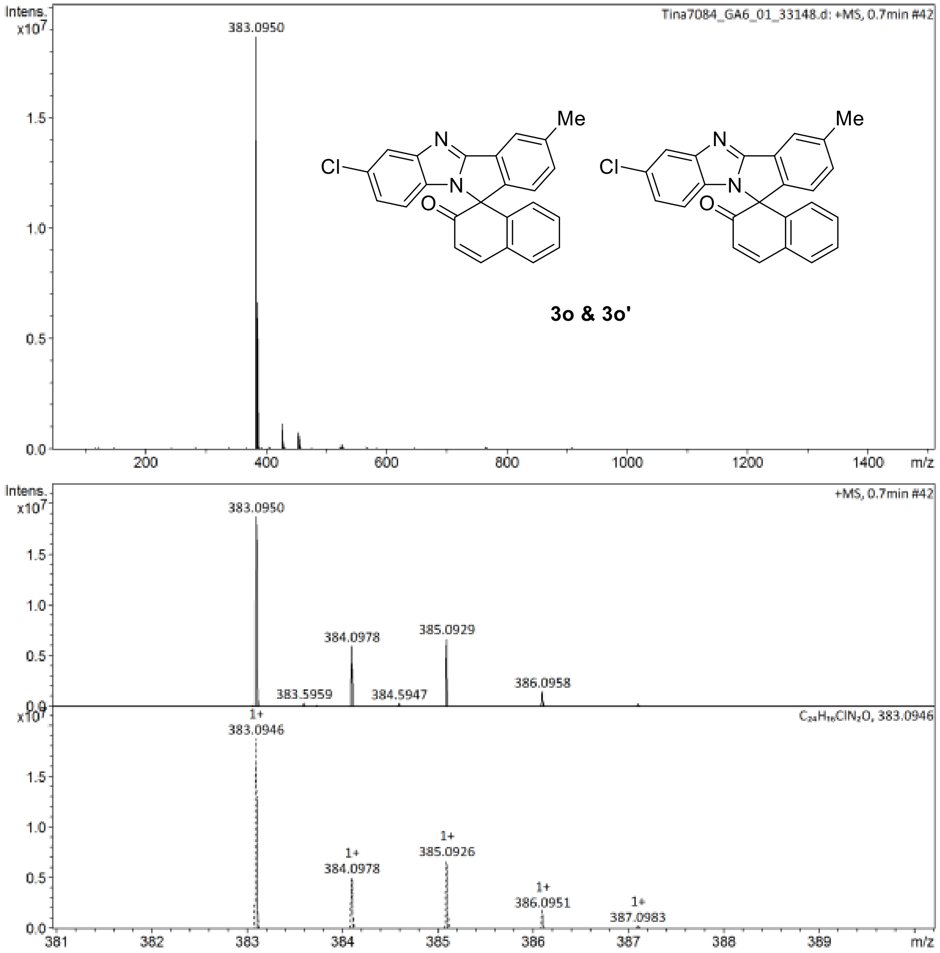

Display Report

Meas. m/z	#	Ion Formula	m/z	err [ppm]	mSigma	# Sigma	Score	rdb	e⁻ Conf	N-Rule	Adduct
383.0950	1	C24H16ClN2O	383.0946	-1.0	23.1	1	100.00	17.5	even	ok	M+H

HRMS (ESI) of compound **3o & 3o'**

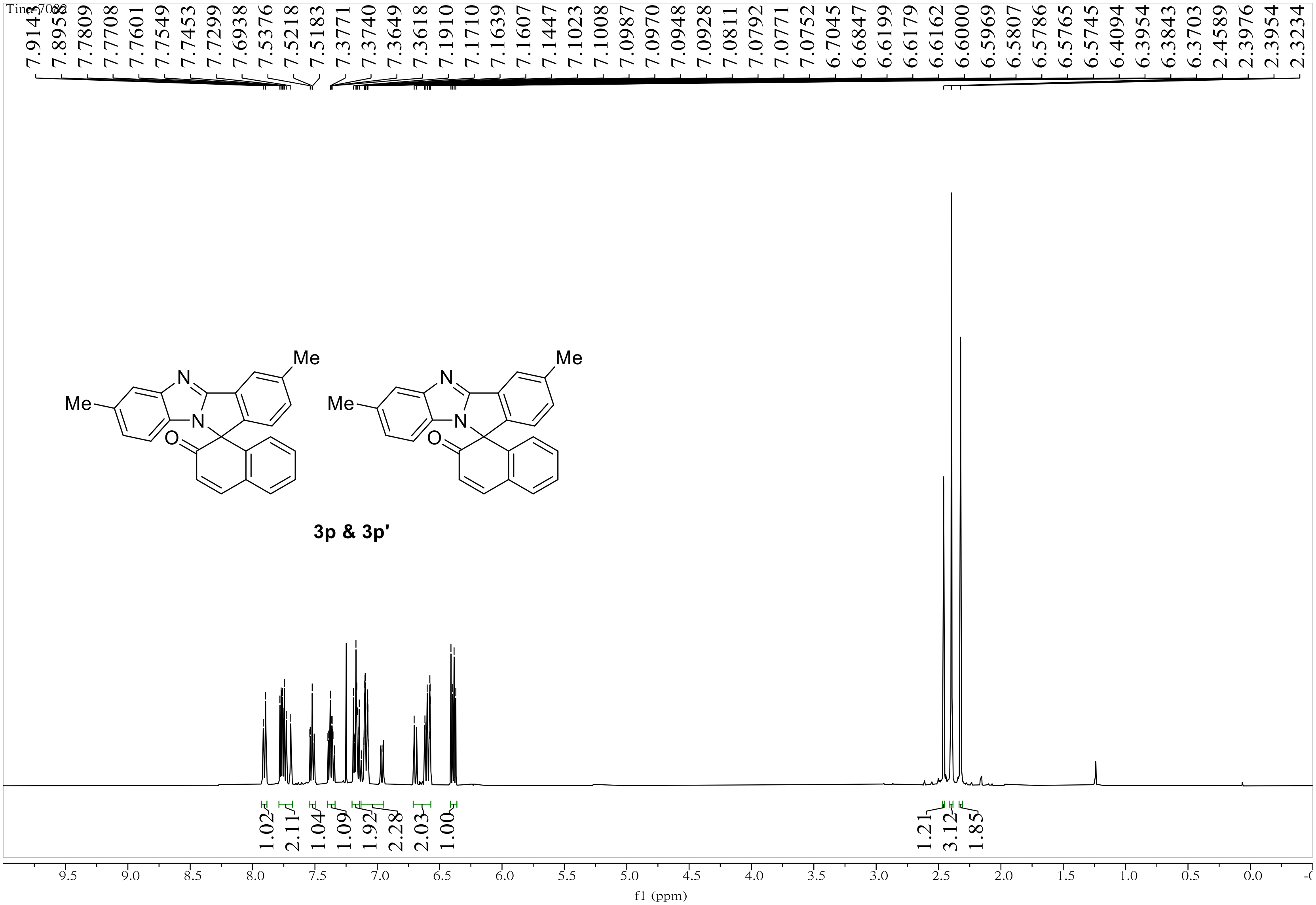

^{1}H NMR spectrum (400 MHz) of compound **3p & 3p'** in $CDCl_3$.

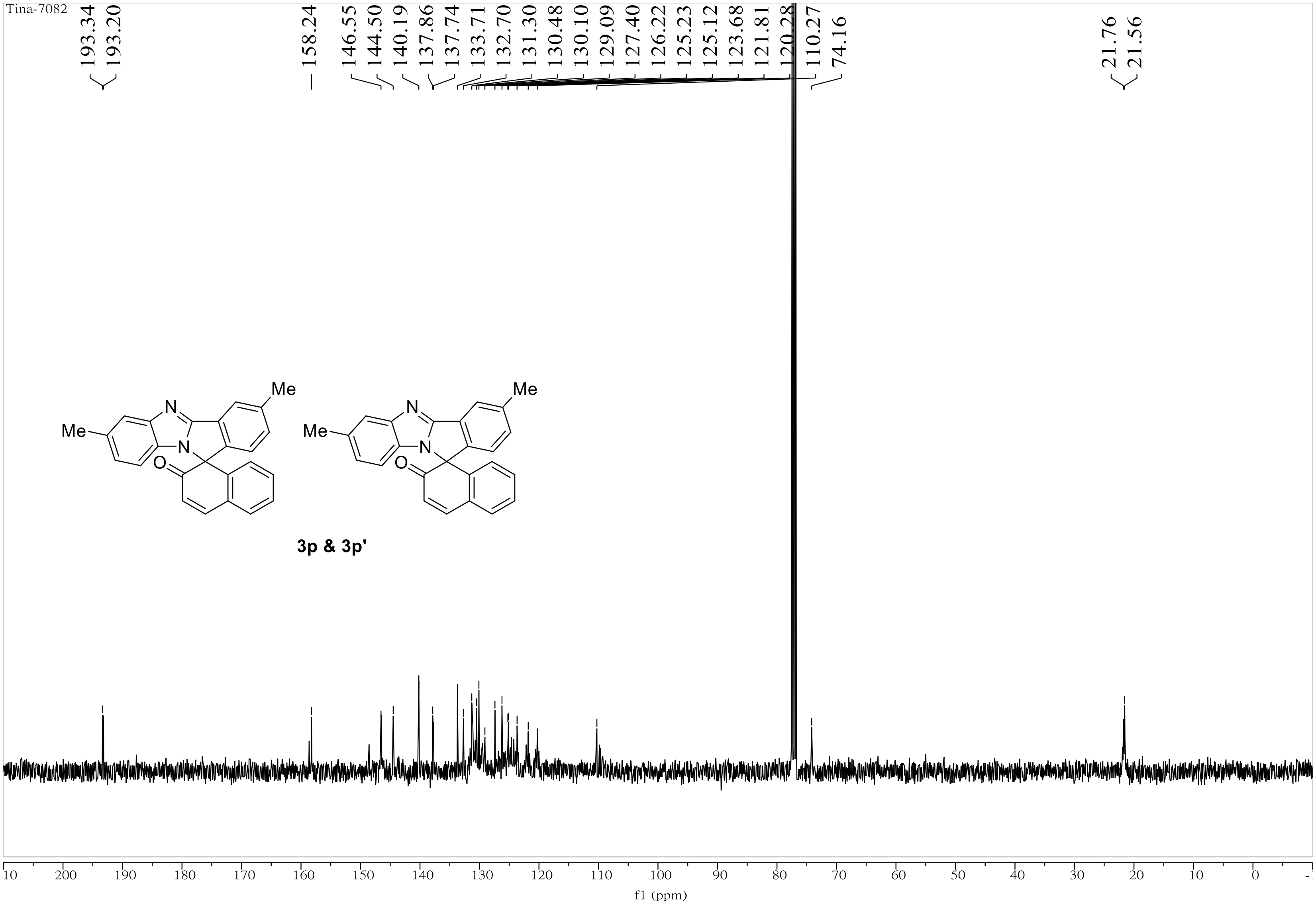

$^{13}C\{1H\}$ NMR spectrum (101 MHz) of compound **3p & 3p'** in DMSO-d_6.

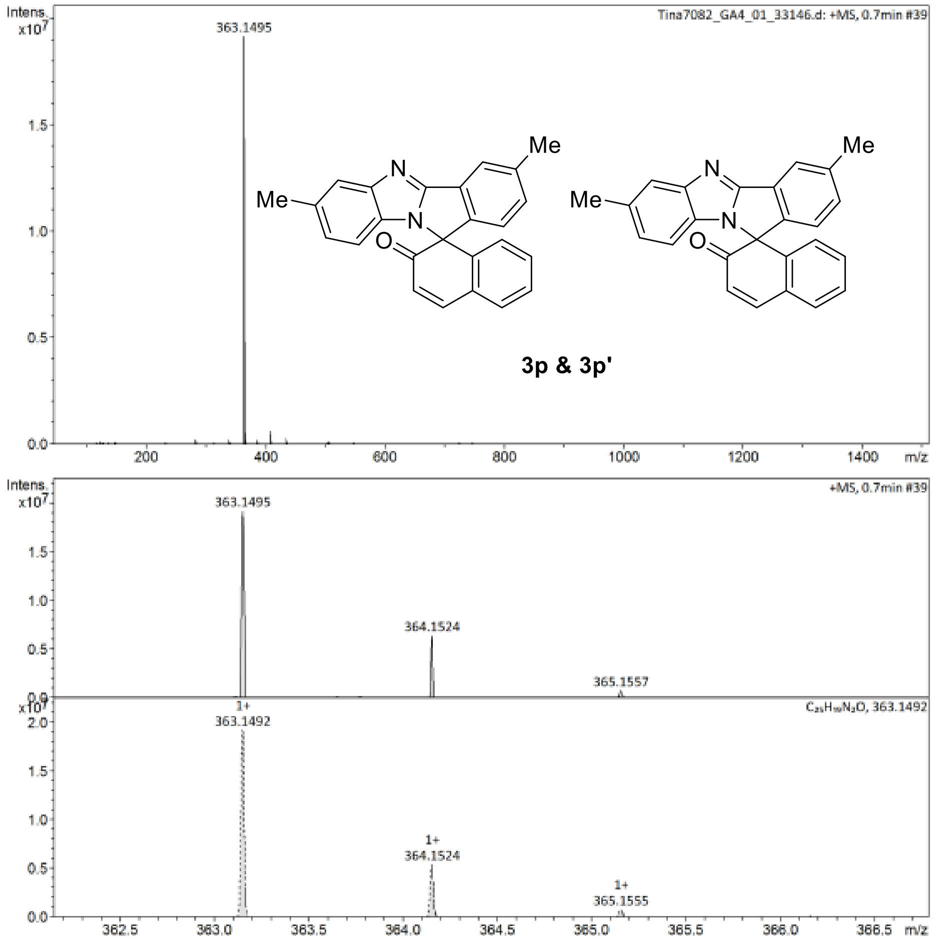

Display Report

Meas. m/z	#	Ion Formula	m/z	err [ppm]	mSigma	# Sigma	Score	rdb	e⁻ Conf	N-Rule	Adduct
363.1495	1	C25H19N2O	363.1492	0.8	26.3	1	100.00	17.5	even	ok	M+H

HRMS (ESI) of compound **3p & 3p'**

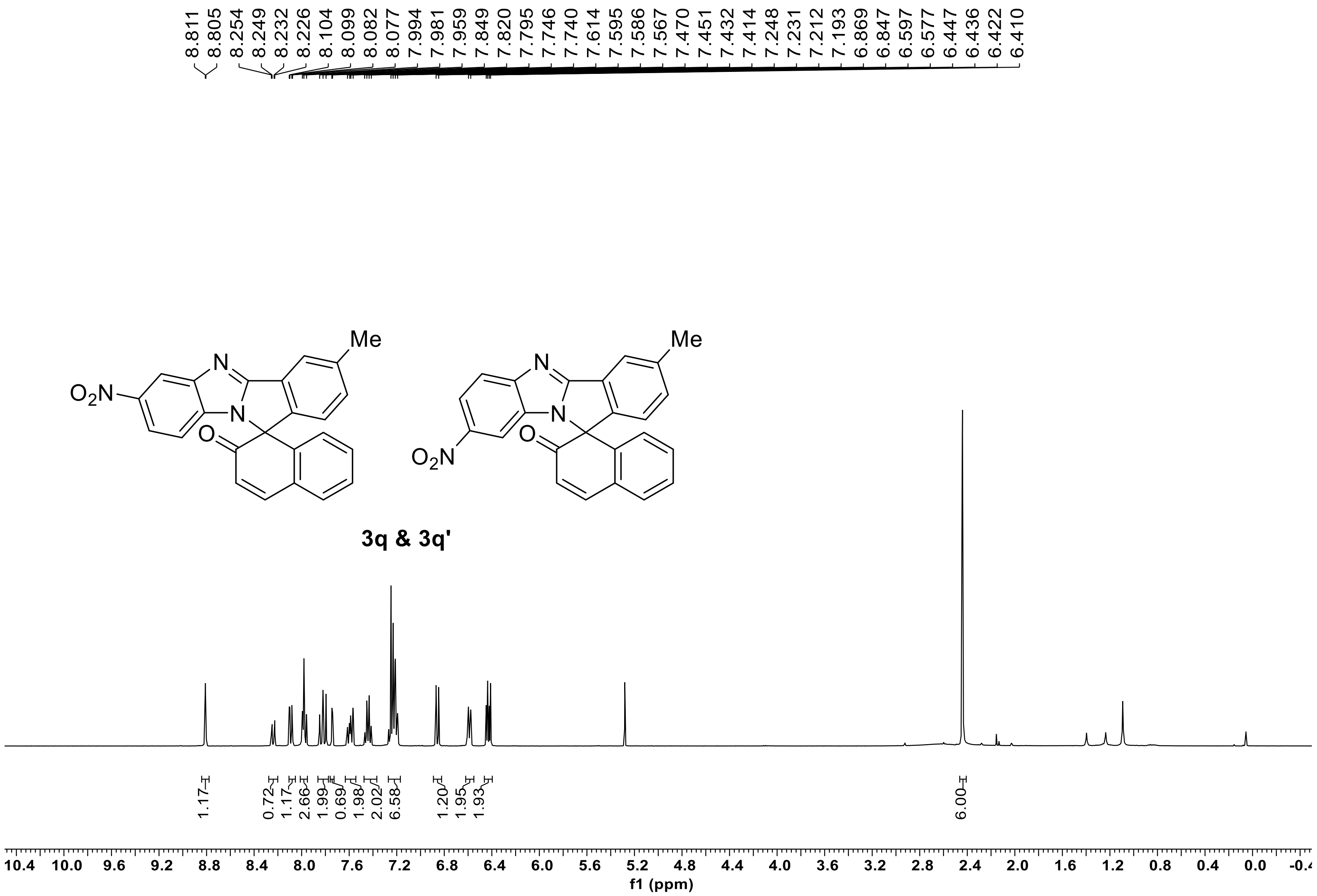

^{1}H NMR spectrum (400 MHz) of compound **3q & 3q'** in $CDCl_3$.

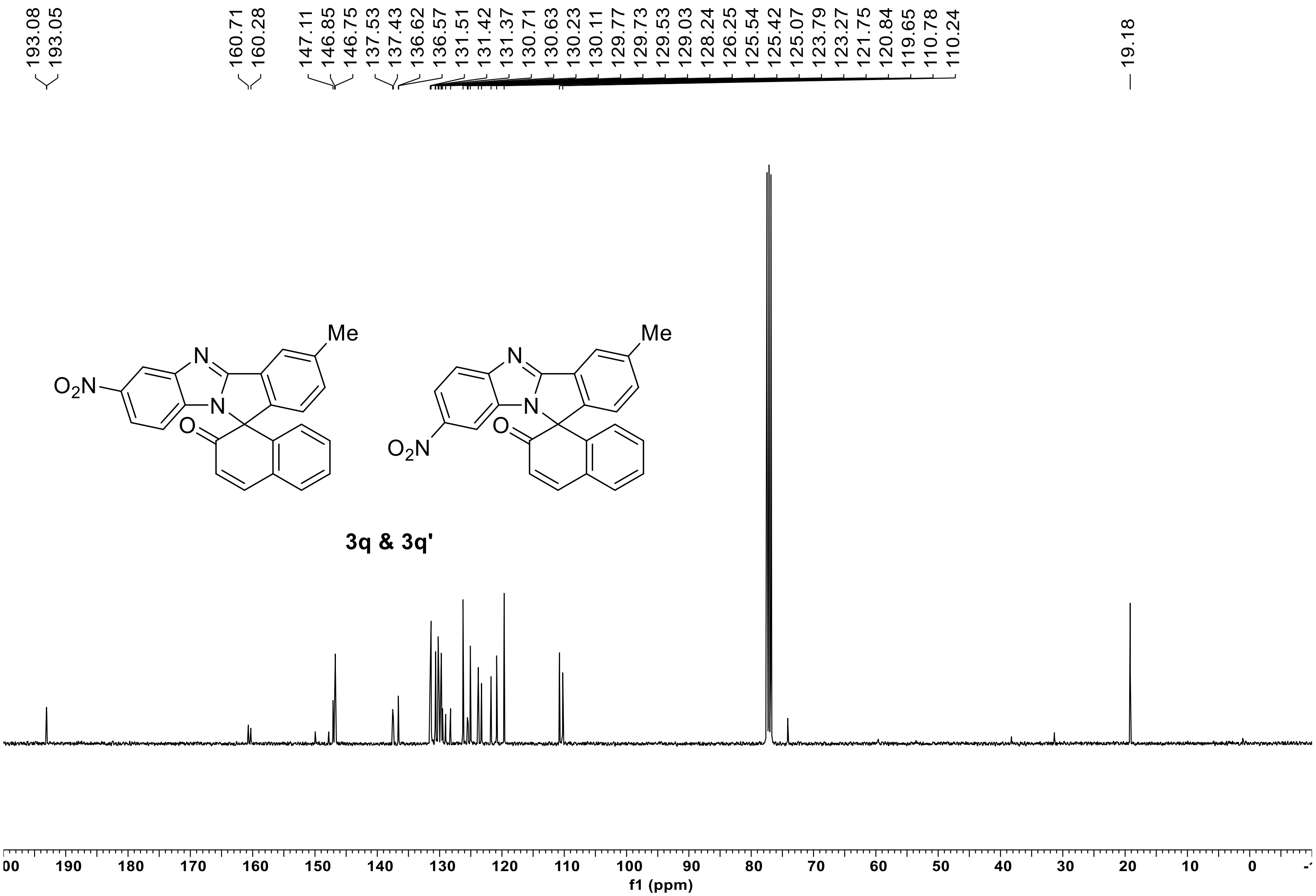

$^{13}C\{1H\}$ NMR spectrum (101 MHz) of compound **3q & 3q'** in DMSO-d_6.

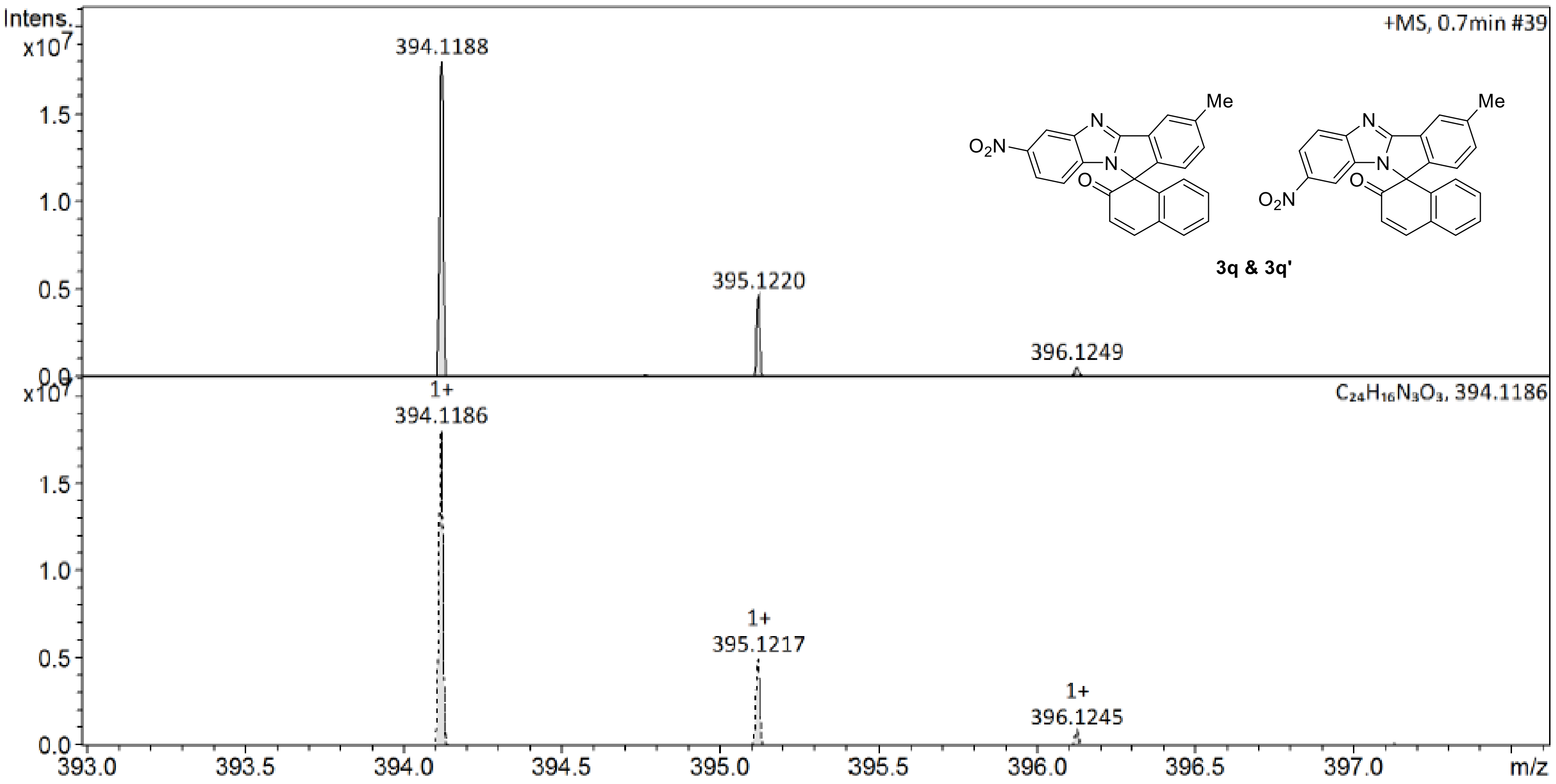

Display Report

Meas. m/z	#	Ion Formula	m/z	err [ppm]	mSigma	# Sigma	Score	rdb	e⁻ Conf	N-Rule	Adduct
394.1188	1	C24H16N3O3	394.1186	0.6	9.9	1	100.00	18.5	even	ok	M+H

HRMS (ESI) of compound **3q & 3q'**

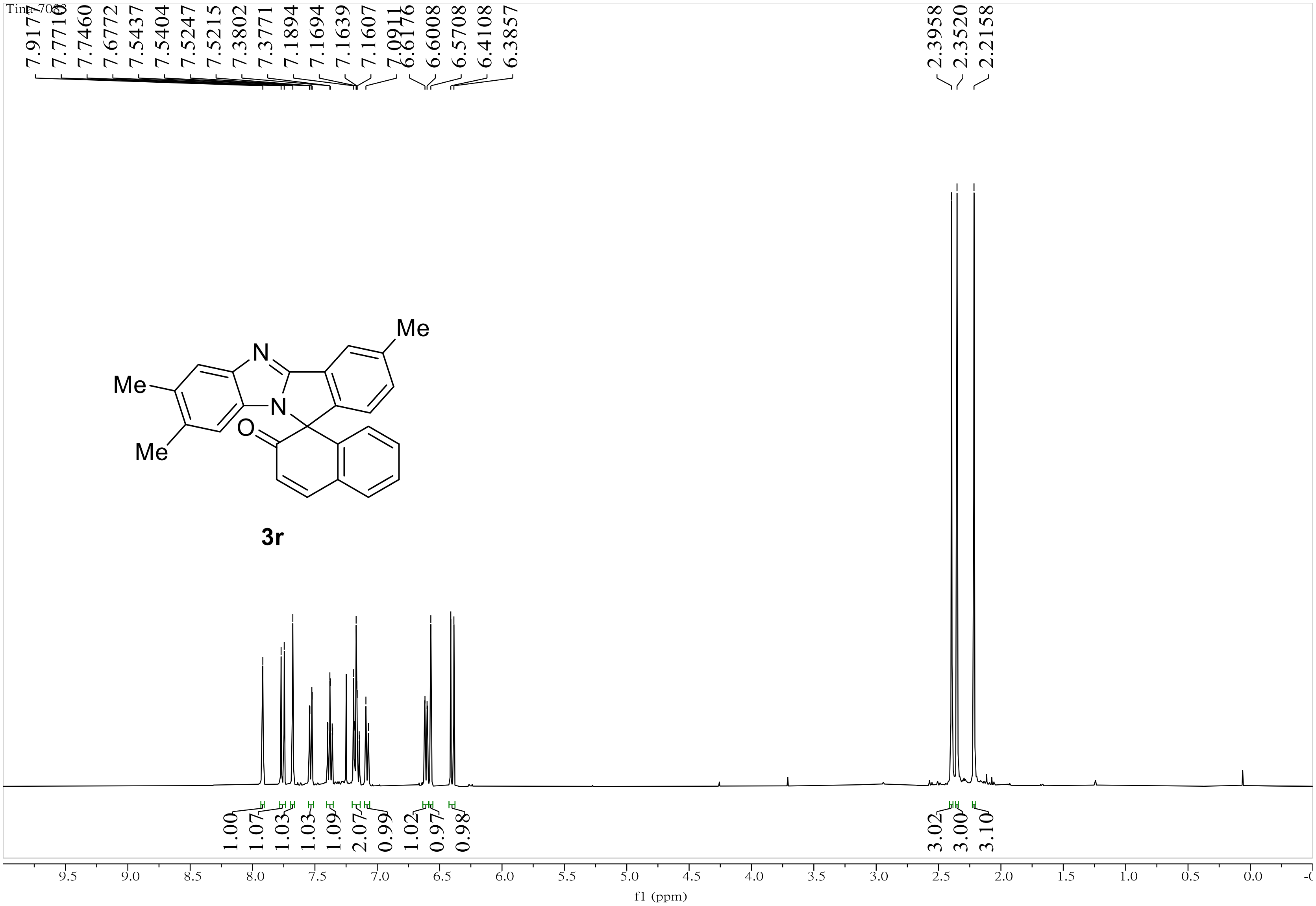

^{1}H NMR spectrum (400 MHz) of compound **3r** in $CDCl_3$.

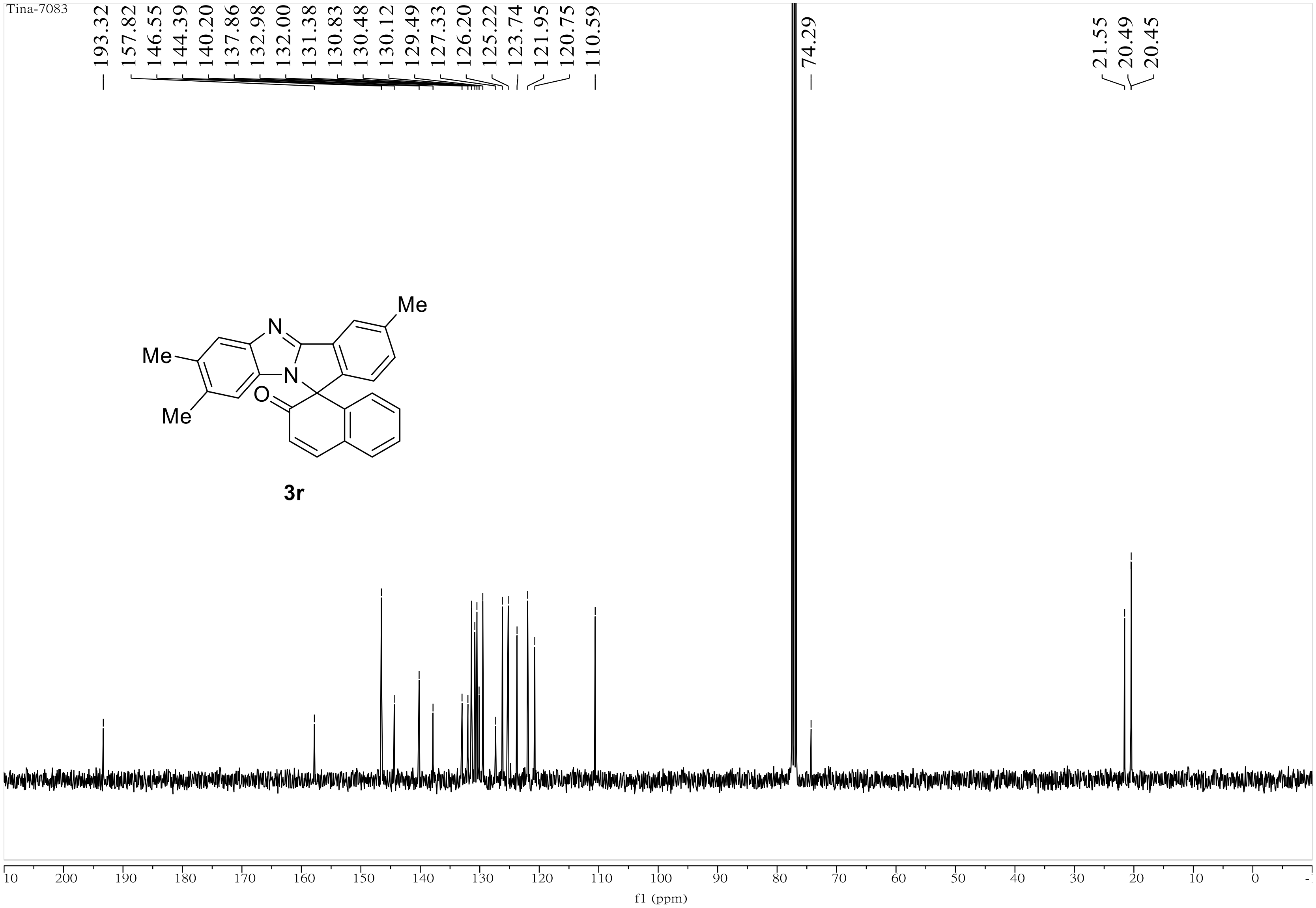

$^{13}C\{1H\}$ NMR spectrum (101 MHz) of compound **3r** in $CDCl_3$.

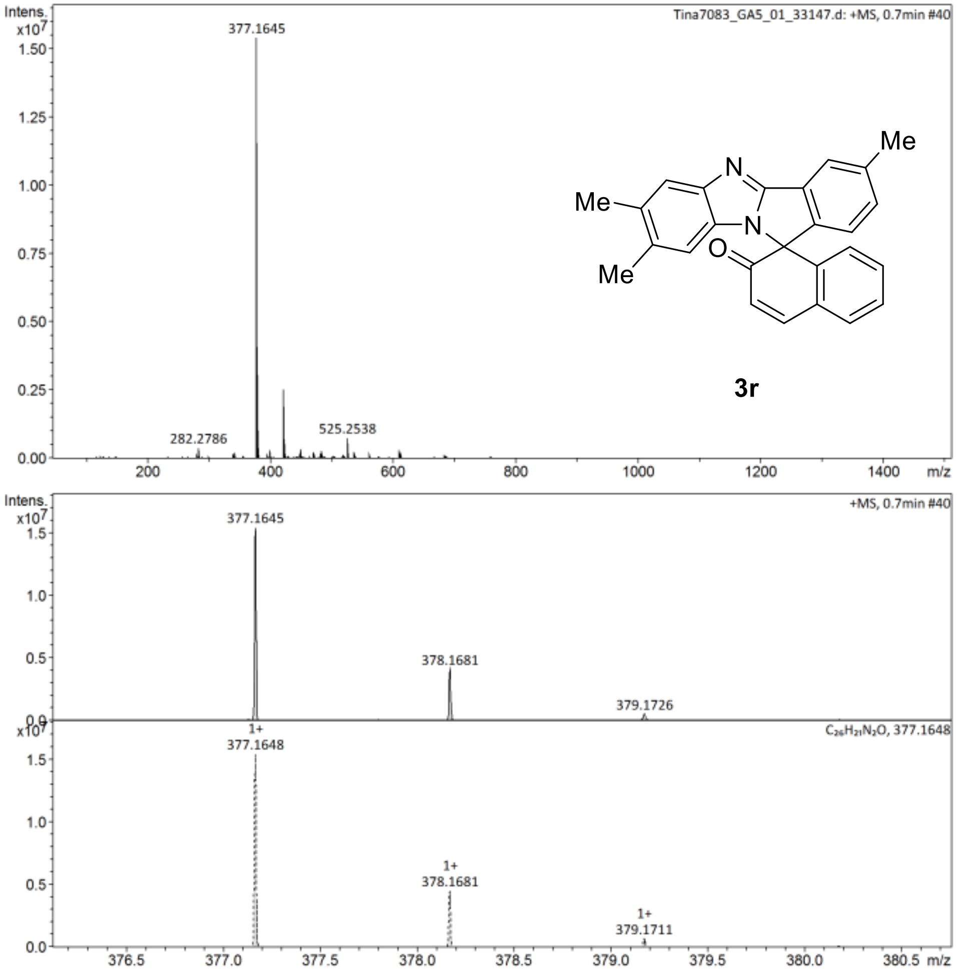

Display Report

Meas. m/z	#	Ion Formula	m/z	err [ppm]	mSigma	# Sigma	Score	rdb	e⁻ Conf	N-Rule	Adduct
377.1645	1	C26H21N2O	377.1648	0.8	14.5	1	100.00	17.5	even	ok	M+H

HRMS (ESI) of compound **3r**

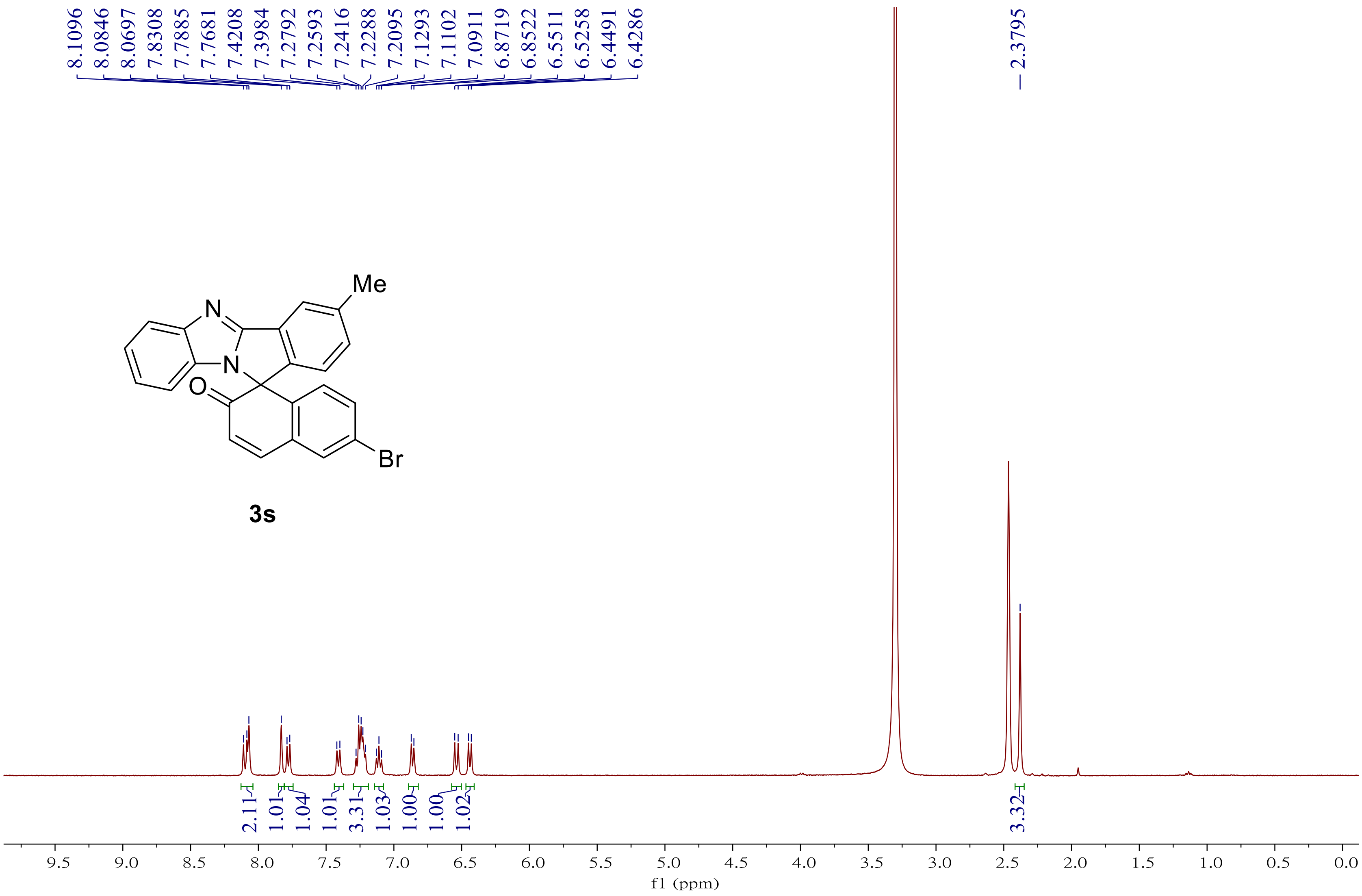

^{1}H NMR spectrum (400 MHz) of compound **3s** in DMSO-d_6.

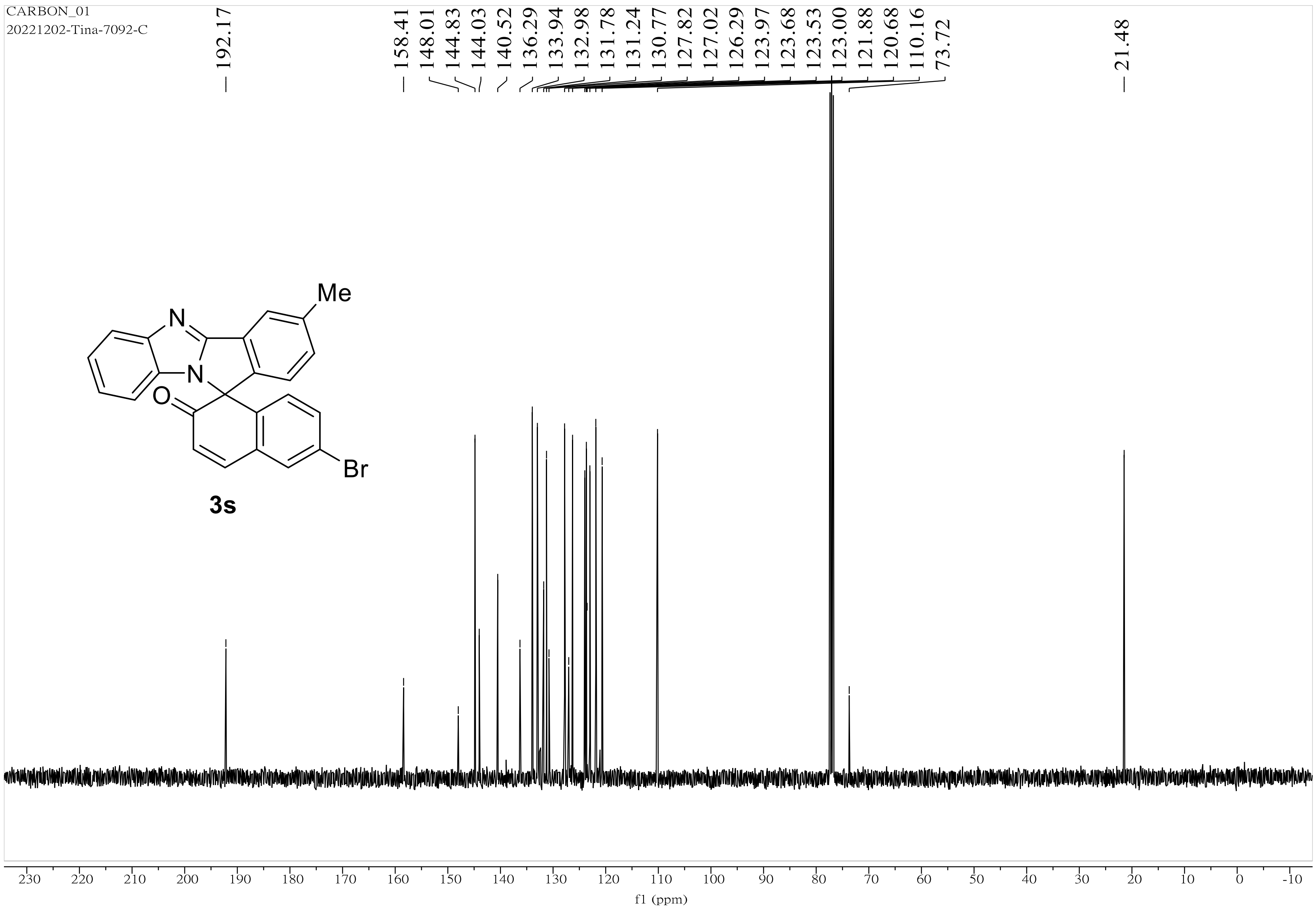

^{13}C{1H} NMR spectrum (101 MHz) of compound **3s** in $CDCl_3$.

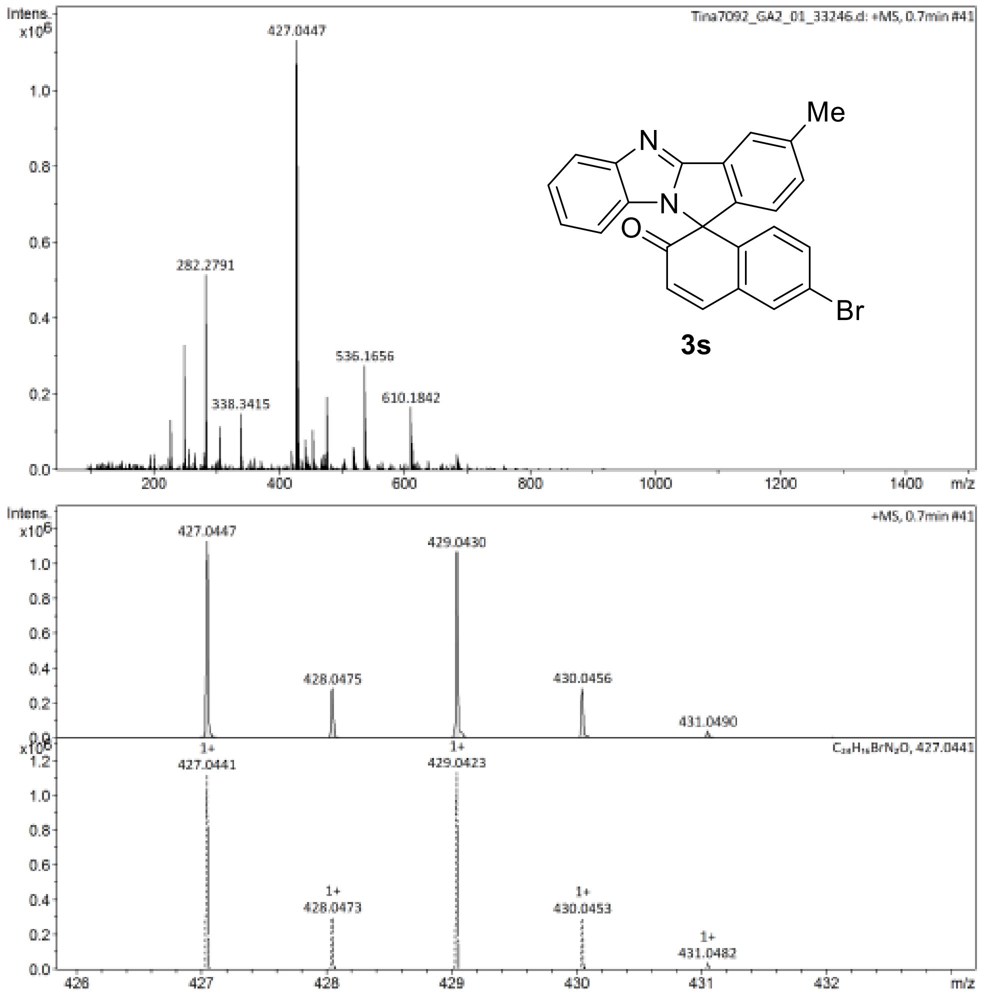

Display Report

Meas. m/z	#	Ion Formula	m/z	err [ppm]	mSigma	# Sigma	Score	rdb	e^- Conf	N-Rule	Adduct
427.0447	1	C24H16BrN2O	427.0441	1.6	26.6	1	100.00	17.5	even	ok	M+H

HRMS (ESI) of compound **3s**

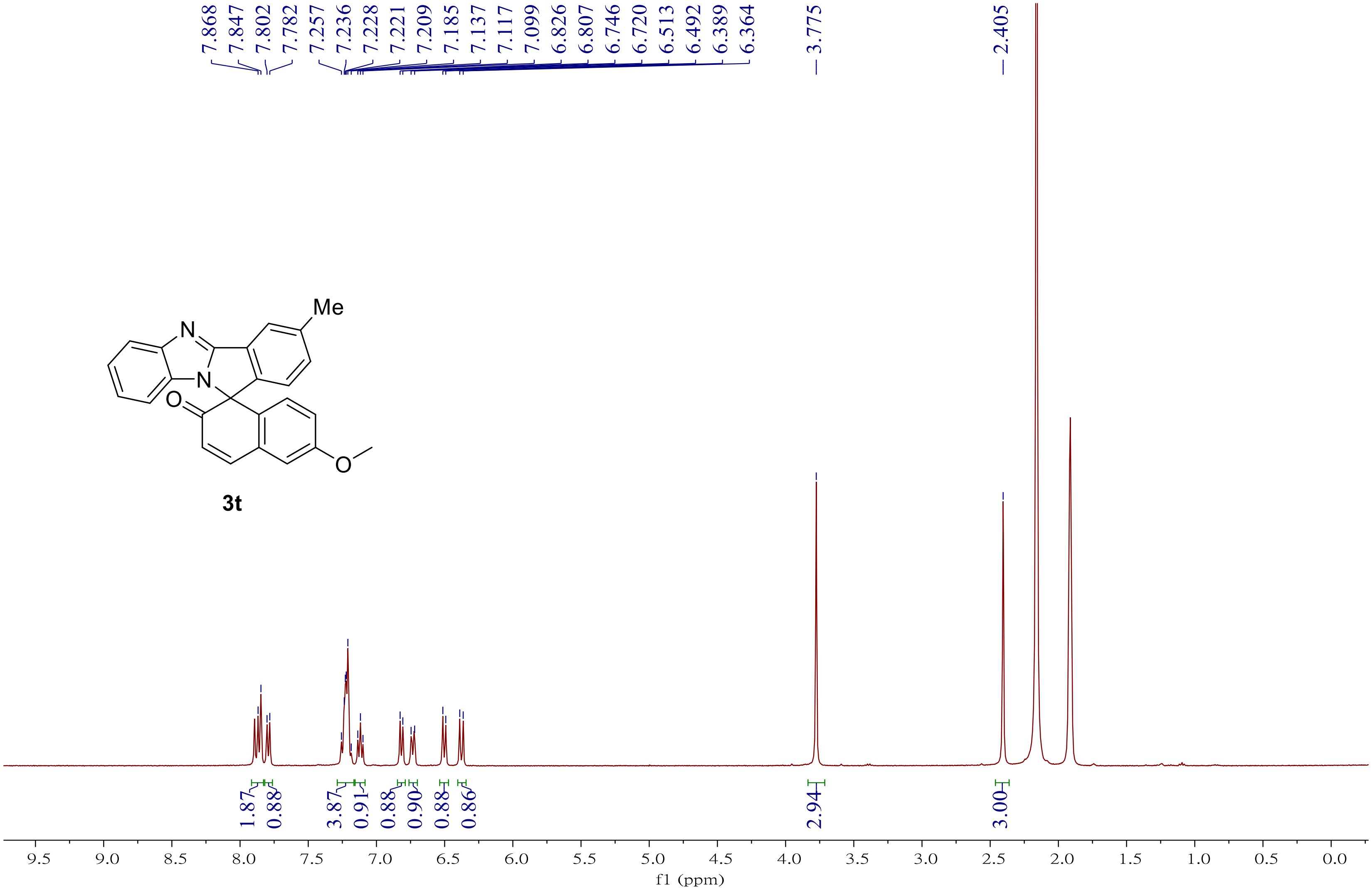

^{1}H NMR spectrum (400 MHz) of compound **3t** in Acetonitrile-d_3.

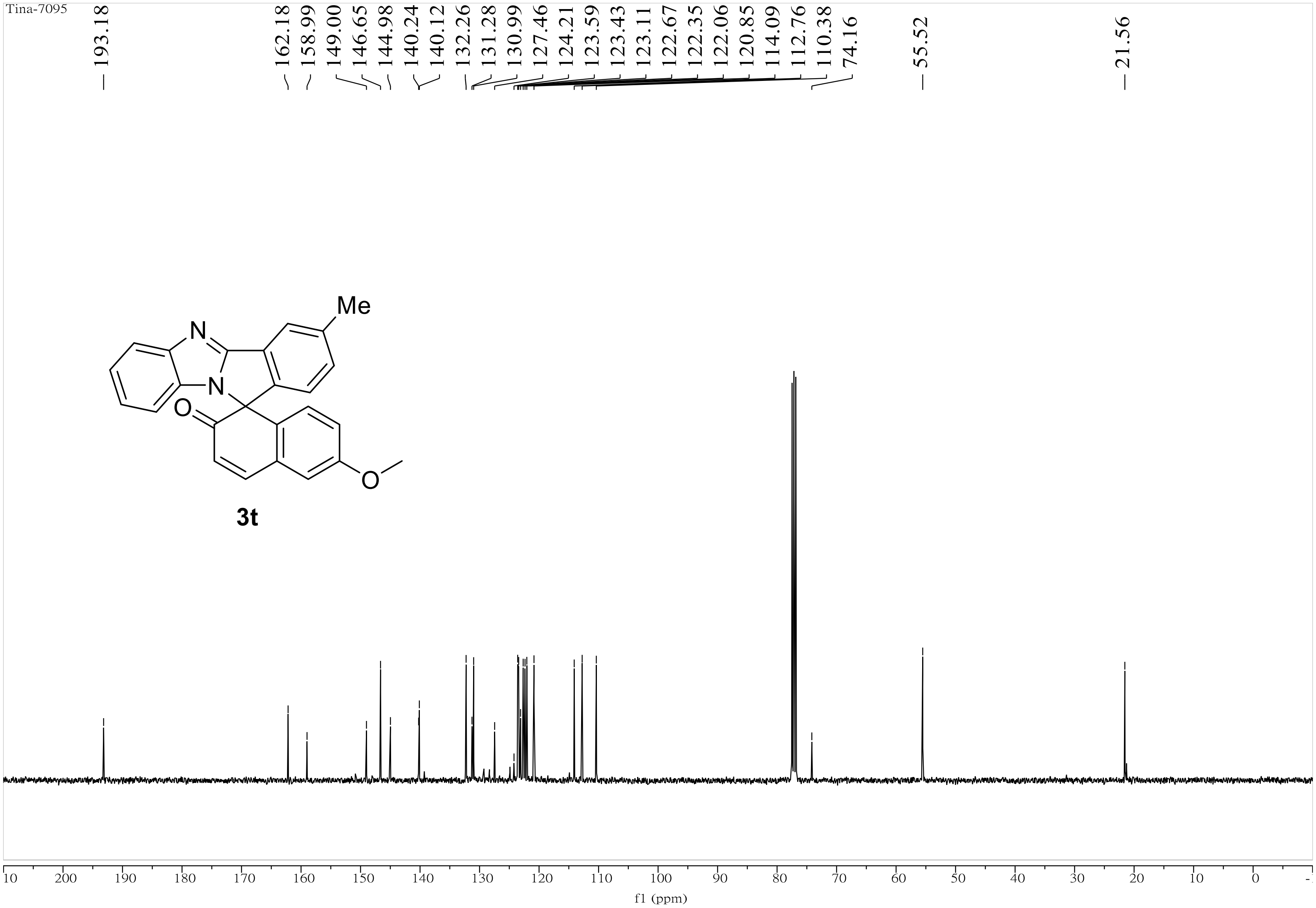

$^{13}C\{1H\}$ NMR spectrum (101 MHz) of compound **3t** in $CDCl_3$.

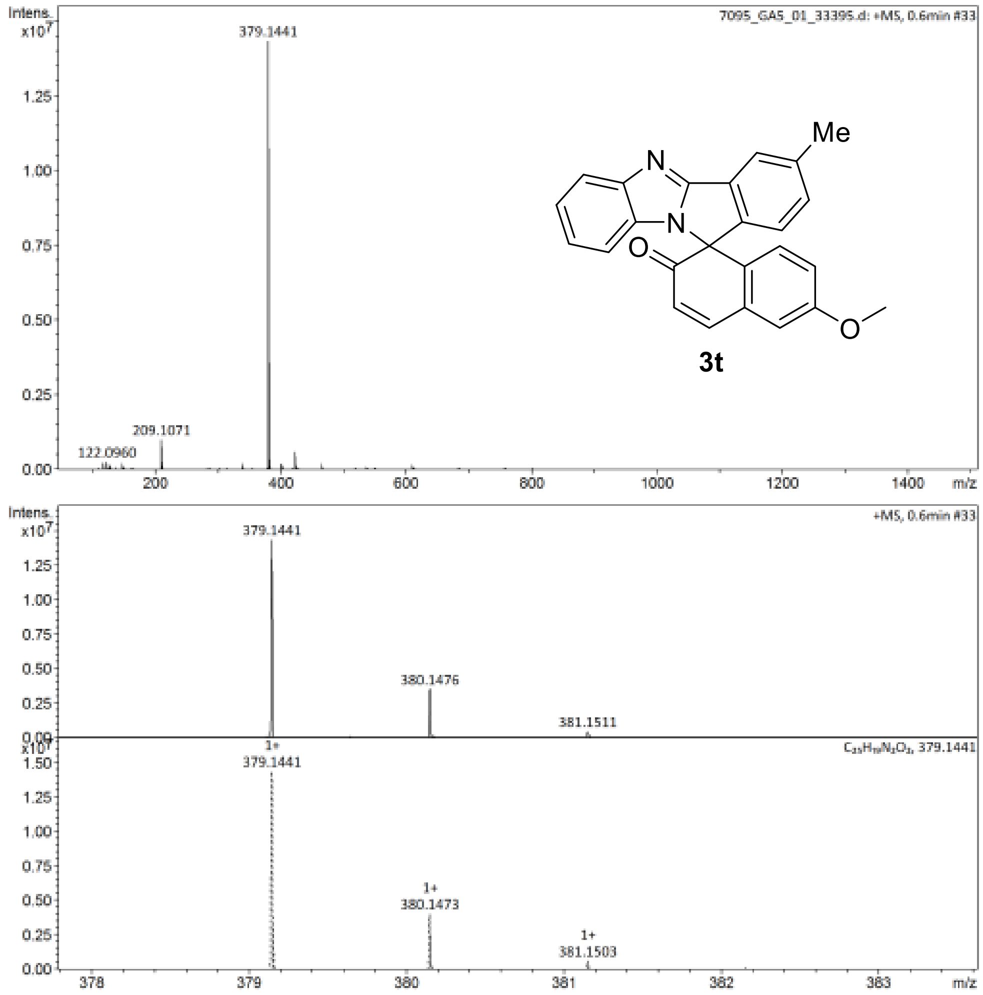

Display Report

Meas. m/z	#	Ion Formula	m/z	err [ppm]	mSigma	# Sigma	Score	rdb	e^- Conf	N-Rule	Adduct
379.1441	1	C25H19N2O2	379.1441	0.1	19.4	1	100.00	17.5	even	ok	M+H

HRMS (ESI) of compound **3t**

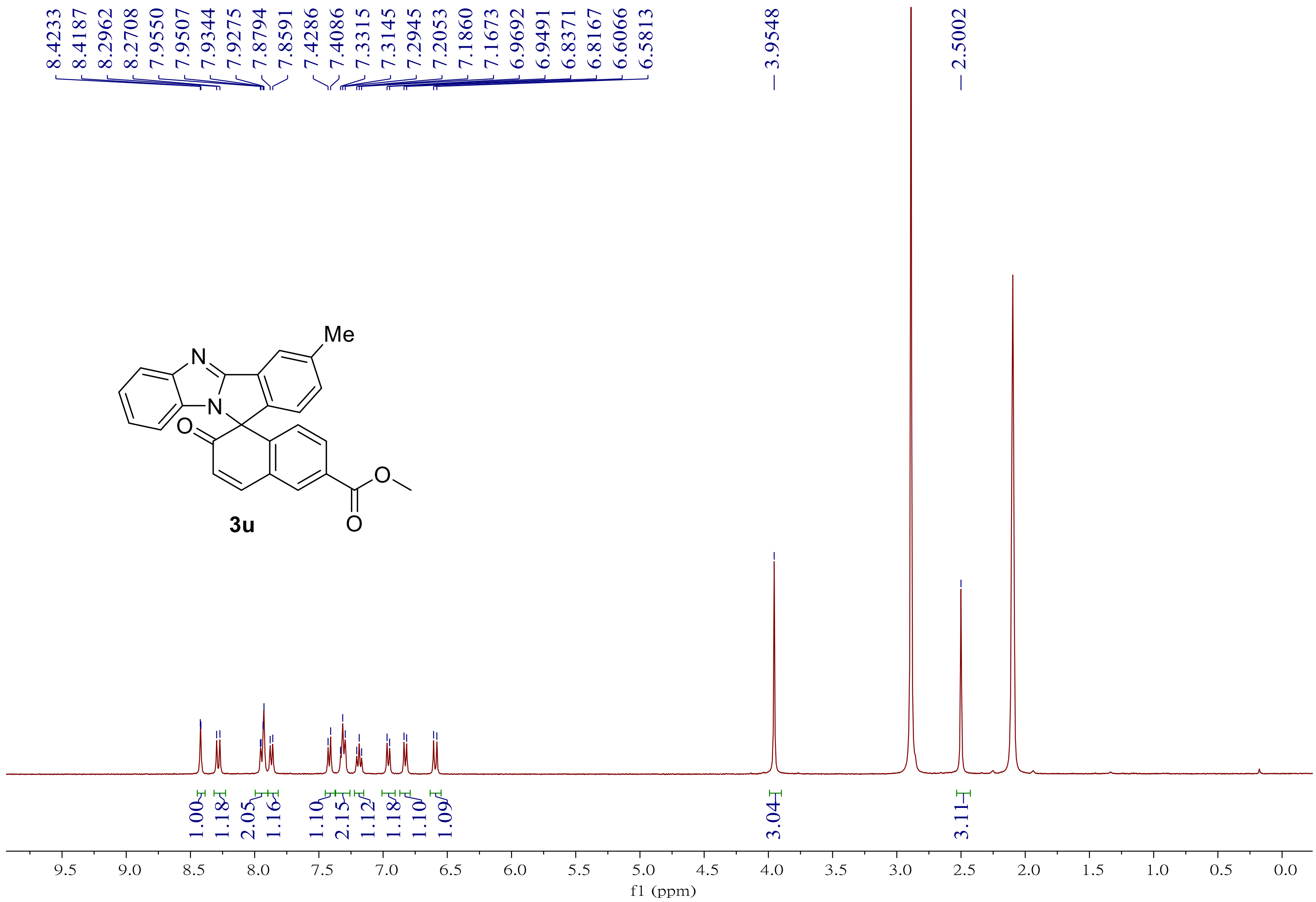

^{1}H NMR spectrum (400 MHz) of compound **3u** in Acetone-d_6.

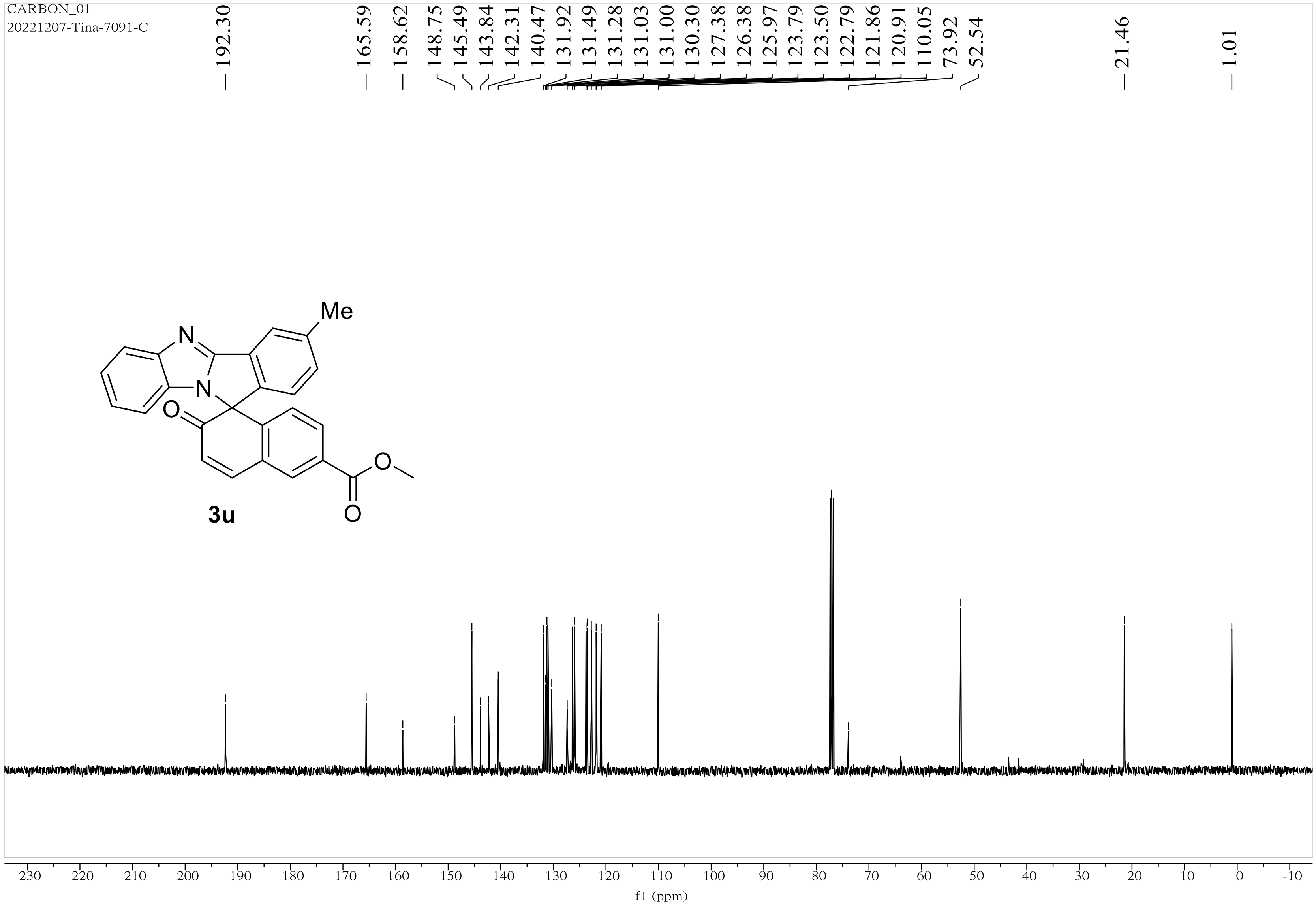

$^{13}C\{1H\}$ NMR spectrum (101 MHz) of compound **3u** in $CDCl_3$.

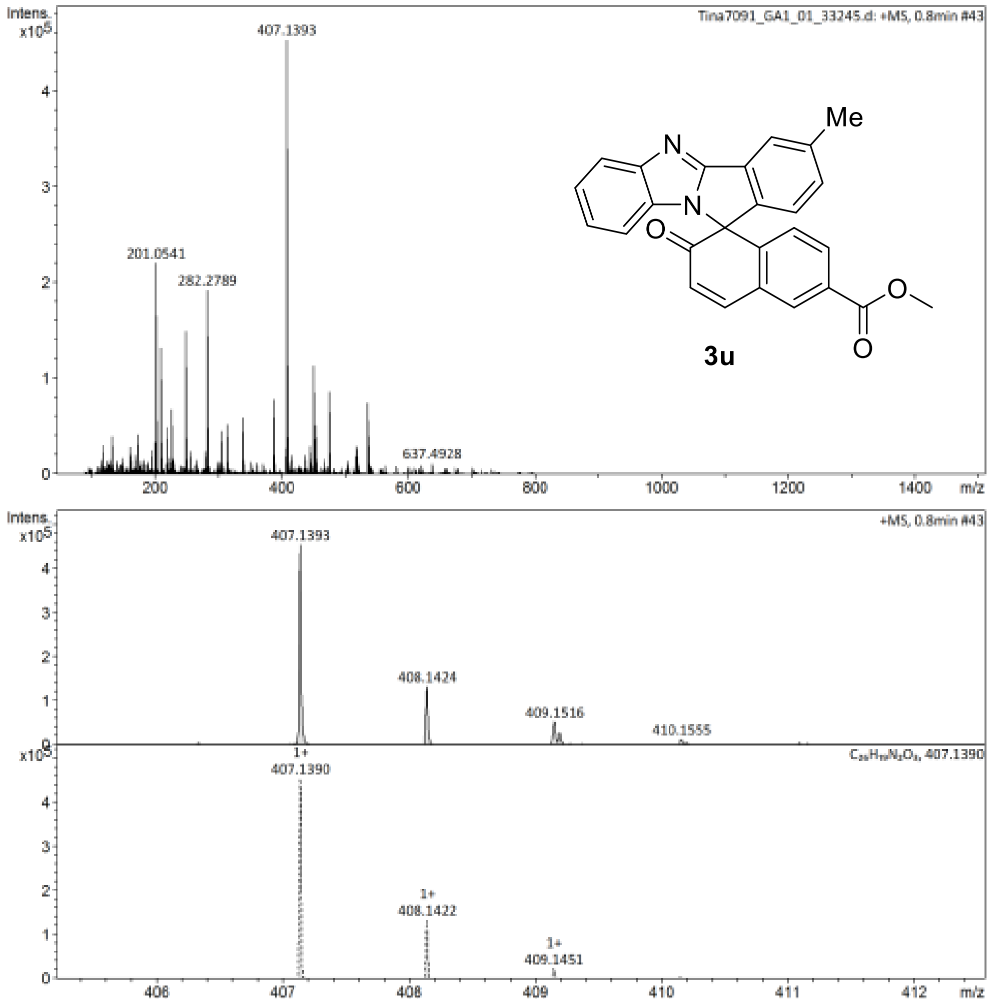

Display Report

Meas. m/z	#	Ion Formula	m/z	err [ppm]	mSigma	# Sigma	Score	rdb	e⁻ Conf	N-Rule	Adduct
407.1393	1	C26H19N2O3	407.1390	-0.7	34.1	1	100.00	18.5	even	ok	M+H

HRMS (ESI) of compound **3u**

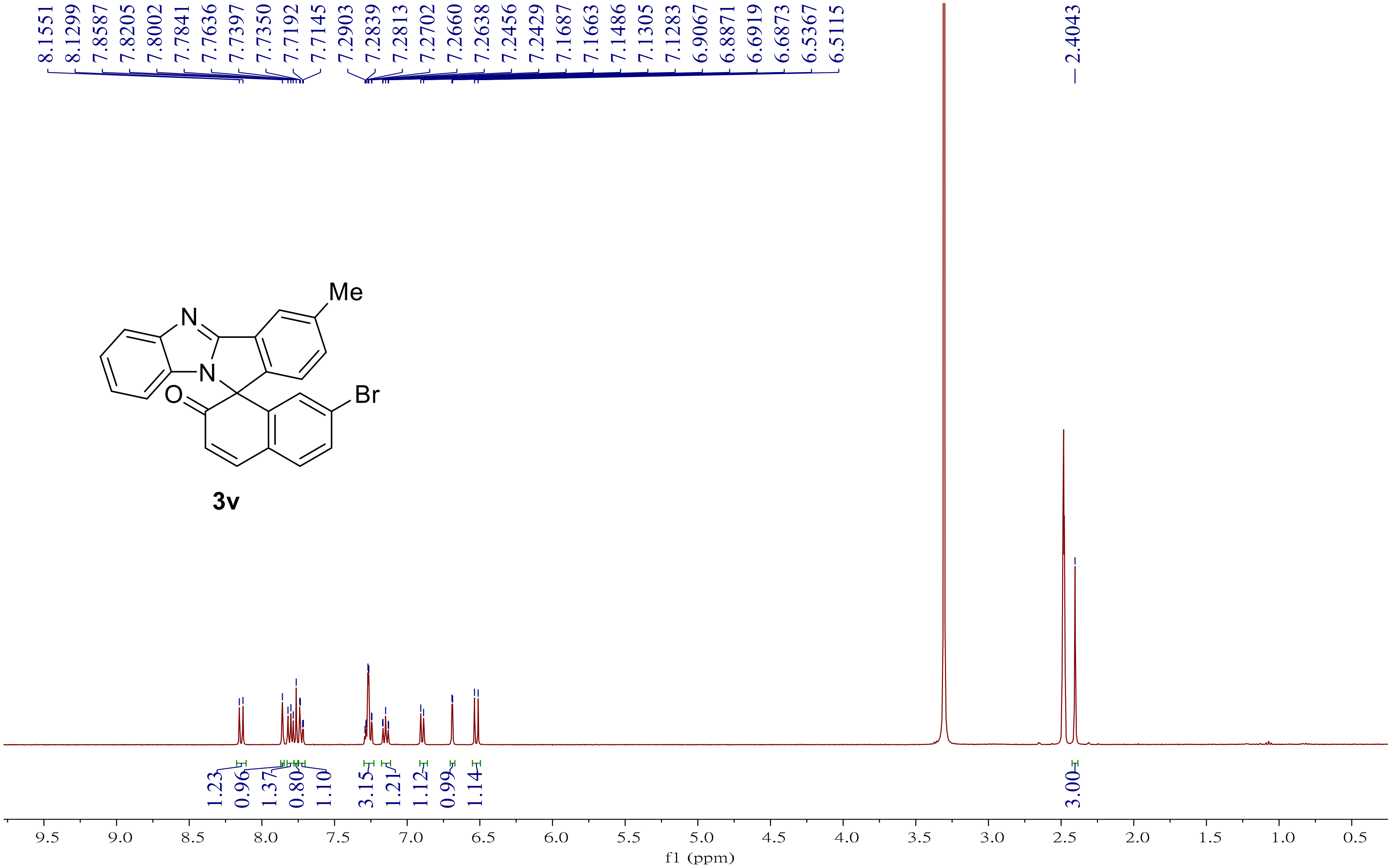

^{1}H NMR spectrum (400 MHz) of compound **3v** in DMSO-d_6.

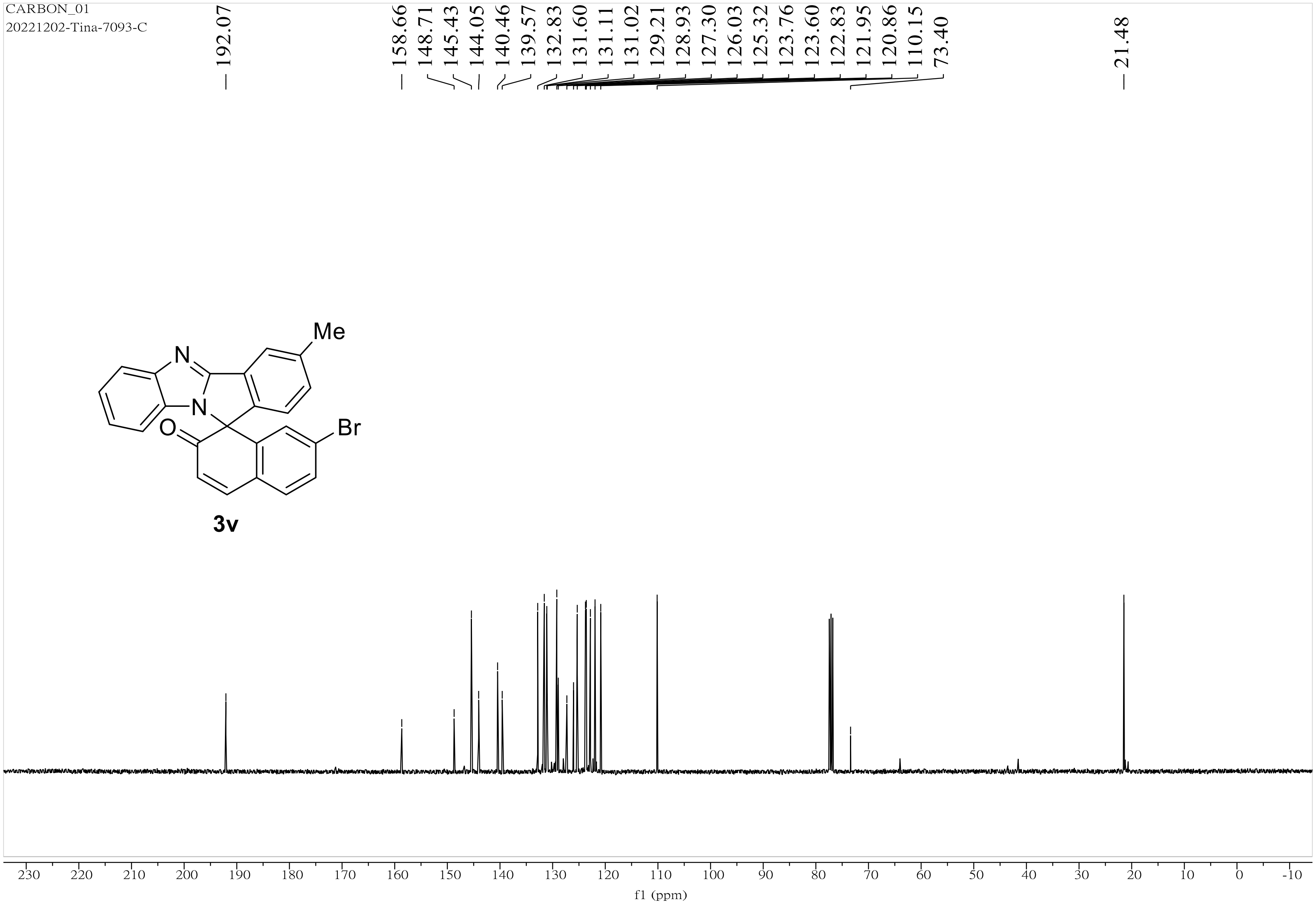

$^{13}C\{1H\}$ NMR spectrum (101 MHz) of compound **3v** in $CDCl_3$.

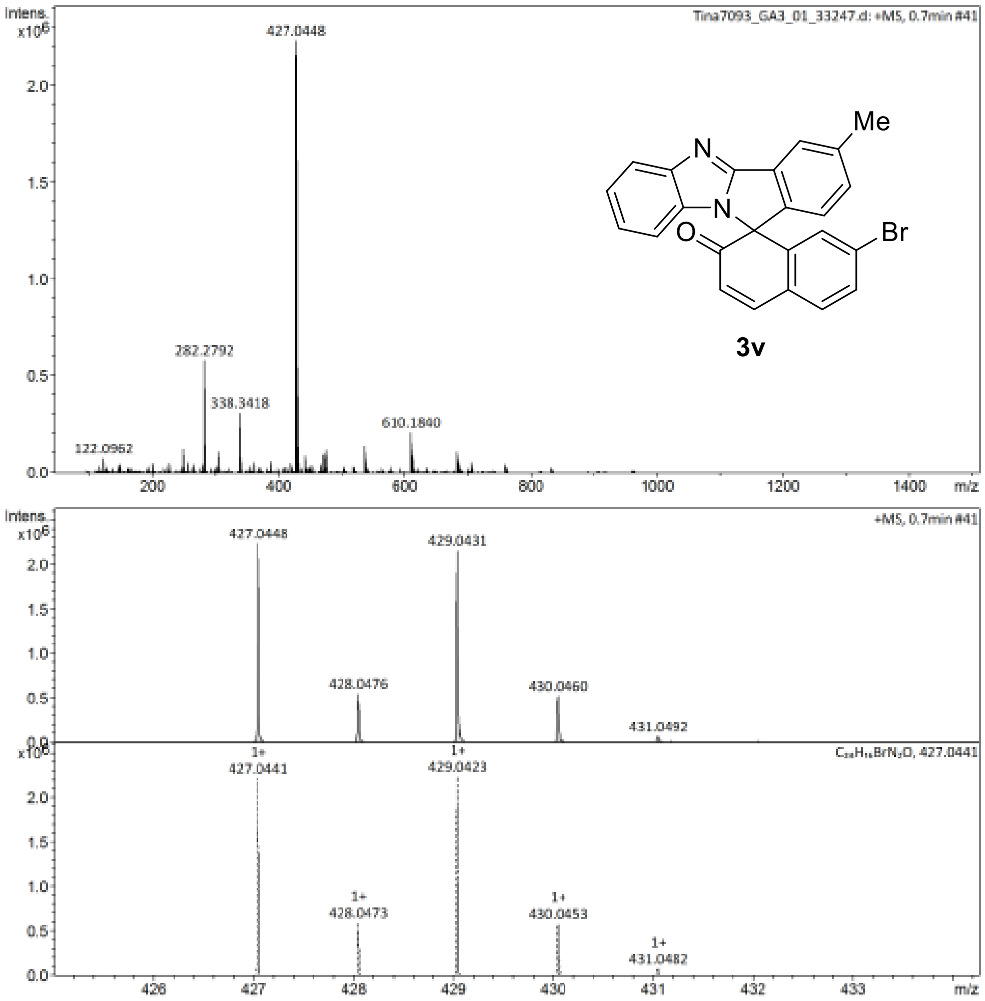

Display Report

Meas. m/z	#	Ion Formula	m/z	err [ppm]	mSigma	# Sigma	Score	rdb	e⁻ Conf	N-Rule	Adduct
427.0448	1	C24H16BrN2O	427.0441	1.8	22.6	1	100.00	17.5	even	ok	M+H

HRMS (ESI) of compound **3v**

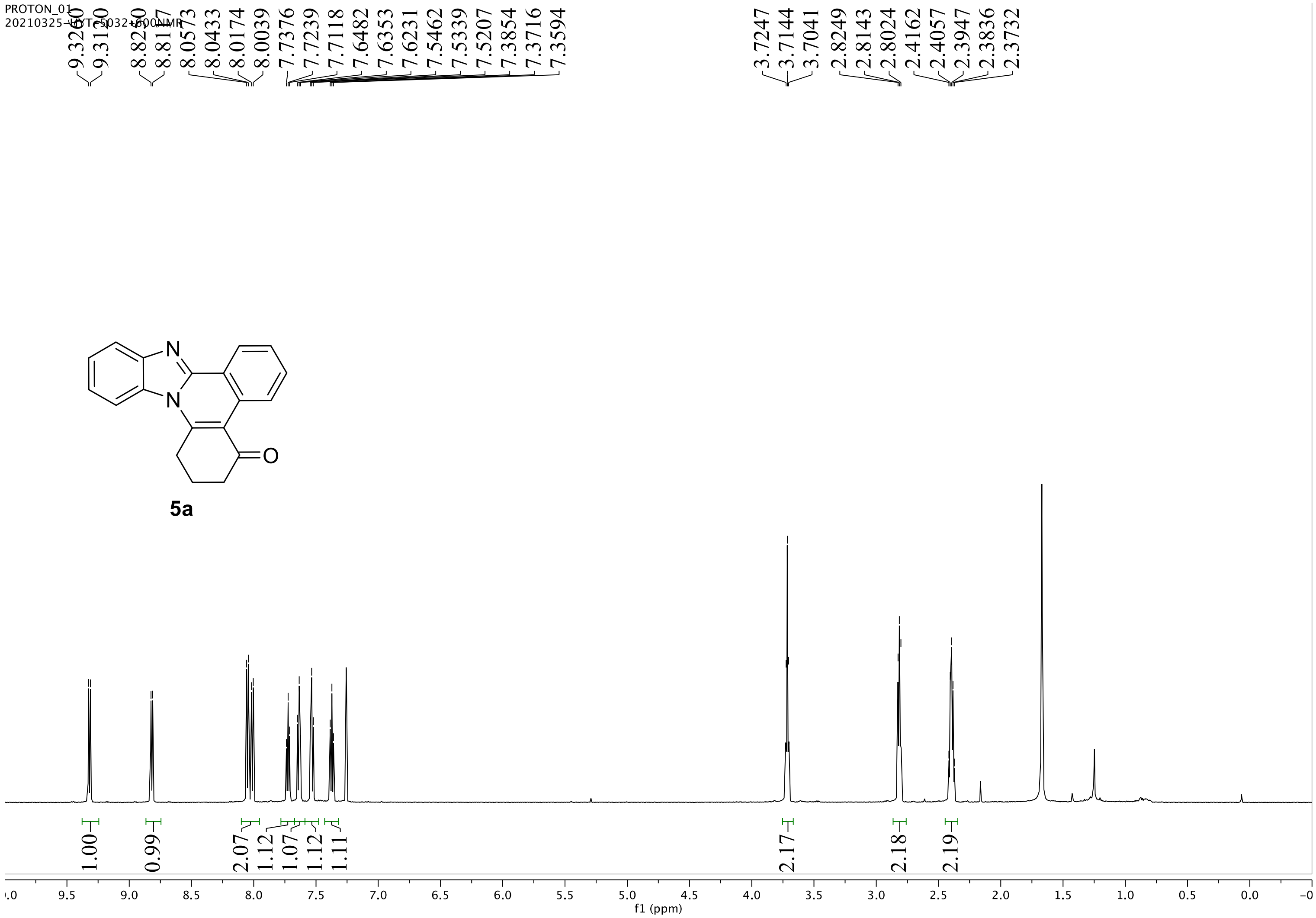

^{1}H NMR spectrum (600 MHz) of compound **5a** in $CDCl_3$.

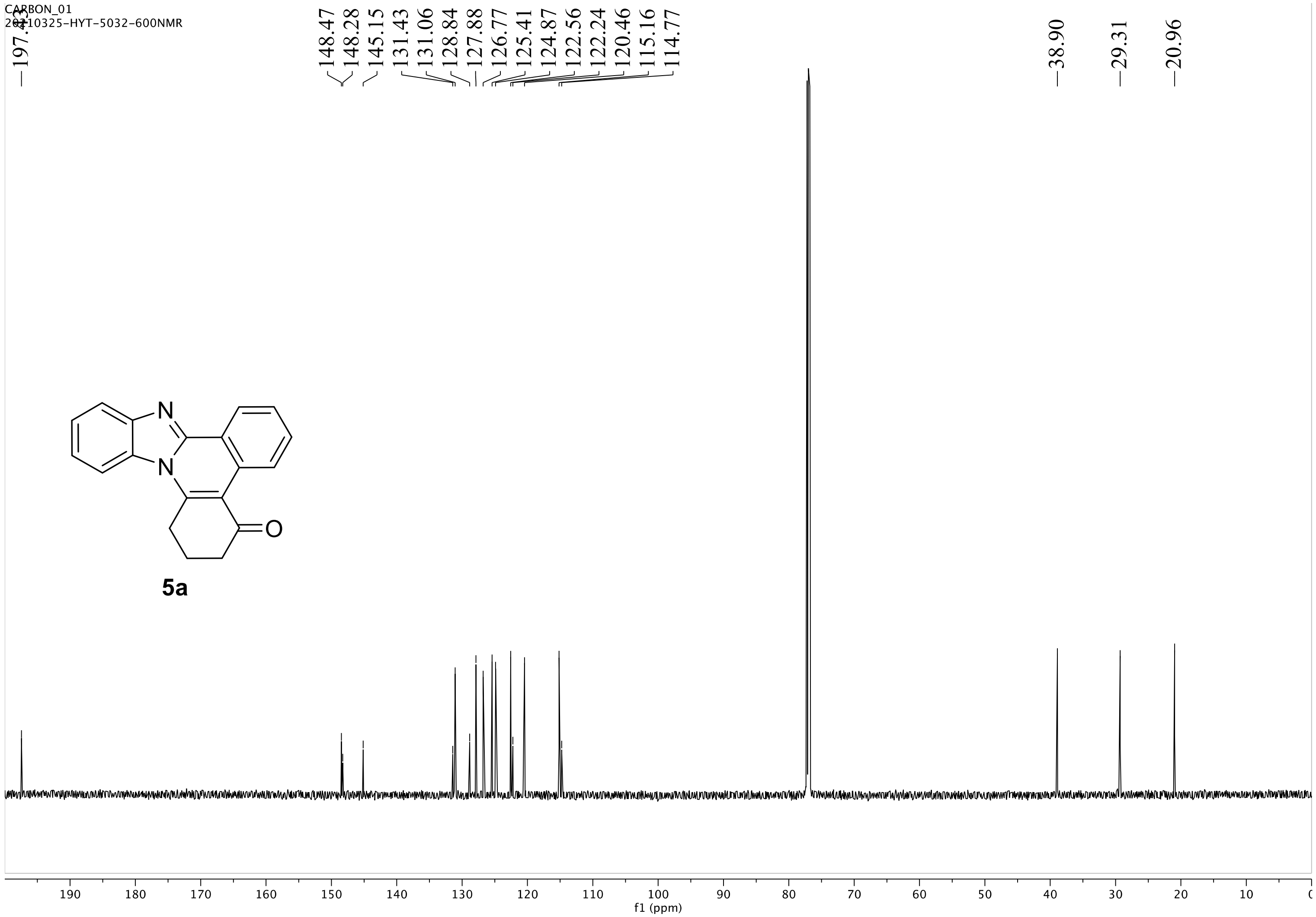

$^{13}C\{1H\}$ NMR spectrum (150 MHz) of compound **5a** in $CDCl_3$.

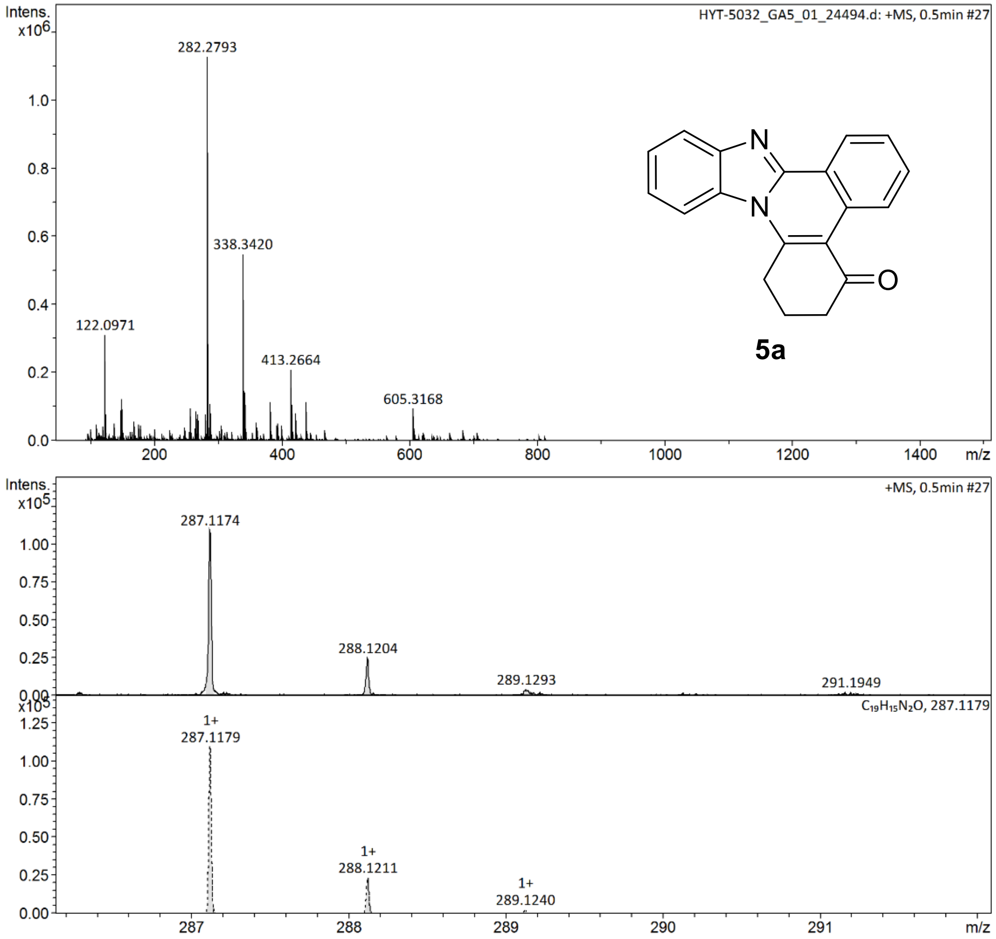

Display Report

Meas. m/z	#	Ion Formula	m/z	err [ppm]	mSigma	# Sigma	Score	rdb	e⁻ Conf	N-Rule	Adduct
287.1174	1	C19H15N2O	287.1179	1.7	10.9	1	100.00	13.5	even	ok	M+H

HRMS (ESI) of compound **5a**

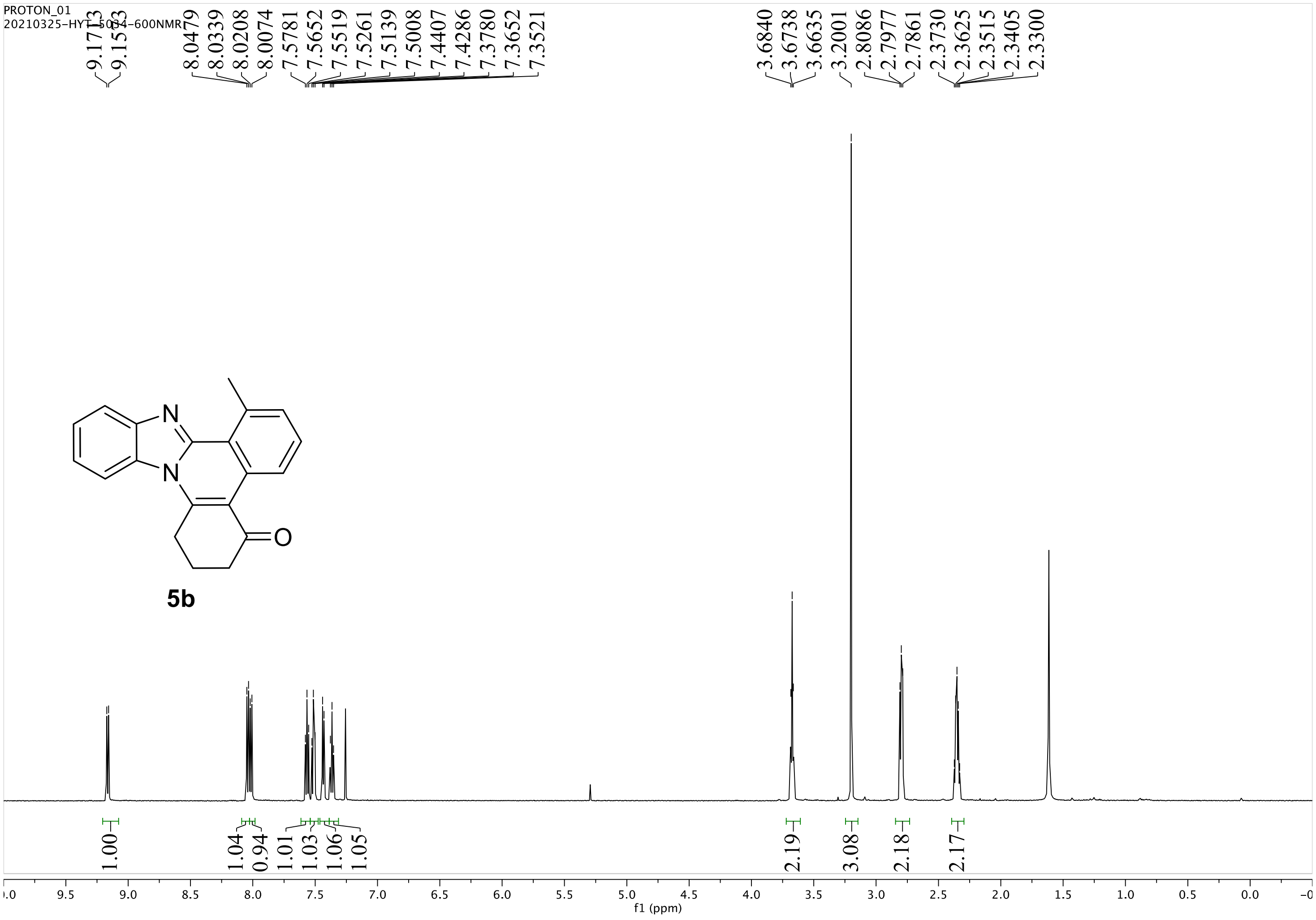

^{1}H NMR spectrum (600 MHz) of compound **5b** in $CDCl_3$.

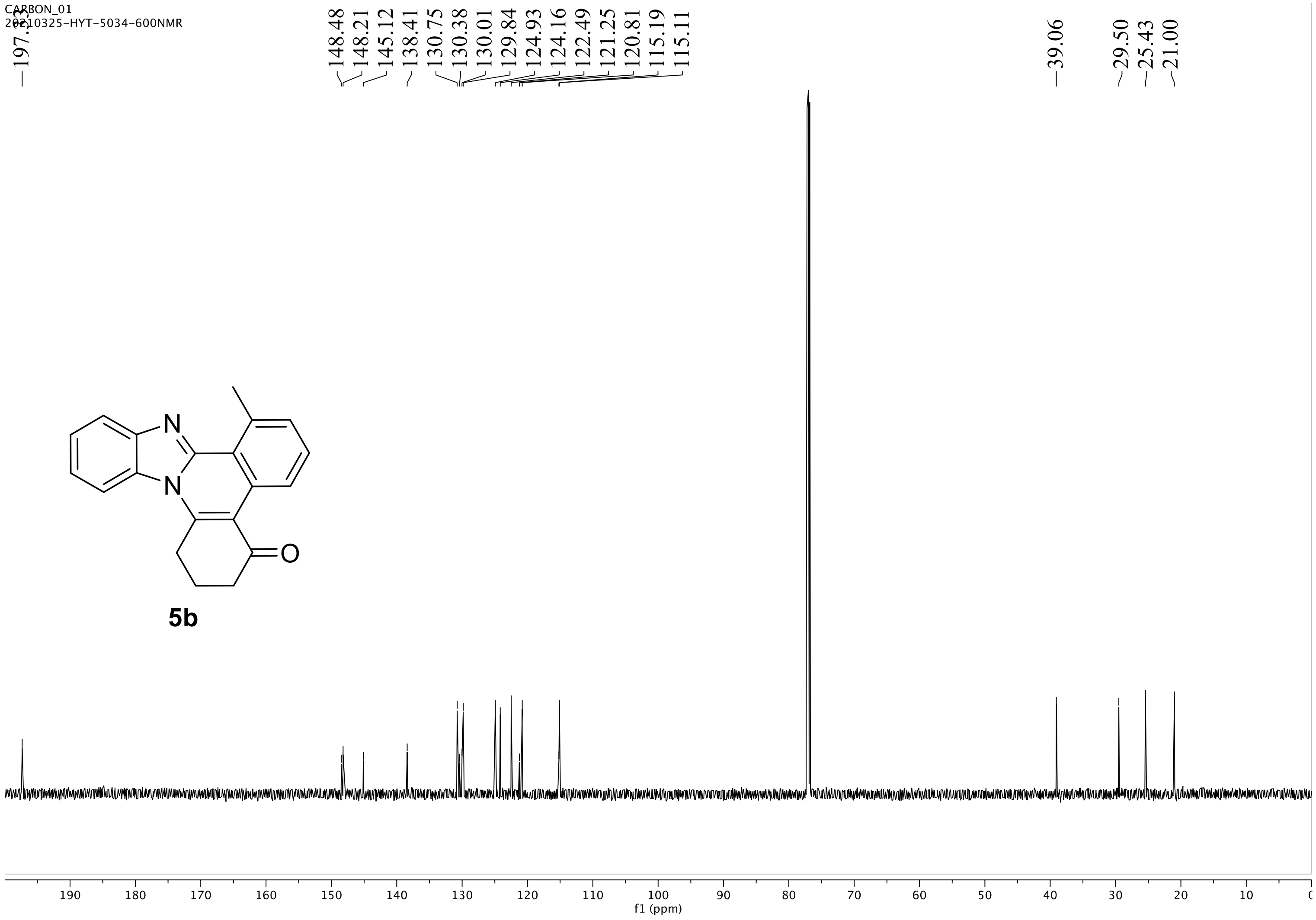

$^{13}C\{1H\}$ NMR spectrum (150 MHz) of compound **5b** in $CDCl_3$.

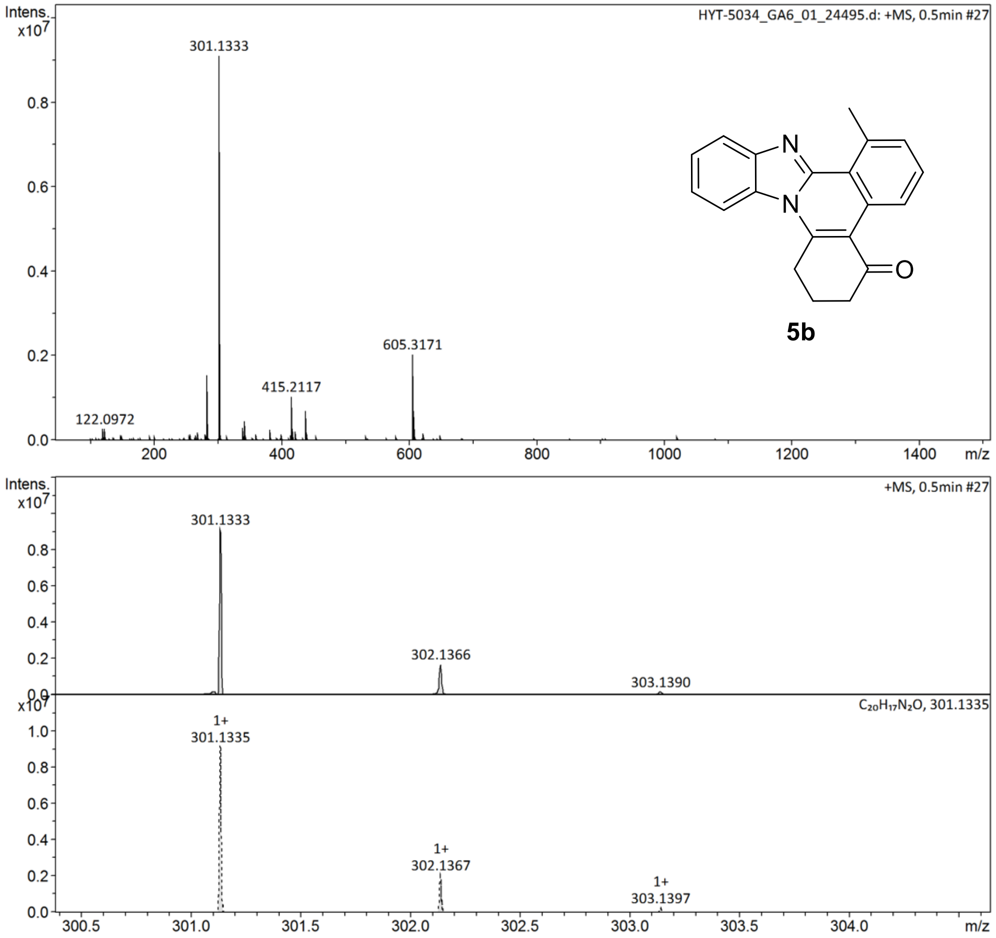

Display Report

Meas. m/z	#	Ion Formula	m/z	err [ppm]	mSigma	# Sigma	Score	rdb	e⁻ Conf	N-Rule	Adduct
301.1333	1	C20H17N2O	301.1335	-0.8	24.2	1	100.00	13.5	even	ok	M+H

HRMS (ESI) of compound **5b**

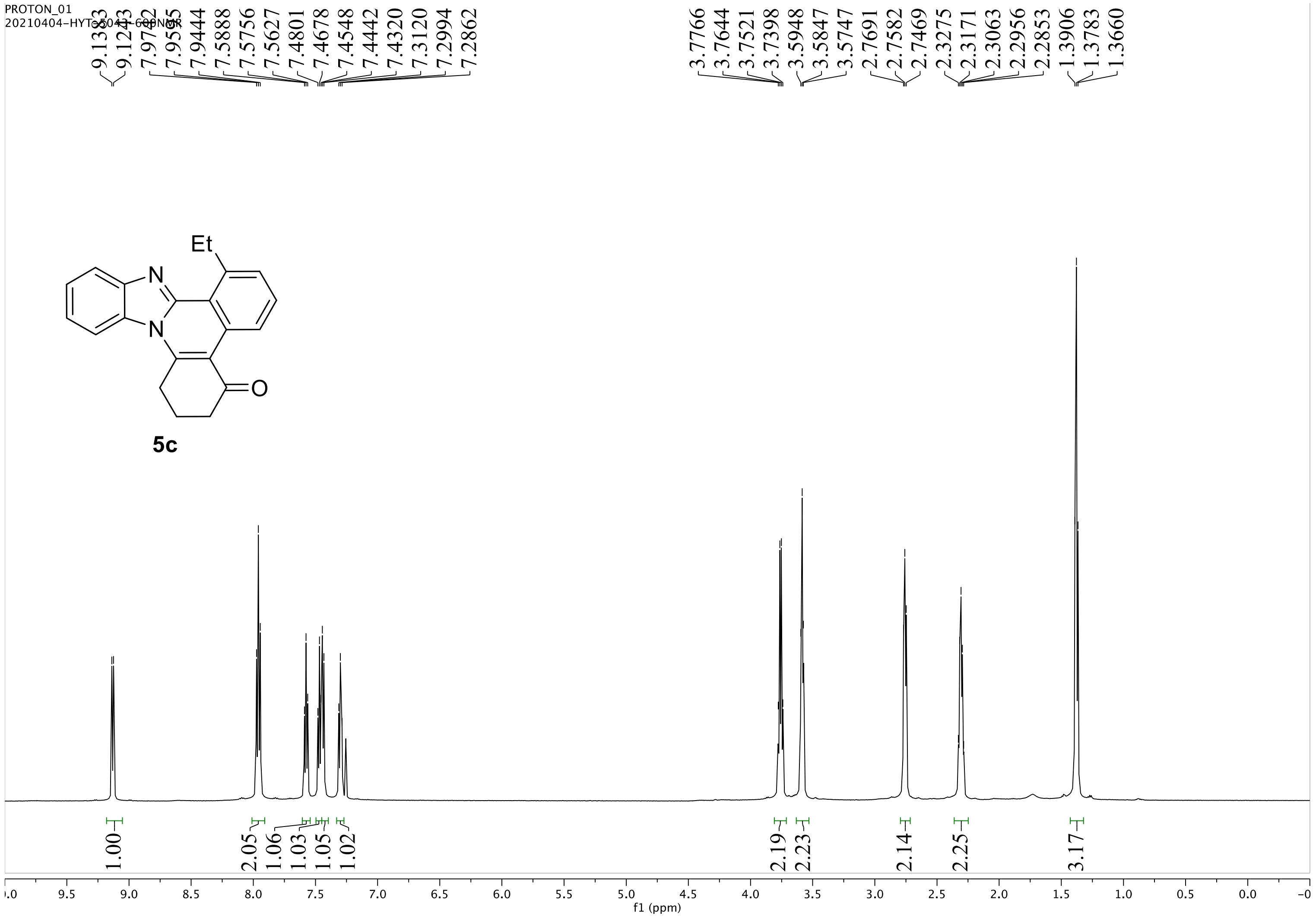

^{1}H NMR spectrum (600 MHz) of compound **5c** in $CDCl_3$

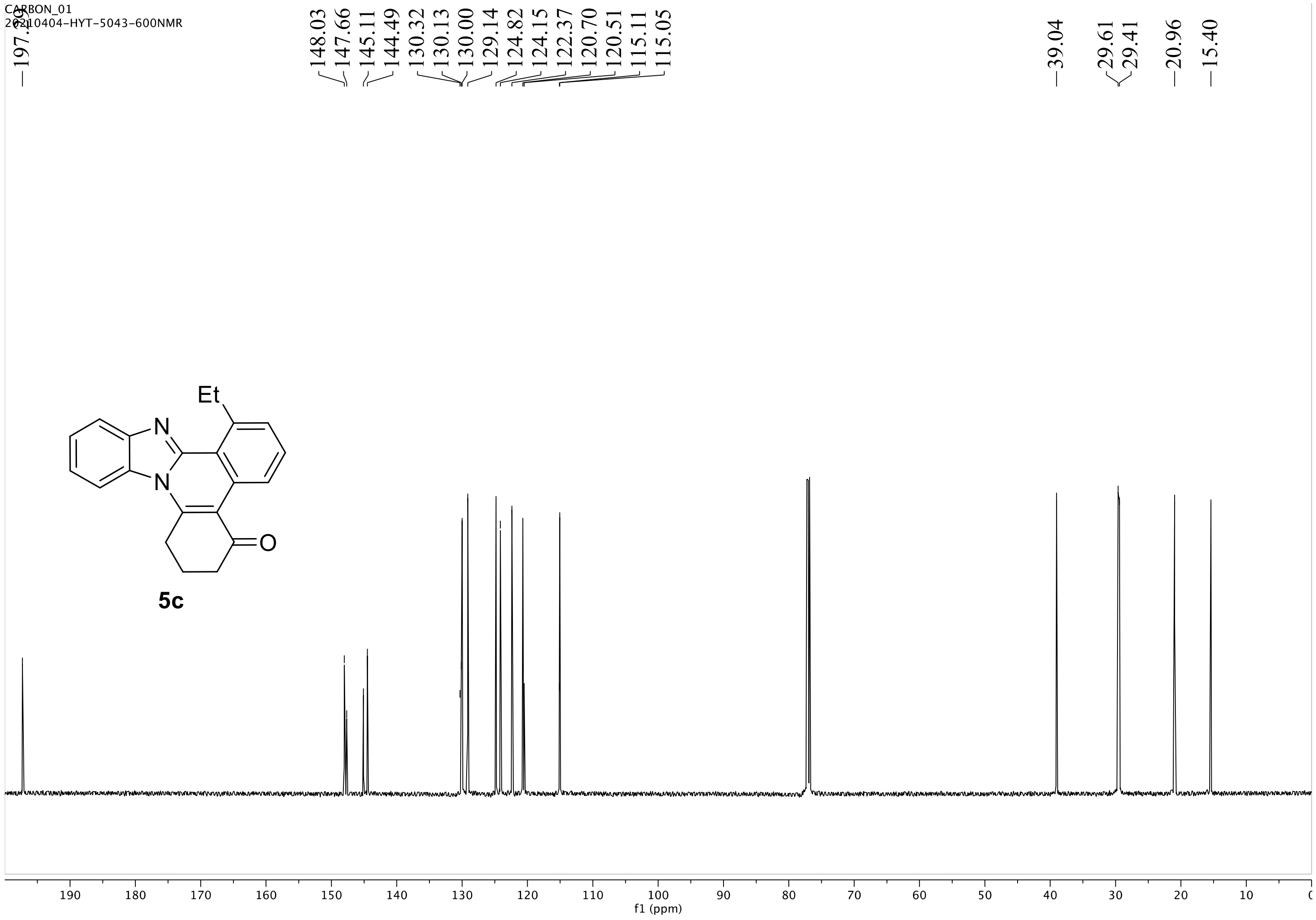

$^{13}C\{^1H\}$ NMR spectrum (150 MHz) of compound **5c** in $CDCl_3$

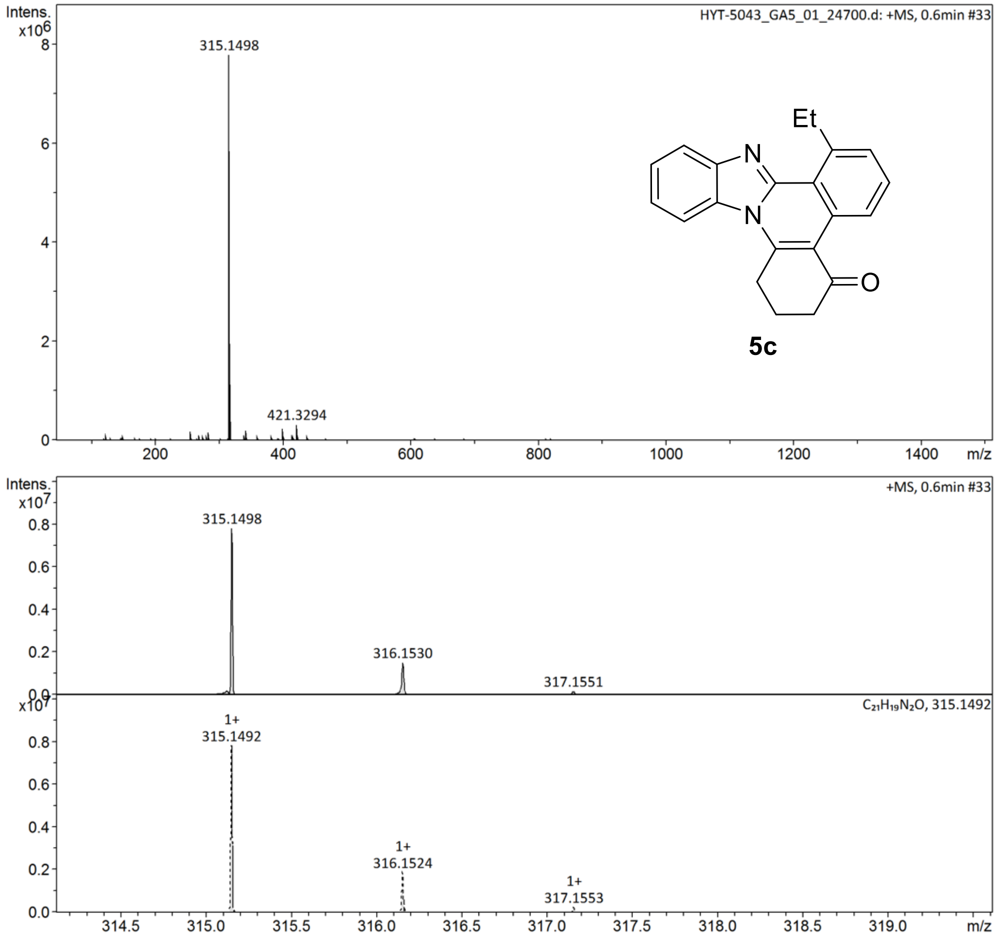

Display Report

Meas. m/z	#	Ion Formula	m/z	err [ppm]	mSigma	# Sigma	Score	rdb	e⁻ Conf	N-Rule	Adduct
315.1498	1	C21H19N2O	315.1492	-1.8	25.9	1	100.00	13.5	even	ok	M+H

HRMS (ESI) of compound **5c**

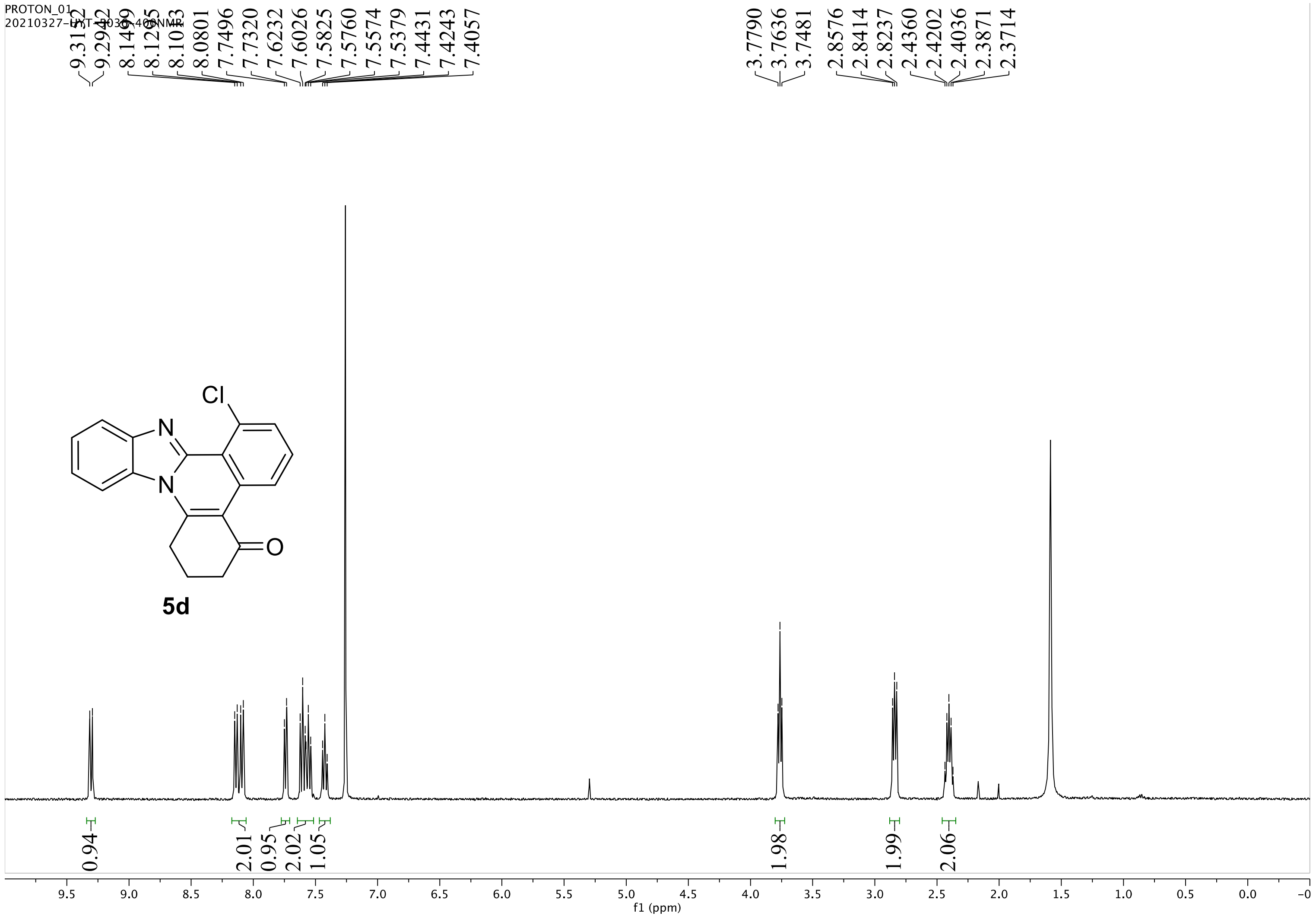

^{1}H NMR spectrum (400 MHz) of compound **5d** in $CDCl_3$

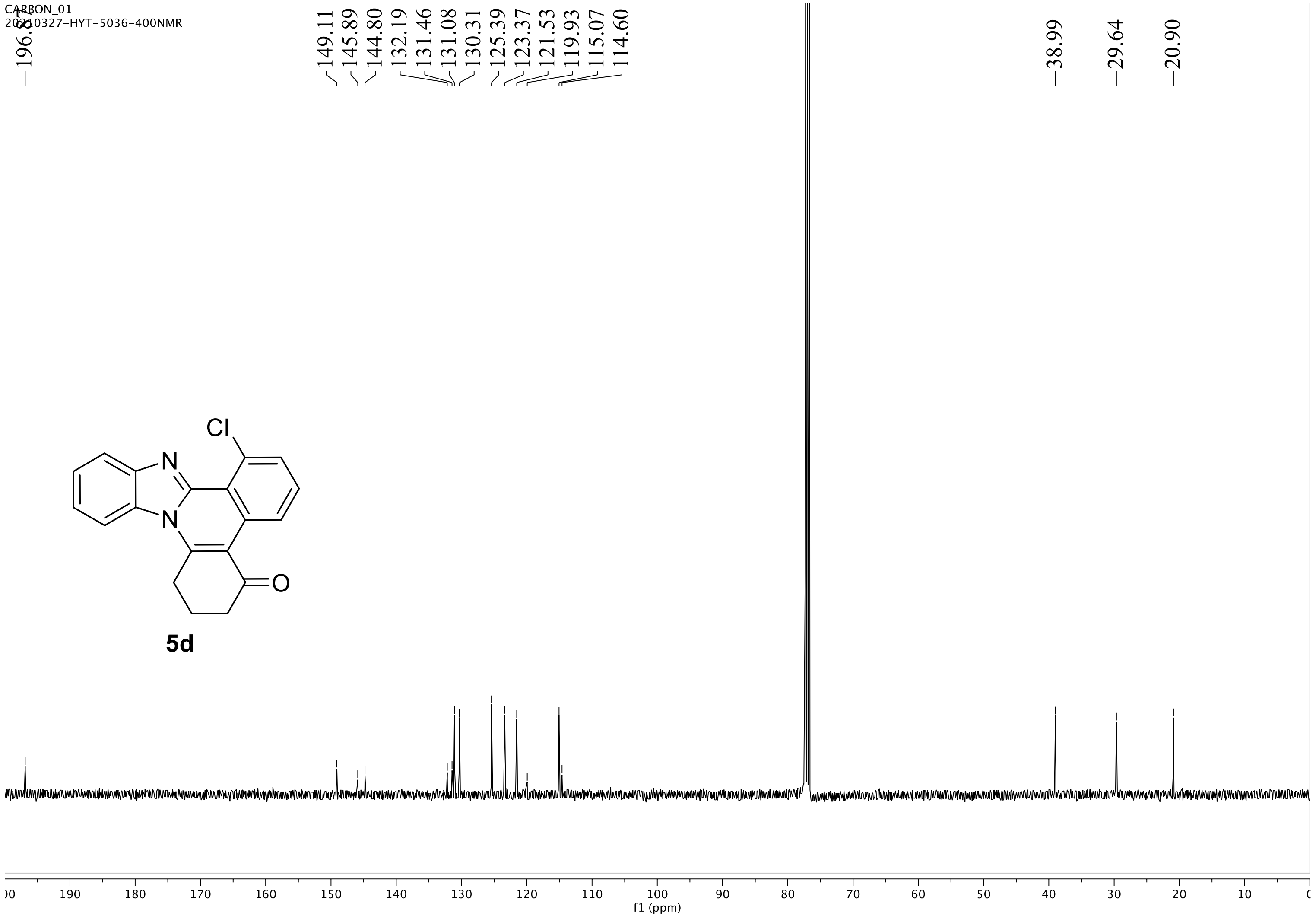

$^{13}C\{1H\}$ NMR spectrum (100 MHz) of compound **5d** in $CDCl_3$

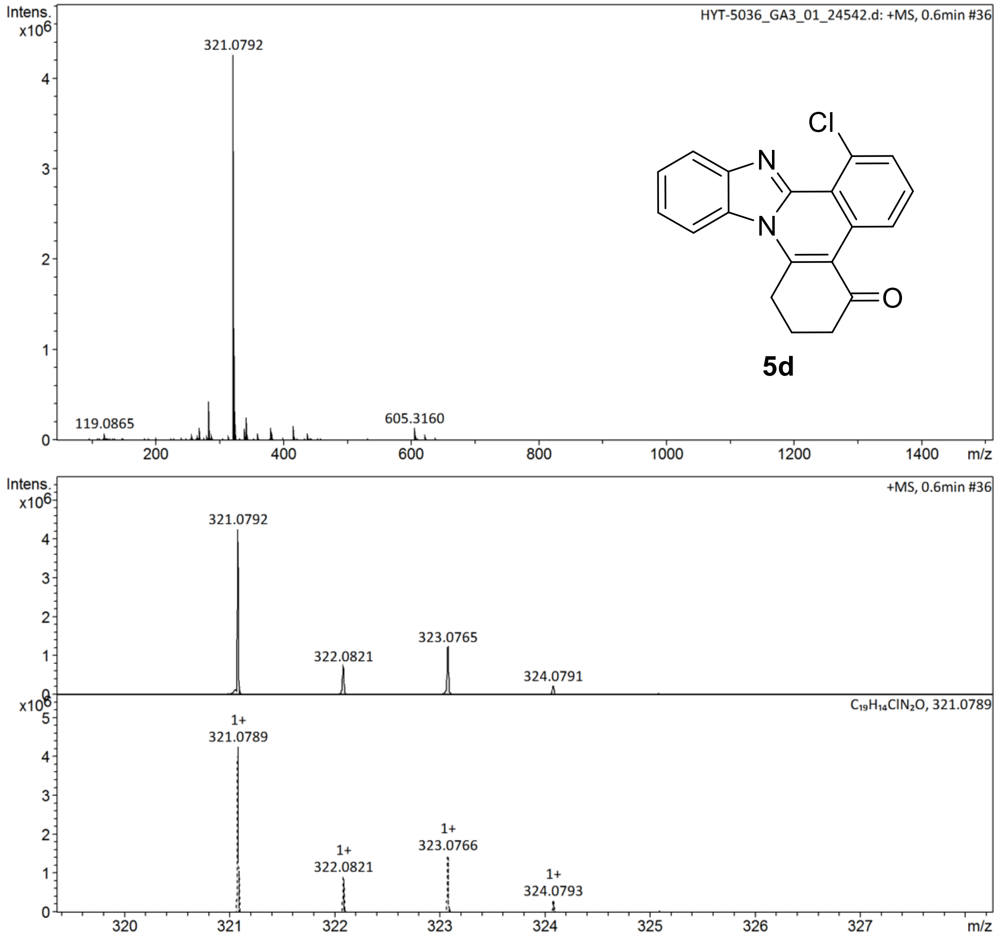

Display Report

Meas. m/z	#	Ion Formula	m/z	err [ppm]	mSigma	# Sigma	Score	rdb	e⁻ Conf	N-Rule	Adduct
321.0792	1	C19H14ClN2O	321.0789	-0.7	31.6	1	100.00	13.5	even	ok	M+H

HRMS (ESI) of compound **5d**

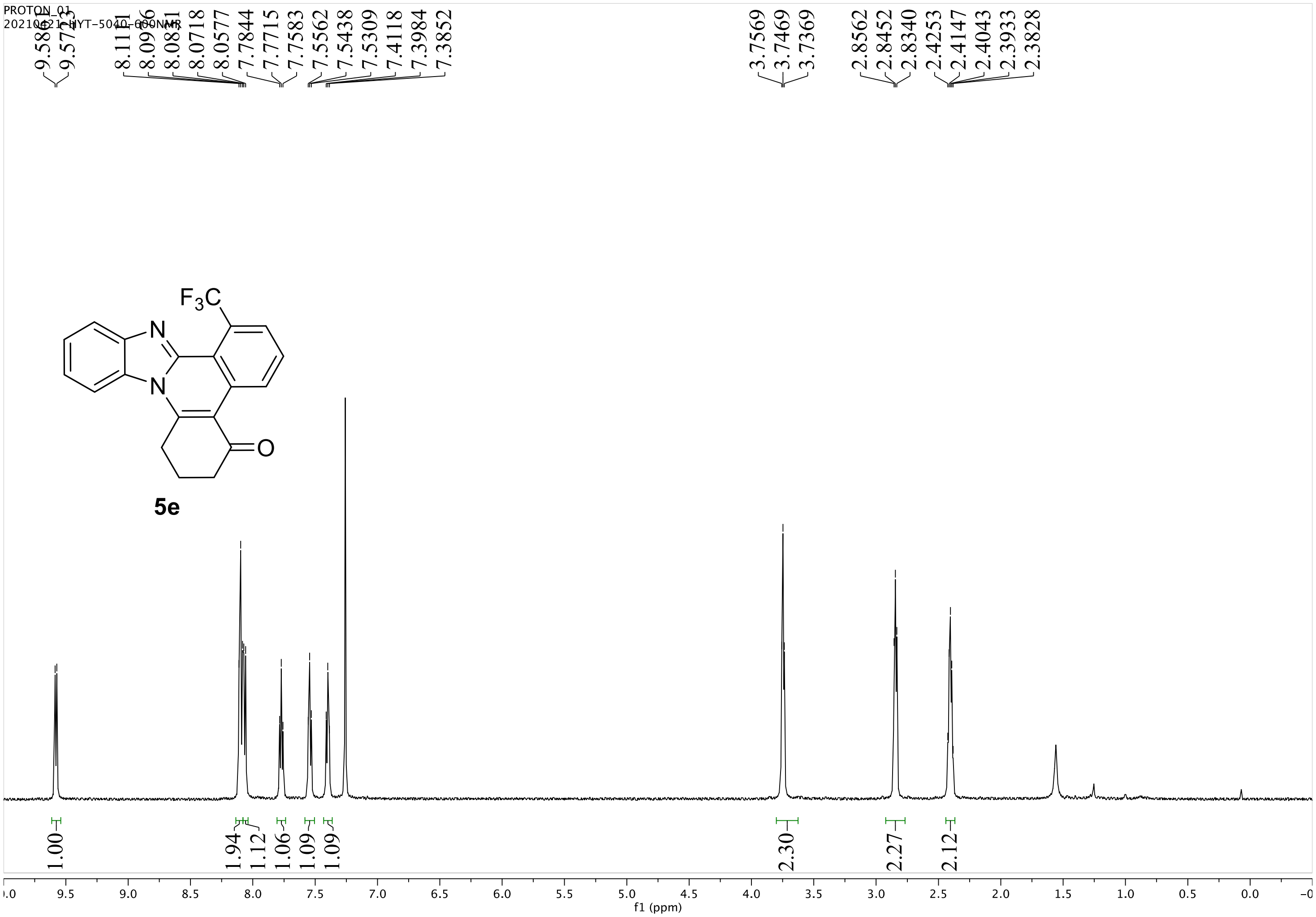

^{1}H NMR spectrum (600 MHz) of compound **5e** in $CDCl_3$

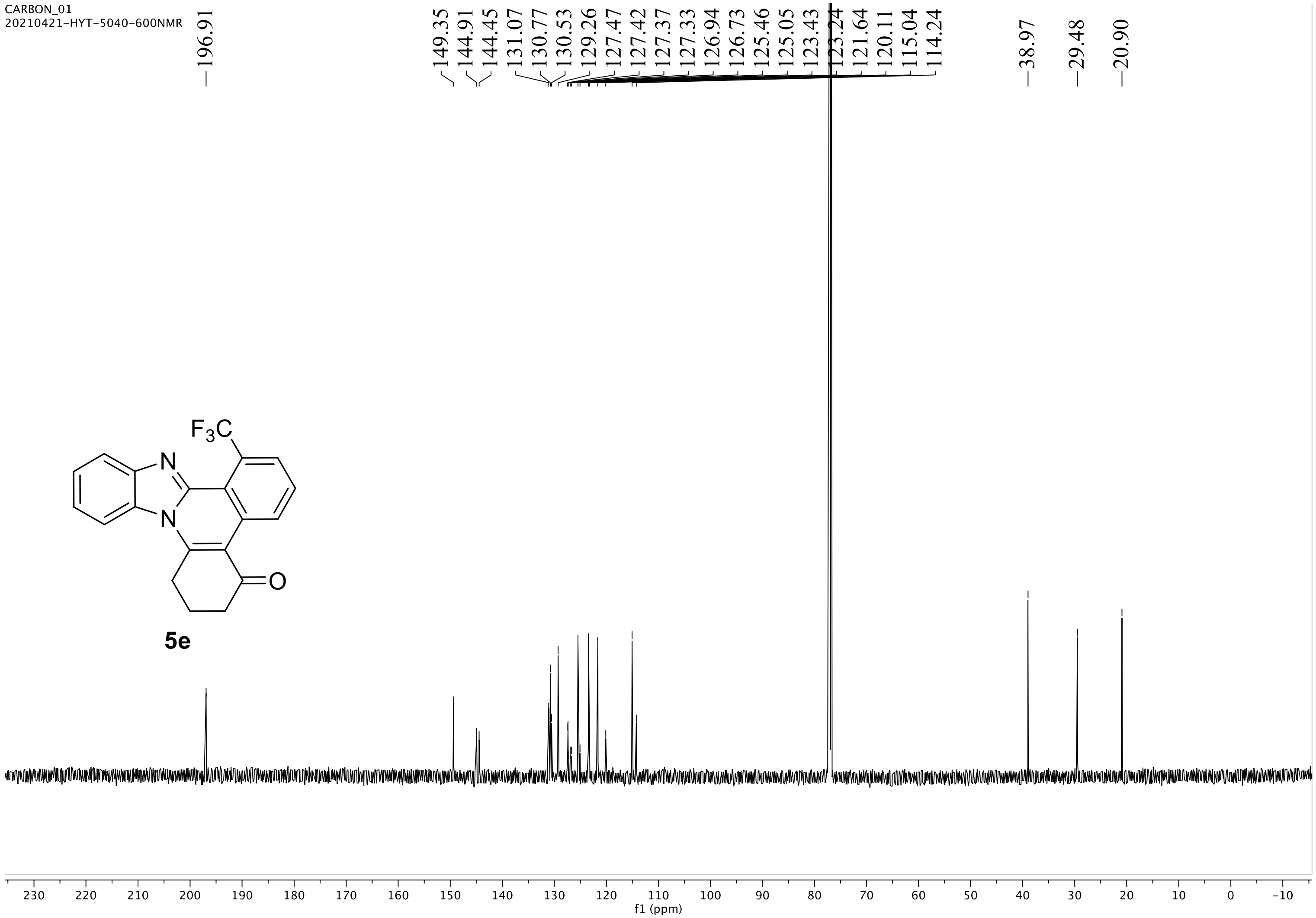

^{13}C{1H} NMR spectrum (150 MHz) of compound **5e** in $CDCl_3$

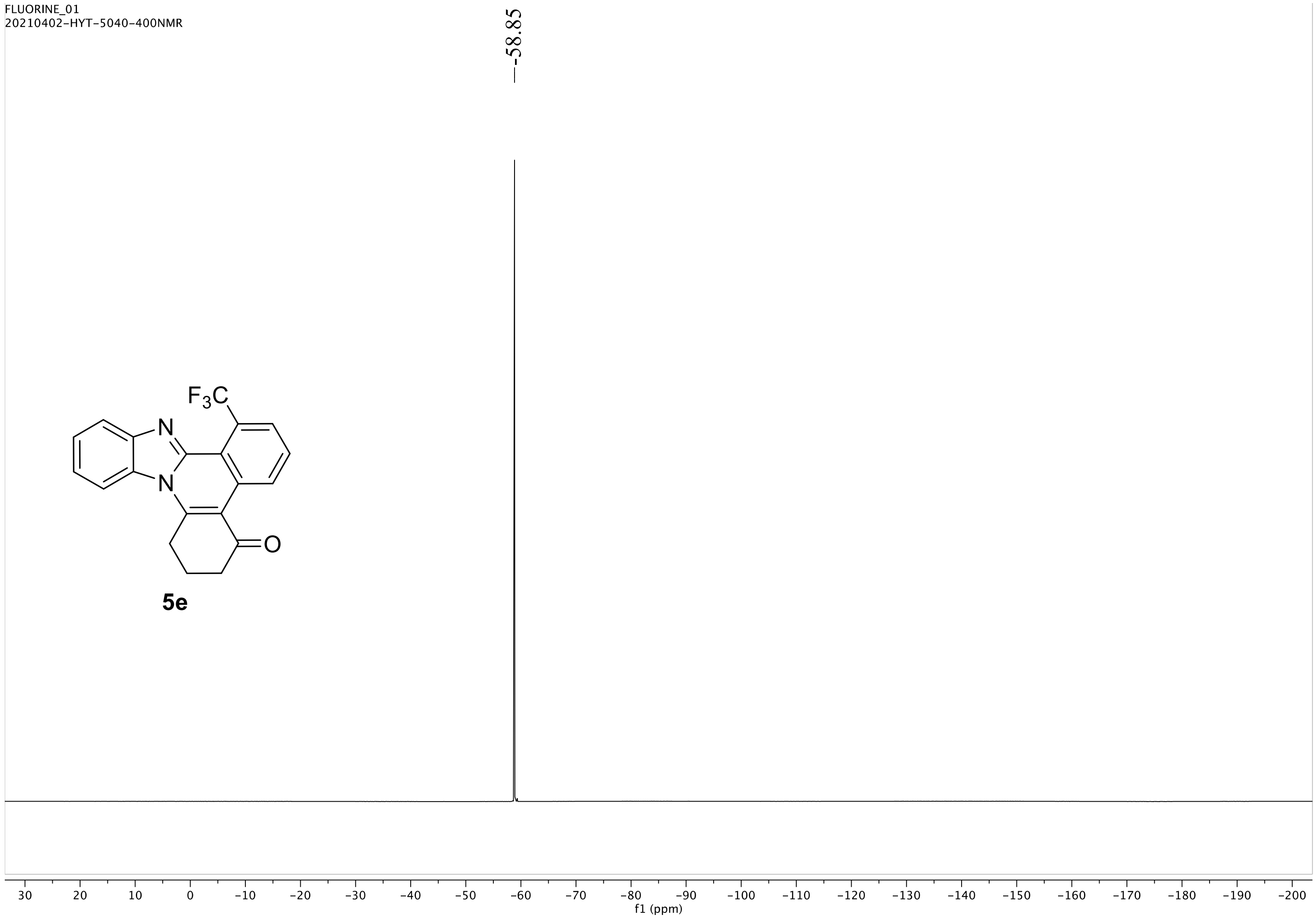

^{19}F NMR spectrum (376 MHz) of compound **5e** in $CDCl_3$

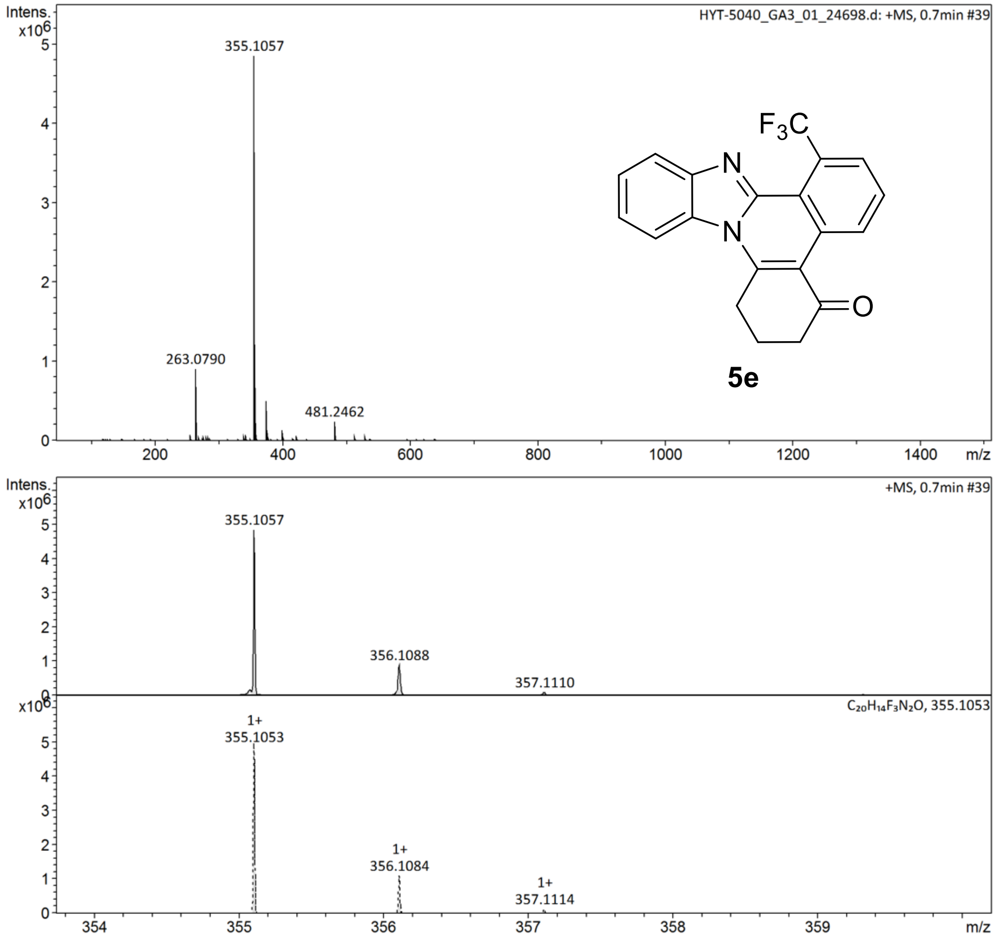

Display Report

Meas. m/z	#	Ion Formula	m/z	err [ppm]	mSigma	# Sigma	Score	rdb	e⁻ Conf	N-Rule	Adduct
355.1057	1	C20H14F3N2O	355.1053	1.2	25.0	1	100.00	13.5	even	ok	M+H

HRMS (ESI) of compound **5e**

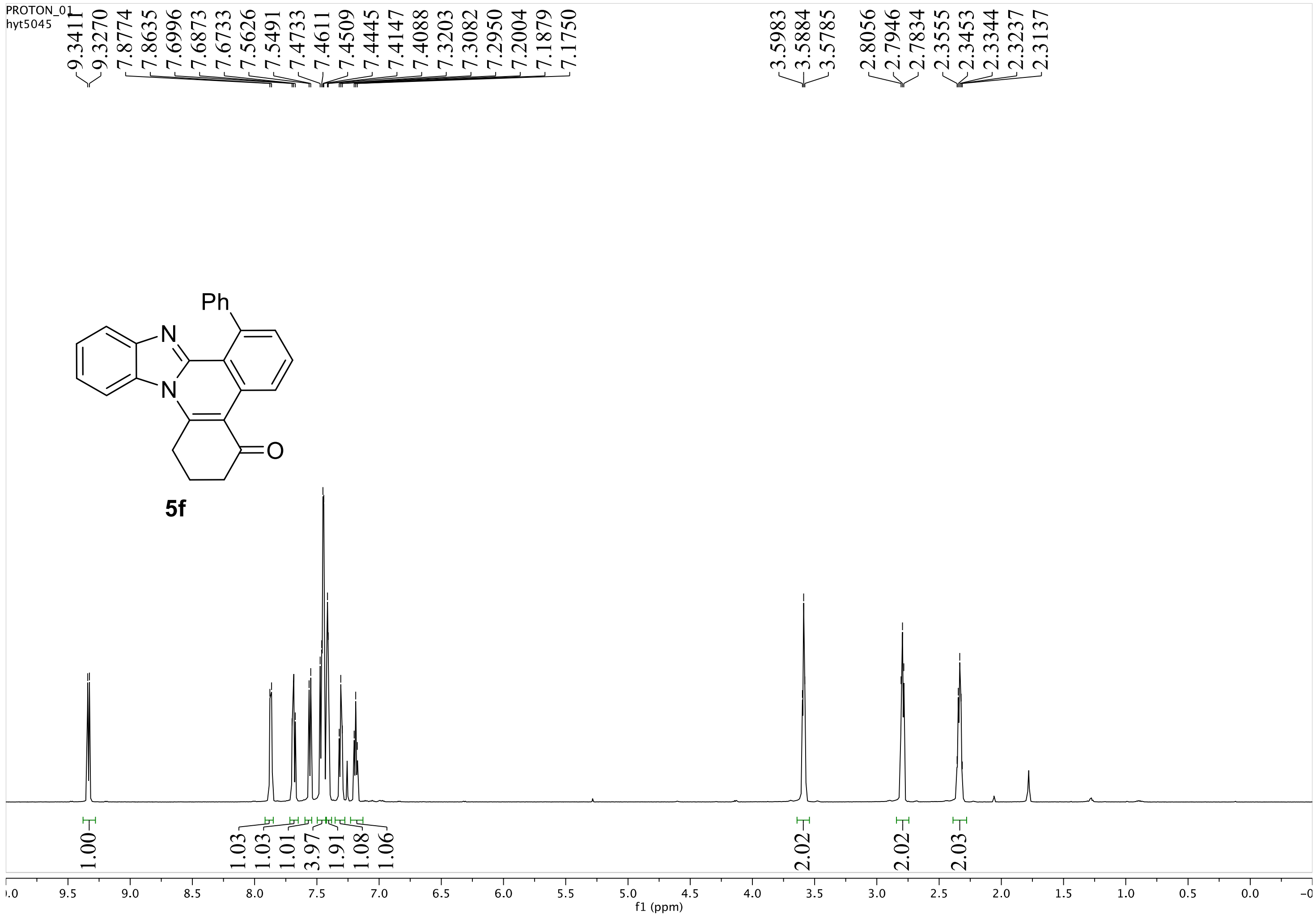

^{1}H NMR spectrum (600 MHz) of compound **5f** in $CDCl_3$

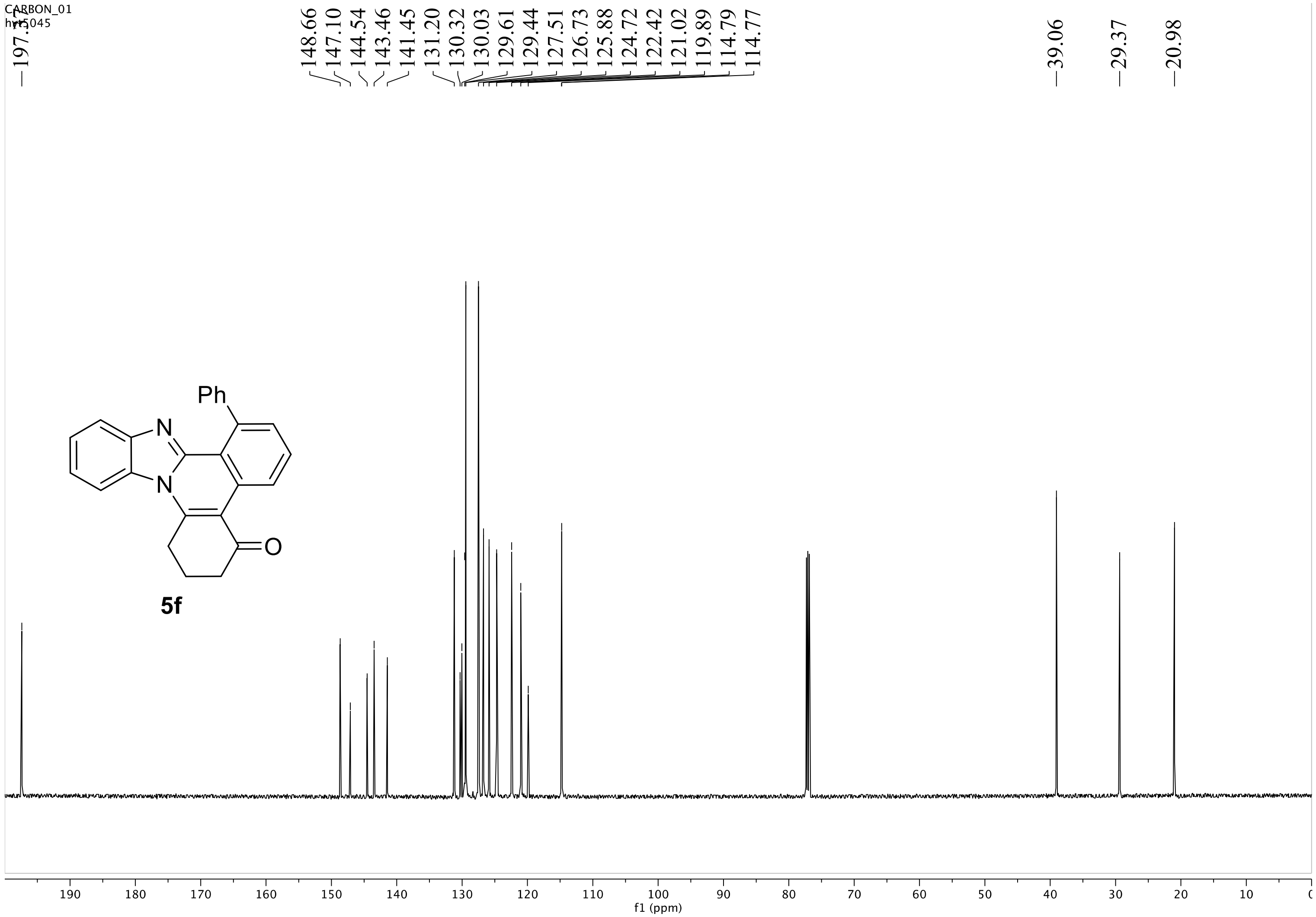

$^{13}C\{1H\}$ NMR spectrum (150 MHz) of compound **5f** in $CDCl_3$

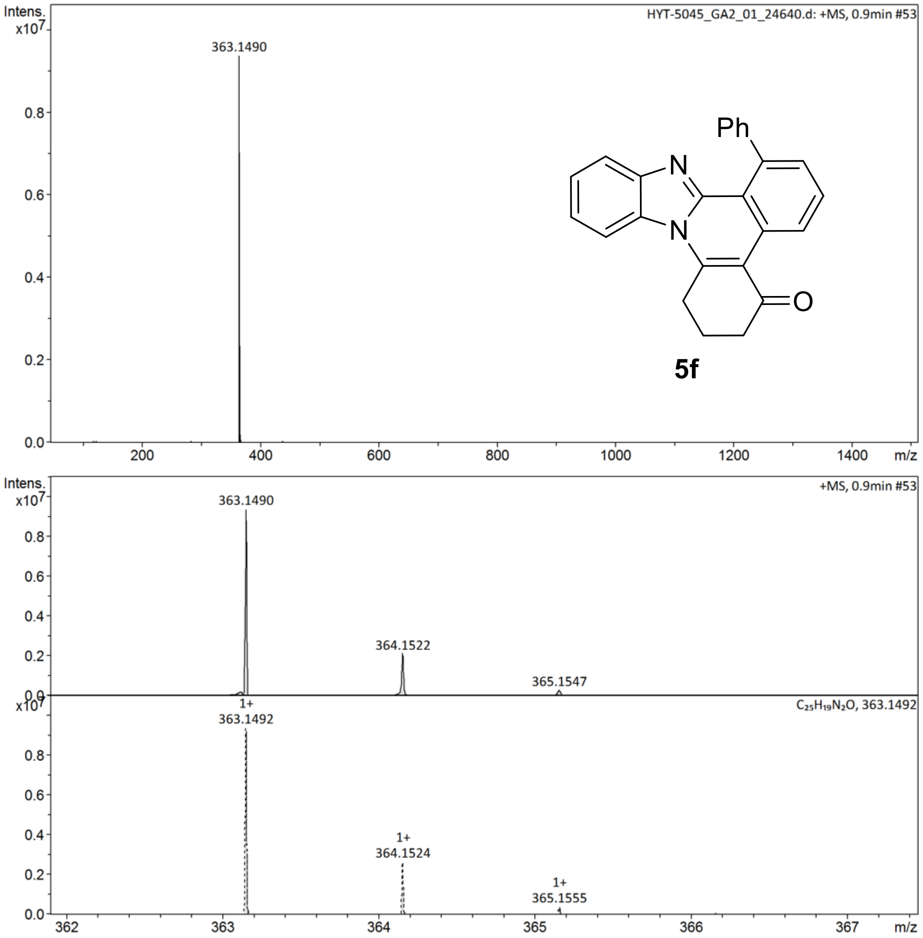

Display Report

Meas. m/z	#	Ion Formula	m/z	err [ppm]	mSigma	# Sigma	Score	rdb	e⁻ Conf	N-Rule	Adduct
363.1490	1	C25H19N2O	363.1492	0.6	29.8	1	100.00	17.5	even	ok	M+H

HRMS (ESI) of compound **5f**

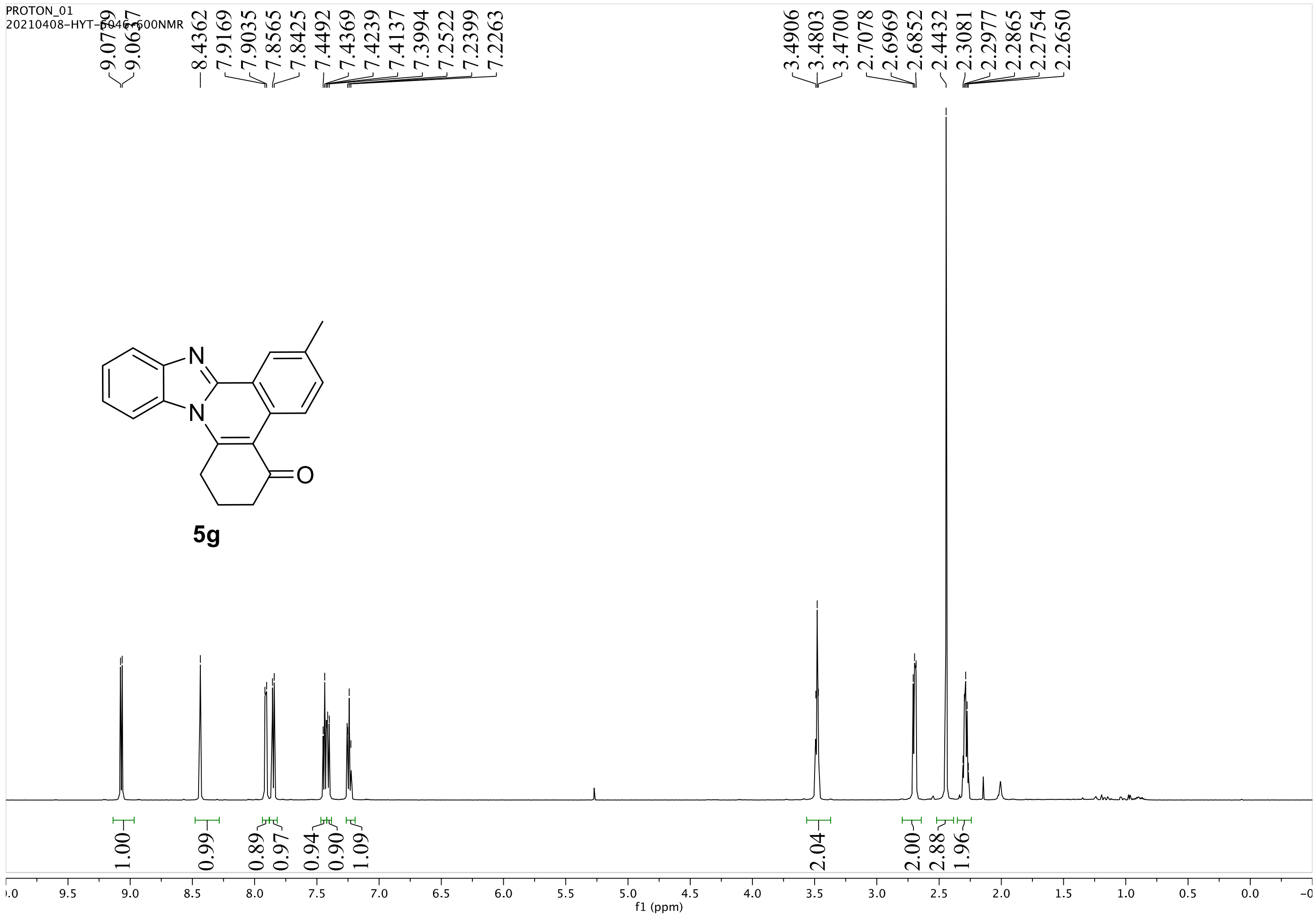

^{1}H NMR spectrum (600 MHz) of compound **5g** in $CDCl_3$

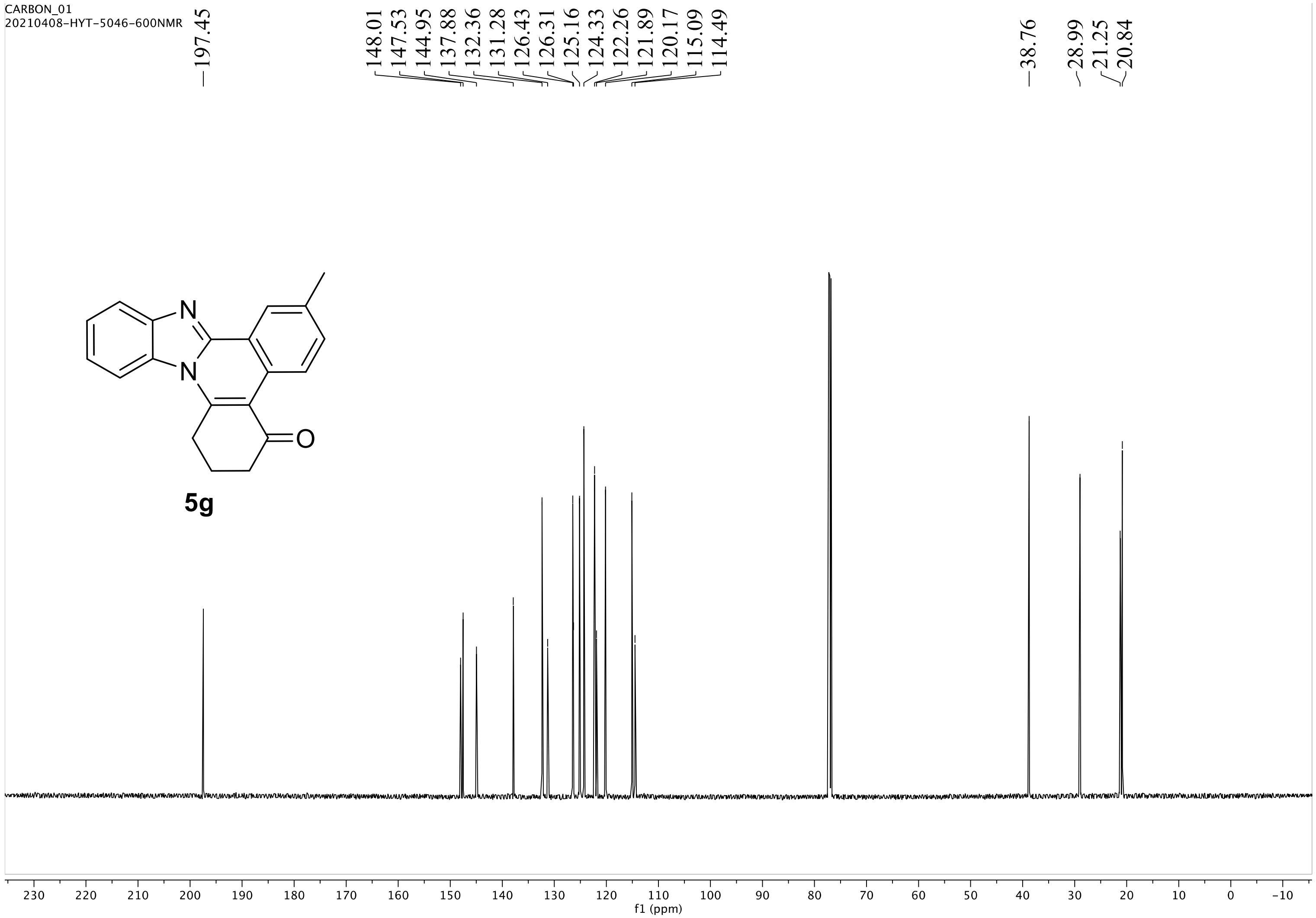

^{13}C{1H} NMR spectrum (150 MHz) of compound **5g** in $CDCl_3$

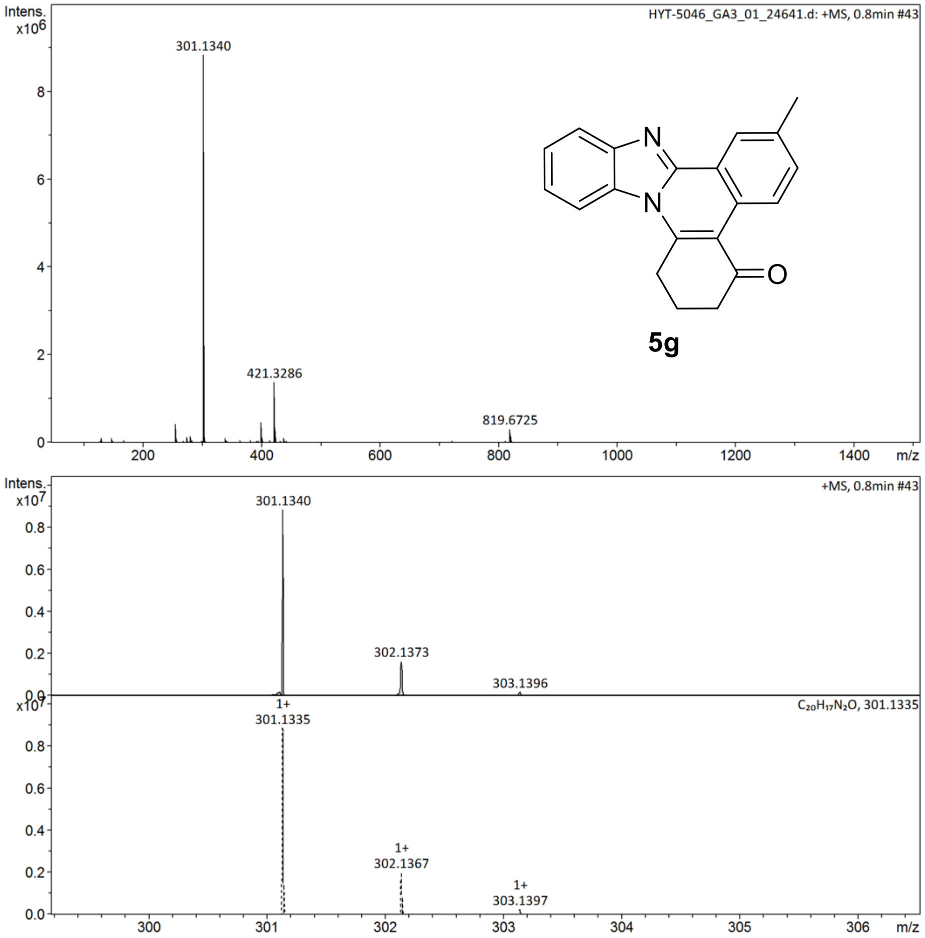

Display Report

Meas. m/z	#	Ion Formula	m/z	err [ppm]	mSigma	# Sigma	Score	rdb	e¯ Conf	N-Rule	Adduct
301.1340	1	C20H17N2O	301.1335	1.5	24.3	1	100.00	13.5	even	ok	M+H

HRMS (ESI) of compound **5g**

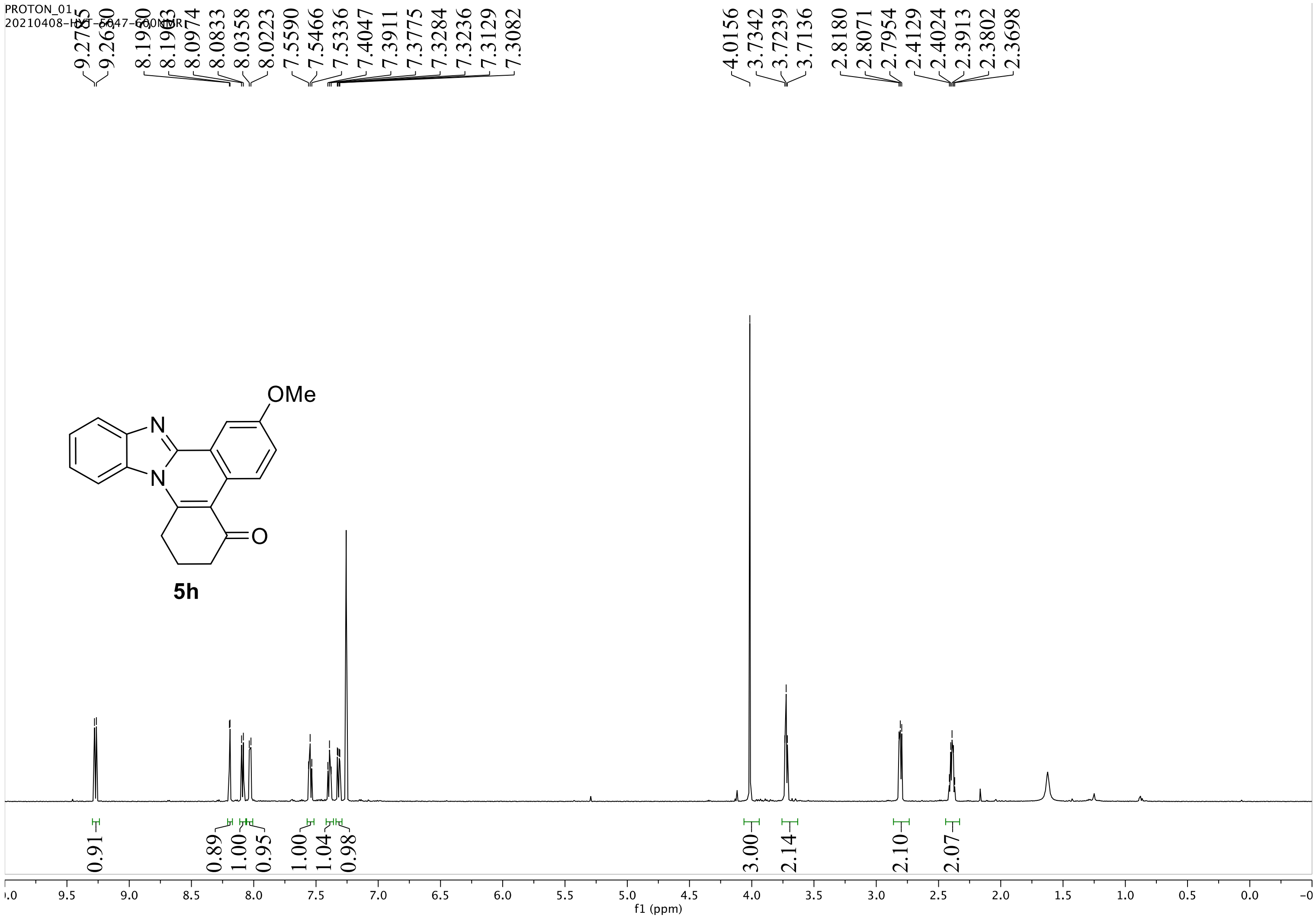

^{1}H NMR spectrum (600 MHz) of compound **5h** in $CDCl_3$

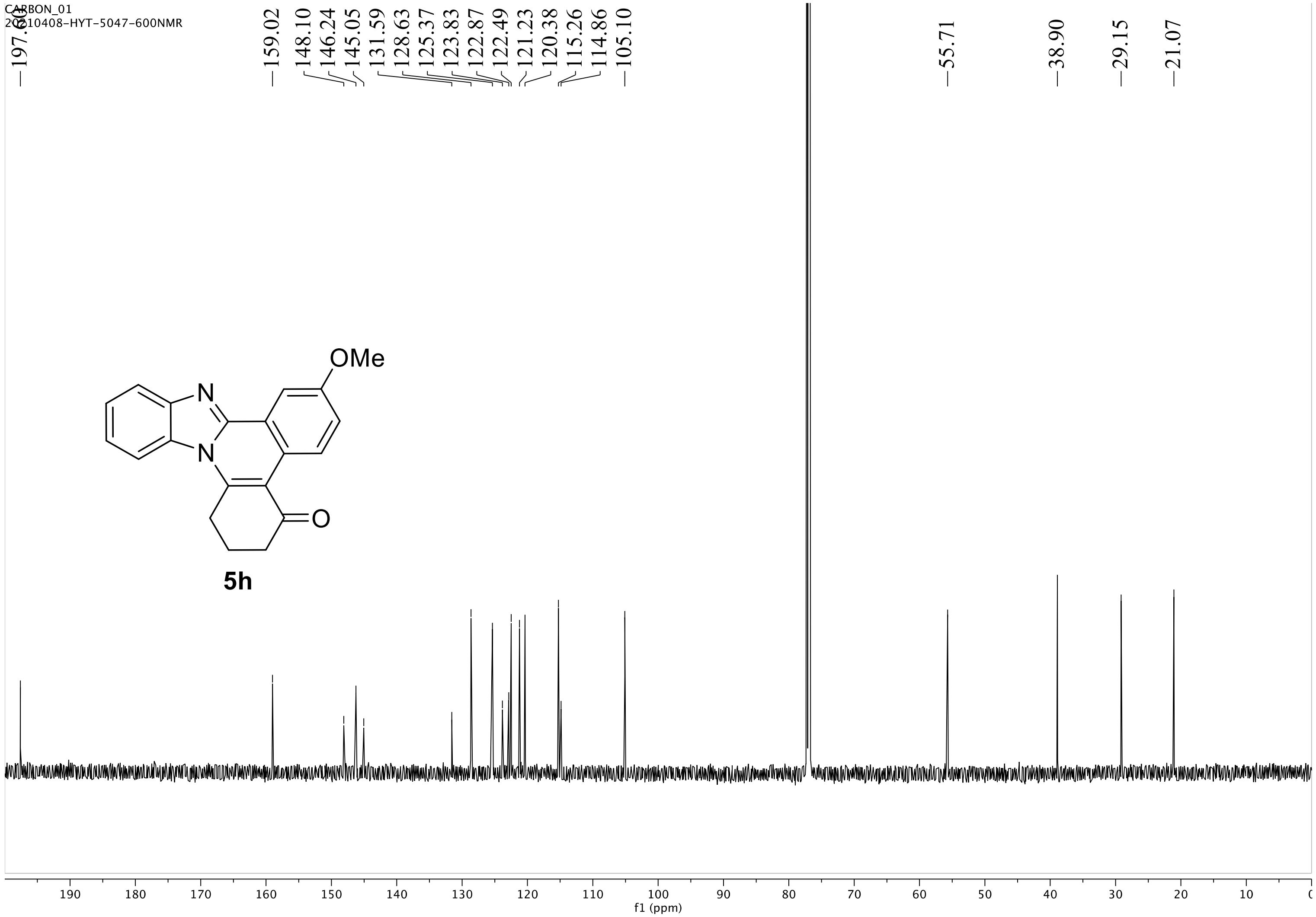

^{13}C{1H} NMR spectrum (150 MHz) of compound **5h** in $CDCl_3$

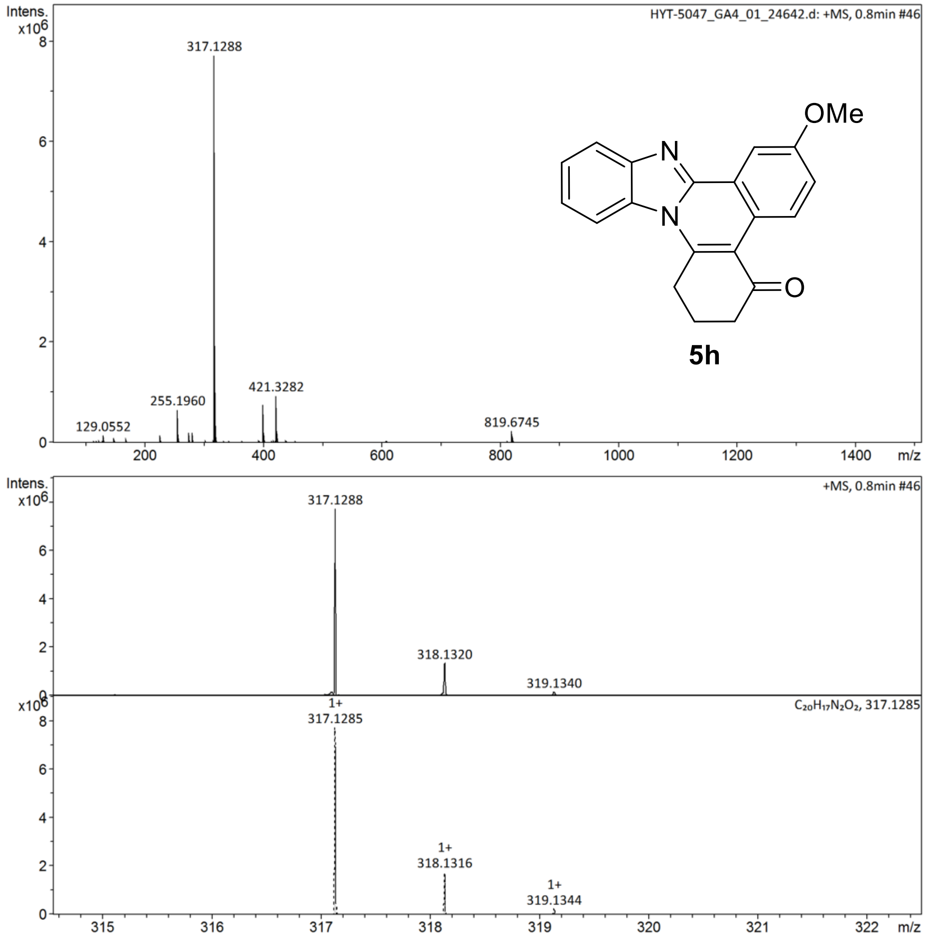

Display Report

Meas. m/z	#	Ion Formula	m/z	err [ppm]	mSigma	# Sigma	Score	rdb	e⁻ Conf	N-Rule	Adduct
317.1288	1	C20H17N2O2	317.1285	-1.0	31.6	1	100.00	13.5	even	ok	M+H

HRMS (ESI) of compound **5h**

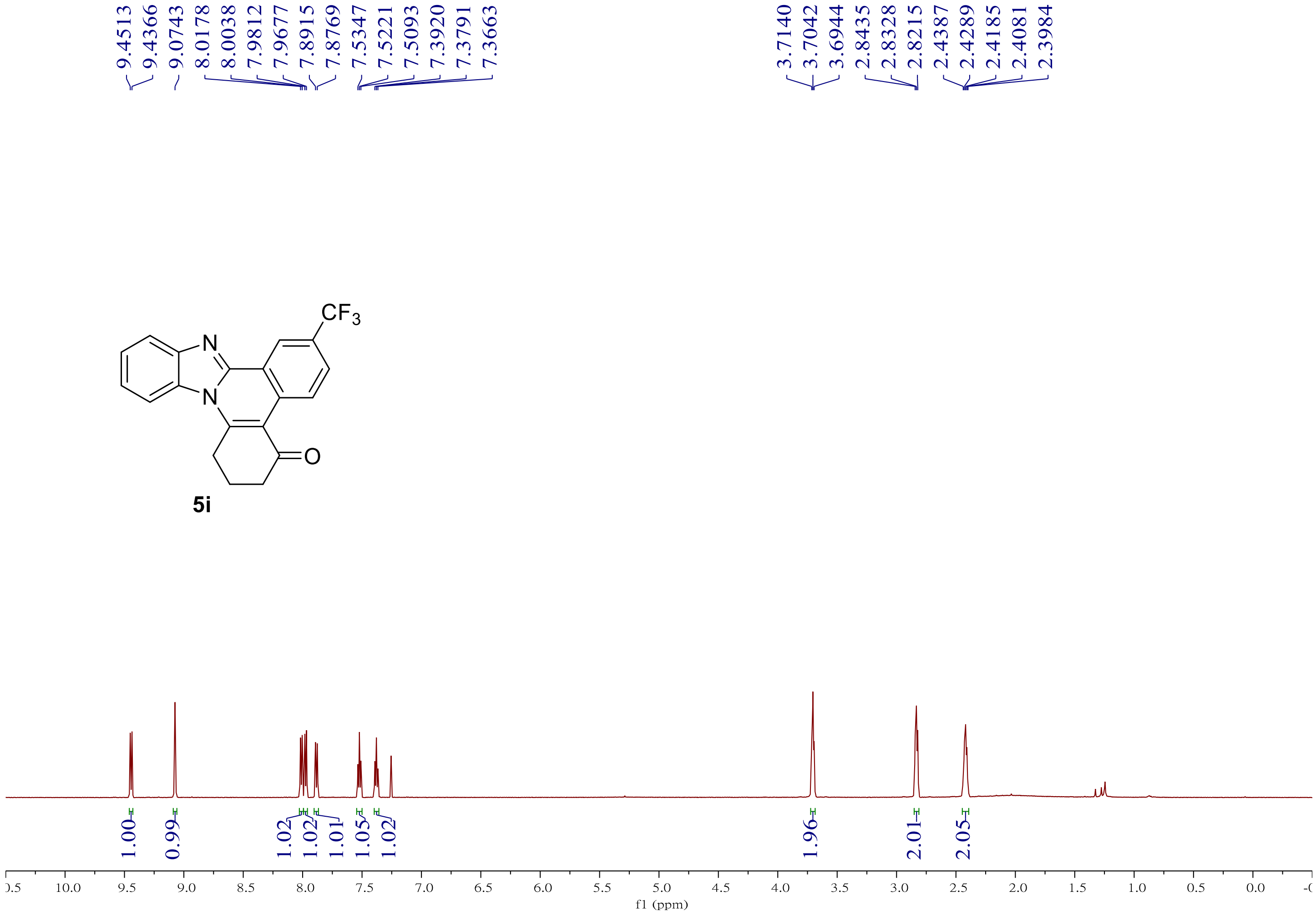

^{1}H NMR spectrum (600 MHz) of compound **5i** in $CDCl_3$

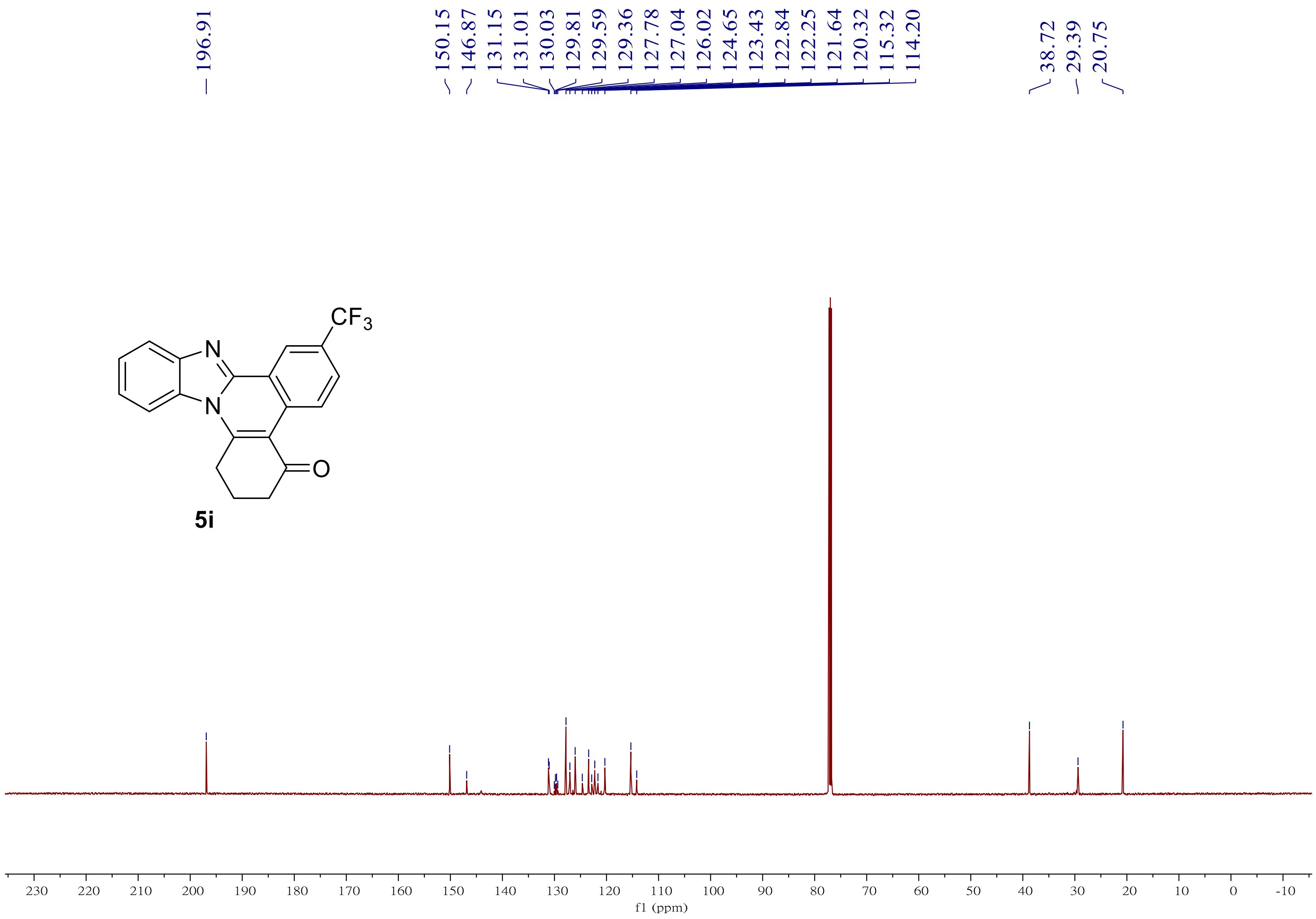

^{13}C{1H} NMR spectrum (150 MHz) of compound **5i** in $CDCl_3$

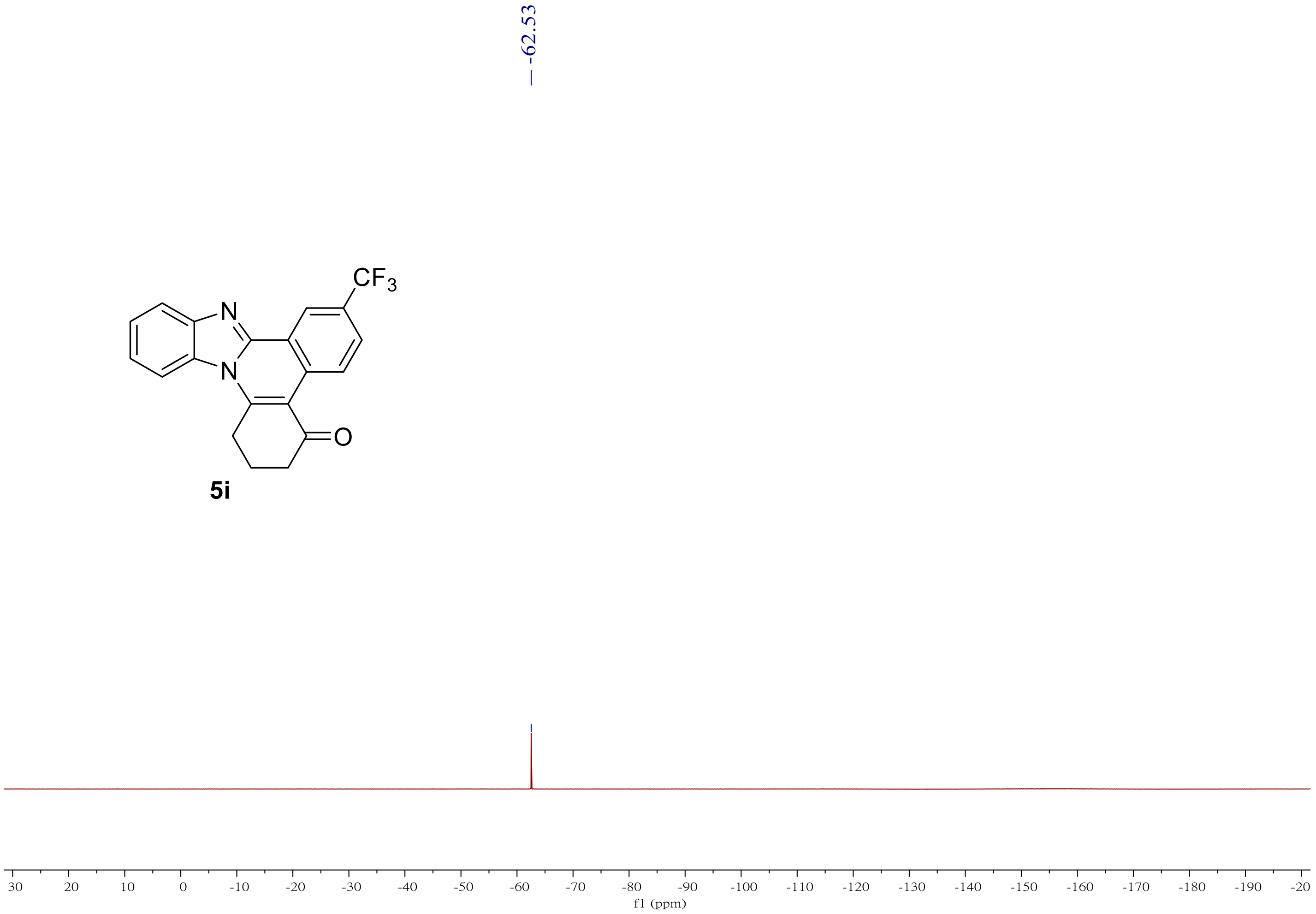

^{19}F NMR spectrum (376 MHz) of compound **5i** in $CDCl_3$

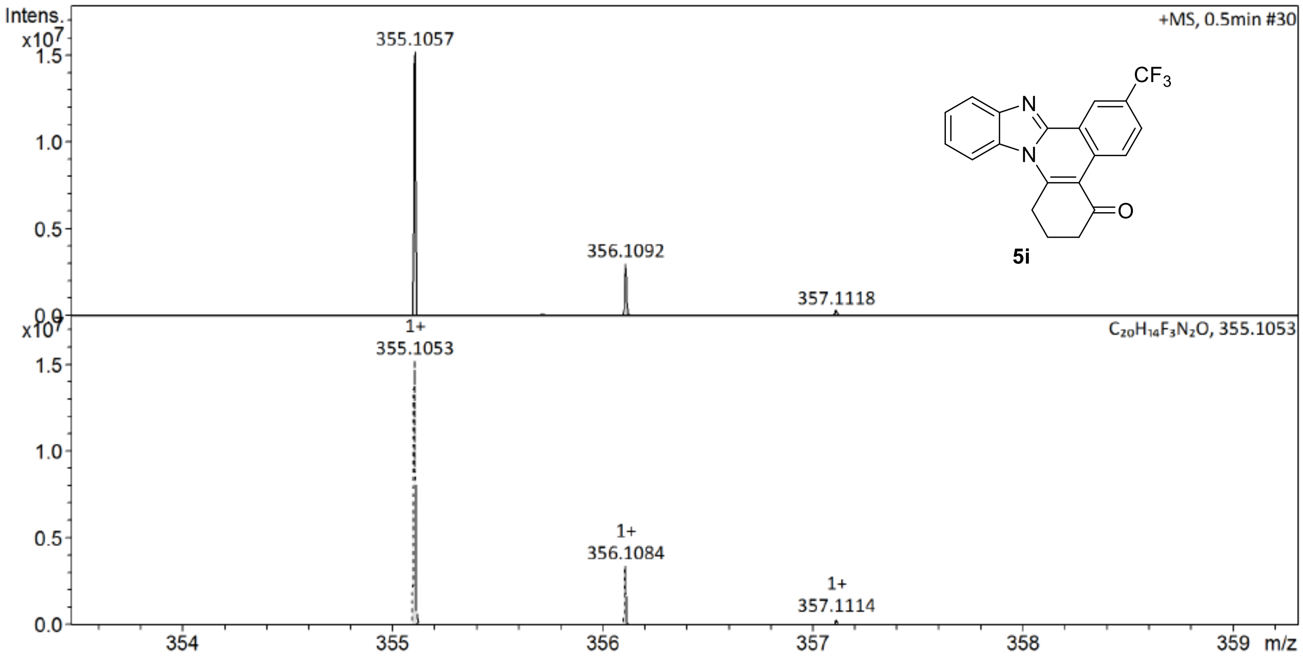

Display Report

Meas. m/z	#	Ion Formula	m/z	err [ppm]	mSigma	# Sigma	Score	rdb	e⁻ Conf	N-Rule	Adduct
355.1057	1	C20H14F3N2O	355.1053	-1.3	15.8	1	100.00	13.5	even	ok	M+H

HRMS (ESI) of compound **5i**

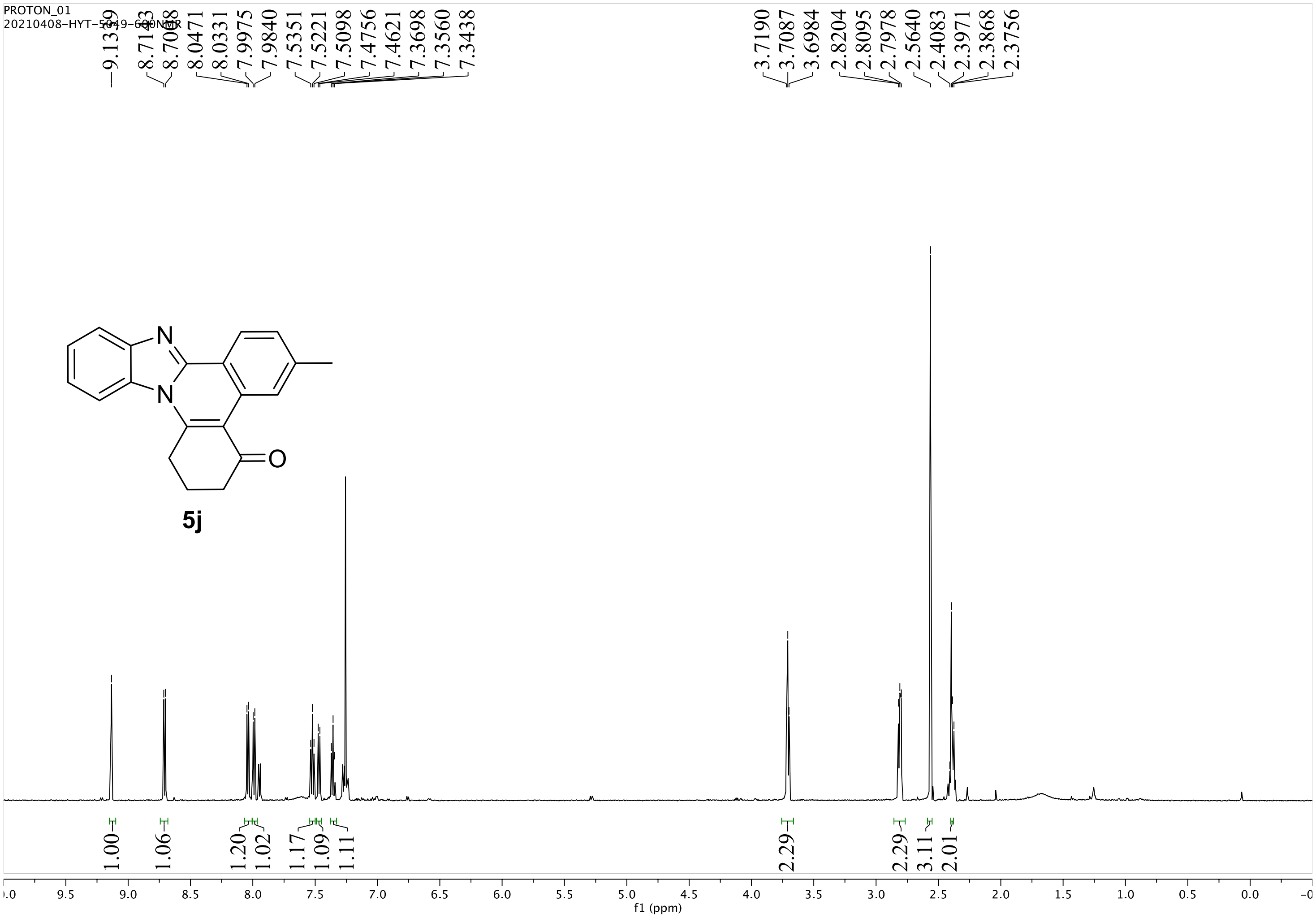

^{1}H NMR spectrum (600 MHz) of compound **5j** in $CDCl_3$

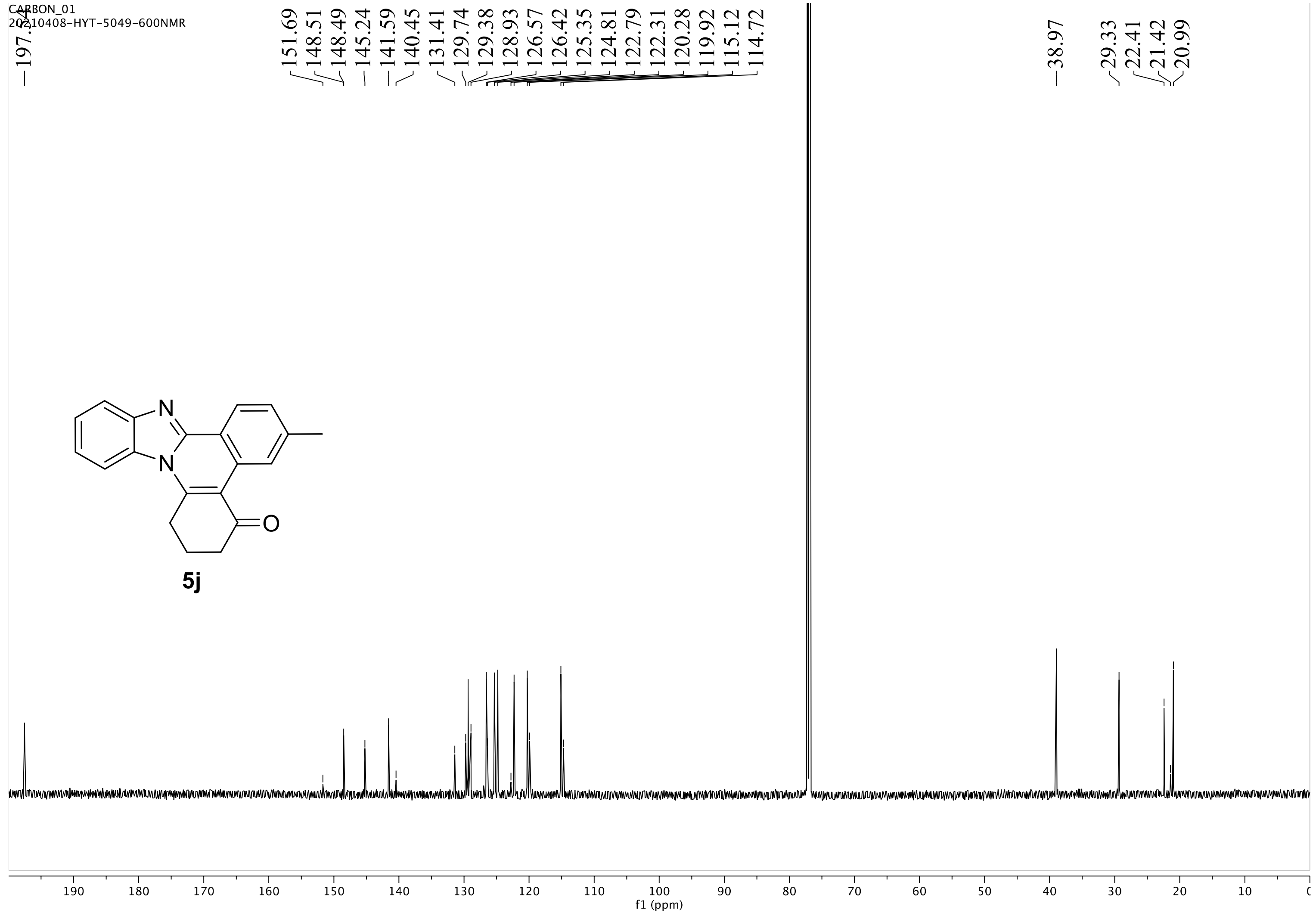

$^{13}C\{1H\}$ NMR spectrum (150 MHz) of compound **5j** in $CDCl_3$

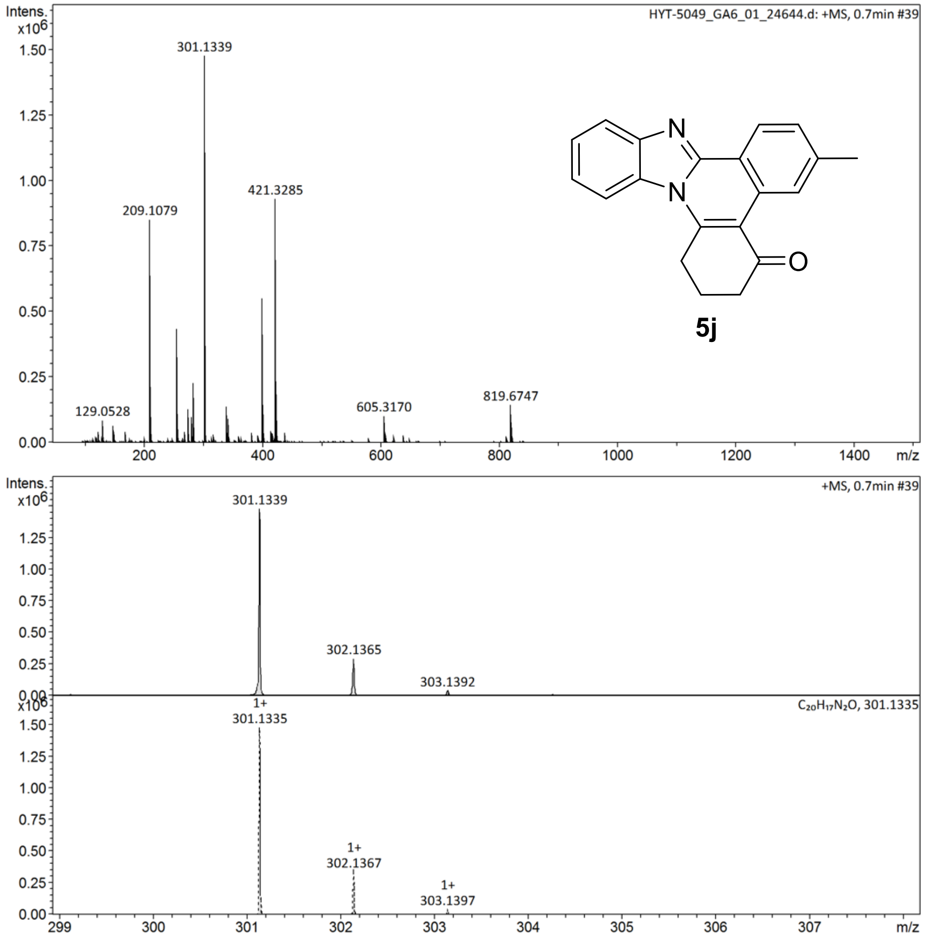

Display Report

Meas. m/z	#	Ion Formula	m/z	err [ppm]	mSigma	# Sigma	Score	rdb	e⁻ Conf	N-Rule	Adduct
301.1339	1	C20H17N2O	301.1335	-1.2	15.6	1	100.00	13.5	even	ok	M+H

HRMS (ESI) of compound **5j**

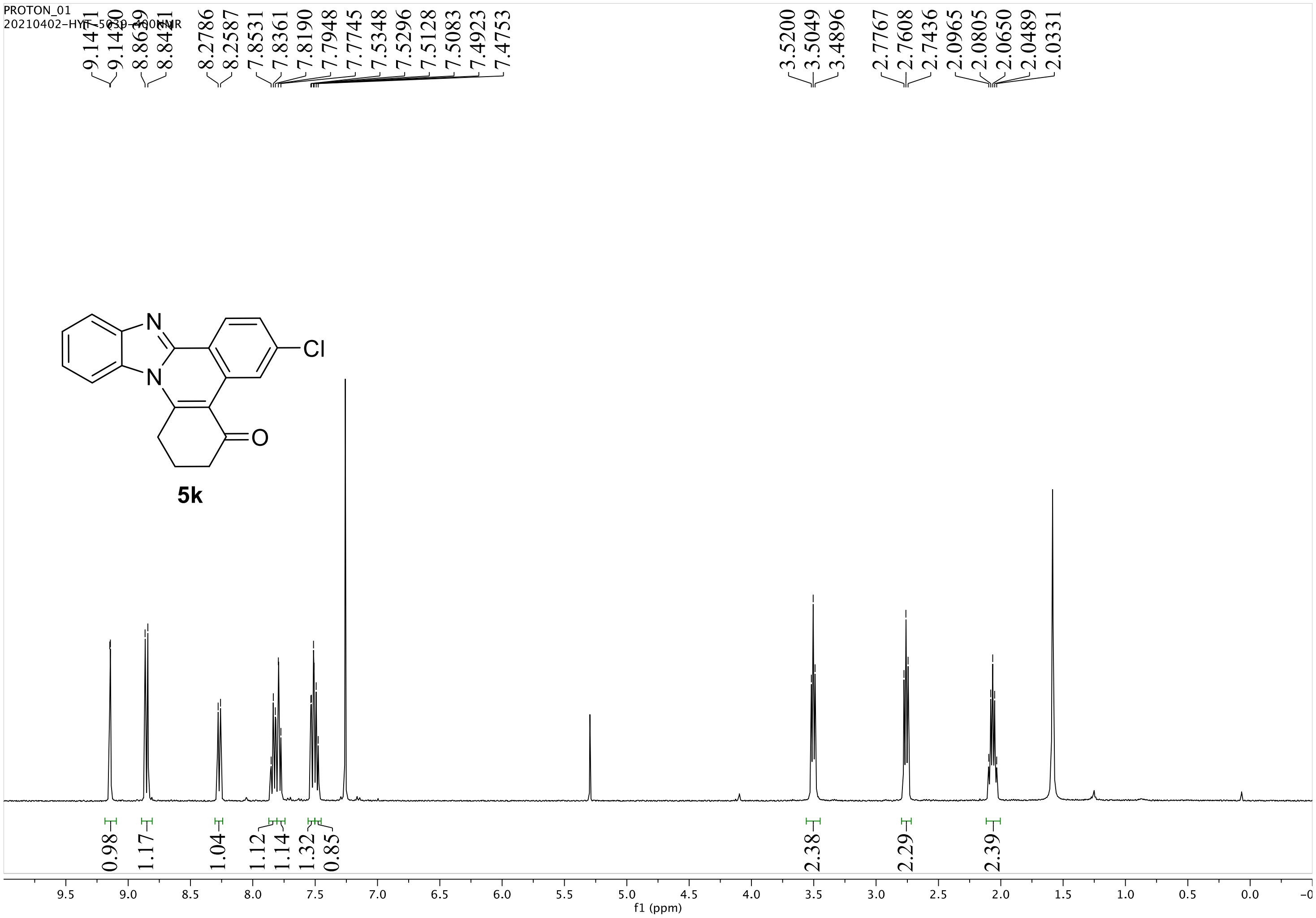

^{1}H NMR spectrum (400 MHz) of compound **5k** in $CDCl_3$

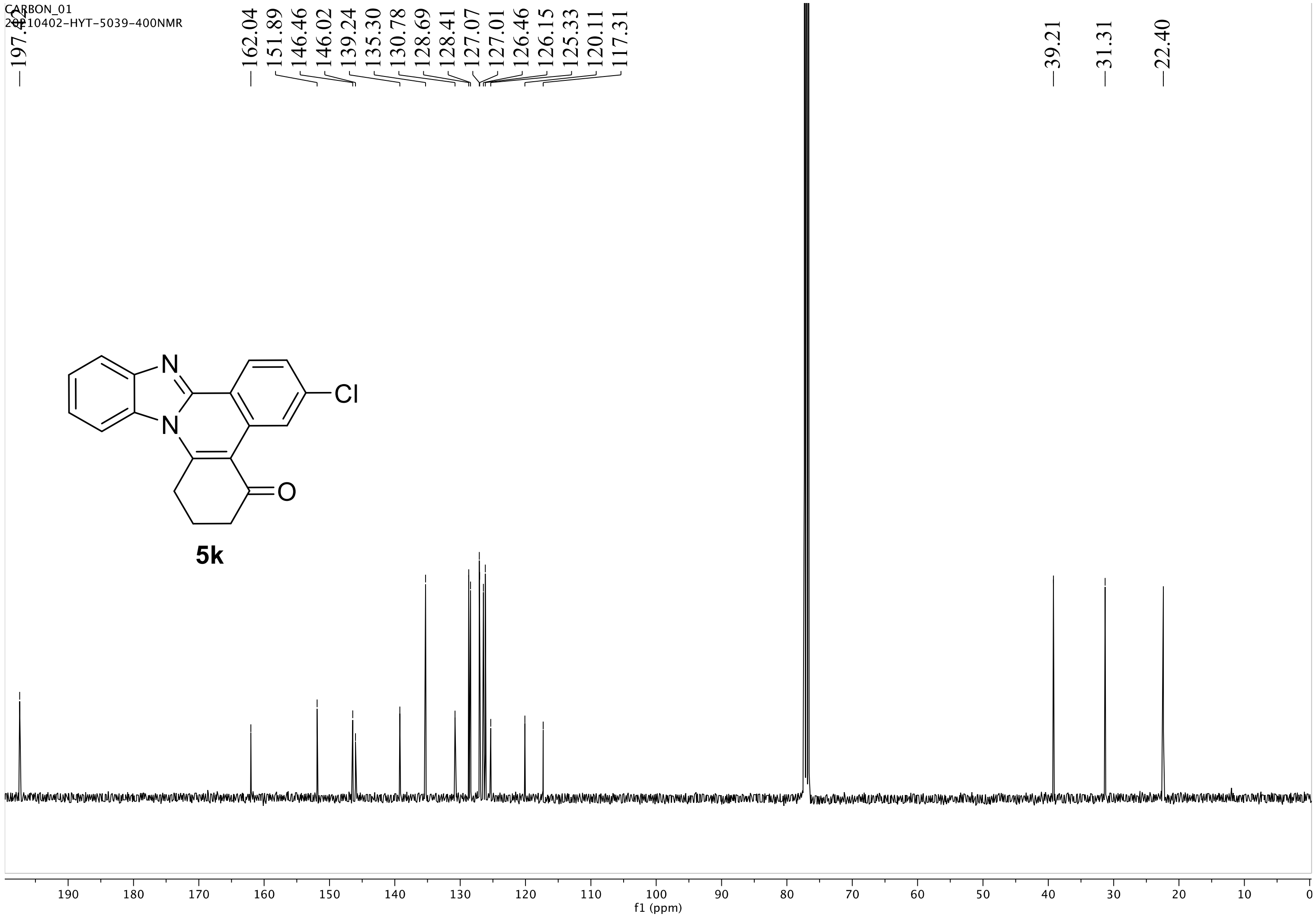

$^{13}C\{1H\}$ NMR spectrum (100 MHz) of compound **5k** in $CDCl_3$

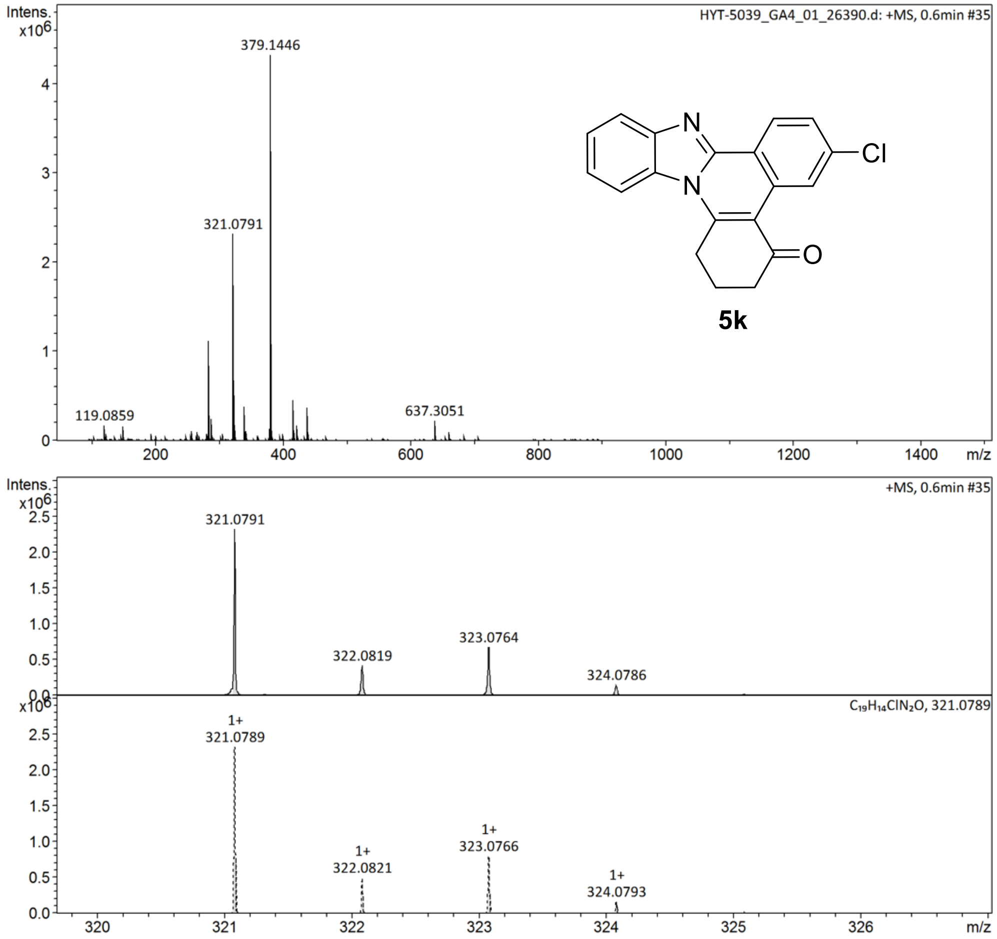

Display Report

Meas. m/z	#	Ion Formula	m/z	err [ppm]	mSigma	# Sigma	Score	rdb	e⁻ Conf	N-Rule	Adduct
321.0791	1	C19H14ClN2O	321.0789	-0.6	27.5	1	100.00	13.5	even	ok	M

HRMS (ESI) of compound **5k**

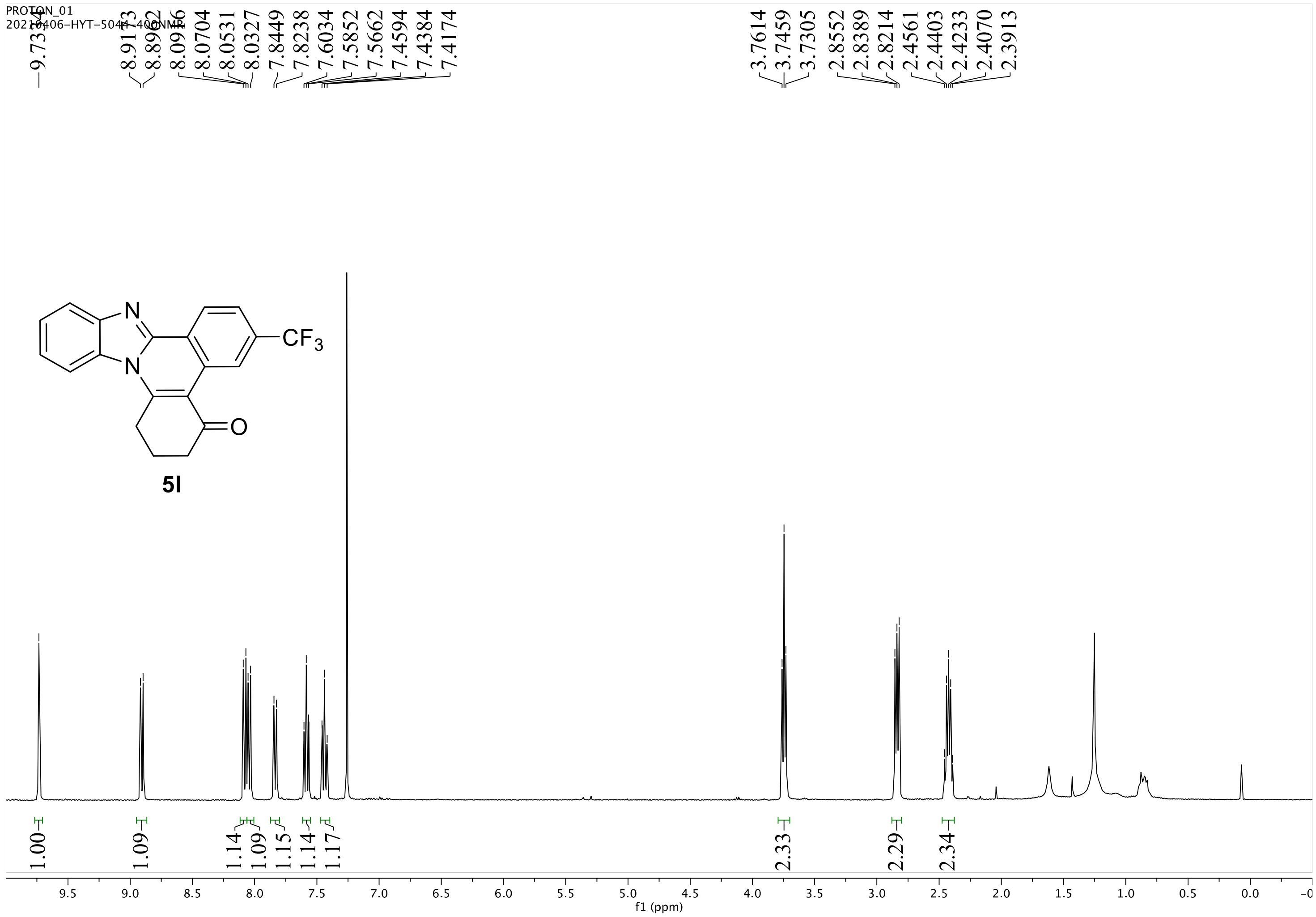

^1H NMR spectrum (400 MHz) of compound **5l** in $CDCl_3$

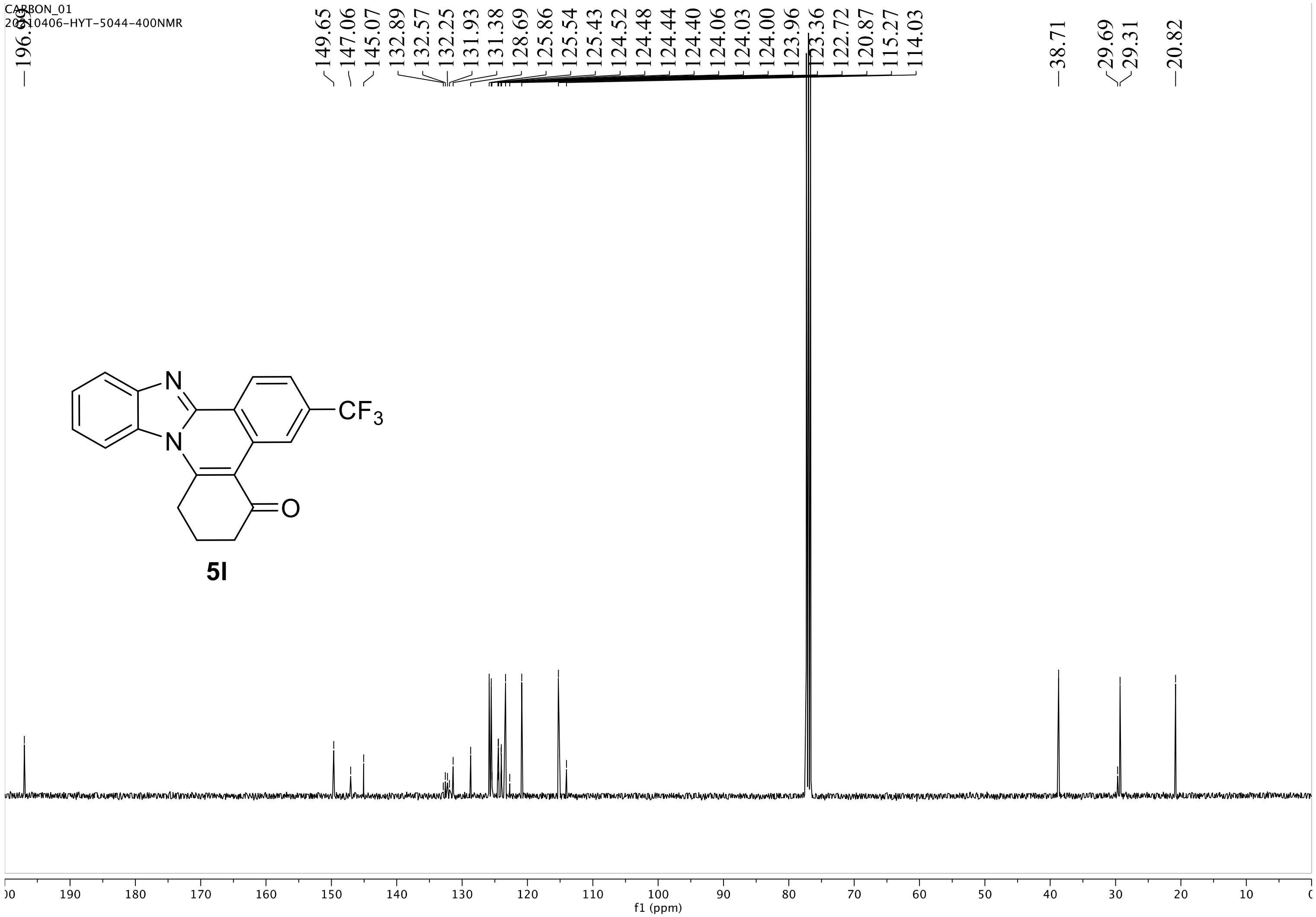

$^{13}C\{1H\}$ NMR spectrum (100 MHz) of compound **5l** in $CDCl_3$

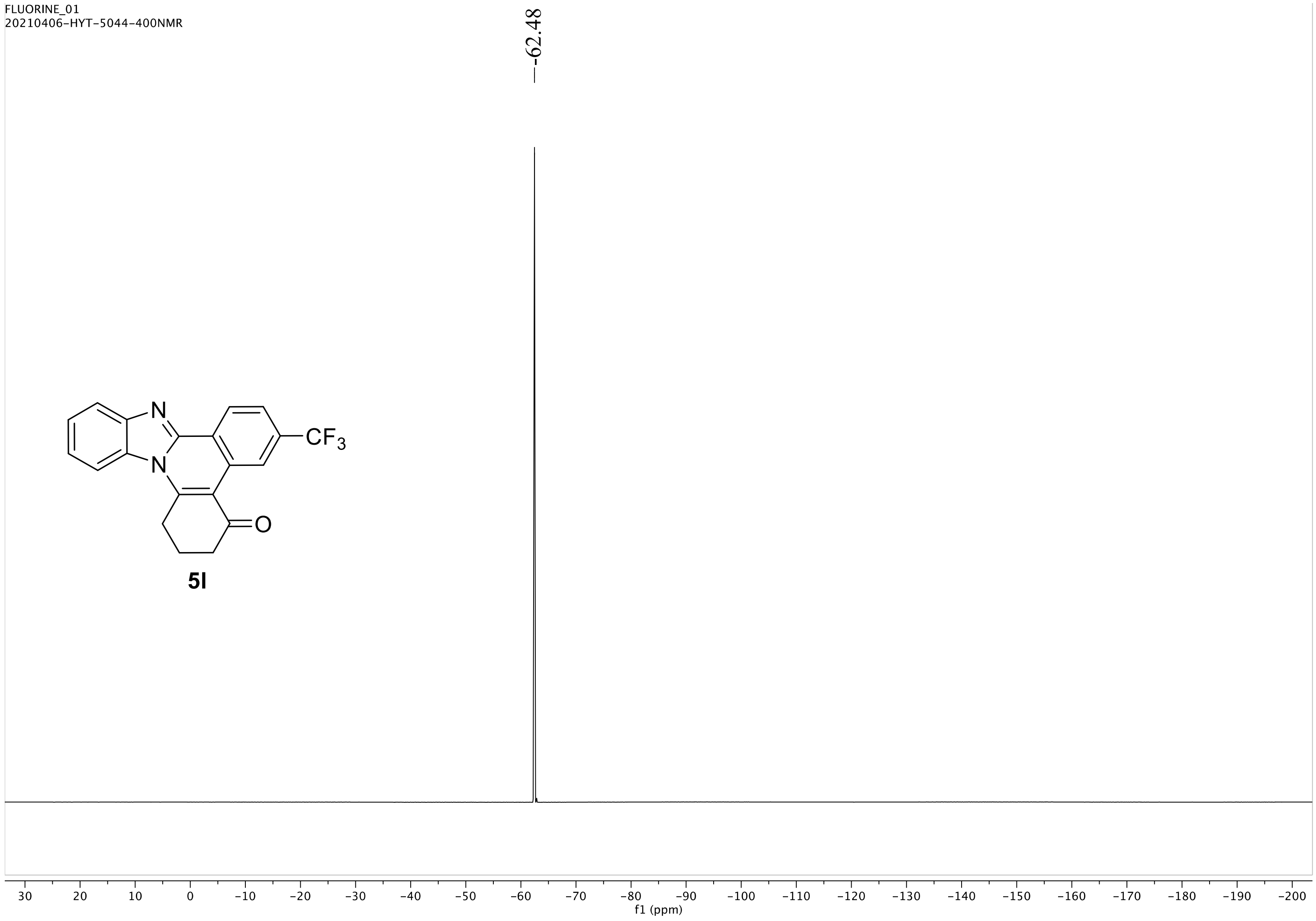

5l

^{19}F NMR spectrum (376 MHz) of compound **5l** in $CDCl_3$.

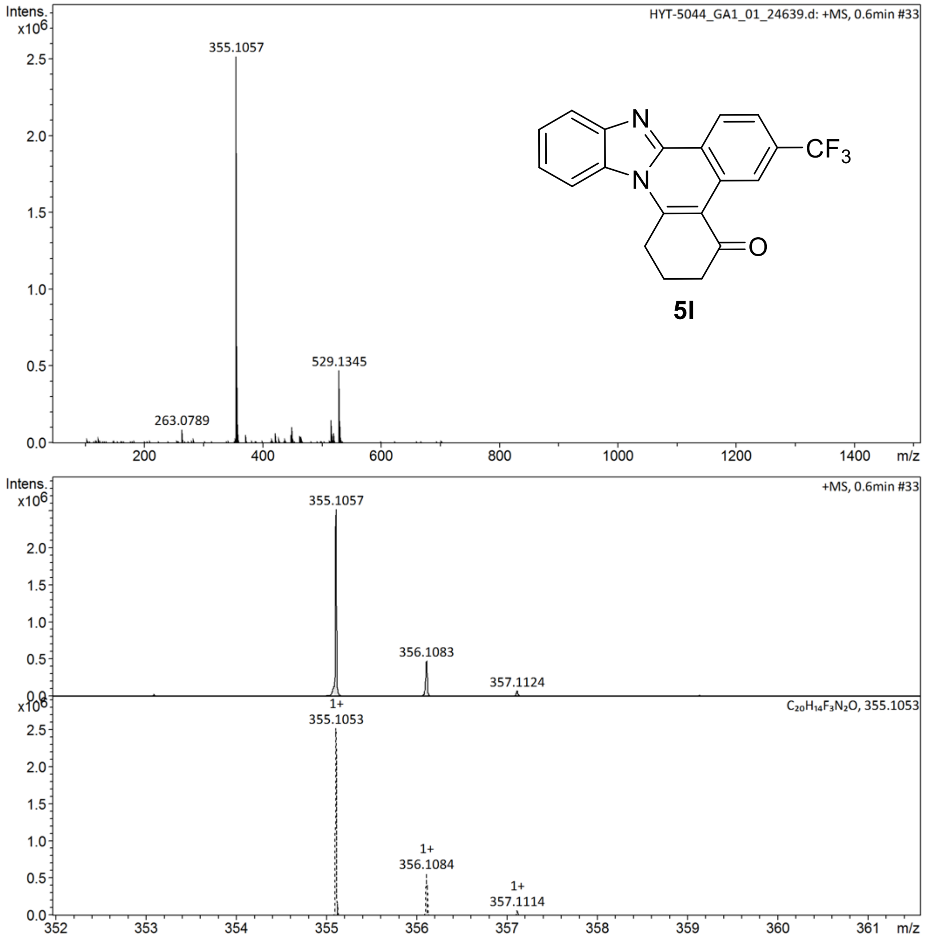

Display Report

Meas. m/z	#	Ion Formula	m/z	err [ppm]	mSigma	# Sigma	Score	rdb	e⁻ Conf	N-Rule	Adduct
355.1057	1	C20H14F3N2O	355.1053	-1.1	20.6	1	100.00	13.5	even	ok	M+H

HRMS (ESI) of compound **5l**

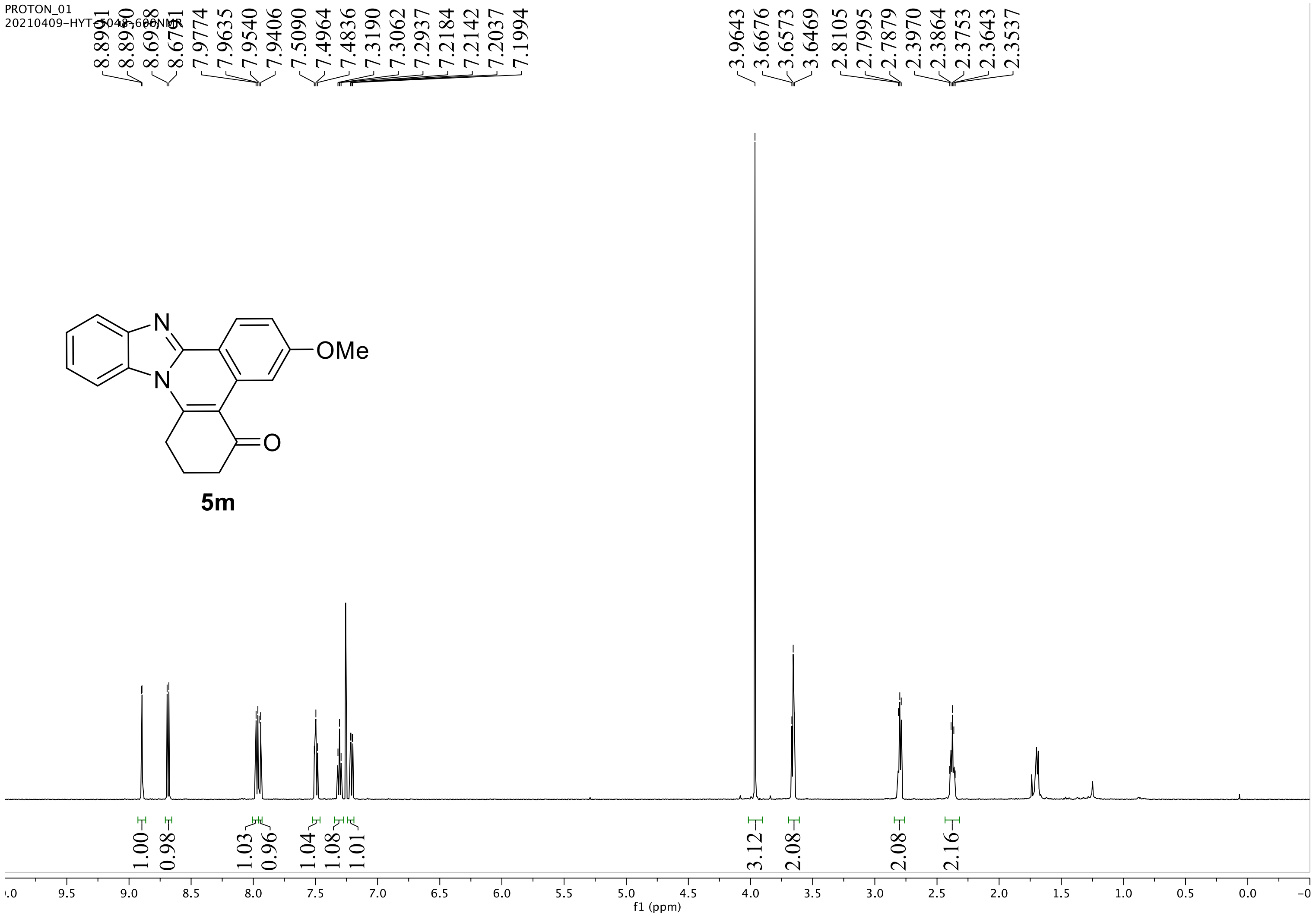

^{1}H NMR spectrum (600 MHz) of compound **5m** in $CDCl_3$

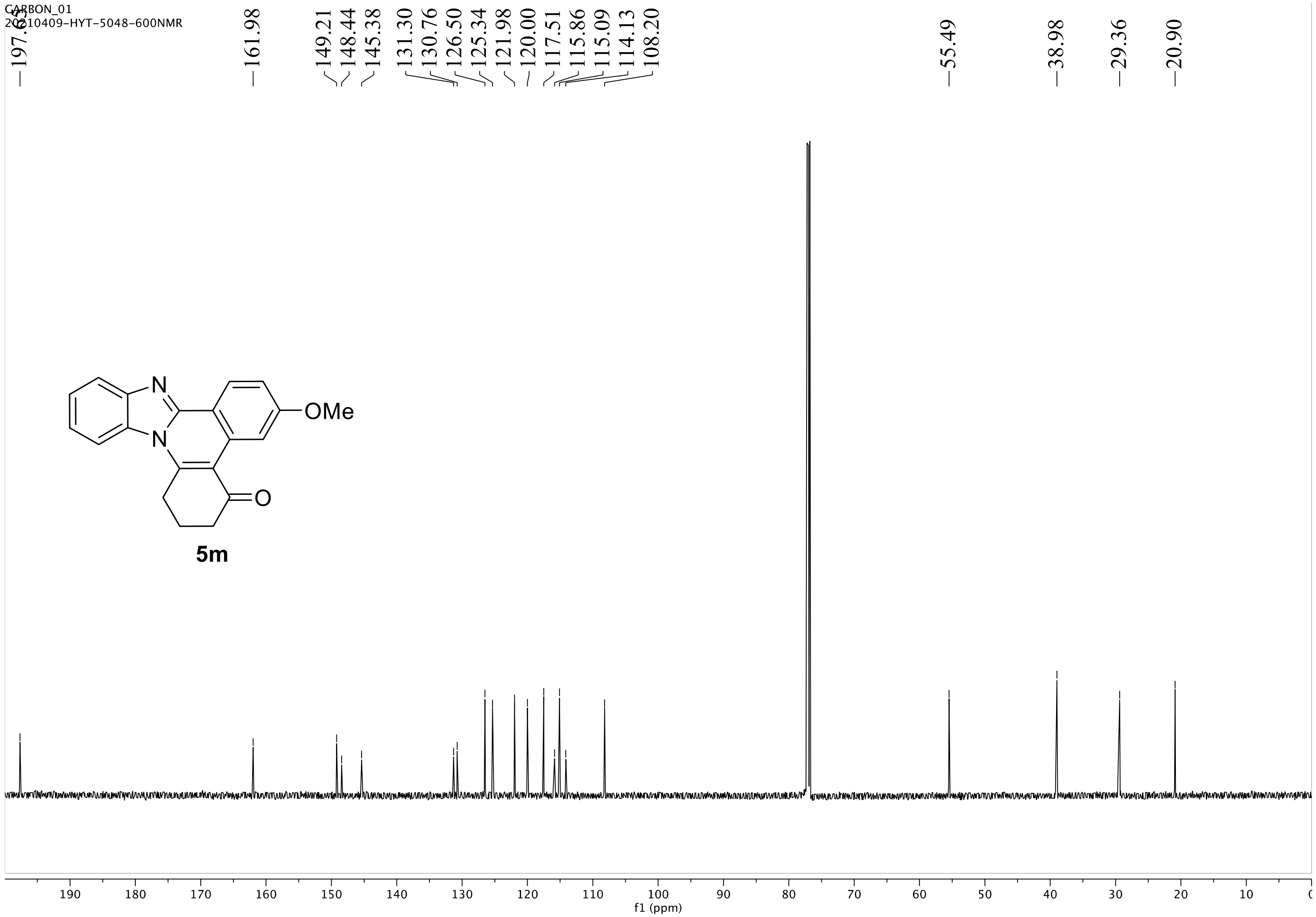

$^{13}C\{1H\}$ NMR spectrum (150 MHz) of compound **5m** in $CDCl_3$

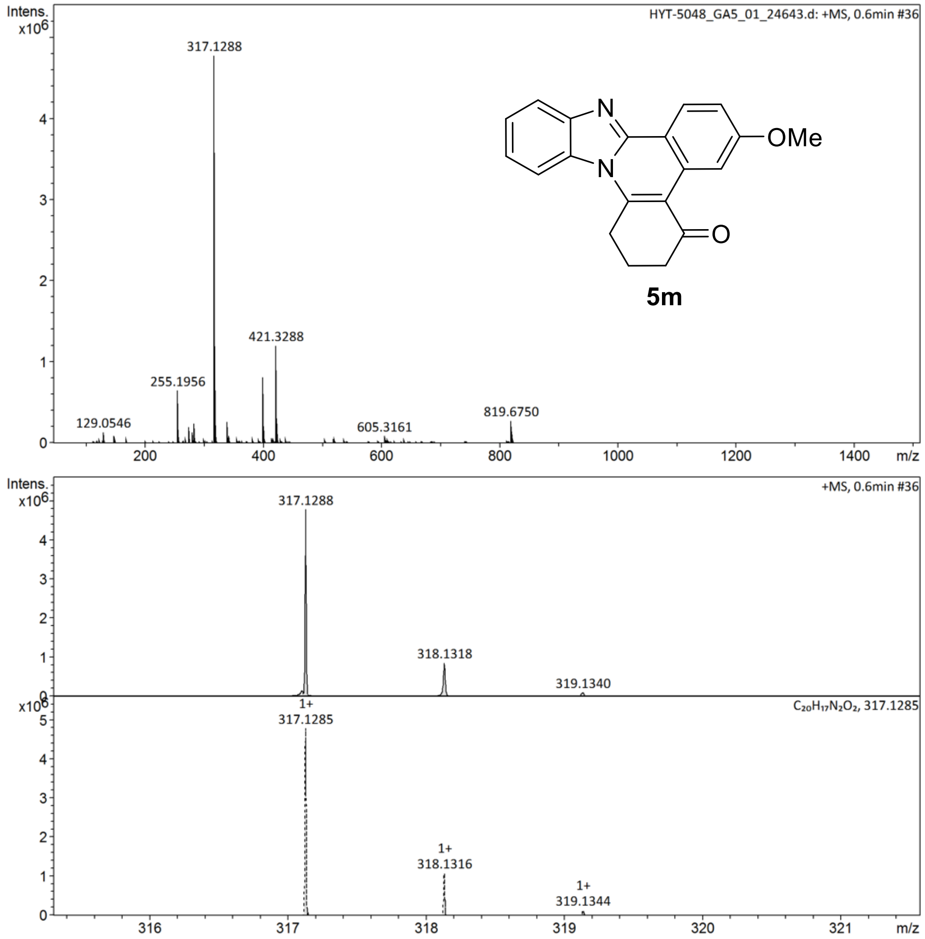

Display Report

Meas. m/z	#	Ion Formula	m/z	err [ppm]	mSigma	# Sigma	Score	rdb	e⁻ Conf	N-Rule	Adduct
317.1288	1	C20H17N2O2	317.1285	-1.0	26.6	1	100.00	13.5	even	ok	M+H

HRMS (ESI) of compound **5m**

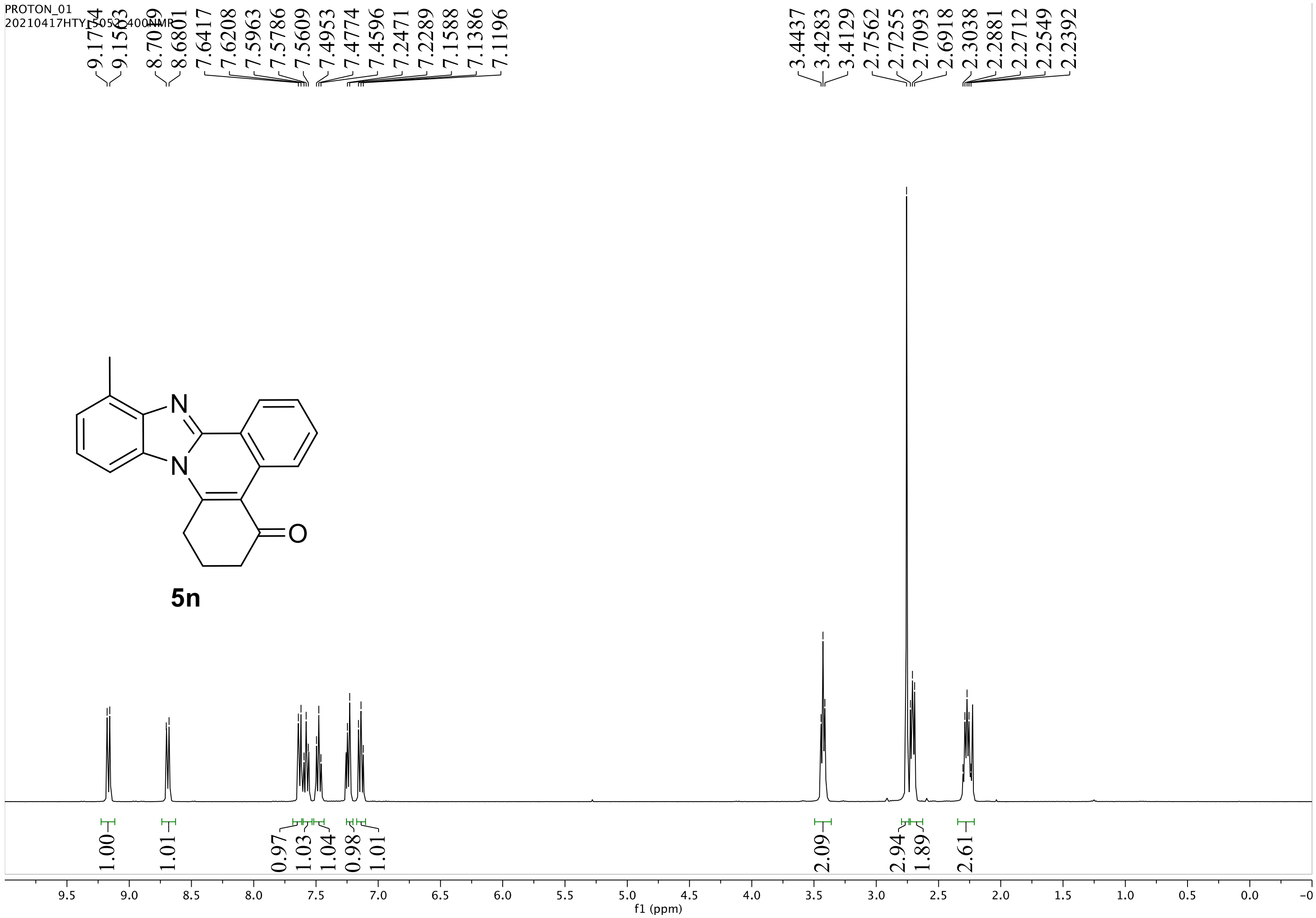

^{1}H NMR spectrum (400 MHz) of compound **5n** in $CDCl_3$.

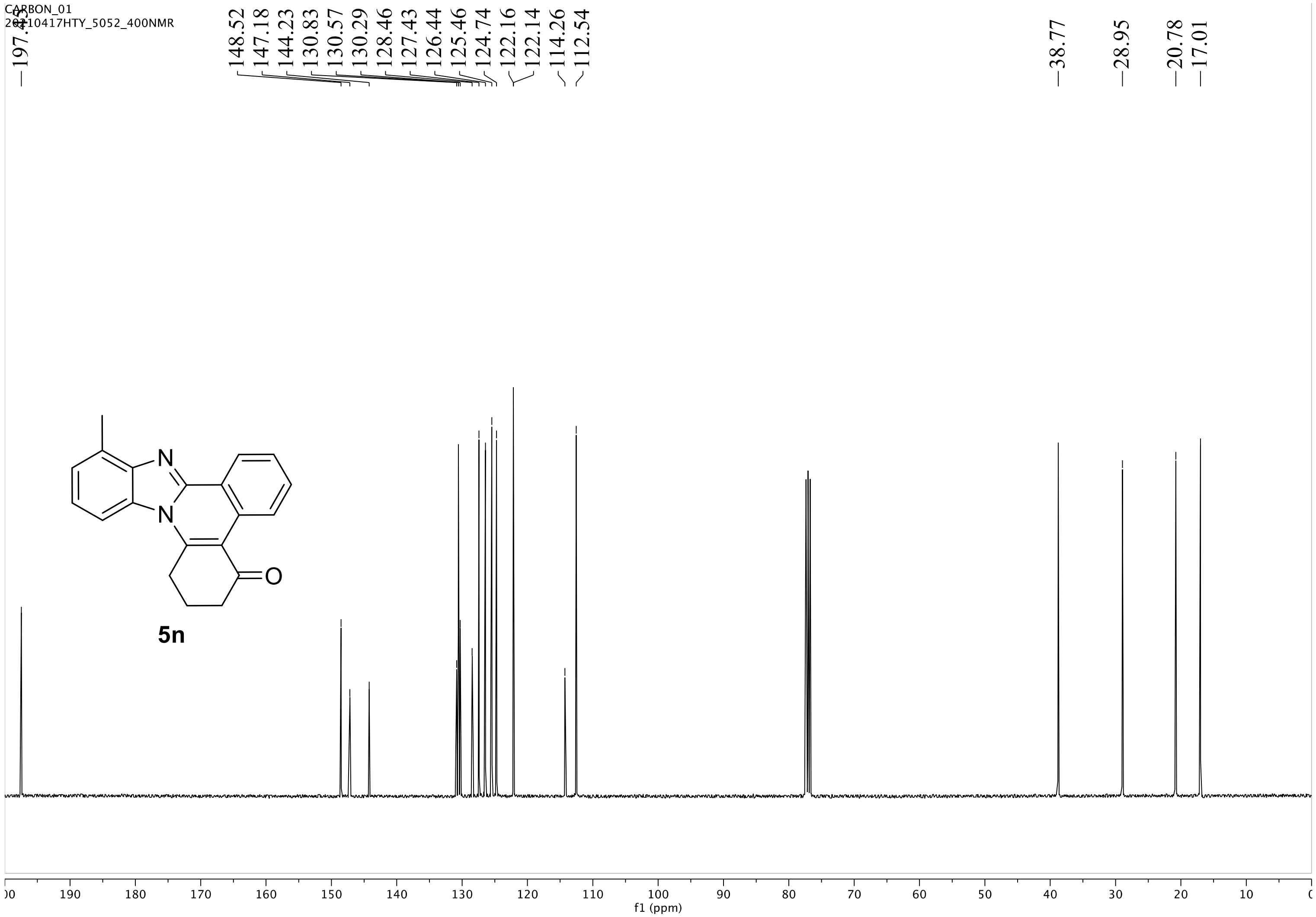

$^{13}C\{1H\}$ NMR spectrum (100 MHz) of compound **5n** in $CDCl_3$.

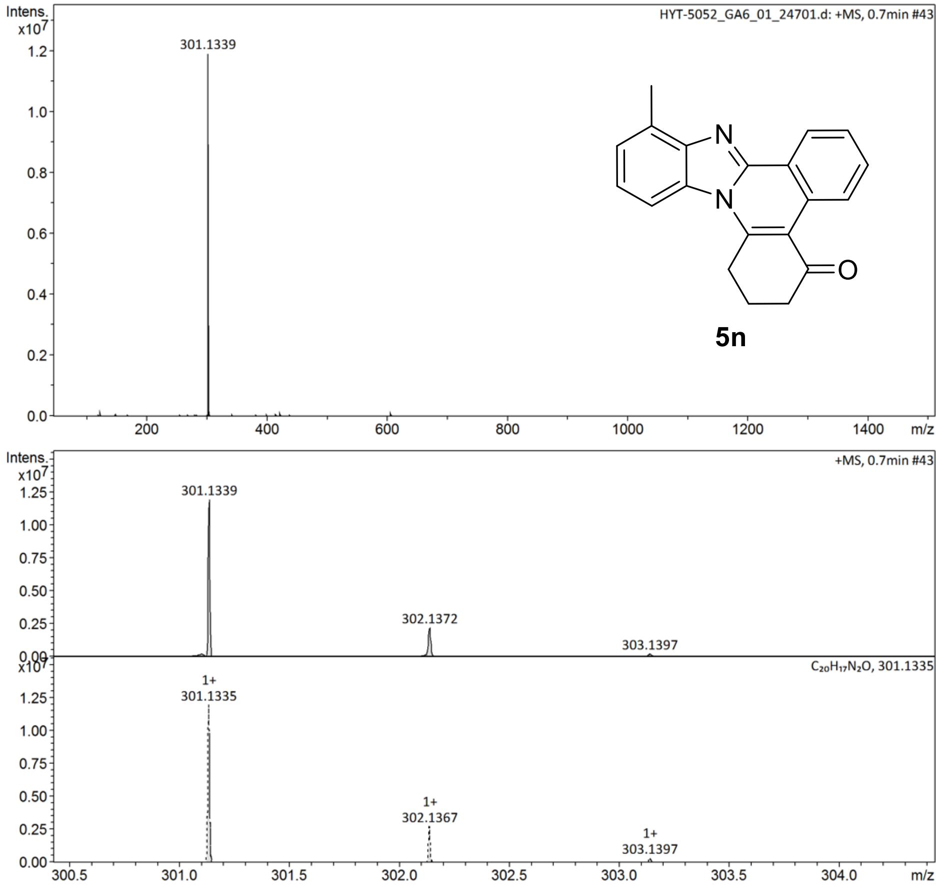

Display Report

Meas. m/z	#	Ion Formula	m/z	err [ppm]	mSigma	# Sigma	Score	rdb	e⁻ Conf	N-Rule	Adduct
301.1339	1	C20H17N2O	301.1335	-1.3	25.0	1	100.00	13.5	even	ok	M+H

HRMS (ESI) of compound **5n**

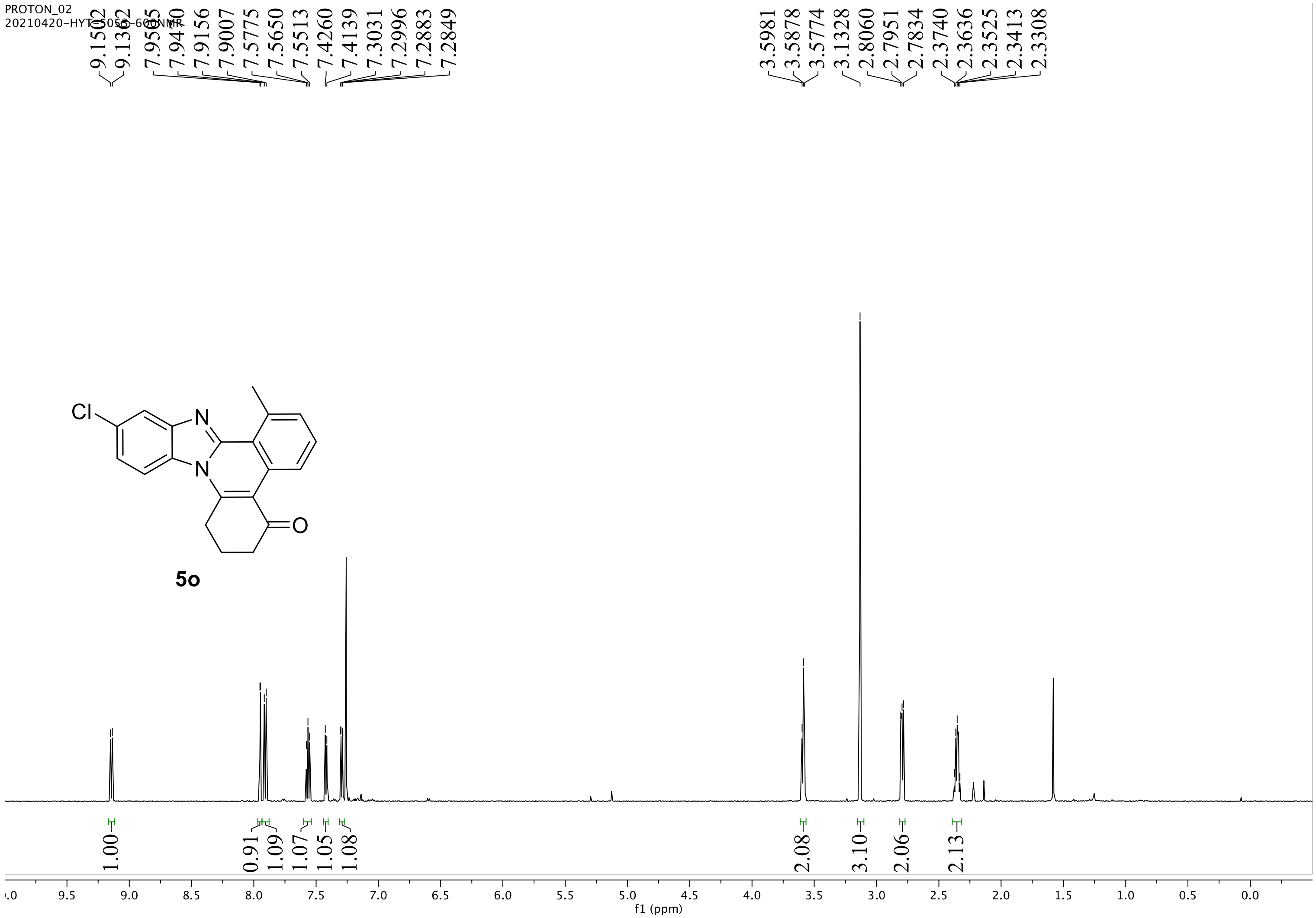

^{1}H NMR spectrum (600 MHz) of compound **5o** in $CDCl_3$.

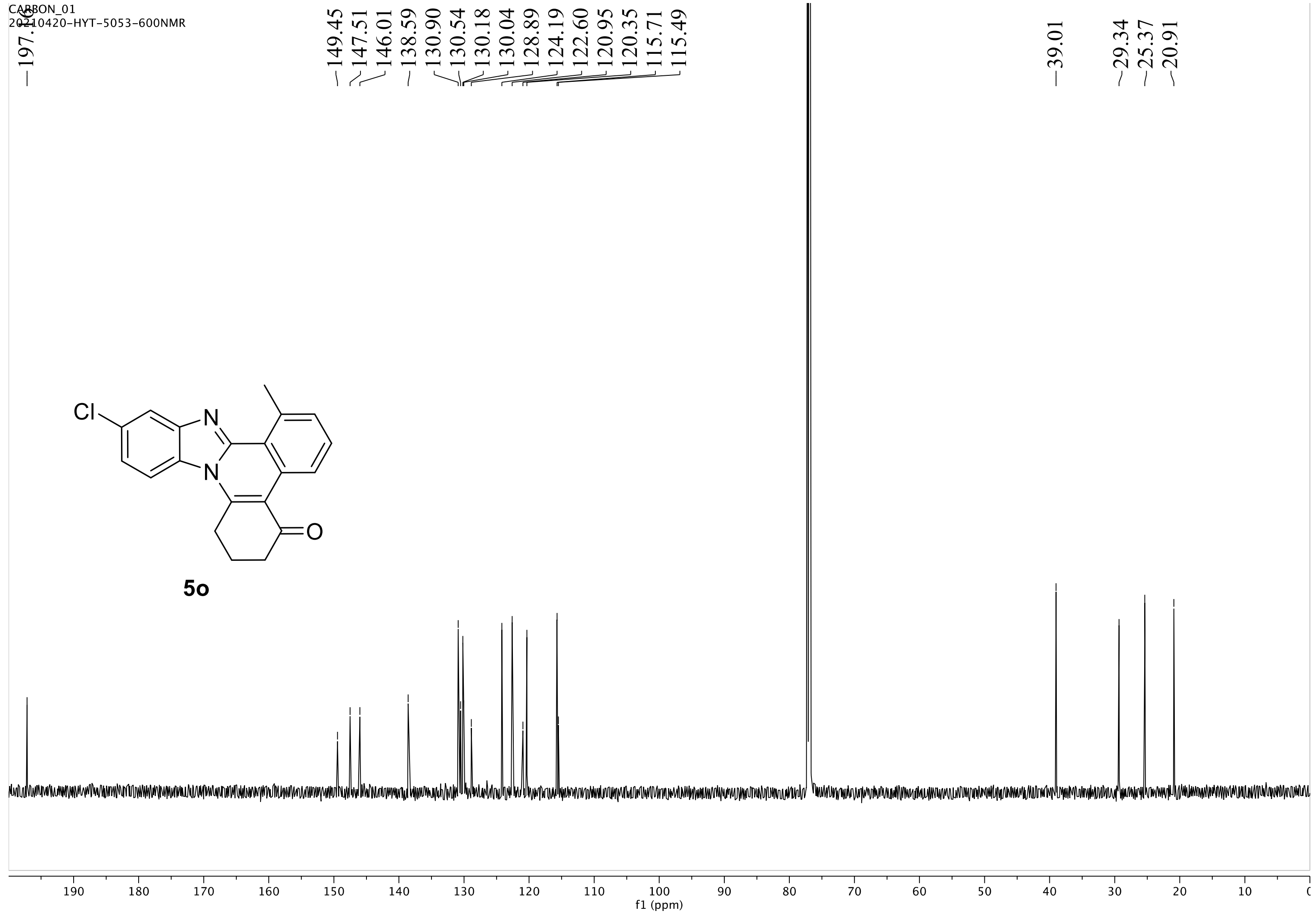

$^{13}C\{^1H\}$ NMR spectrum (150 MHz) of compound **5o** in $CDCl_3$.

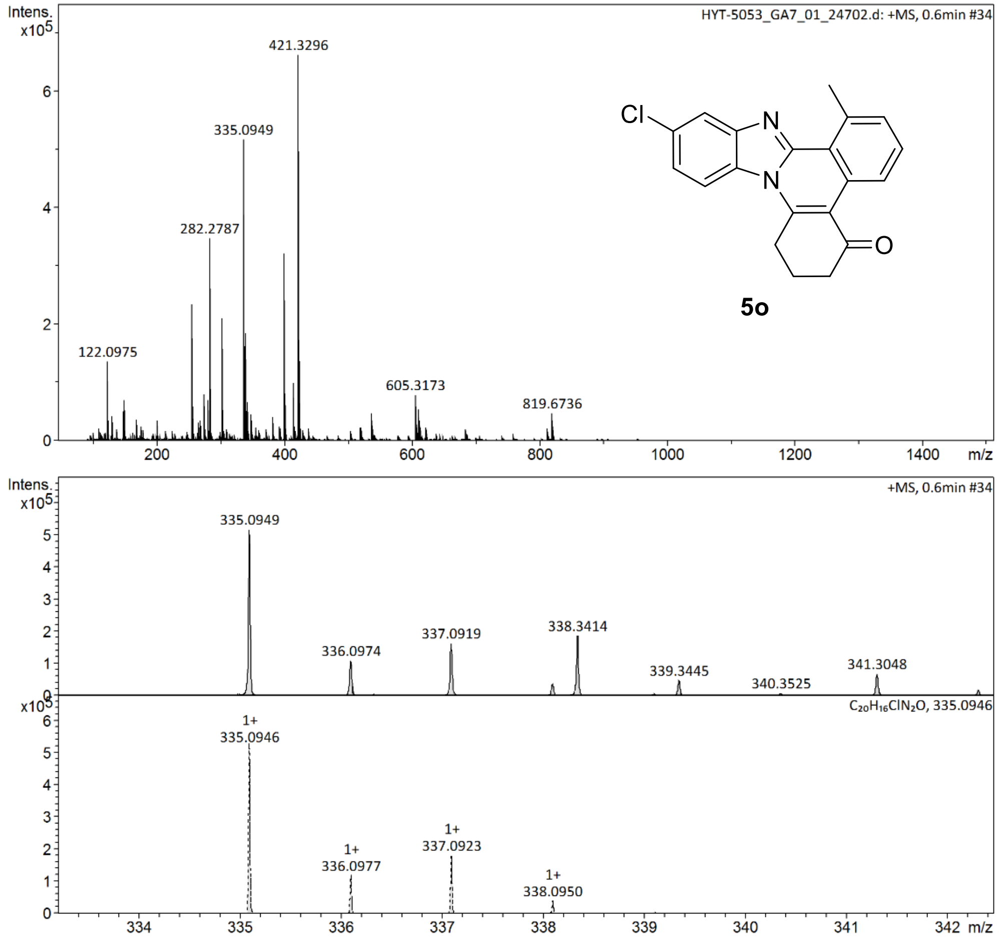

Display Report

Meas. m/z	#	Ion Formula	m/z	err [ppm]	mSigma	# Sigma	Score	rdb	e⁻ Conf	N-Rule	Adduct
335.0949	1	C20H16ClN2O	335.0946	1.0	16.2	1	100.00	13.5	even	ok	M+H

HRMS (ESI) of compound **5o**

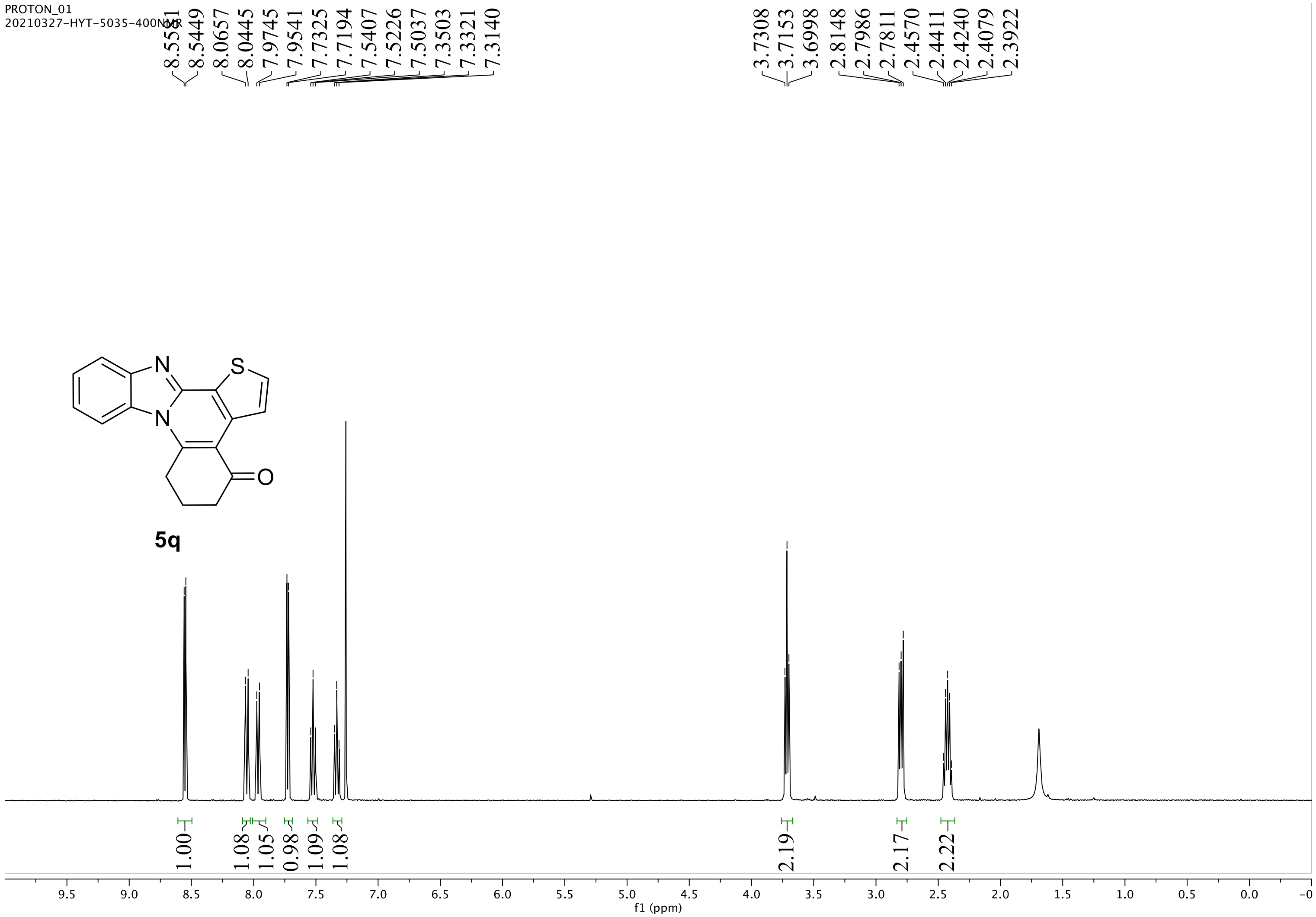

^{1}H NMR spectrum (400 MHz) of compound **5q** in $CDCl_3$.

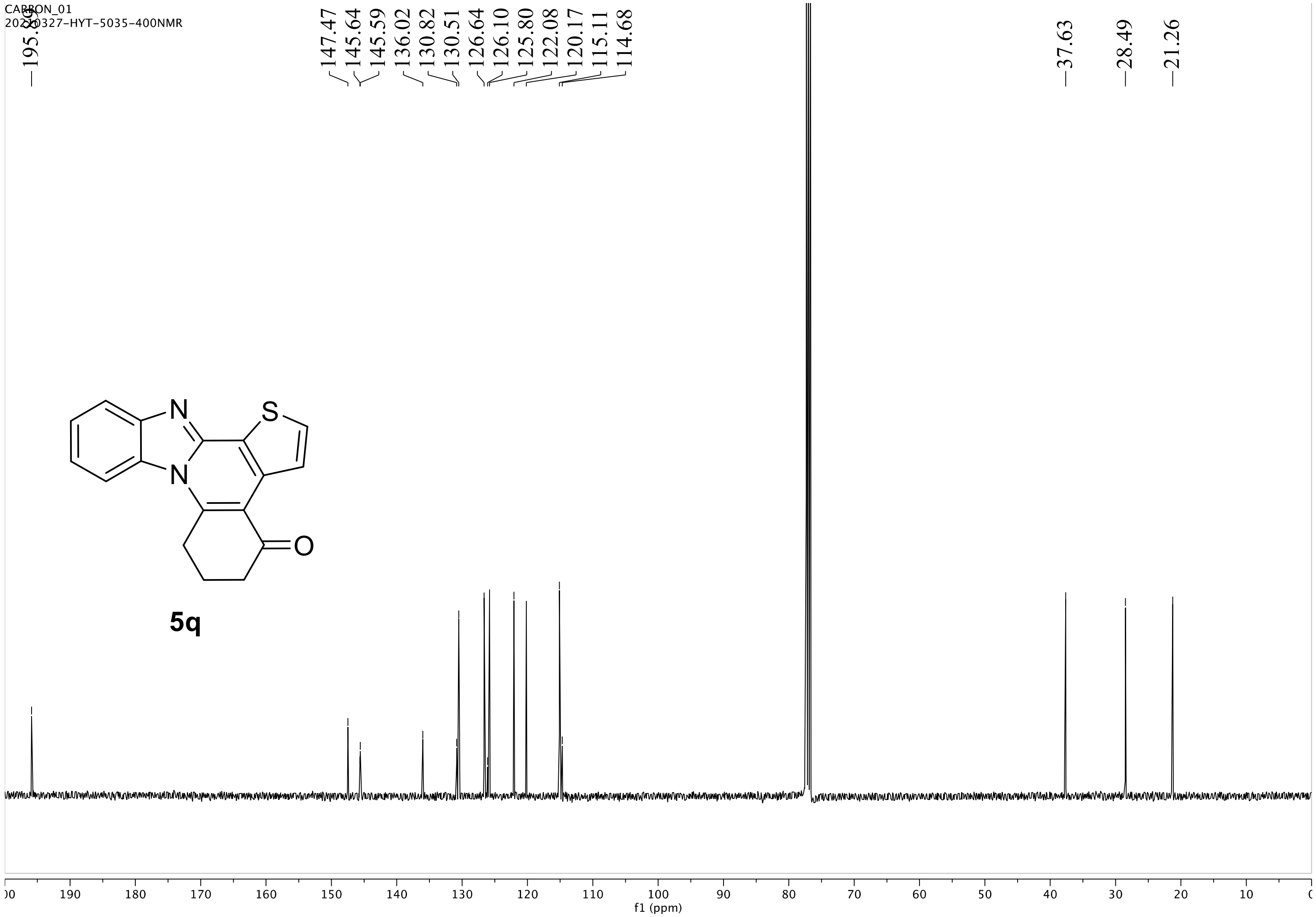

$^{13}C\{1H\}$ NMR spectrum (100 MHz) of compound **5q** in $CDCl_3$.

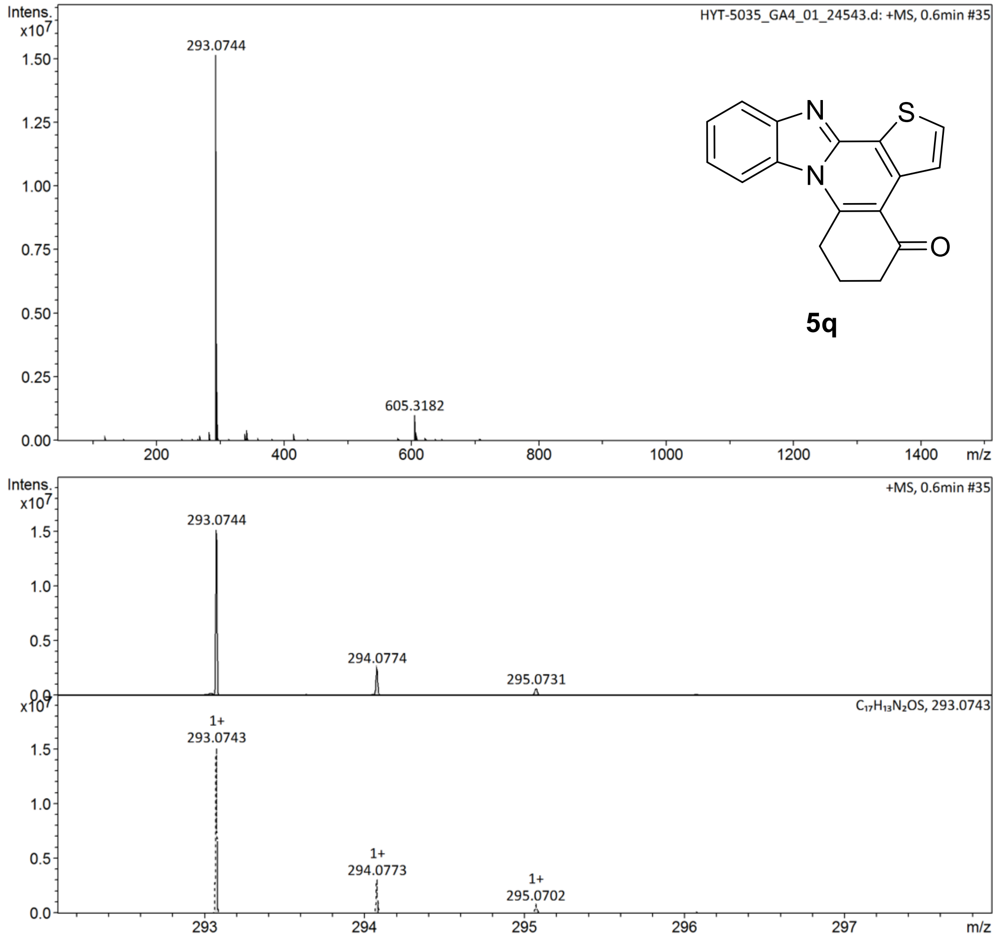

Display Report

Meas. m/z	#	Ion Formula	m/z	err [ppm]	mSigma	# Sigma	Score	rdb	e⁻ Conf	N-Rule	Adduct
293.0744	1	C17H13N2OS	293.0743	-0.2	21.3	1	100.00	12.5	even	ok	M+H

HRMS (ESI) of compound **5q**

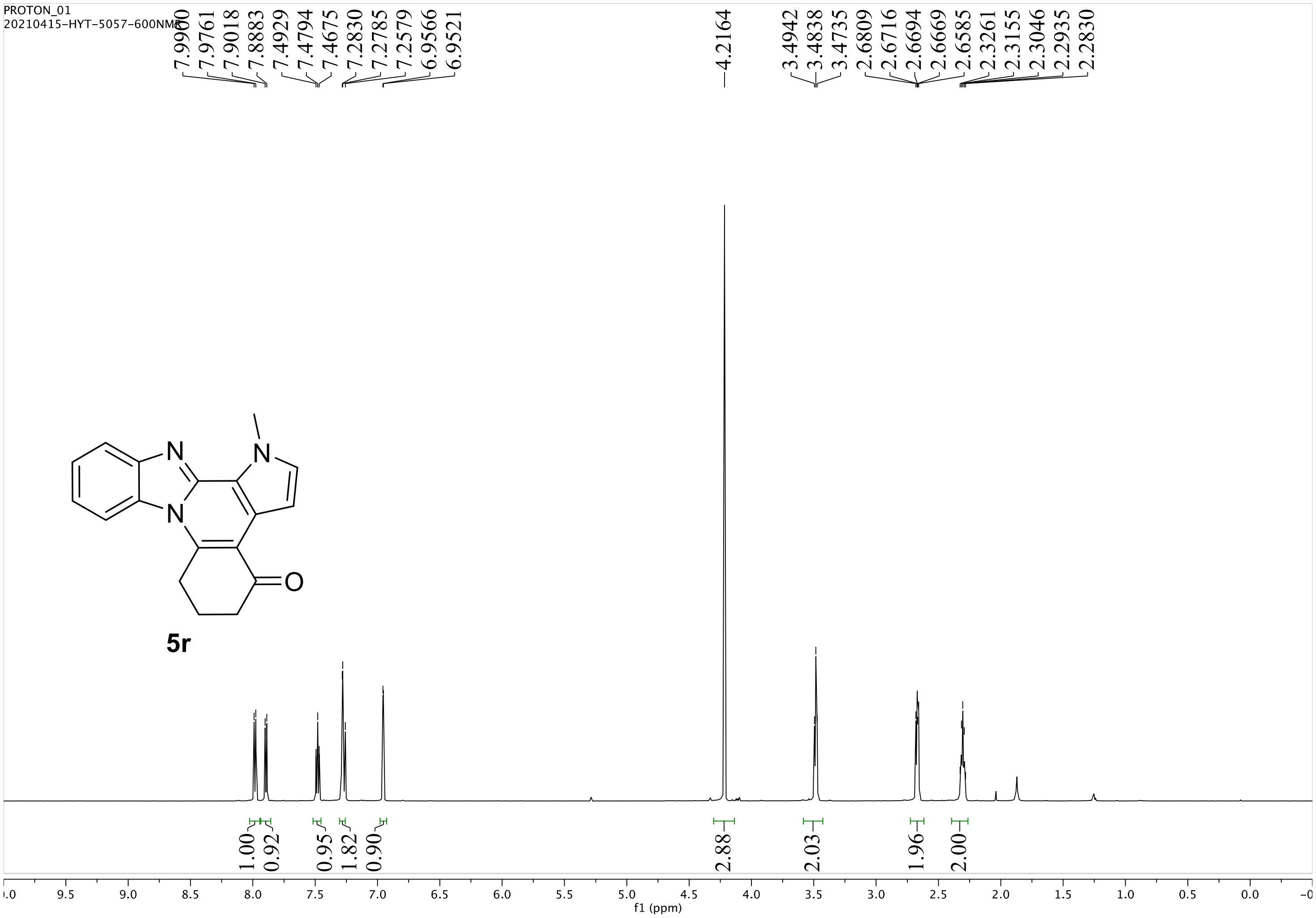

^{1}H NMR spectrum (600 MHz) of compound **5r** in $CDCl_3$.

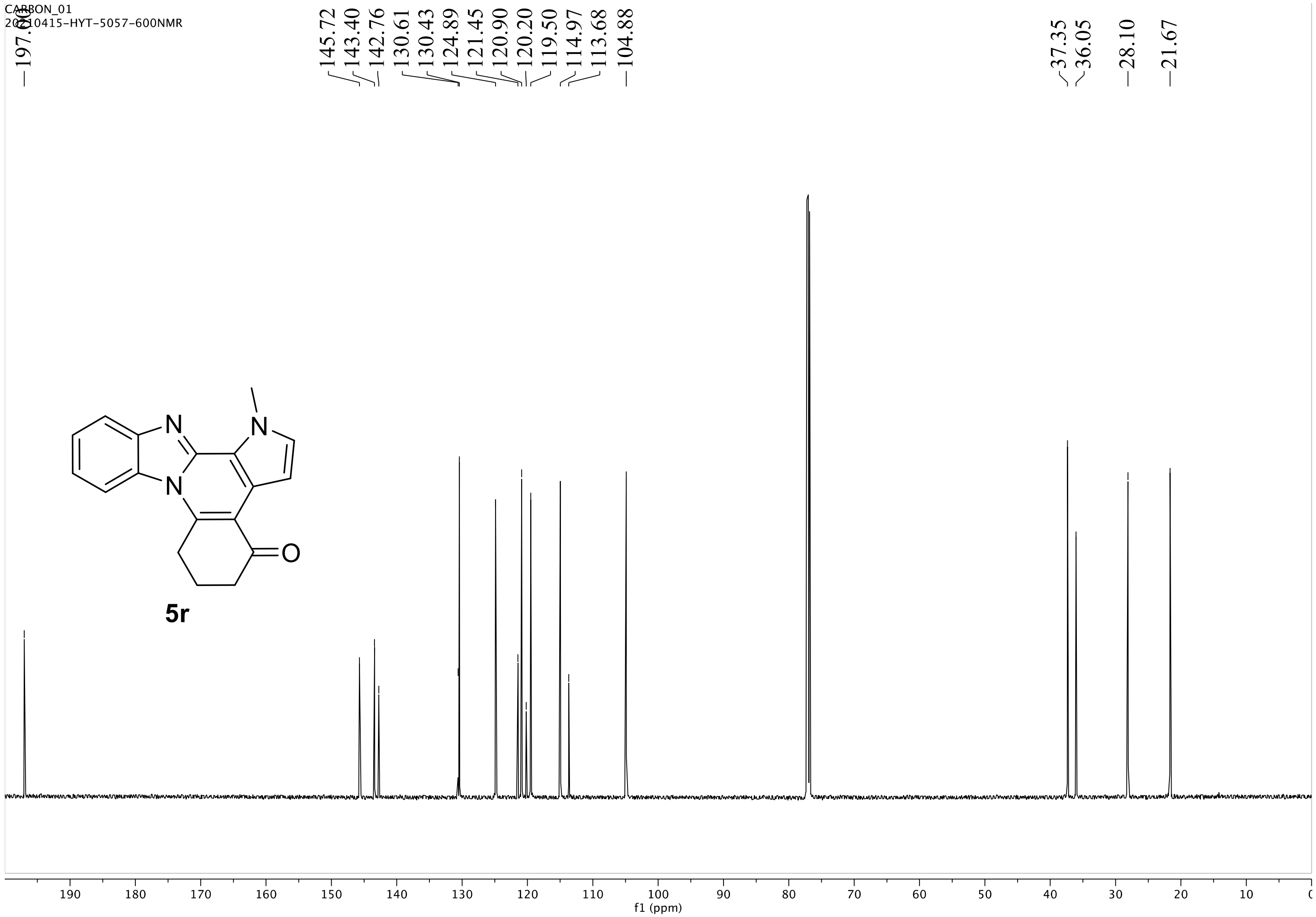

^{13}C{1H} NMR spectrum (150 MHz) of compound **5r** in $CDCl_3$.

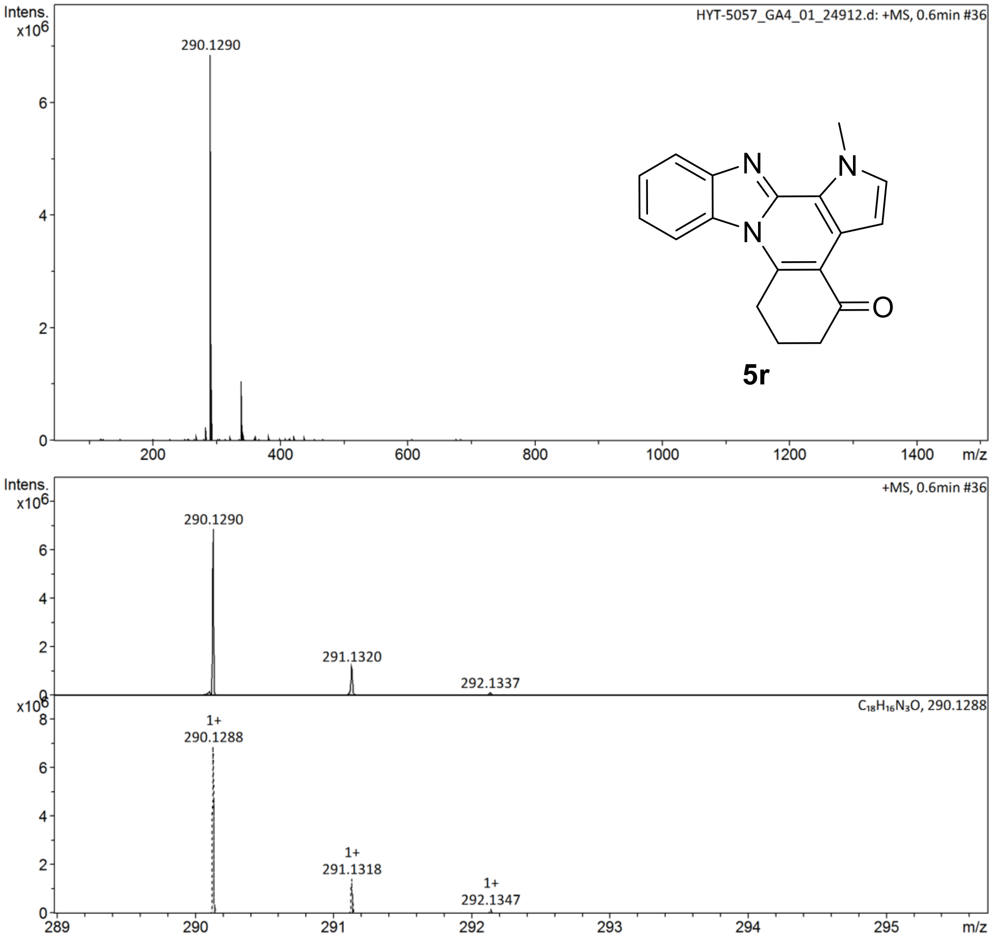

Display Report

Meas. m/z	#	Ion Formula	m/z	err [ppm]	mSigma	# Sigma	Score	rdb	e⁻ Conf	N-Rule	Adduct
290.1290	1	C18H16N3O	290.1288	0.6	19.3	1	100.00	12.5	even	ok	M+H

HRMS (ESI) of compound **5r**

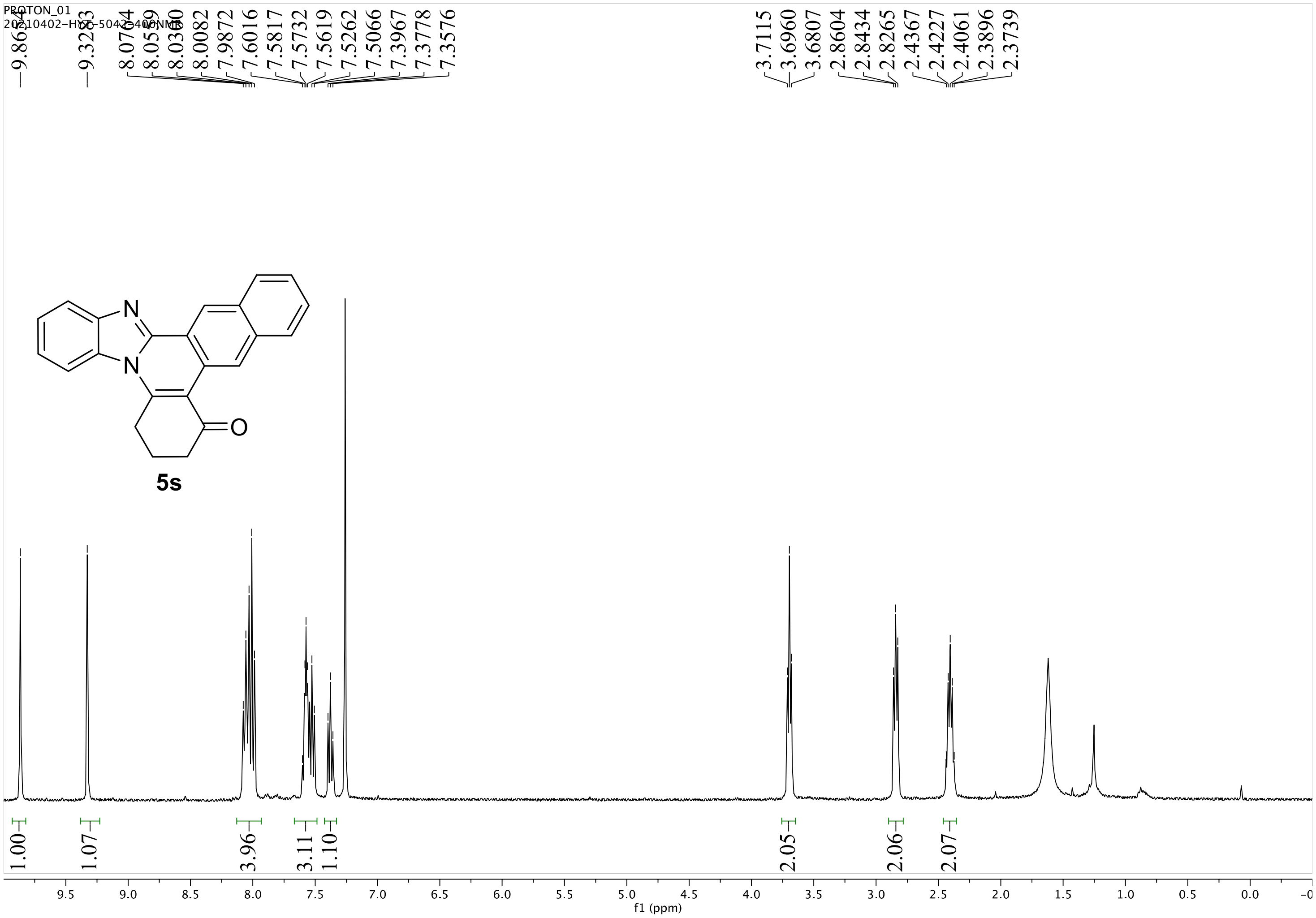

^{1}H NMR spectrum (400 MHz) of compound **5s** in $CDCl_3$.

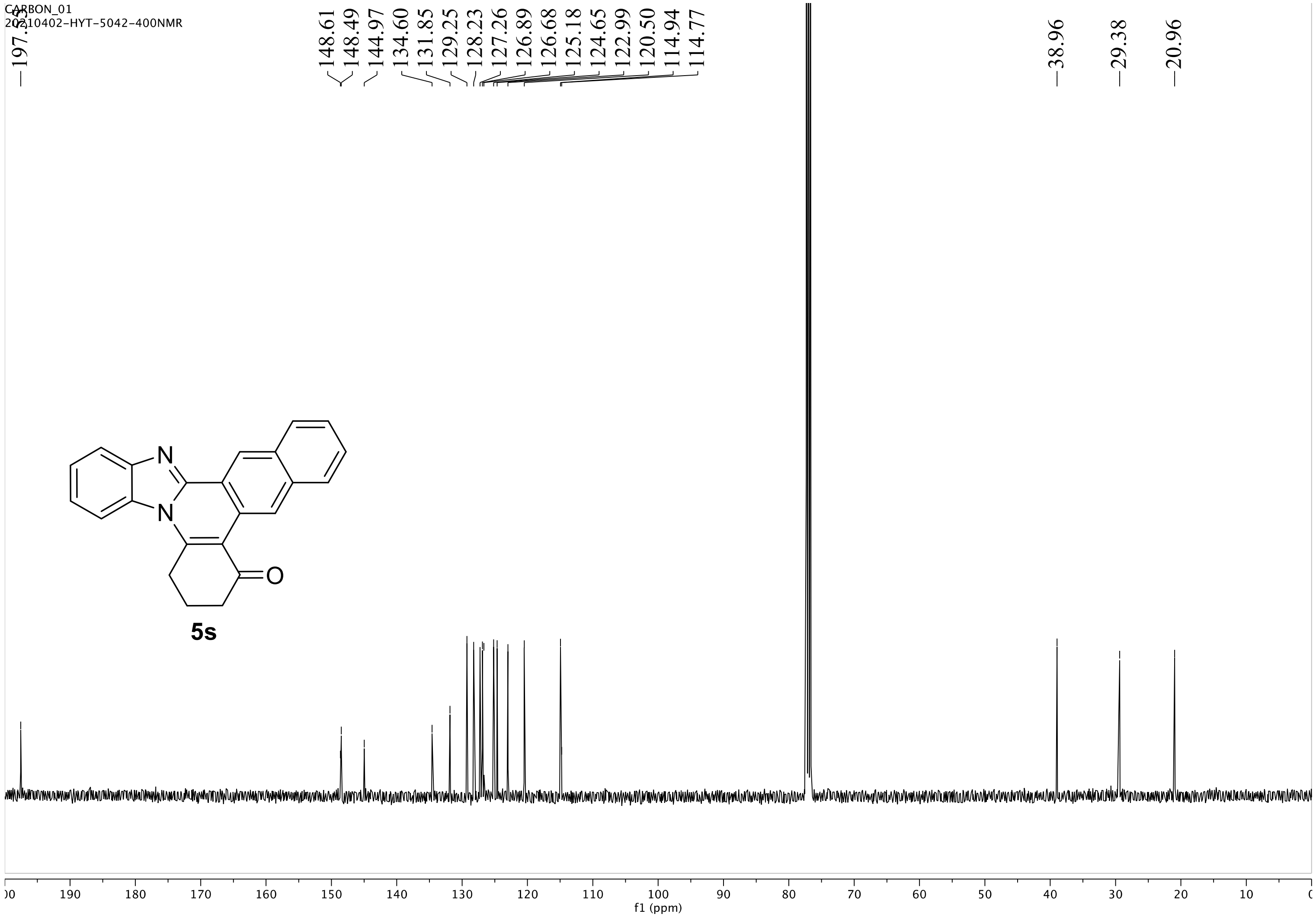

$^{13}C\{1H\}$ NMR spectrum (100 MHz) of compound **5s** in $CDCl_3$.

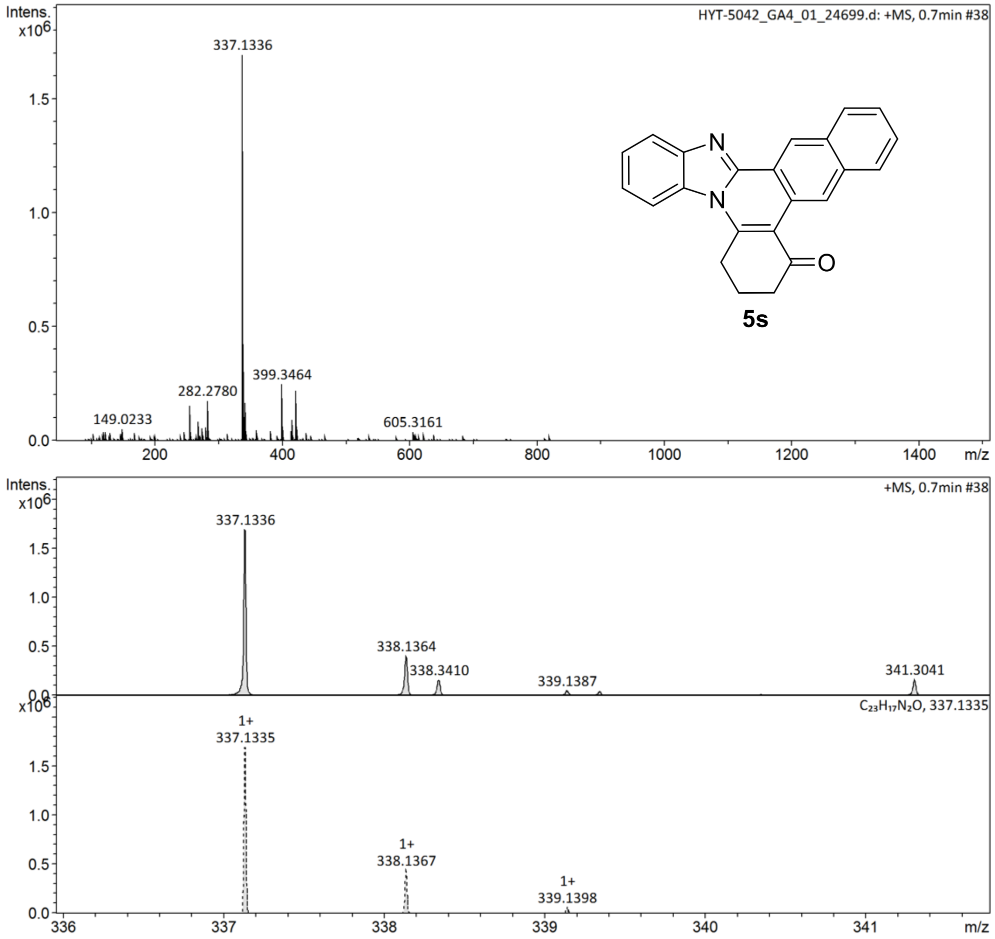

Display Report

Meas. m/z	#	Ion Formula	m/z	err [ppm]	mSigma	# Sigma	Score	rdb	e⁻ Conf	N-Rule	Adduct
337.1336	1	C23H17N2O	337.1335	0.1	12.4	1	100.00	16.5	even	ok	M+H

HRMS (ESI) of compound **5s**

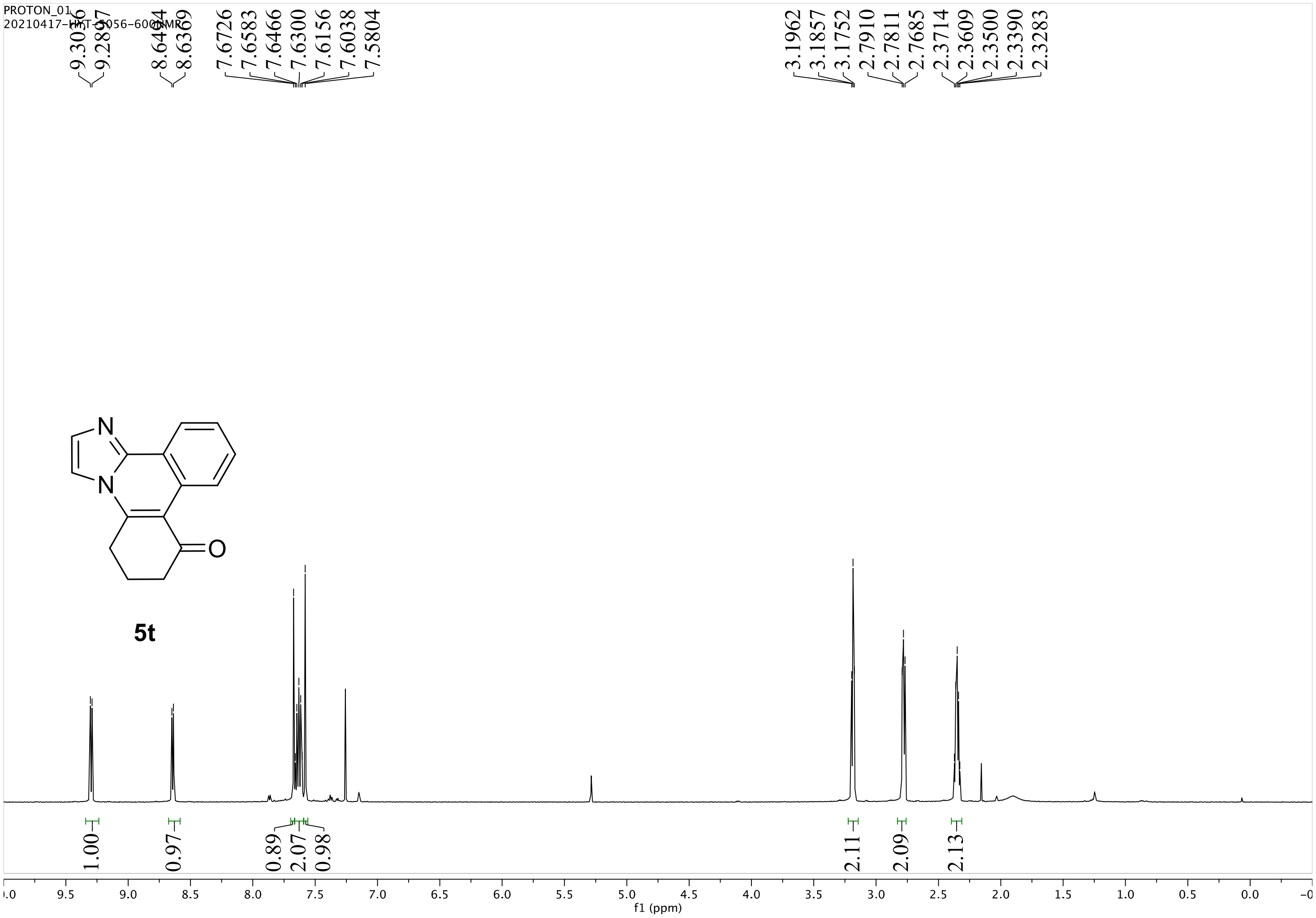

^{1}H NMR spectrum (600 MHz) of compound **5t** in $CDCl_3$.

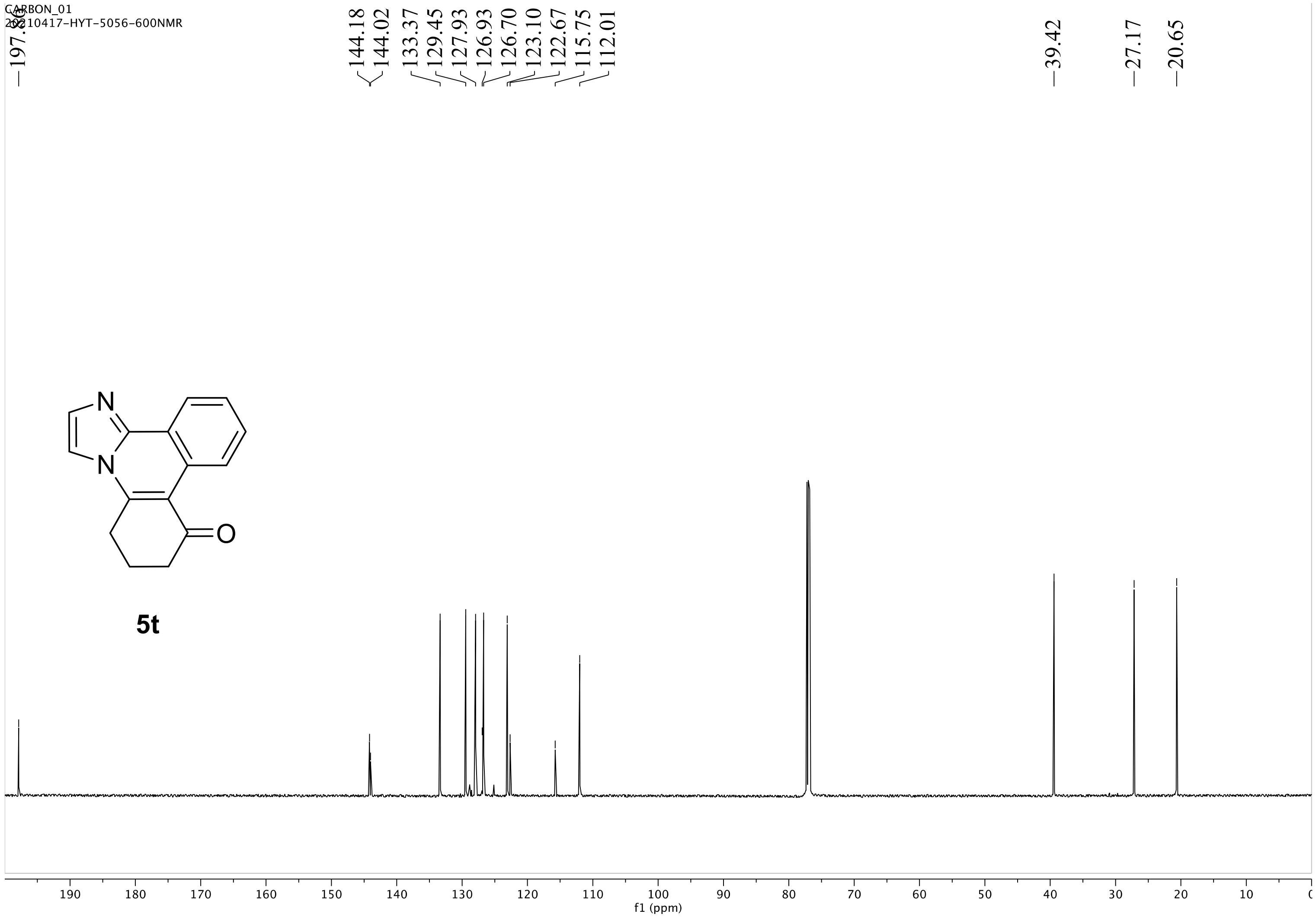

$^{13}C\{^1H\}$ NMR spectrum (150 MHz) of compound **5t** in $CDCl_3$.

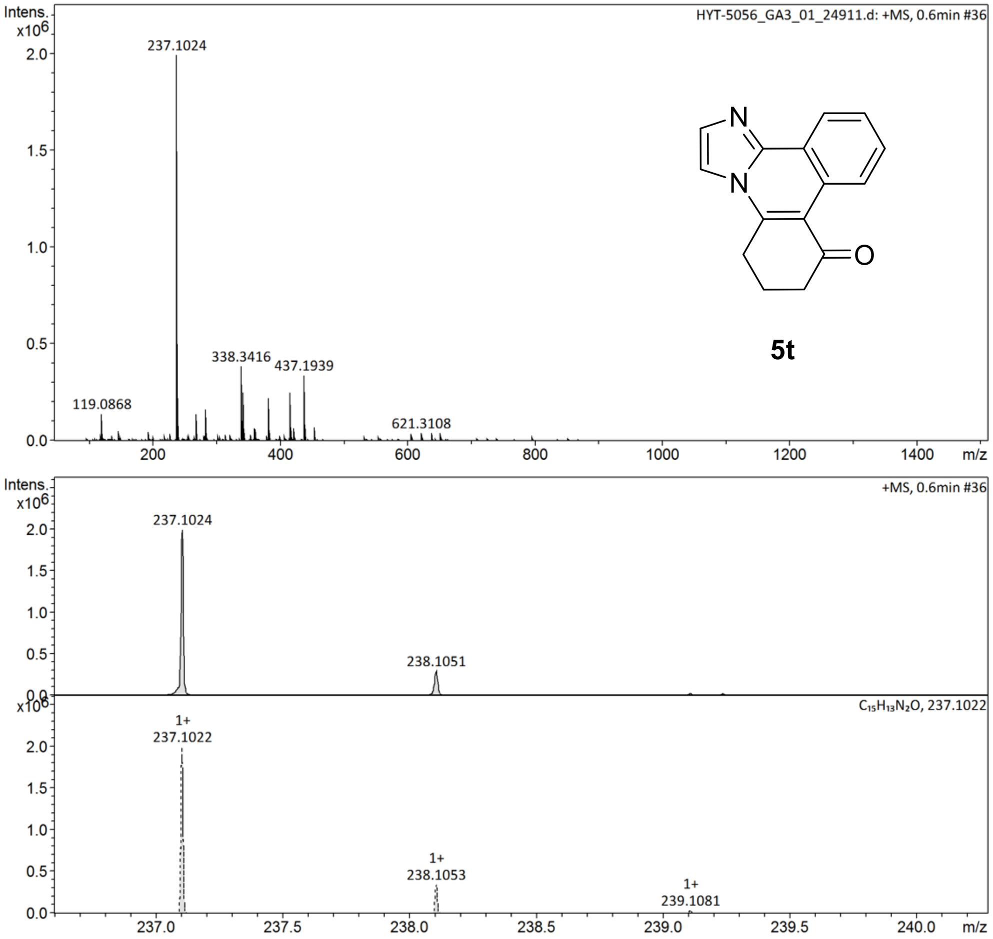

Display Report

Meas. m/z	#	Ion Formula	m/z	err [ppm]	mSigma	# Sigma	Score	rdb	e¯ Conf	N-Rule	Adduct
237.1024	1	C15H13N2O	237.1022	-0.8	13.8	1	100.00	10.5	even	ok	M+H

HRMS (ESI) of compound **5t**

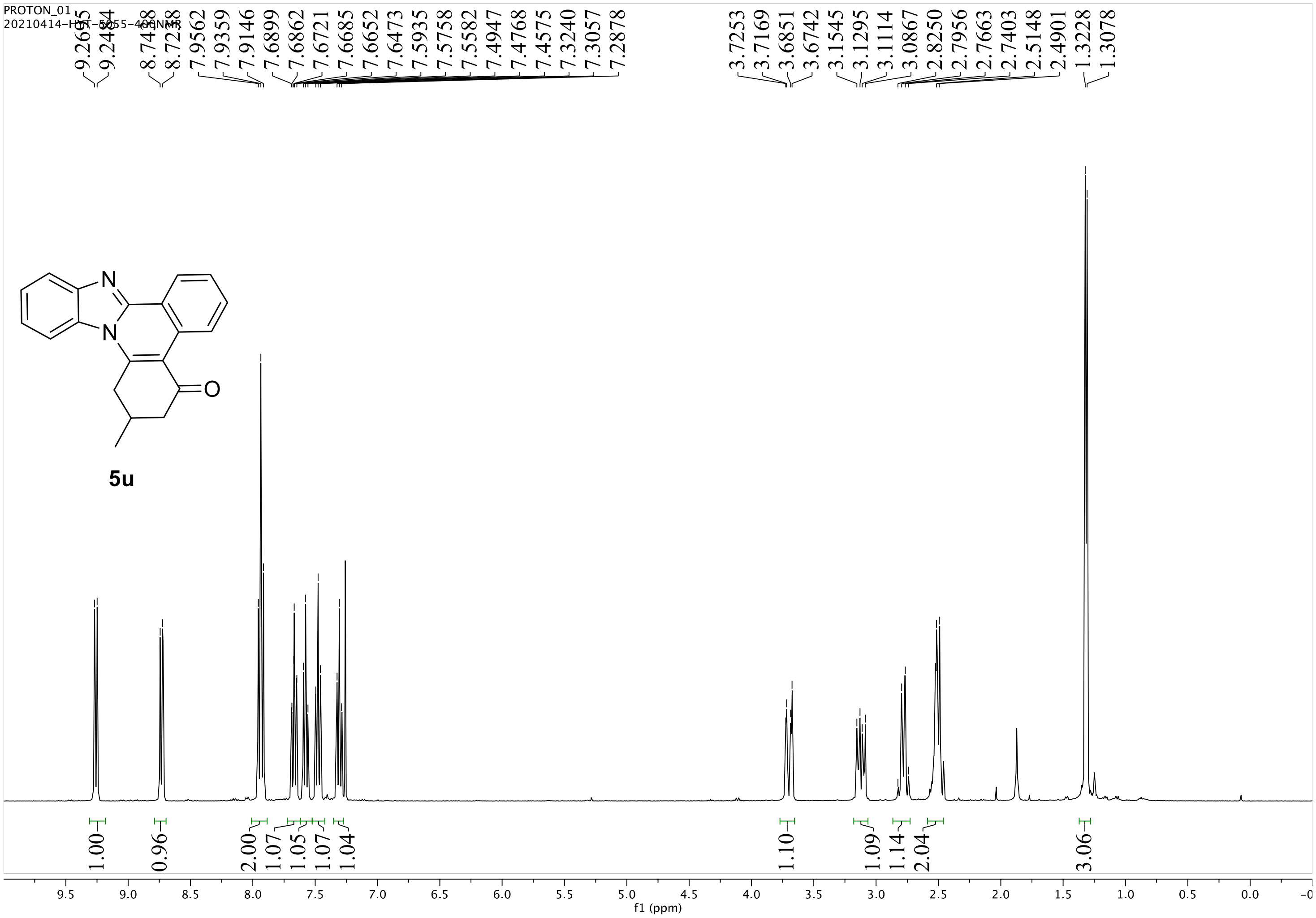

^{1}H NMR spectrum (400 MHz) of compound **5u** in $CDCl_3$

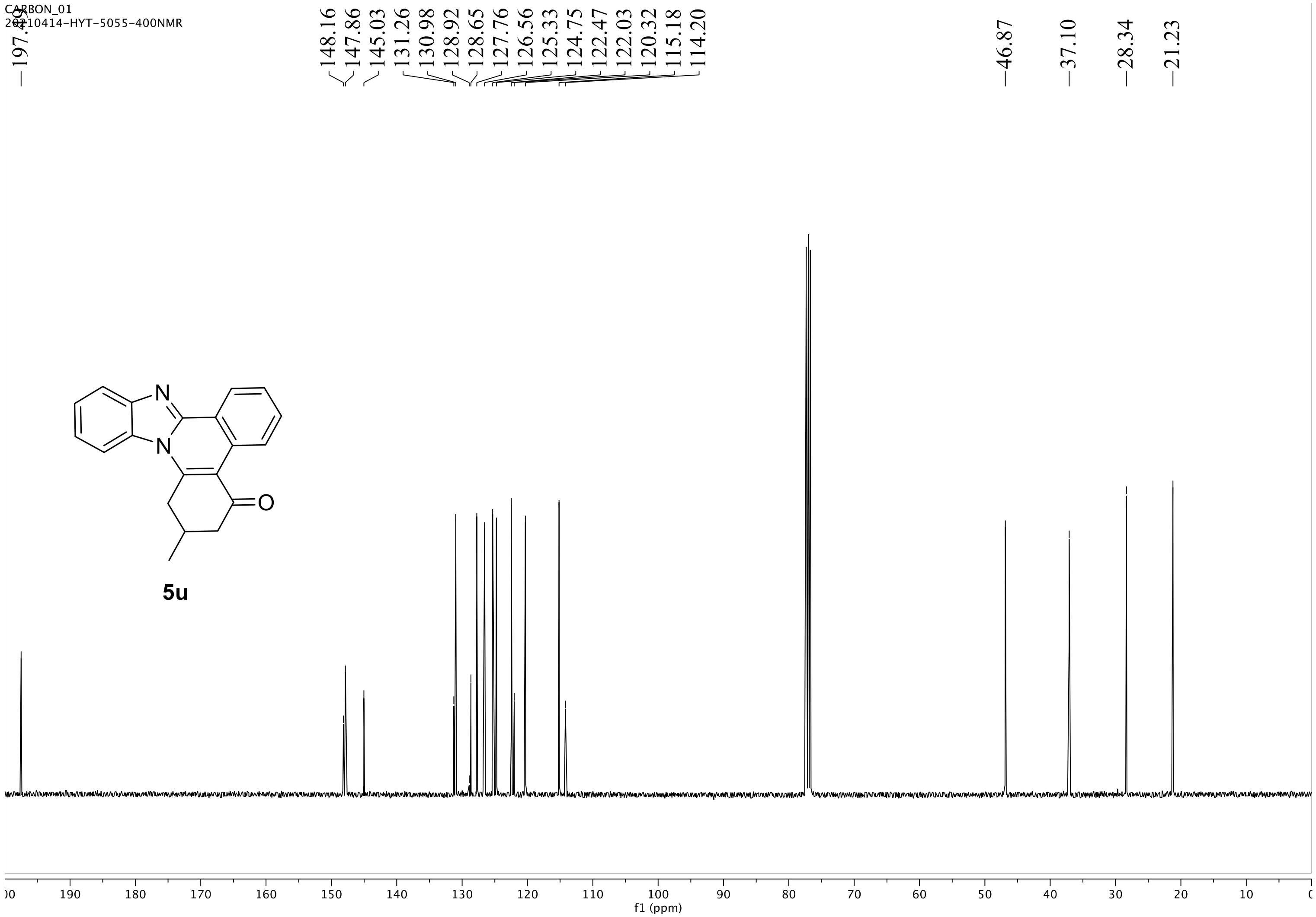

$^{13}C\{1H\}$ NMR spectrum (100 MHz) of compound **5u** in $CDCl_3$.

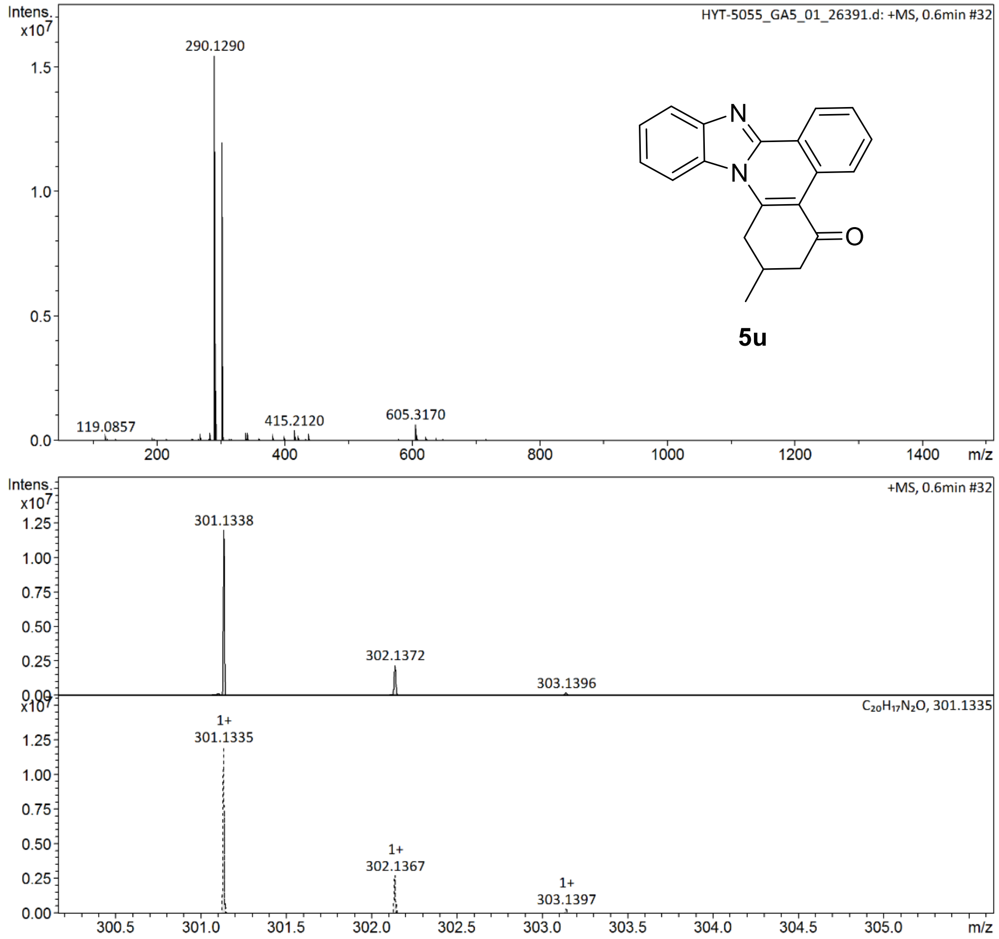

Display Report

Meas. m/z	#	Ion Formula	m/z	err [ppm]	mSigma	# Sigma	Score	rdb	e¯ Conf	N-Rule	Adduct
301.1338	1	C20H17N2O	301.1335	0.9	25.0	1	100.00	13.5	even	ok	M

HRMS (ESI) of compound **5u**

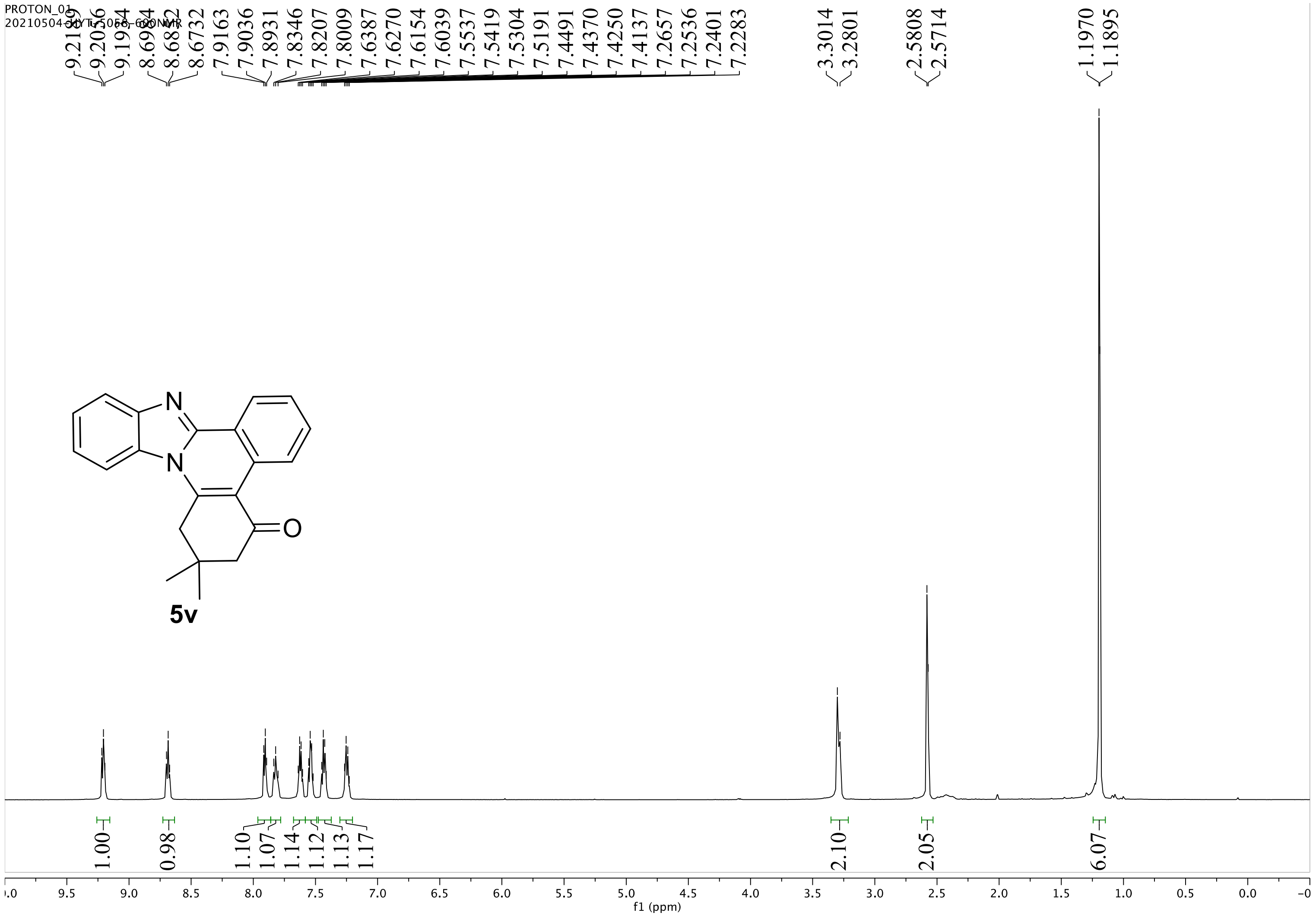

^{1}H NMR spectrum (600 MHz) of compound **5v** in $CDCl_3$

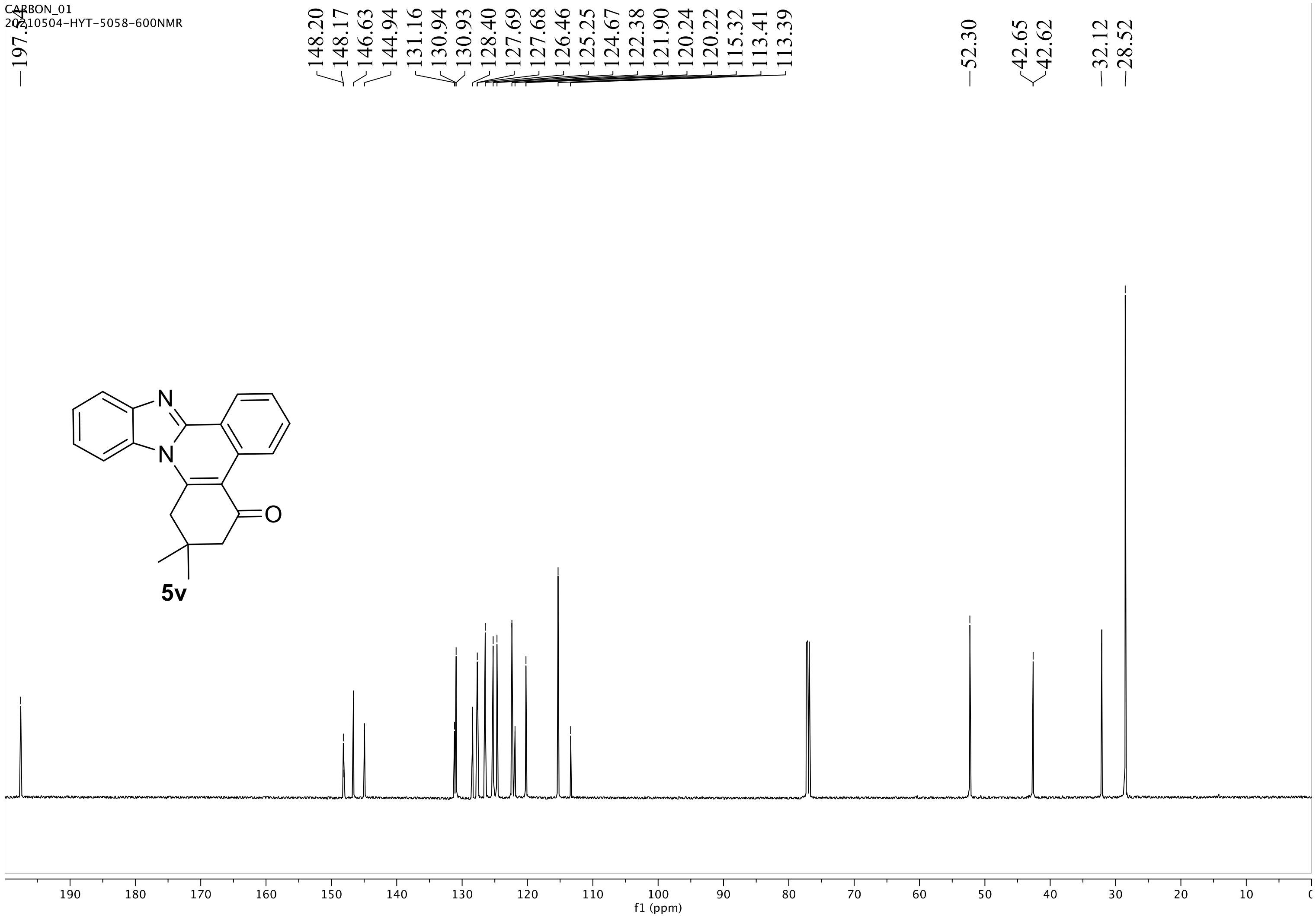

^{13}C{1H} NMR spectrum (150 MHz) of compound **5v** in $CDCl_3$.

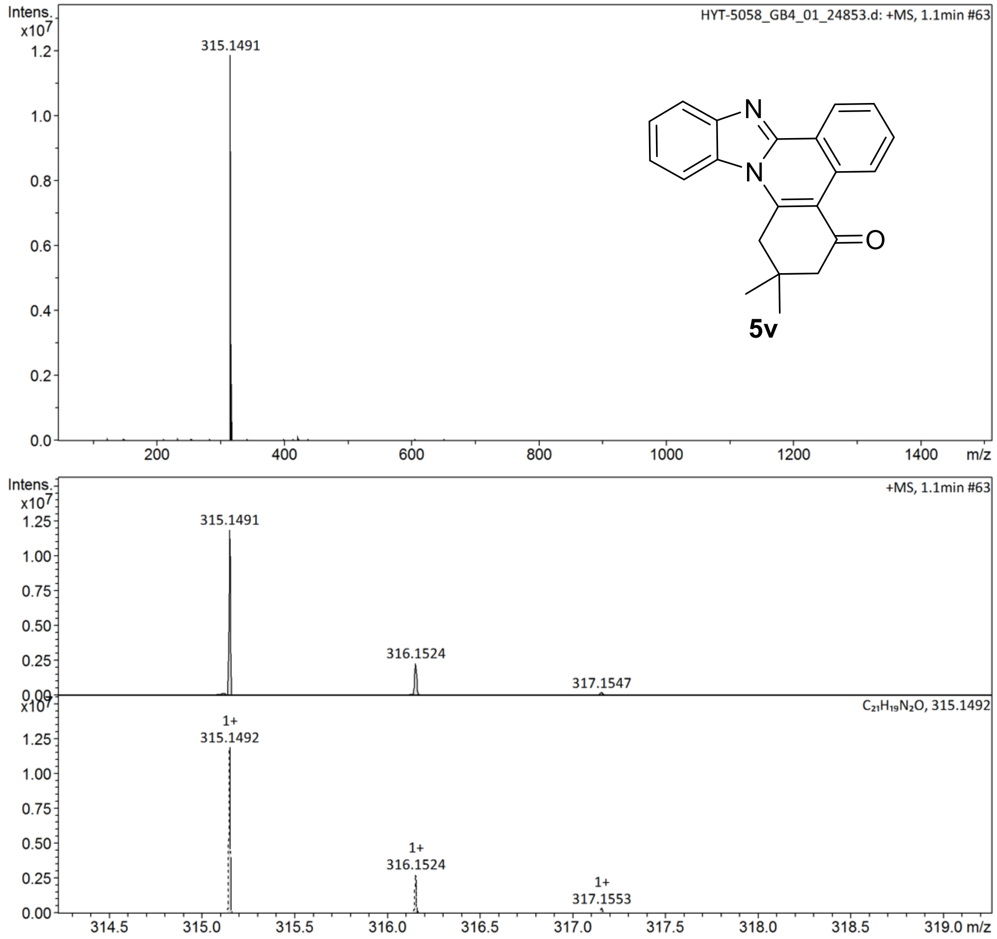

Display Report

Meas. m/z	#	Ion Formula	m/z	err [ppm]	mSigma	# Sigma	Score	rdb	e⁻ Conf	N-Rule	Adduct
315.1491	1	C21H19N2O	315.1492	0.4	24.8	1	100.00	13.5	even	ok	M+H

HRMS (ESI) of compound **5v**

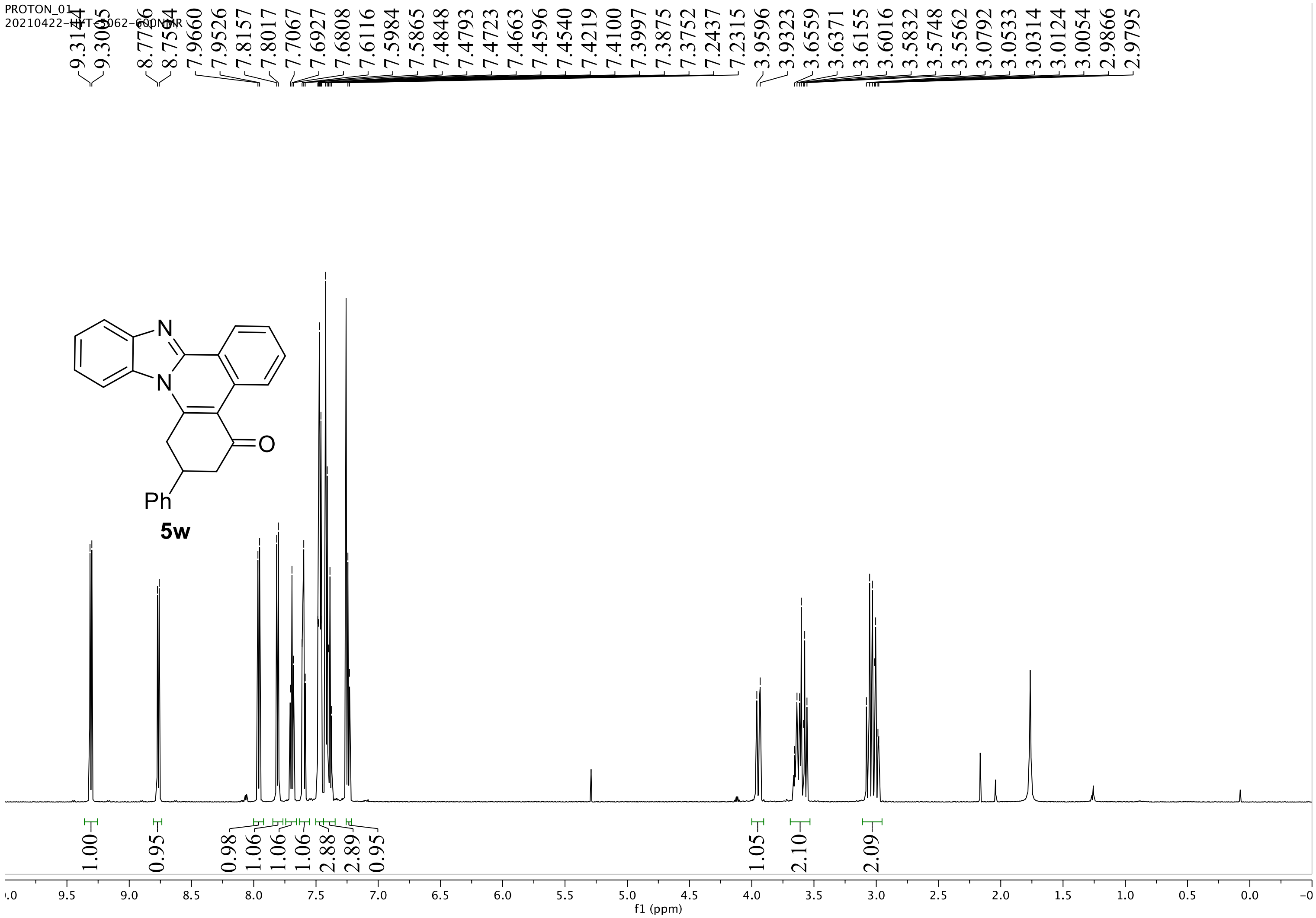

^{1}H NMR spectrum (600 MHz) of compound **5w** in $CDCl_3$.

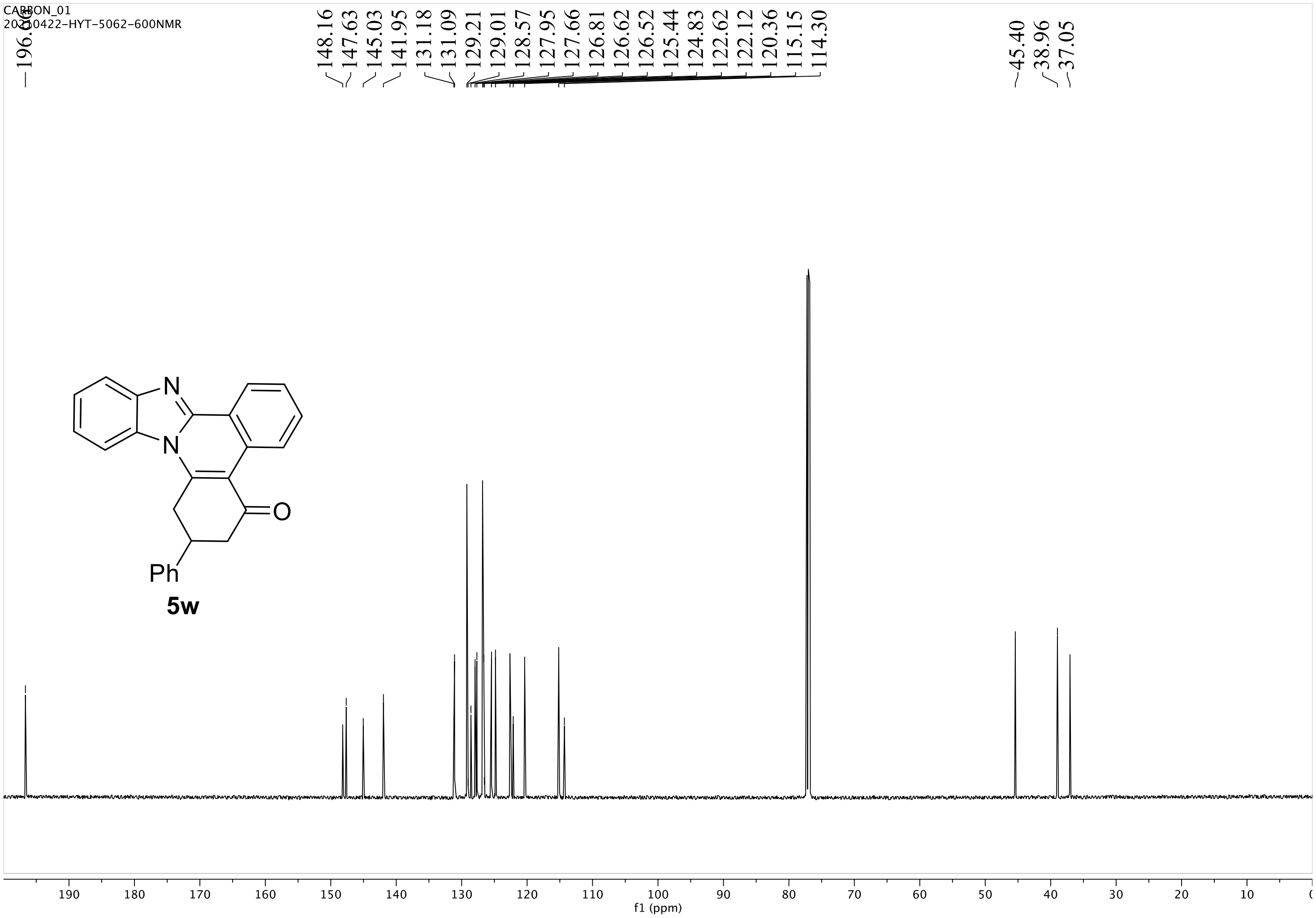

$^{13}C\{1H\}$ NMR spectrum (150 MHz) of compound **5w** in $CDCl_3$.

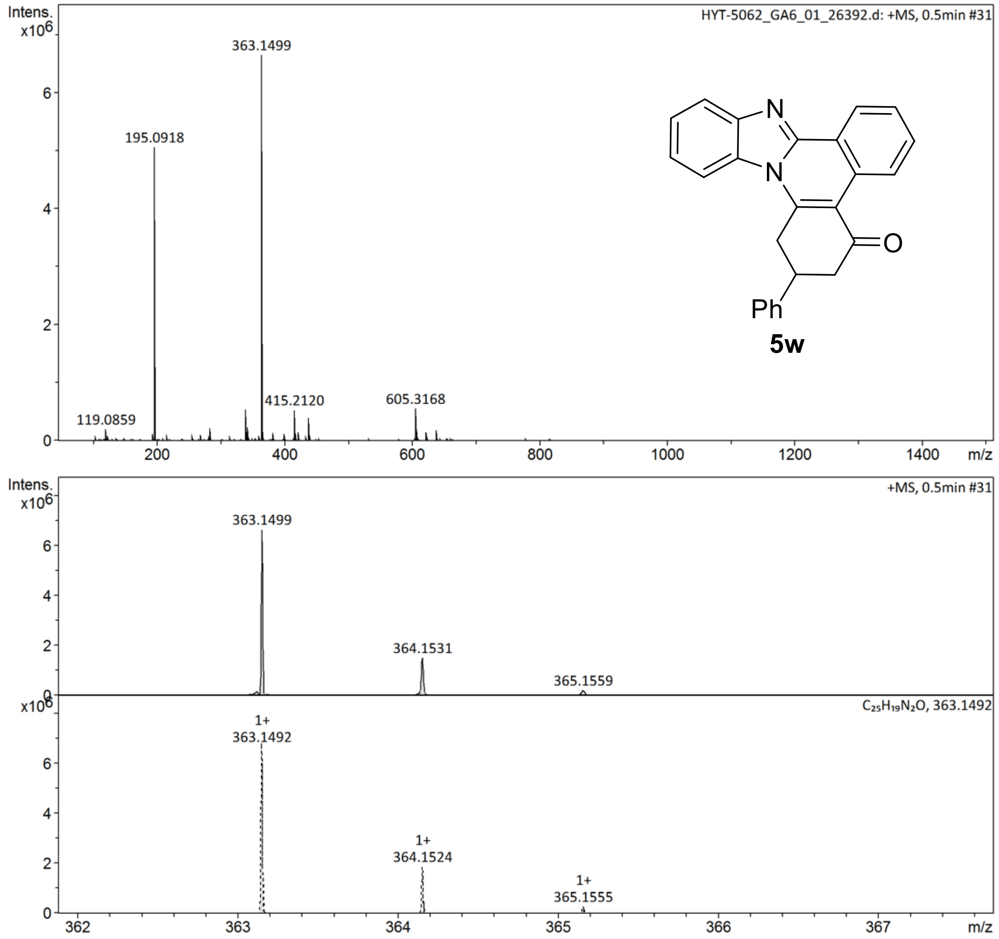

Display Report

Meas. m/z	#	Ion Formula	m/z	err [ppm]	mSigma	# Sigma	Score	rdb	e⁻ Conf	N-Rule	Adduct
363.1499	1	C25H19N2O	363.1492	-1.8	27.8	1	100.00	17.5	even	ok	M

HRMS (ESI) of compound **5w**

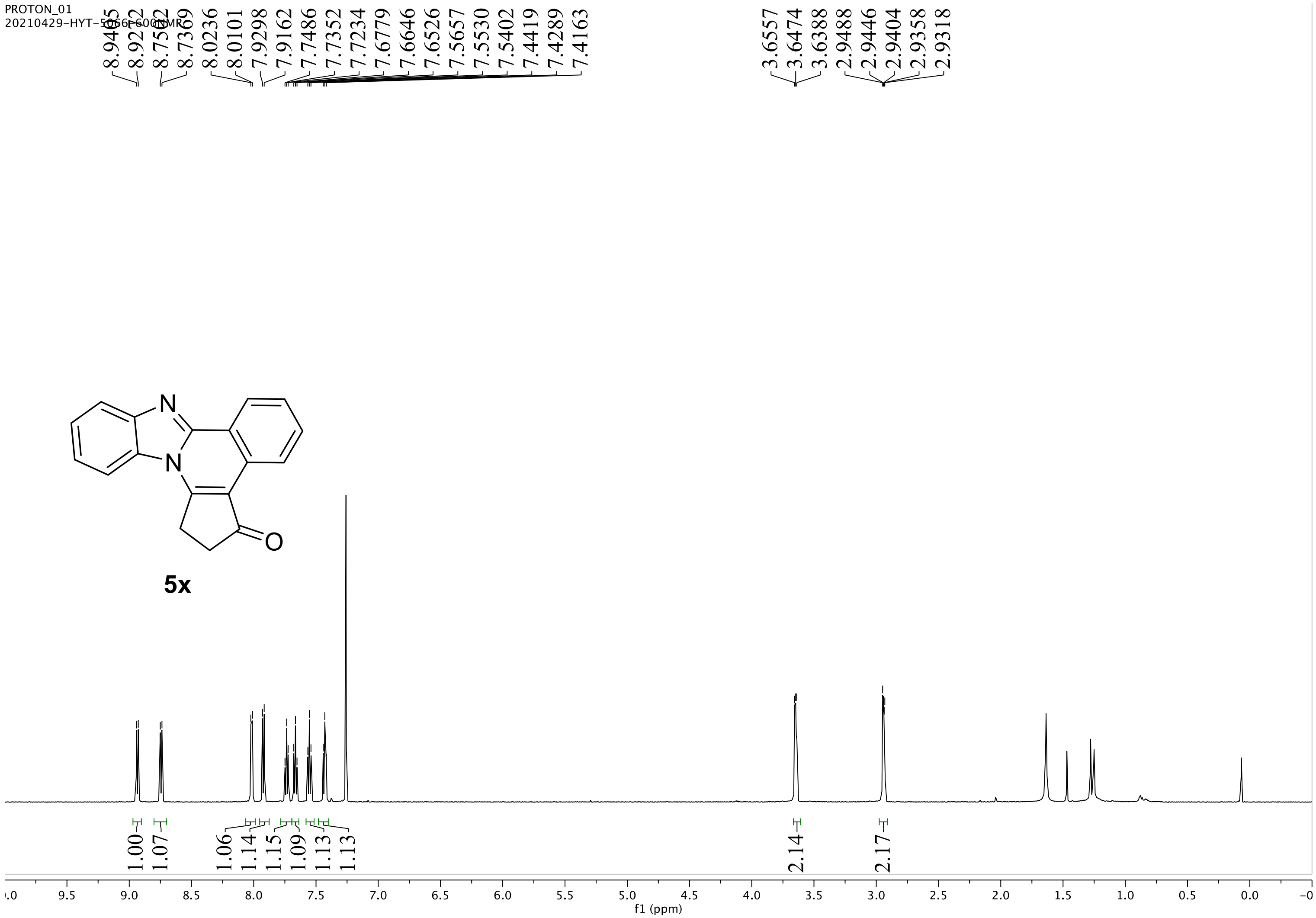

^{1}H NMR spectrum (600 MHz) of compound **5x** in $CDCl_3$.

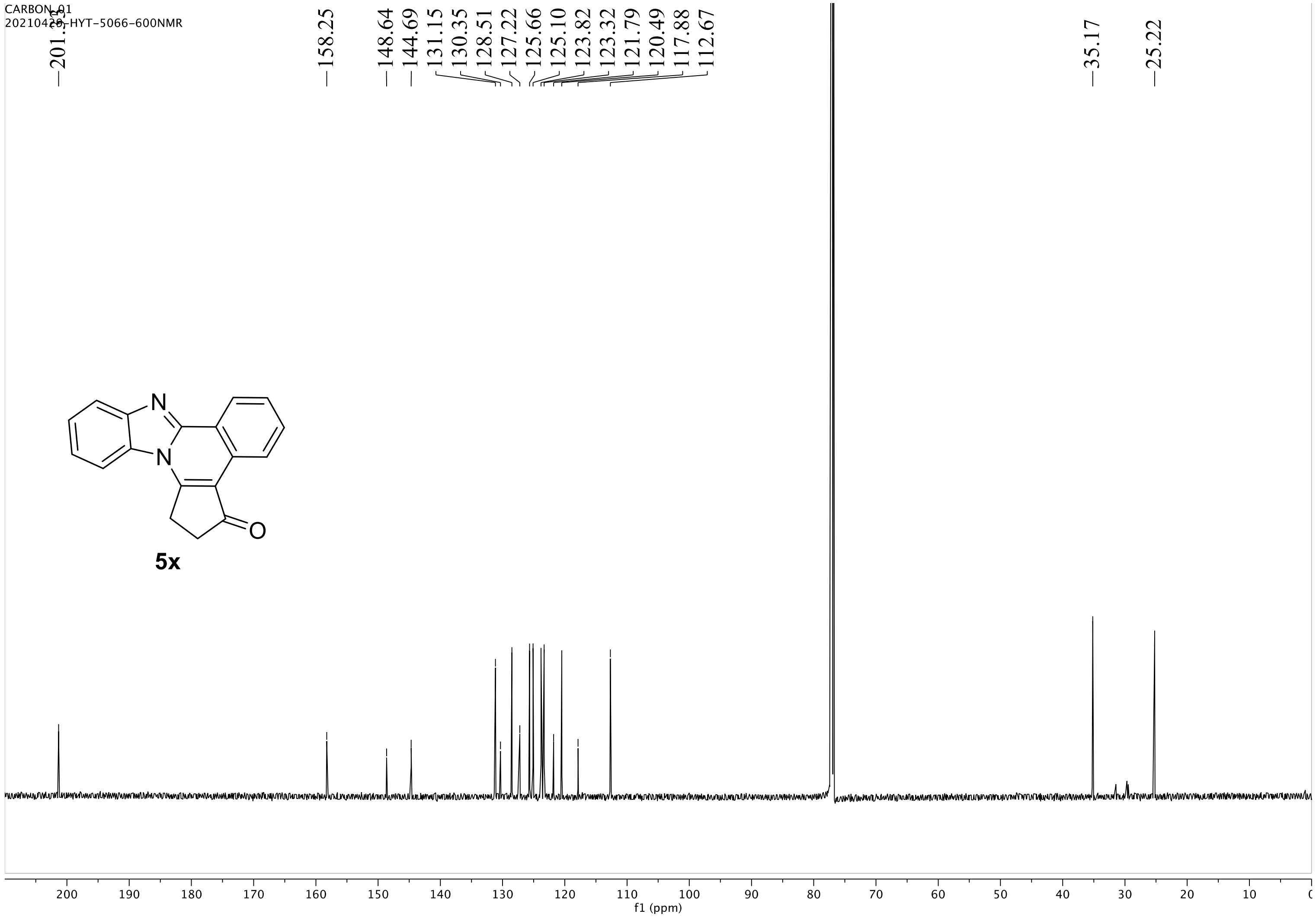

$^{13}C\{1H\}$ NMR spectrum (150 MHz) of compound **5x** in $CDCl_3$.

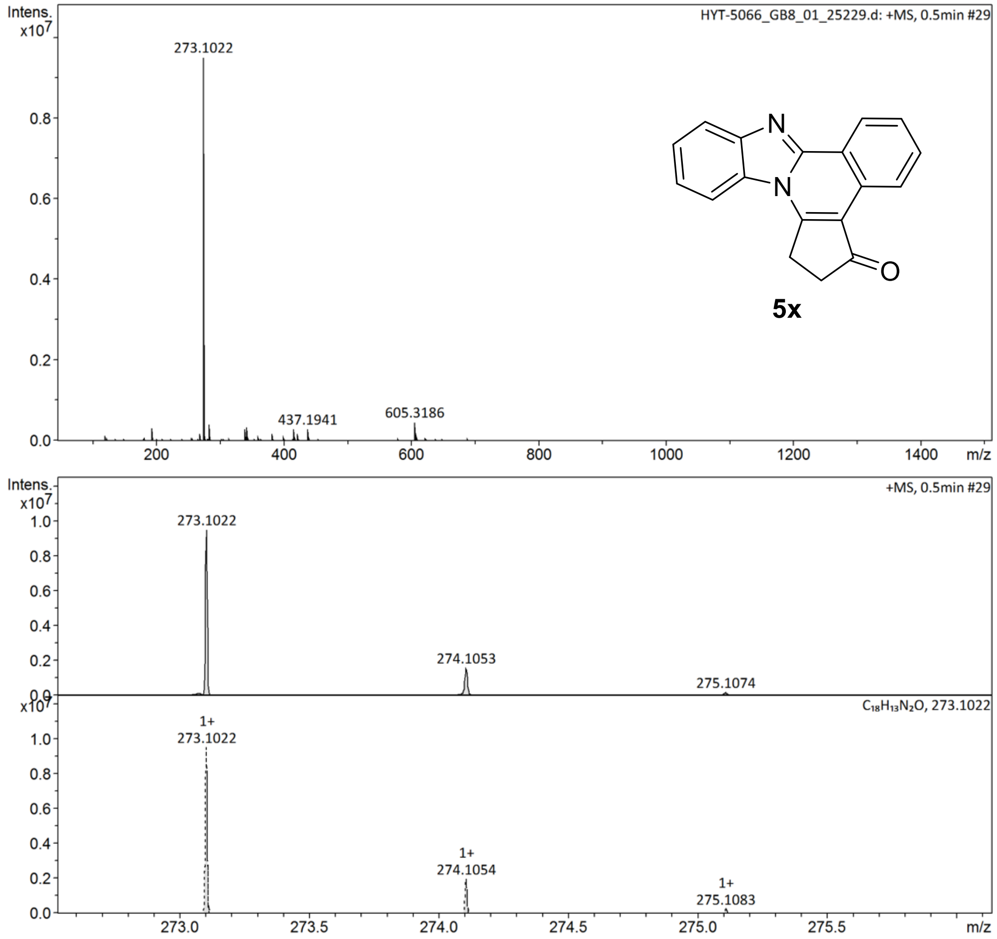

Display Report

Meas. m/z	#	Ion Formula	m/z	err [ppm]	mSigma	# Sigma	Score	rdb	e⁻ Conf	N-Rule	Adduct
273.1022	1	C18H13N2O	273.1022	-0.1	23.0	1	100.00	13.5	even	ok	M+H

HRMS (ESI) of compound **5x**

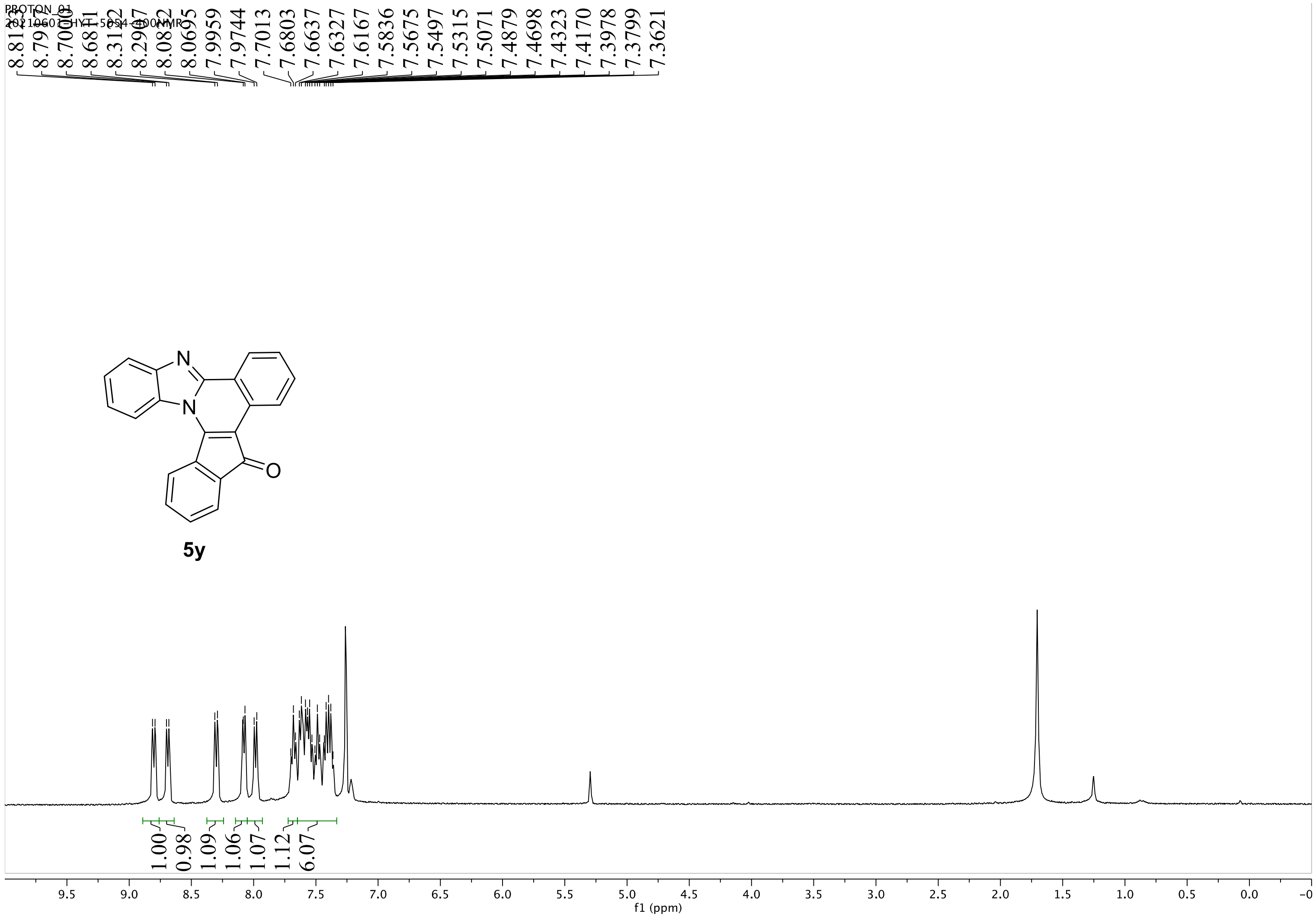

^{1}H NMR spectrum (400 MHz) of compound **5y** in $CDCl_3$.

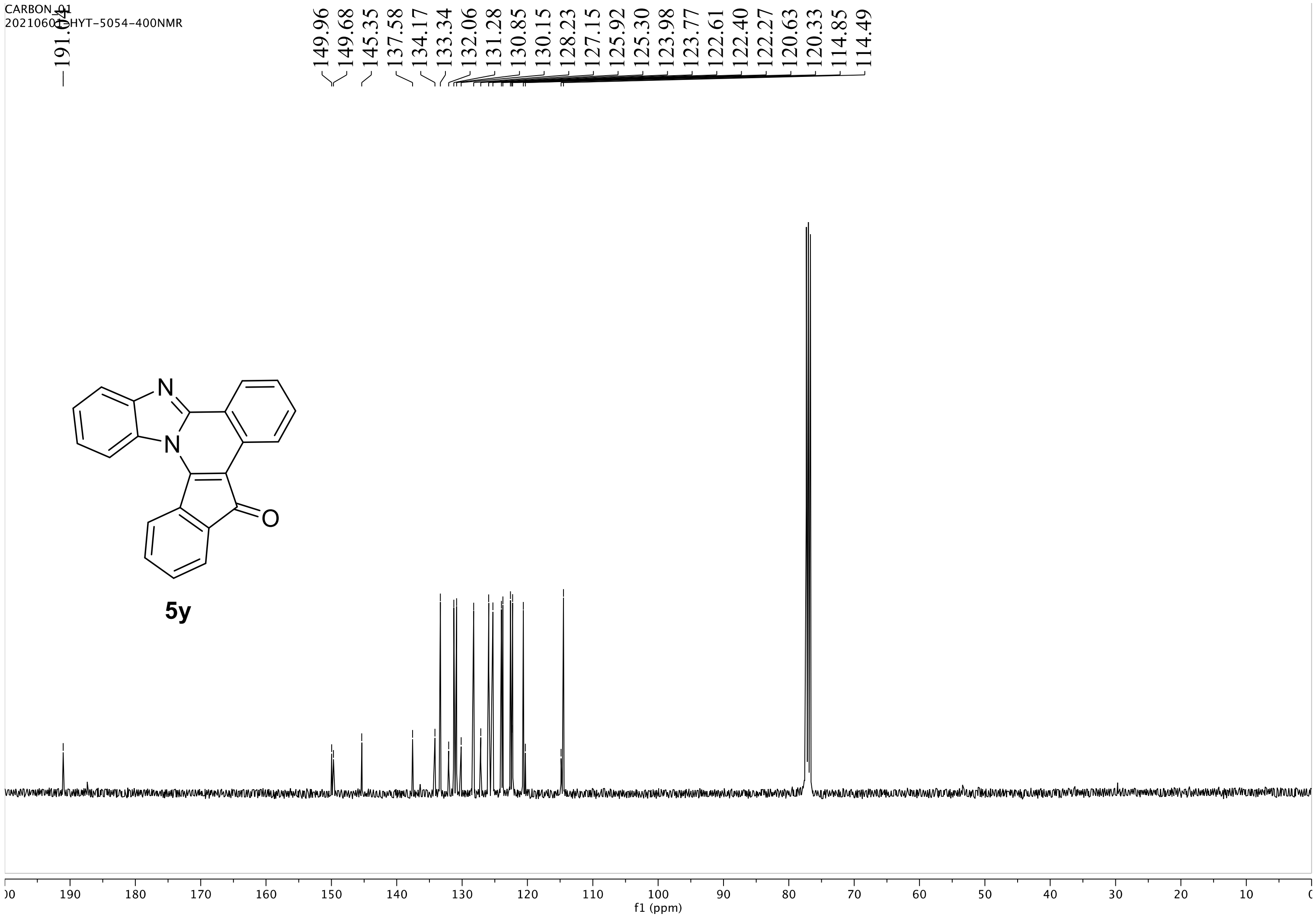

^{13}C{1H} NMR spectrum (100 MHz) of compound **5y** in $CDCl_3$.

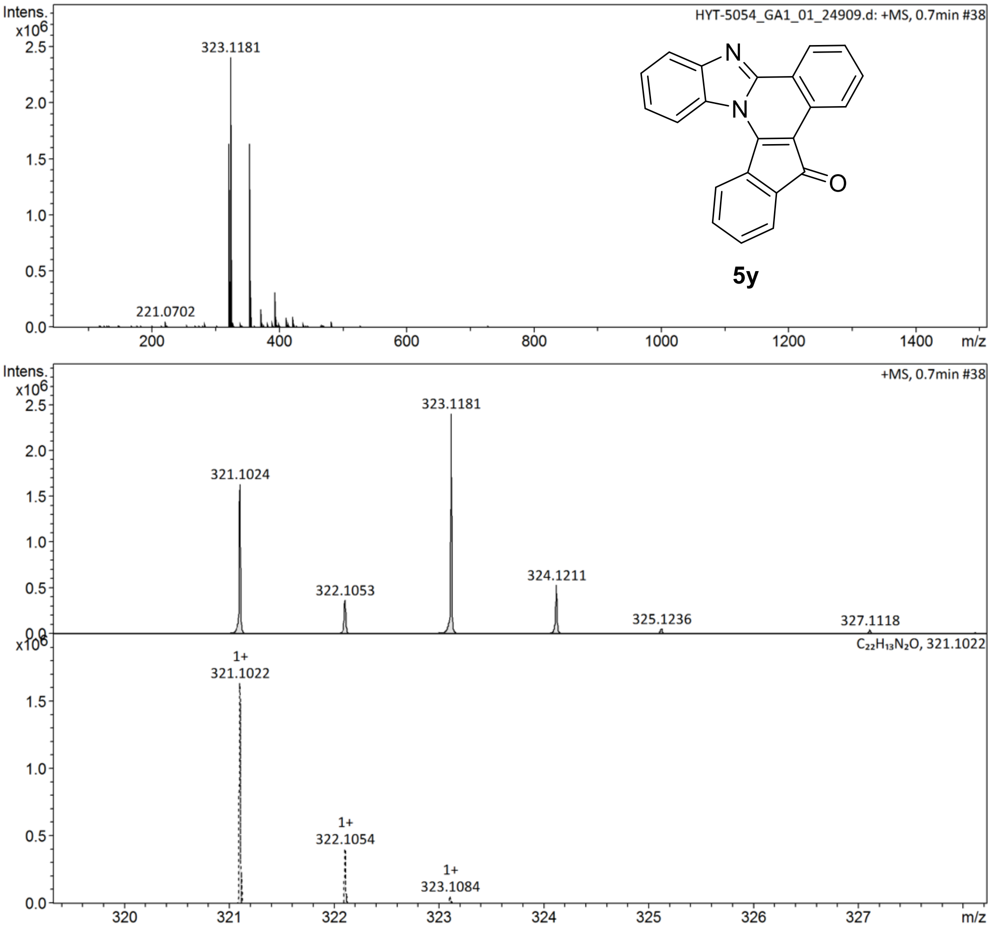

Display Report

Meas. m/z	#	Ion Formula	m/z	err [ppm]	mSigma	# Sigma	Score	rdb	e¯ Conf	N-Rule	Adduct
321.1024	1	C22H13N2O	321.1022	0.5	21.7	1	100.00	17.5	even	ok	M+H

HRMS (ESI) of compound **5y**

X-ray crystallographic data of compound 3h

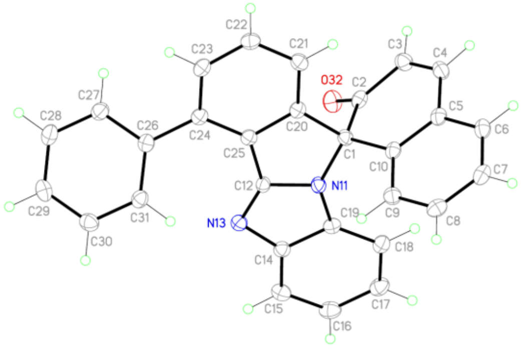

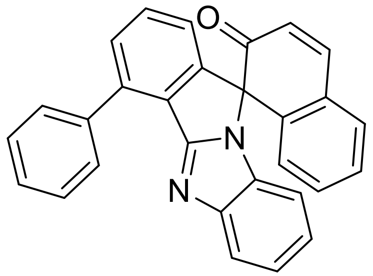

3h

ORTEP diagram of compound 3h. Atomic displacement ellipsoids are drawn at the 50% probability level

Table S1 Crystal data and structure refinement for 221293lt_auto.

Identification code	221293lt_auto
Empirical formula	$C_{29}H_{18}N_2O$
Formula weight	410.45
Temperature/K	100.00(10)
Crystal system	orthorhombic
Space group	Pbca

a/Å	15.08430(10)
b/Å	16.37780(10)
c/Å	16.4285(2)
α/°	90
β/°	90
γ/°	90
Volume/$Å^3$	4058.62(6)
Z	8
ρ_{calc}g/cm^3	1.343
μ/mm^{-1}	0.644
F(000)	1712.0
Crystal size/mm^3	0.23 × 0.22 × 0.2
Radiation	Cu Kα (λ = 1.54184)
2Θ range for data collection/°	9.62 to 134.144
Index ranges	-18 ≤ h ≤ 16, -19 ≤ k ≤ 19, -19 ≤ l ≤ 19
Reflections collected	39977
Independent reflections	3627 [R_{int} = 0.0293, R_{sigma} = 0.0145]
Data/restraints/parameters	3627/0/290
Goodness-of-fit on F^2	1.032

Final R indexes [I>=2σ (I)]	R_1 = 0.0315, wR_2 = 0.0779
Final R indexes [all data]	R_1 = 0.0329, wR_2 = 0.0788
Largest diff. peak/hole / e Å^{-3}	0.26/-0.16

Table S2 Fractional Atomic Coordinates (×10^4) and Equivalent Isotropic Displacement Parameters (Å^2×10^3) for 221293lt_auto. U_{eq} is defined as 1/3 of the trace of the orthogonalised U_{IJ} tensor.

Atom	*x*	*y*	*z*	U(eq)
C1	6853.8(7)	3024.3(7)	3372.3(7)	16.5(2)
C2	7087.7(7)	2099.8(7)	3457.1(7)	18.3(2)
C3	6952.2(7)	1596.5(7)	2736.3(7)	21.0(3)
C4	6896.4(7)	1934.1(7)	1993.8(7)	20.3(2)
C5	6953.1(7)	2815.4(7)	1853.6(7)	17.7(2)

Table S2 Fractional Atomic Coordinates (×10⁴) and Equivalent Isotropic Displacement Parameters (Å²×10³) for 221293lt_auto. U_{eq} is defined as 1/3 of the trace of the orthogonalised U_{IJ} tensor.

Atom	*x*	*y*	*z*	U(eq)
C6	7047.9(7)	3126.3(7)	1065.9(7)	20.2(2)
C7	7162.2(7)	3958.5(7)	938.9(7)	21.3(3)
C8	7165.0(7)	4487.9(7)	1598.8(7)	21.9(3)
C9	7054.5(7)	4190.5(7)	2384.4(7)	19.9(2)
C10	6954.3(7)	3356.8(7)	2515.6(7)	16.7(2)
C12	6878.5(7)	3693.2(6)	4654.0(6)	15.2(2)
C14	8243.0(7)	3914.8(7)	4932.1(7)	17.2(2)
C15	9057.6(8)	4097.6(7)	5292.3(7)	22.0(3)
C16	9820.5(8)	3971.5(7)	4840.2(8)	23.8(3)
C17	9787.8(8)	3684.5(7)	4036.1(8)	22.8(3)
C18	8989.7(7)	3501.8(7)	3662.0(7)	19.9(2)
C19	8231.1(7)	3609.8(6)	4128.0(7)	17.0(2)
C20	5917.9(7)	3100.8(6)	3733.9(7)	16.4(2)
C21	5146.9(7)	2788.4(7)	3406.4(7)	19.2(2)
C22	4377.6(7)	2855.3(7)	3868.0(7)	20.7(2)
C23	4389.8(7)	3227.4(7)	4627.5(7)	19.5(2)
C24	5166.6(7)	3555.1(6)	4968.0(7)	16.8(2)
C25	5944.7(7)	3480.2(6)	4503.3(7)	16.0(2)

Table S2 Fractional Atomic Coordinates (×10[4]) and Equivalent Isotropic Displacement Parameters (Å^2×10^3) for 221293lt_auto. U_{eq} is defined as 1/3 of the trace of the orthogonalised U_{IJ} tensor.

Atom	*x*	*y*	*z*	U(eq)
C26	5123.9(7)	3955.4(7)	5779.9(7)	17.6(2)
C27	4557.9(7)	3641.7(7)	6380.3(7)	20.8(2)
C28	4488.6(8)	4013.2(7)	7135.2(7)	24.1(3)
C29	4983.8(8)	4707.0(7)	7310.1(7)	24.1(3)
C30	5540.3(8)	5027.8(7)	6718.3(7)	22.3(3)
C31	5610.7(7)	4659.8(7)	5960.5(7)	18.9(2)
N11	7344.7(6)	3488.6(6)	3967.0(5)	16.2(2)
N13	7380.8(6)	3968.6(6)	5247.8(5)	17.1(2)
O32	7343.7(6)	1835.9(5)	4107.5(5)	24.4(2)

Table S3 Anisotropic Displacement Parameters (Å^2×10^3) for 221293lt_auto. The Anisotropic displacement factor exponent takes the form: -2π^2[h^{2}a*^{2}U$_{11}$+2hka*b*U$_{12}$+…].

Atom	U_{11}	U_{22}	U_{33}	U_{23}	U_{13}	U_{12}
C1	15.6(5)	19.2(5)	14.8(5)	-1.9(4)	-1.2(4)	-0.4(4)
C2	14.3(5)	22.2(6)	18.4(6)	2.1(5)	2.4(4)	0.8(4)
C3	21.9(6)	17.9(5)	23.1(6)	-1.9(5)	0.6(5)	0.9(4)
C4	18.7(5)	22.6(6)	19.5(6)	-5.2(5)	-0.4(5)	1.2(4)

Table S3 Anisotropic Displacement Parameters ($Å^2×10^3$) for 221293lt_auto. The Anisotropic displacement factor exponent takes the form: $-2\pi^2[h^2a^{*2}U_{11}+2hka^*b^*U_{12}+…]$.

Atom	U_{11}	U_{22}	U_{33}	U_{23}	U_{13}	U_{12}
C5	11.9(5)	23.6(6)	17.4(5)	-0.4(4)	-0.4(4)	1.3(4)
C6	15.6(5)	28.2(6)	16.7(6)	-1.6(5)	-0.3(4)	2.4(4)
C7	15.5(5)	30.9(6)	17.4(6)	5.4(5)	0.2(4)	1.0(5)
C8	18.2(5)	22.9(6)	24.5(6)	5.2(5)	-1.5(5)	-1.0(4)
C9	18.1(5)	22.0(6)	19.6(6)	-0.9(5)	-0.6(5)	0.0(4)
C10	12.0(5)	22.6(5)	15.6(5)	0.4(5)	-0.1(4)	0.9(4)
C12	16.9(5)	14.8(5)	13.9(5)	0.8(4)	2.1(4)	0.3(4)
C14	17.9(5)	15.6(5)	18.3(6)	0.6(4)	0.3(4)	-0.4(4)
C15	21.7(6)	21.4(6)	22.9(6)	-2.4(5)	-3.1(5)	-1.6(5)
C16	16.7(6)	22.8(6)	31.9(7)	-0.2(5)	-3.8(5)	-1.2(5)
C17	16.7(6)	22.3(6)	29.4(7)	1.4(5)	3.1(5)	0.6(4)
C18	18.9(6)	20.5(6)	20.2(6)	0.9(5)	2.5(5)	0.6(4)
C19	16.7(5)	15.9(5)	18.3(6)	2.3(4)	-1.4(4)	-0.3(4)
C20	16.9(5)	16.4(5)	16.1(5)	2.4(4)	0.0(4)	1.1(4)
C21	20.4(6)	19.6(5)	17.7(6)	-0.9(4)	-2.1(4)	-0.1(4)
C22	16.0(5)	22.0(6)	24.2(6)	0.0(5)	-2.5(5)	-1.9(4)

Table S3 Anisotropic Displacement Parameters (Å²×10³) for 221293lt_auto. The Anisotropic displacement factor exponent takes the form: -2π²[h²a*²U₁₁+2hka*b*U₁₂+…].

Atom	U_{11}	U_{22}	U_{33}	U_{23}	U_{13}	U_{12}
C23	15.9(5)	21.5(6)	21.1(6)	2.3(5)	2.2(5)	0.7(4)
C24	17.3(5)	14.7(5)	18.5(6)	2.7(4)	0.7(4)	1.2(4)
C25	17.7(5)	14.2(5)	16.2(5)	1.8(4)	-0.5(4)	0.1(4)
C26	16.3(5)	17.9(5)	18.5(6)	1.2(4)	0.7(4)	3.6(4)
C27	19.1(6)	20.1(6)	23.1(6)	0.8(5)	3.0(5)	-0.4(4)
C28	23.5(6)	27.0(6)	21.7(6)	1.2(5)	6.7(5)	1.5(5)
C29	26.2(6)	25.9(6)	20.2(6)	-4.5(5)	2.0(5)	5.7(5)
C30	21.9(6)	18.5(5)	26.4(6)	-3.1(5)	-0.5(5)	1.5(4)
C31	17.2(5)	18.3(5)	21.2(6)	1.2(4)	3.0(4)	2.1(4)
N11	14.4(5)	20.5(5)	13.8(4)	-2.1(4)	-0.2(4)	-0.4(4)
N13	17.3(5)	17.9(5)	16.2(5)	-0.3(4)	-0.4(4)	-1.2(4)
O32	28.4(5)	26.1(4)	18.8(4)	3.1(3)	-1.9(3)	4.5(3)

Table S4 Bond Lengths for 221293lt_auto.

Atom	Atom	Length/Å	Atom	Atom	Length/Å
C1	C2	1.5609(15)	C14	N13	1.4029(14)
C1	C10	1.5168(15)	C15	C16	1.3852(17)
C1	C20	1.5367(15)	C16	C17	1.4031(17)
C1	N11	1.4425(14)	C17	C18	1.3845(16)
C2	C3	1.4573(16)	C18	C19	1.3880(16)
C2	O32	1.2156(14)	C19	N11	1.3774(14)
C3	C4	1.3418(16)	C20	C21	1.3798(16)
C4	C5	1.4640(16)	C20	C25	1.4091(15)
C5	C6	1.3981(16)	C21	C22	1.3907(16)
C5	C10	1.4032(16)	C22	C23	1.3887(16)
C6	C7	1.3896(17)	C23	C24	1.4049(16)
C7	C8	1.3882(17)	C24	C25	1.4054(15)
C8	C9	1.3895(16)	C24	C26	1.4877(15)
C9	C10	1.3905(16)	C26	C27	1.4019(16)
C12	C25	1.4722(15)	C26	C31	1.3993(16)
C12	N11	1.3714(14)	C27	C28	1.3854(17)
C12	N13	1.3150(14)	C28	C29	1.3899(17)
C14	C15	1.3963(16)	C29	C30	1.3878(17)

Table S4 Bond Lengths for 221293lt_auto.

Atom	Atom	Length/Å	Atom	Atom	Length/Å
C14	C19	1.4125(16)	C30	C31	1.3873(16)

Table S5 Bond Angles for 221293lt_auto.

Atom	Atom	Atom	Angle/˚	Atom	Atom	Atom	Angle/˚
C10	C1	C2	114.11(9)	C18	C17	C16	121.40(11)
C10	C1	C20	114.91(9)	C17	C18	C19	116.39(11)
C20	C1	C2	104.61(8)	C18	C19	C14	123.40(10)
N11	C1	C2	109.56(9)	N11	C19	C14	104.06(9)
N11	C1	C10	112.83(9)	N11	C19	C18	132.53(10)
N11	C1	C20	99.60(8)	C21	C20	C1	126.40(10)
C3	C2	C1	116.39(9)	C21	C20	C25	122.51(10)
O32	C2	C1	119.68(10)	C25	C20	C1	110.88(9)
O32	C2	C3	123.90(10)	C20	C21	C22	117.48(10)
C4	C3	C2	120.95(10)	C23	C22	C21	120.90(10)

Table S5 Bond Angles for 221293lt_auto.

Atom	Atom	Atom	Angle/˚	Atom	Atom	Atom	Angle/˚
C3	C4	C5	123.07(11)	C22	C23	C24	122.44(10)
C6	C5	C4	120.71(10)	C23	C24	C25	116.54(10)
C6	C5	C10	119.15(10)	C23	C24	C26	119.28(10)
C10	C5	C4	120.07(10)	C25	C24	C26	124.17(10)
C7	C6	C5	120.60(11)	C20	C25	C12	106.43(9)
C8	C7	C6	119.71(10)	C24	C25	C12	133.39(10)
C7	C8	C9	120.40(11)	C24	C25	C20	120.12(10)
C8	C9	C10	120.09(11)	C27	C26	C24	119.71(10)
C5	C10	C1	119.48(10)	C31	C26	C24	122.05(10)
C9	C10	C1	120.48(10)	C31	C26	C27	118.20(10)
C9	C10	C5	120.03(10)	C28	C27	C26	120.99(11)
N11	C12	C25	107.11(9)	C27	C28	C29	120.24(11)
N13	C12	C25	139.23(10)	C30	C29	C28	119.32(11)
N13	C12	N11	113.51(9)	C31	C30	C29	120.70(11)
C15	C14	C19	118.90(10)	C30	C31	C26	120.55(10)
C15	C14	N13	130.22(10)	C12	N11	C1	115.03(9)
N13	C14	C19	110.87(9)	C12	N11	C19	107.73(9)

Table S5 Bond Angles for 221293lt_auto.

Atom	Atom	Atom	Angle/˚	Atom	Atom	Atom	Angle/˚
C16	C15	C14	118.16(11)	C19	N11	C1	134.77(9)
C15	C16	C17	121.70(11)	C12	N13	C14	103.79(9)

Table S6 Torsion Angles for 221293lt_auto.

A	B	C	D	Angle/˚	A	B	C	D	Angle/˚
C1	C2	C3	C4	19.61(15)	C20	C1	C10	C9	80.14(13)
C1	C20	C21	C22	173.87(10)	C20	C1	N11	C12	10.07(11)
C1	C20	C25	C12	2.31(12)	C20	C1	N11	C19	169.55(11)
C1	C20	C25	C24	-175.23(9)	C20	C21	C22	C23	0.35(16)
C2	C1	C10	C5	20.43(14)	C21	C20	C25	C12	177.32(10)
C2	C1	C10	C9	-159.03(10)	C21	C20	C25	C24	-0.22(16)
C2	C1	C20	C21	-68.62(13)	C21	C22	C23	C24	0.18(17)
C2	C1	C20	C25	106.15(10)	C22	C23	C24	C25	-0.72(16)
C2	C1	N11	C12	-99.29(10)	C22	C23	C24	C26	179.10(10)
C2	C1	N11	C19	60.19(15)	C23	C24	C25	C12	-176.03(11)

Table S6 Torsion Angles for 221293lt_auto.

A	B	C	D	Angle/˚	A	B	C	D	Angle/˚
C2	C3	C4	C5	-0.89(17)	C23	C24	C25	C20	0.72(15)
C3	C4	C5	C6	168.67(11)	C23	C24	C26	C27	36.34(15)
C3	C4	C5	C10	-8.34(17)	C23	C24	C26	C31	-141.35(11)
C4	C5	C6	C7	-175.58(10)	C24	C26	C27	C28	-178.45(10)
C4	C5	C10	C1	-2.81(15)	C24	C26	C31	C30	178.57(10)
C4	C5	C10	C9	176.66(10)	C25	C12	N11	C1	-9.36(12)
C5	C6	C7	C8	-1.26(17)	C25	C12	N11	C19	-174.22(9)
C6	C5	C10	C1	-179.87(10)	C25	C12	N13	C14	173.01(13)
C6	C5	C10	C9	-0.40(16)	C25	C20	C21	C22	-0.33(16)
C6	C7	C8	C9	0.01(17)	C25	C24	C26	C27	-143.86(11)
C7	C8	C9	C10	1.04(17)	C25	C24	C26	C31	38.45(16)
C8	C9	C10	C1	178.63(10)	C26	C24	C25	C12	4.17(19)
C8	C9	C10	C5	-0.83(16)	C26	C24	C25	C20	-179.08(10)
C10	C1	C2	C3	-28.50(13)	C26	C27	C28	C29	-0.09(18)

Table S6 Torsion Angles for 221293lt_auto.

A	B	C	D	Angle/˚	A	B	C	D	Angle/˚
C10	C1	C2	O32	153.45(10)	C27	C26	C31	C30	0.85(16)
C10	C1	C20	C21	57.28(15)	C27	C28	C29	C30	0.67(18)
C10	C1	C20	C25	-127.95(10)	C28	C29	C30	C31	-0.50(18)
C10	C1	N11	C12	132.40(10)	C29	C30	C31	C26	-0.27(17)
C10	C1	N11	C19	-68.12(15)	C31	C26	C27	C28	-0.67(16)
C10	C5	C6	C7	1.45(16)	N11	C1	C2	C3	-156.10(9)
C14	C15	C16	C17	1.31(17)	N11	C1	C2	O32	25.86(14)
C14	C19	N11	C1	-162.10(11)	N11	C1	C10	C5	146.33(10)
C14	C19	N11	C12	-1.58(11)	N11	C1	C10	C9	-33.13(14)
C15	C14	C19	C18	-1.90(17)	N11	C1	C20	C21	178.11(10)
C15	C14	C19	N11	179.36(10)	N11	C1	C20	C25	-7.12(11)
C15	C14	N13	C12	-177.87(12)	N11	C12	C25	C20	3.89(12)
C15	C16	C17	C18	-1.05(18)	N11	C12	C25	C24	-179.04(11)
C16	C17	C18	C19	-0.66(17)	N11	C12	N13	C14	-1.94(12)
C17	C18	C19	C14	2.14(16)	N13	C12	C25	C20	-

Table S6 Torsion Angles for 221293lt_auto.

A	B	C	D	Angle/˚	A	B	C	D	Angle/˚
									171.27(13)
C17	C18	C19	N11	-179.52(11)	N13	C12	C25	C24	5.8(2)
C18	C19	N11	C1	19.3(2)	N13	C12	N11	C1	167.19(9)
C18	C19	N11	C12	179.85(12)	N13	C12	N11	C19	2.34(13)
C19	C14	C15	C16	0.11(16)	N13	C14	C15	C16	178.75(11)
C19	C14	N13	C12	0.86(12)	N13	C14	C19	C18	179.21(10)
C20	C1	C2	C3	97.91(11)	N13	C14	C19	N11	0.47(12)
C20	C1	C2	O32	-80.14(12)	O32	C2	C3	C4	-162.43(11)
C20	C1	C10	C5	-100.40(12)					

Table S7 Hydrogen Atom Coordinates (Å×10^4) and Isotropic Displacement Parameters (Å^2×10^3) for 221293lt_auto.

Atom	*x*	*y*	*z*	U(eq)
H3	6902.67	1021.04	2792.79	25
H4	6816.45	1585.06	1537.89	24
H6	7033.97	2765.42	613.36	24

Table S7 Hydrogen Atom Coordinates (Å×10[4]) and Isotropic Displacement Parameters (Å[2]×10[3]) for 221293lt_auto.

Atom	*x*	*y*	*z*	U(eq)
H7	7238.08	4164.32	402.71	26
H8	7243.05	5057	1512.81	26
H9	7047.32	4557.13	2832.37	24
H15	9087.74	4302.77	5832.45	26
H16	10380.46	4082.16	5080.9	29
H17	10324.71	3614.17	3742.03	27
H18	8962.61	3312.65	3115.88	24
H21	5141.78	2537.2	2885.16	23
H22	3836.33	2643.57	3661.08	25
H23	3853	3261.75	4927.97	23
H27	4216.88	3167.32	6267.76	25
H28	4101.19	3792.96	7534.77	29
H29	4941.78	4959.1	7829.52	29
H30	5876.68	5504.44	6833.4	27
H31	5992.49	4887.89	5560.78	23

Experimental

Single crystals of $C_{29}H_{18}N_2O$ **[221293lt_auto]** were **[]**. A suitable crystal was selected and **[]** on a **XtaLAB Synergy R, DW system, HyPix-Arc 150** diffractometer. The crystal was kept at 100.00(10) K during data collection. Using Olex2 [1], the structure was solved with the SHELXT [2] structure solution program using Intrinsic Phasing and refined with the SHELXL [3] refinement package using Least Squares minimisation.

1. Dolomanov, O.V., Bourhis, L.J., Gildea, R.J, Howard, J.A.K. & Puschmann, H. (2009), J. Appl. Cryst. 42, 339-341.
2. Sheldrick, G.M. (2015). Acta Cryst. A71, 3-8.
3. Sheldrick, G.M. (2015). Acta Cryst. C71, 3-8.

Crystal structure determination of [221293lt_auto]

Crystal Data for $C_{29}H_{18}N_2O$ (M =410.45 g/mol): orthorhombic, space group Pbca (no. 61), a = 15.08430(10) Å, b = 16.37780(10) Å, c = 16.4285(2) Å, V = 4058.62(6) Å^3, Z = 8, T = 100.00(10) K, μ(Cu Kα) = 0.644 mm^{-1}, $Dcalc$ = 1.343 g/cm^3, 39977 reflections measured (9.62° ≤ 2Θ ≤ 134.144°), 3627 unique (R_{int} = 0.0293, R_{sigma} = 0.0145) which were used in all calculations. The final R_1 was 0.0315 (I > 2σ(I)) and wR_2 was 0.0788 (all data).

Refinement model description

Number of restraints - 0, number of constraints - unknown.

Details:

```
1. Fixed Uiso
 At 1.2 times of:
  All C(H) groups
2.a Aromatic/amide H refined with riding coordinates:
 C3(H3), C4(H4), C6(H6), C7(H7), C8(H8), C9(H9), C15(H15), C16(H16), C17(H17),
 C18(H18), C21(H21), C22(H22), C23(H23), C27(H27), C28(H28), C29(H29), C30(H30),
  C31(H31)
```

This report has been created with Olex2, compiled on 2022.04.07 svn.rca3783a0 for OlexSys. Please let us know if there are any errors or if you would like to have additional features.

X-ray crystallographic data of compound 5q

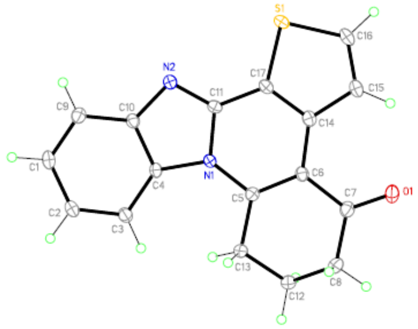

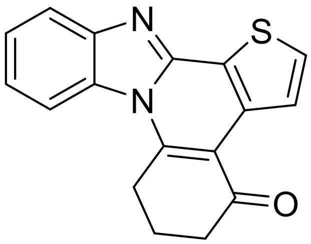

5q

ORTEP diagram of compound 5q. Atomic displacement ellipsoids are drawn at the 50% probability level

Table S8. Crystal data and structure refinement for **5q**.

Identification code	twin5
Empirical formula	C17 H12 N2 O S
Formula weight	292.35
Temperature	100(2) K
Wavelength	0.71073 Å
Crystal system	Triclinic
Space group	P-1

Unit cell dimensions	a = 6.8441(4) Å	α= 66.273(3)°.
	b = 9.3603(6) Å	β= 79.570(3)°.
	c = 11.4250(7) Å	γ = 69.805(2)°.
Volume	628.10(7) $Å^3$	
Z	2	
Density (calculated)	1.546 Mg/m^3	
Absorption coefficient	0.257 mm^{-1}	
F(000)	304	
Crystal size	0.12 x 0.03 x 0.03 mm^3	
Theta range for data collection	1.949 to 26.413°.	
Index ranges	-8<=h<=8, -10<=k<=11, 0<=l<=14	
Reflections collected	2561	
Independent reflections	2561 [R(int) = 0.0216]	
Completeness to theta = 25.242°	99.6 %	
Absorption correction	Semi-empirical from equivalents	
Max. and min. transmission	0.745373 and 0.702549	
Refinement method	Full-matrix least-squares on F^2	
Data / restraints / parameters	2561 / 0 / 191	
Goodness-of-fit on F^2	1.131	

Final R indices [I>2sigma(I)]	R1 = 0.0310, wR2 = 0.0889
R indices (all data)	R1 = 0.0330, wR2 = 0.0911
Extinction coefficient	n/a
Largest diff. peak and hole	0.344 and -0.248 e.$Å^{-3}$

Table S9. Atomic coordinates (x 10^4) and equivalent isotropic displacement parameters (Å^{2}x 10^3) for **5q**. U(eq) is defined as one third of the trace of the orthogonalized U^{ij} tensor.

	x	y	z	U(eq)
S(1)	4423(1)	-4751(1)	1465(1)	17(1)
O(1)	2567(2)	-1561(1)	4620(1)	23(1)
N(1)	2378(2)	199(1)	102(1)	12(1)
N(2)	3255(2)	-1425(2)	-1080(1)	14(1)
C(1)	1948(2)	2544(2)	-3824(1)	17(1)
C(2)	1475(2)	3574(2)	-3133(1)	16(1)
C(3)	1553(2)	2961(2)	-1813(1)	15(1)
C(4)	2114(2)	1256(2)	-1190(1)	12(1)
C(5)	2039(2)	506(2)	1227(1)	12(1)
C(6)	2479(2)	-793(2)	2379(1)	13(1)
C(7)	2302(2)	-464(2)	3567(1)	16(1)
C(8)	1835(3)	1292(2)	3420(2)	21(1)
C(9)	2551(2)	859(2)	-3213(1)	16(1)
C(10)	2652(2)	212(2)	-1881(1)	13(1)

C(11)	3086(2)	-1393(2)	74(1)	12(1)
C(12)	292(3)	2429(2)	2386(1)	20(1)
C(13)	1149(2)	2264(2)	1104(1)	15(1)
C(14)	3198(2)	-2456(2)	2408(1)	14(1)
C(15)	3732(2)	-3986(2)	3470(1)	16(1)
C(16)	4416(2)	-5291(2)	3099(1)	18(1)
C(17)	3493(2)	-2710(2)	1269(1)	14(1)

Table S10. Bond lengths [Å] and angles [°] for **5q**.

S(1)-C(17)	1.7237(14)
S(1)-C(16)	1.7264(15)
O(1)-C(7)	1.2210(19)
N(1)-C(5)	1.3902(18)
N(1)-C(4)	1.4099(17)
N(1)-C(11)	1.4105(17)
N(2)-C(11)	1.3136(18)
N(2)-C(10)	1.3880(18)
C(1)-C(9)	1.384(2)
C(1)-C(2)	1.400(2)
C(1)-H(1)	0.9500
C(2)-C(3)	1.385(2)
C(2)-H(10)	0.9500
C(3)-C(4)	1.4007(19)
C(3)-H(11)	0.9500
C(4)-C(10)	1.408(2)
C(5)-C(6)	1.3794(19)

C(5)-C(13)	1.5007(19)
C(6)-C(14)	1.4492(19)
C(6)-C(7)	1.485(2)
C(7)-C(8)	1.506(2)
C(8)-C(12)	1.518(2)
C(8)-H(3)	0.9900
C(8)-H(2)	0.9900
C(9)-C(10)	1.398(2)
C(9)-H(12)	0.9500
C(11)-C(17)	1.4174(19)
C(12)-C(13)	1.5238(19)
C(12)-H(4)	0.9900
C(12)-H(5)	0.9900
C(13)-H(6)	0.9900
C(13)-H(7)	0.9900
C(14)-C(17)	1.383(2)
C(14)-C(15)	1.434(2)
C(15)-C(16)	1.357(2)
C(15)-H(9)	0.9500

C(16)-H(8)	0.9500
C(17)-S(1)-C(16)	90.14(7)
C(5)-N(1)-C(4)	131.78(12)
C(5)-N(1)-C(11)	123.26(11)
C(4)-N(1)-C(11)	104.95(11)
C(11)-N(2)-C(10)	104.47(12)
C(9)-C(1)-C(2)	121.13(14)
C(9)-C(1)-H(1)	119.4
C(2)-C(1)-H(1)	119.4
C(3)-C(2)-C(1)	121.87(14)
C(3)-C(2)-H(10)	119.1
C(1)-C(2)-H(10)	119.1
C(2)-C(3)-C(4)	117.09(14)
C(2)-C(3)-H(11)	121.5
C(4)-C(3)-H(11)	121.5
C(3)-C(4)-C(10)	121.36(13)
C(3)-C(4)-N(1)	133.68(13)
C(10)-C(4)-N(1)	104.91(11)

C(6)-C(5)-N(1)	119.24(13)
C(6)-C(5)-C(13)	123.92(13)
N(1)-C(5)-C(13)	116.82(12)
C(5)-C(6)-C(14)	119.85(13)
C(5)-C(6)-C(7)	119.29(13)
C(14)-C(6)-C(7)	120.80(13)
O(1)-C(7)-C(6)	122.14(14)
O(1)-C(7)-C(8)	120.81(13)
C(6)-C(7)-C(8)	117.03(13)
C(7)-C(8)-C(12)	110.92(13)
C(7)-C(8)-H(3)	109.5
C(12)-C(8)-H(3)	109.5
C(7)-C(8)-H(2)	109.5
C(12)-C(8)-H(2)	109.5
H(3)-C(8)-H(2)	108.0
C(1)-C(9)-C(10)	118.02(14)
C(1)-C(9)-H(12)	121.0
C(10)-C(9)-H(12)	121.0
N(2)-C(10)-C(9)	127.83(13)

N(2)-C(10)-C(4)	111.68(12)
C(9)-C(10)-C(4)	120.49(13)
N(2)-C(11)-N(1)	113.98(12)
N(2)-C(11)-C(17)	129.46(13)
N(1)-C(11)-C(17)	116.56(12)
C(8)-C(12)-C(13)	110.70(13)
C(8)-C(12)-H(4)	109.5
C(13)-C(12)-H(4)	109.5
C(8)-C(12)-H(5)	109.5
C(13)-C(12)-H(5)	109.5
H(4)-C(12)-H(5)	108.1
C(5)-C(13)-C(12)	111.95(12)
C(5)-C(13)-H(6)	109.2
C(12)-C(13)-H(6)	109.2
C(5)-C(13)-H(7)	109.2
C(12)-C(13)-H(7)	109.2
H(6)-C(13)-H(7)	107.9
C(17)-C(14)-C(15)	110.70(13)
C(17)-C(14)-C(6)	119.11(13)

C(15)-C(14)-C(6)	130.18(13)
C(16)-C(15)-C(14)	112.49(13)
C(16)-C(15)-H(9)	123.8
C(14)-C(15)-H(9)	123.8
C(15)-C(16)-S(1)	113.34(11)
C(15)-C(16)-H(8)	123.3
S(1)-C(16)-H(8)	123.3
C(14)-C(17)-C(11)	121.93(13)
C(14)-C(17)-S(1)	113.34(10)
C(11)-C(17)-S(1)	124.73(11)

Symmetry transformations used to generate equivalent atoms:

Table S11. Anisotropic displacement parameters ($Å^2 x 10^3$) for **5q**. The anisotropic displacement factor exponent takes the form: $-2\pi^2[h^2 a^{*2}U^{11} + ... + 2 h k a^* b^* U^{12}]$

	U^{11}	U^{22}	U^{33}	U^{23}	U^{13}	U^{12}
S(1)	21(1)	10(1)	16(1)	-3(1)	-3(1)	-2(1)
O(1)	29(1)	21(1)	12(1)	-4(1)	-3(1)	-2(1)
N(1)	13(1)	10(1)	10(1)	-2(1)	-2(1)	-3(1)
N(2)	17(1)	13(1)	12(1)	-4(1)	-2(1)	-4(1)
C(1)	18(1)	19(1)	11(1)	-1(1)	-2(1)	-7(1)
C(2)	18(1)	14(1)	14(1)	-1(1)	-2(1)	-4(1)
C(3)	16(1)	14(1)	14(1)	-4(1)	-1(1)	-4(1)
C(4)	11(1)	14(1)	10(1)	-3(1)	-2(1)	-4(1)
C(5)	11(1)	14(1)	13(1)	-5(1)	-1(1)	-3(1)
C(6)	12(1)	14(1)	12(1)	-4(1)	-2(1)	-3(1)
C(7)	14(1)	18(1)	14(1)	-6(1)	-2(1)	-2(1)
C(8)	29(1)	20(1)	16(1)	-9(1)	-4(1)	-6(1)
C(9)	17(1)	18(1)	14(1)	-6(1)	0(1)	-5(1)
C(10)	12(1)	13(1)	13(1)	-4(1)	-1(1)	-3(1)

C(11)	11(1)	11(1)	15(1)	-4(1)	-1(1)	-3(1)
C(12)	26(1)	14(1)	15(1)	-6(1)	-2(1)	0(1)
C(13)	18(1)	12(1)	14(1)	-5(1)	-2(1)	-3(1)
C(14)	11(1)	14(1)	14(1)	-3(1)	-2(1)	-4(1)
C(15)	16(1)	16(1)	14(1)	-1(1)	-3(1)	-4(1)
C(16)	19(1)	13(1)	16(1)	1(1)	-4(1)	-4(1)
C(17)	13(1)	11(1)	14(1)	-2(1)	-2(1)	-3(1)

Table S12. Hydrogen coordinates (x 10^4) and isotropic displacement parameters ($Å^2$x 10 ^{3}) for **5q**.

	x	y	z	U(eq)
H(1)	1854	3011	-4727	21
H(10)	1090	4726	-3582	20
H(11)	1239	3666	-1350	18
H(3)	3143	1594	3193	25
H(2)	1252	1419	4243	25
H(12)	2886	162	-3684	19
H(4)	8	3575	2307	24
H(5)	-1038	2160	2631	24
H(6)	17	2825	495	18
H(7)	2249	2810	752	18
H(9)	3620	-4075	4336	19
H(8)	4848	-6393	3681	22
